# Linking genetic, metabolic, and phenotypic diversity among *Saccharomyces cerevisiae* strains using multi-omics associations

**DOI:** 10.1093/gigascience/giz015

**Published:** 2019-01-31

**Authors:** Kang Kang, Basti Bergdahl, Daniel Machado, Laura Dato, Ting-Li Han, Jun Li, Silas Villas-Boas, Markus J Herrgård, Jochen Förster, Gianni Panagiotou

**Affiliations:** 1 Systems Biology & Bioinformatics Group, School of Biological Sciences, The University of Hong Kong, Hong Kong S.A.R., China; 2 Systems Biology & Bioinformatics Unit, Leibniz Institute for Natural Product Research and Infection Biology – Hans Knöll Institute, Jena, Germany; 3 The Novo Nordisk Foundation Center for Biosustainability, Technical University of Denmark, Kongens Lyngby, Denmark; 4 Department of Biological Engineering, School of Engineering, University of Minho, Braga, Portugal; 5 The European Molecular Biology Laboratory (EMBL), Heidelberg, Germany; 6 Centre for Microbial Innovation, School of Biological Sciences, University of Auckland, Auckland, New Zealand; 8 Department of Microbiology, Li Ka Shing Faculty of Medicine, The University of Hong Kong, Hong Kong S.A.R., China

**Keywords:** *Saccharomyces cerevisiae*, multi-omic study, platform strain, stress resistance, geno-to-phenotype association

## Abstract

**Background:**

The selection of bioengineering platform strains and engineering strategies to improve the stress resistance of *Saccharomyces cerevisiae* remains a pressing need in bio-based chemical production. Thus, a systematic effort to exploit genotypic and phenotypic diversity to boost yeast's industrial value is still urgently needed.

**Results:**

We analyzed 5,400 growth curves obtained from 36 *S. cerevisiae* strains and comprehensively profiled their resistances against 13 industrially relevant stresses. We observed that bioethanol and brewing strains exhibit higher resistance against acidic conditions; however, plant isolates tend to have a wider range of resistance, which may be associated with their metabolome and fluxome signatures in the tricarboxylic acid cycle and fatty acid metabolism. By deep genomic sequencing, we found that industrial strains have more genomic duplications especially affecting transcription factors, showing that they result from disparate evolutionary paths in comparison with the environmental strains, which have more indels, gene deletions, and strain-specific genes. Genome-wide association studies coupled with protein-protein interaction networks uncovered novel genetic determinants of stress resistances.

**Conclusions:**

These resistance-related engineering targets and strain rankings provide a valuable source for engineering significantly improved industrial platform strains.

## Introduction

We are facing a paradigm shift, in which our economy and industrial processes need to exchange current petroleum-based technologies for new and sustainable biotechnologies. The transition to a bio-based economy has already begun as several products have reached commercial production scale, e.g., cellulosic ethanol, succinic acid, lactic acid, acetic acid, itaconic acid, 1,3-propanediol, 1,4-butanediol, farnesene, and acetone–*n*-butanol–ethanol) [1, 2]. To build a microbial cell factory to convert sugars or other carbon sources into bio-products, the first step is to select a host organism with metabolic and physiological properties suitable for the intended bioprocess. These properties include tolerance to substrates, products, and byproducts in high concentration, and resistance to abiotic stresses including low/high pH, high temperature, osmotic stress, etc., during industrial fermentation.

Most current metabolic engineering projects are carried out using laboratory strains of bacteria [3] or yeast [4] as the starting host. Although such strains are easily manipulated genetically, they do not always meet the requirements set by stressful industrial fermentation conditions [5]. To improve the stress resistance of the host organism, different strategies have been adopted: (i) comparative transcriptomic or proteomic studies [6], (ii) directed evolution of the host genome under extreme conditions [7], and (iii) knowledge-based engineering [8]. Nevertheless, all these methods neglect the abundant genetic diversity present in environmental or industrial strains by focusing on narrow genetic resources (limited gene candidates, random mutations, and simple genetic changes) in a single host laboratory strain. Global screening incorporating genetic variation present in a broader strain collection could significantly speed up development of improved industrial production strains.

Herein, we aim to address the aforementioned scientific and engineering challenges through a *Sacharomyces cerevisiae* population multi-omics approach. We explored the diversity of 36 industrial, environmental, clinical, and laboratory strains of *S. cerevisiae* regarding the genetic composition, metabolic properties—metabolite levels and predicted flux distributions—and resistance to 13 industrially relevant stress conditions. To characterize and rank the strains’ industrial values in stress resistance, we defined 2 scores, robustness and performance, calculated from 5 growth parameters with variable weights to meet different bioengineering purposes. The outcomes of this study are as follows: (i) identification of strains with multiple or specific stress resistance as potential platform strains for cell factory construction, (ii) construction of strain-specific metabolic models explaining the divergence in metabolic phenotypes, and (iii) establishing novel resistance phenotype-genotype links including suggestions of potential engineering targets to boost industrially relevant phenotypic properties.

## Materials and Methods

### Data Description

We performed a multi-omic study on a collection of 36 *S. cerevisiae* strains, including natural and industrial strains with different geographical origins. The genomes of these strains were sequenced with high depths (>50×), and the variant profiles and phylogenetic tree were constructed. In addition, intra- and extracellular metabolomes were measured. Strain-specific genome-scale metabolic models (GSMMs) were constructed to predict the fluxomes of all the strains. Regarding phenome, the strain collection was exposed to 13 industrially relevant stresses (including low pH, high temperature, and various inhibitory compounds) in different inhibitory levels. We analyzed more than 5400 growth curves to score the strains on 2 phenotypic parameters: robustness and performance. Genome-wide association studies (GWAS) were performed to establish the geno-to-phenotype associations, and protein-protein interaction network (PPIN) modules associated to the stress resistance were built. Suggestions for potential engineering targets to improve the stress resistances of the bioengineering platform strain were given based on the multi-omic study.

### Yeast strain collection storage

A collection of 36 *S. cerevisiae* strains were studied in this research, and the details are summarized in Supplemental Note S1. The strains were stored at −80°C in cryogenic tubes containing yeast extract peptone dextrose (YPD) medium with 20% (vol/vol) glycerol. From the stock tubes, a sterile inoculation loop was used to transfer cells onto YPD plates. Plates were incubated for 48 h before cells were used for precultivation.

### Medium for precultures and under various stress conditions

Precultures of yeast strains were grown in a defined mineral medium containing 7.5 g/L (NH_4_)_2_SO_4_, 14.4 g/L KH_2_PO_4_, 0.5 g/L MgSO_4_•7H_2_O, 2 mL/L of trace element solution, 1 mL/L vitamin solution (prepared according to Verduyn et al. [9]), and 20 g/L glucose. The pH of the salts together with trace elements was adjusted to 6.0 with NaOH before autoclaving. The glucose solution was autoclaved separately before being added to the salt solution together with the filter-sterilized vitamin solution. Precultures were made in 24-deepwell plates (CR1424, Enzyscreen, The Netherlands) containing 1 mL mineral medium in each well. A single yeast colony was inoculated from a YPD agar plate and grown for 20 h at 30°C and 300 rpm in an incubator with 51 mm shaking orbit. Media for cultivation under various stress conditions are introduced in detail in Supplemental Note S2.

### Cultivation in the Growth Profiler 1152 and data processing

Yeast strains were precultivated as described above and harvested by centrifugation and then inoculated to 96-well microplates. The inoculated plates were then placed in the Growth Profiler 1152, and growth was monitored for ∼66 h. Details of cultivation, biological replicates, and data processing are provided in Supplemental Note S2.

### Investigation of carbon and nitrogen source utilization with Biolog Phenotype Microarray

Yeast strains CEN.PK113-7D, S288C, and Ethanol Red were pregrown in 50-mL conical tubes using 5 mL YPD medium at 30°C and 280 rpm for 16 h. These precultures were used to inoculate 250-mL shake flasks with 25 mL YPD medium at an initial optical density (OD) of 0.2. The strains were cultivated until the OD reached ∼1 (∼5 h), at which point the cells were washed twice in sterile water. After the final wash, the cells were concentrated to an OD of 4.2 by diluting with an appropriate volume of sterile water. The cell suspensions were diluted 48-fold when added to the media specific for PM1-3, resulting in a starting OD of 0.0875. After inoculation, the PM plates were placed in the OmniLog incubator at 30°C and the development of the colored dye was measured every 15 min for 83 h. Data files were converted and exported to Excel using the dedicated software from the supplier.

### Intra- and extracellular metabolome screening

Intracellular and extracellular metabolites of the yeast strains growing in glucose media were profiled using gas chromatography–mass spectrometry (GC-MS). Metabolite identification and normalization of GC-MS data was performed using the Automated Mass Spectral Deconvolution and Identification System software. Intra- and extracellular metabolites were assigned to different compound classes, and the intracellular metabolites were also assigned to different pathways and pathway groups. The experimental protocol is described in detail in Supplemental Note S3.

### Genome sequencing and estimation of strain ploidy

The genomes of the 36 *S. cerevisiae* strains were sequenced using the Illumina MiSeq or HiSeq 2000 platform. Paired-end sequencing libraries with 350 bp insert size were prepared with the TruSeq Nano DNA kit and sequenced with either 150 or 250 nt read length. Data quality control and filtering were performed by FastQC. Strain ploidy was determined by relative comparison of the DNA amount of the G_0_-G_1_ gated population of the target strains with reference *S. cerevisiae* strains of known ploidy, measured by flow cytometry following the procedure previously described [10]. To avoid the misidentification of aneuploid strains as polyploid strains in the flow cytometry analysis, the ploidy estimation results were verified by the allele frequencies of the heterozygous single-nucleotide polymorphisms (SNPs)/indels.

### Read mapping, variant calling, and annotation

Reads were mapped to the S288c reference genome (SGD release 64 [11]) using BWA (v0.7.12, module *mem*)(RRID:SCR_010910)[12]. A minimum coverage of 50×, after filtration, was set as requirement for each strain. SNPs/indels were identified and filtered using the Genome Analysis Toolkit, version 3.4 [13, 14], with the sequential steps to include RealignerTargetCreator, IndelRealigner, HaplotypeCaller (with parameter “-rf BadCigar”), and VariantFiltration (with parameter “–filterExpression 'DP < 10 || QD < 2.0 || FS > 60.0'”). SNPs and indels were annotated by SnpEff using the *S. cerevisiae* database version EF4.69 [15]. The 800 bp upstream regions of the genes were included as potential regulatory sequences. Copy number variations (CNVs) were detected using Control-FreeC [16], whereas the reads of the S288c haploid strain were used as the reference genome. Genes fully covered by the CNV regions were labeled as affected genes. If a gene was partially overlapped with a CNV region, a gain event would not be assigned due to the incompleteness of the obtained copies, while a loss or deletion event (when the copy number of the CNV region is 0) will be assigned to this gene because at least 1 copy of the gene was truncated.

### Population structure analysis

The consensus sequences of the 36 strains were generated using the Genome Analysis Toolkit (GATK, RRID:SCR_001876)[14] based on the SNP set. Protein sequences were translated from the open reading frames (ORFs) and used for the neighbor-joining tree building by TreeBest [17].

### *De novo* assembly and ORF prediction

*De novo* assemblies were performed with Newbler, version 2.8, using the default parameters. To identify the potential novel genes in the yeast population, compared with the reference genome S288c, ORF predictions were performed with the yeast genome annotation pipeline (YGAP) [18] based on the *de novo* assemblies. For the genes for which no S288c homologous gene was annotated in YGAP, we further extracted the sequences and searched against the NCBI nr protein data set [19] using BLASTX [20]. The ORFs with at least 1 valid hit to S288c (identity ≥95% and *E* < 1e–5) were removed from the potential novel gene list and treated as misidentifications of the YGAP pipeline.

### Strain-specific GSMM construction

Strain-specific GSMMs were constructed from the starting model iMM904 [21]. Severe mutations that were consequences of gene truncation, elongation, or deletion were categorized into different severity levels, as well as the new reactions introduced by non-S288c genes (Supplemental Note S4). Reactions were annotated by the UniProt database [22] and MetaNetX database [23]. The mixed integer linear programming algorithm was applied to build the strain-specific models (Supplemental Note S4). Carbon and nitrogen source utilization and fluxes were simulated with the FRAMED package using Gurobi 6.5 (Supplemental Note S4).

### GWAS for resistance rankings

GWAS were carried out for SNPs/indels and CNVs, respectively (referred to as SNP-based and CNV-based GWAS). Only SNPs/indels in core-genome regions and with minor-allele frequency >5% were used in the SNP-based GWAS. CNV markers were defined due to the overlap relationships and were used for gain and loss events separately. Strain rankings were used as phenotypic values. The mixed-model–based method efficient mixed model association [24] was applied as the main algorithm in GWAS. Details of the core-GWAS markers, CNV marker identification, transformation of genotypic values, linkage disequilibrium (LD) block identification, and *P* value assignment for genes are described in detail in Supplemental Note S5. Different significance cutoffs for SNPs/gain/loss markers were set according to the departure of the observed *P* value from the predicted *P* value distribution (Supplemental Note S5).

### Gene categories used in genotyping and GWAS

The basic gene information was acquired from SGD [11], including gene ID, symbol name, the ORF type (verified, uncharacterized, or dubious), and Enzyme Commission number. The transcription factor (TF) list, with the superfamily classification, and the regulatory relationships were collected from SGD [11] and YEASTRACT [25]. The GO-Slim terms were acquired from SGD [11]. The metabolic pathways (YeastCyc) were downloaded from SGD [11] and MetaCyc [26]. The phenotype-associated gene lists were obtained from the yeast phenotype ontology from SGD [11]. Among the gene and phenotype association entries, only the entries with positive or negative effects on phenotype were retained; those with neutral or unclear consequence were removed. When applied with GWAS profiles, the SGD entries were reclassified according to the phenotype classification and chemicals used (Supplemental Note S5).

### The discovery of the protein-protein interaction modules from the SGD gene list and GWAS profile

The PPIN of *S. cerevisiae* was acquired from the STRING [27] database, and regulatory modules were computed by ModuleDiscoverer [28] for both SGD gene lists and GWAS profiles (see details in Supplemental Note S5).

### Statistical analysis

All statistical analyses were performed in R. For comparative analysis between industrial and environmental strains, Wilcoxon rank sum tests were performed. For comparative analysis among different strain subcategorises, Kruskal-Wallis tests were performed. For multiple comparisons, the Benjamini-Hochberg procedure was used to calculate the false discovery rate (FDR), and FDR < 0.05 or FDR < 0.1 was used as the significance cutoff. Spearman tests with Bonferroni adjustment were performed to correlate the genotypic or phenotypic features, where FDR < 0.05 was used as the significance cutoff. The mixed-model–based method efficient mixed model association [24] was applied as the main algorithm in GWAS.

### Data visualization

R [29] and corresponding packages including ggplot2, ggtree, heatmap.plus, and matplotlib were used for illustration of the statistical results. Cytoscape 3.6.0 [30] was used to visualize the analyses incorporating networks and topology.

## Results

### Systematic screening of resistance to multiple bioprocessing-relevant stress conditions

**Figure 1:**
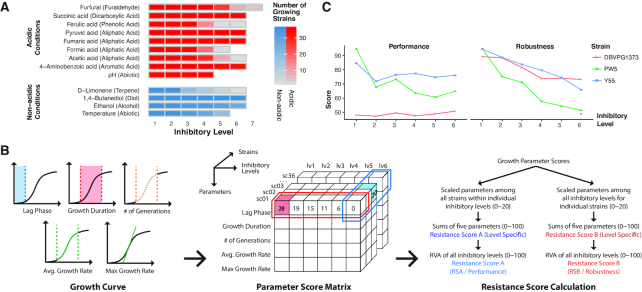
The stress conditions and resistance scores. (**A**) The 13 stress conditions and 4 to 7 inhibitory levels in each condition. The colored cells indicate the number of strains that could grow in each experimental setting. (**B**) Calculation of the resistance scores (performance and robustness) based on 5 growth parameters. (**C**) The inhibitory level–specific performance and robustness scores of 3 strains under the 1,4-butanediol condition: Y55: high ranking in both scores; DBVPG1373: good robustness but poor performance; PW5: good performance but bad robustness. RVA: rank variability analysis.

**Figure 2:**
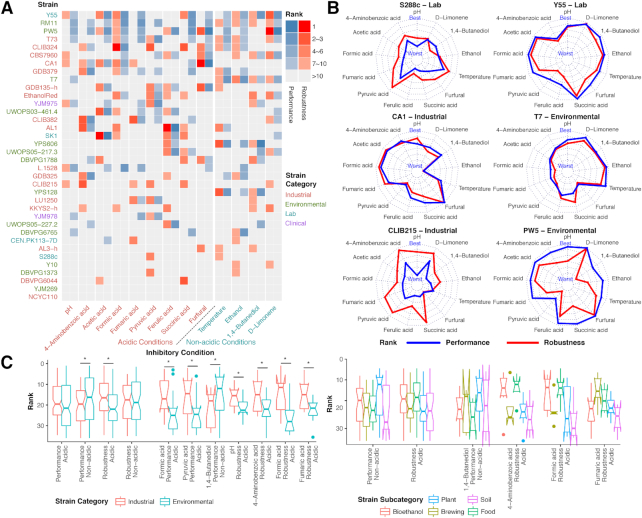
The physiological characterization of various *S. cerevisiae* strains. (**A**) The performance and robustness ranking values in all conditions from selected cases with different patterns. (**B**) The performance and robustness rankings for all strains in all conditions. Strains were sorted by their frequencies to be ranked in the top 10 in all rankings. (**C**) The comparisons of different strain categories and subcategories in multiple resistance rankings. In the industrial vs. environmental comparisons, the asterisk indicates a significant difference (Wilcoxon rank sum test, FDR < 0.1).

In the present study, we used a collection of 36 *S. cerevisiae* strains from various geographic and isolation origins (Supplemental Note S1). Strains were classified into 4 types: 17 industrial strains (from ethanol production, food, and brewing industries), 13 environmental strains (isolated from soil, plants, and animals), 4 laboratory strains, and 2 clinical strains (vaginal isolates). To investigate the phenotypic diversity in the strain collection, all strains were exposed to 13 different stress conditions including 11 inhibitory compounds, acidic pH, and high temperature (Fig. 1a). Acidic pH and the conditions using acids as inhibitory compounds were classified as acidic conditions. In each condition, the cells were exposed to the inhibitory compound at between 4 and 7 distinct levels, resulting in different numbers of strains that exhibited measurable growth (Fig. 1a and Supplemental Note S2). In total, we analyzed more than 5400 growth curves (Dataset S7), and 5 growth parameters were extracted from each growth curve to score the strains in 2 phenotypic traits: robustness and performance (Fig. 1b, Supplemental Note S2, and Dataset S1). Briefly, robustness measures the ability of the strain to withstand increasing inhibitory levels whereas performance measures how well a particular strain compares with other strains in a particular stress condition. The resistance scores were calculated in each inhibitory level and then integrated as final strain rankings for each condition (Fig. 2a and b and Fig. S1, Supplemental Note S2). For instance, strain DBVPG1373 had poor performance scores under 1,4-butanediol compared with other strains but had high robustness according to its ability to maintain its growth parameters when inhibitory levels increased; while PW5 is a counterexample with high performance but low robustness score due to the sharp decrease in fitness when the inhibitory levels increased (Fig. 1c).

Strain Y55, a laboratory strain isolated from grape, was identified as the most resistant strain to multiple conditions, followed by 2 plant strains, RM11 and PW5 (Fig. 2a). These strains with plant origins could be potentially used as platform strain candidates but have seldom been considered in previous bioengineering endeavors [31]. Interestingly, 6 of the top 10 strains are industrial strains (3 bioethanol strains), but nonetheless, the industrial strains are not significantly better than environmental strains overall in multiple stress resistances. The 2 most commonly used laboratory strains, CEN.PK113-7D and S288c, obtained low ranking values (>6) in most conditions, with the only notable exceptions being their resistance to fumaric and pyruvic acids, respectively (Fig. 2b).

Furthermore, diverse resistance patterns were observed for different strains (Fig. 2b), which could also assist the host selection for specific production purposes: e.g., S288c and Y55 are 2 laboratory strains with overall bad and good universal resistance, respectively; CA1, a Brazilian bioethanol strain, shows high resistance to most acidic conditions, while the plant strain T7 is only resistant to nonacidic conditions; another bioethanol strain, CLIB215, has much higher robustness than performance rankings in most conditions; on the contrary, the performance overweighs robustness for the plant strains PW5 and RM11. When comparisons were made between industrial and environmental strains (Brazilian bioethanol strains binned as 1 candidate, Supplemental Note S1; Wilcoxon rank sum test), the industrial strains showed noticeably higher rankings in robustness against acidic conditions (*P* = 6.5e–4, also significant in 4 individual rankings), while the environmental strains scored better in performance under nonacidic conditions (*P* = 4.0e–2, and also significant in 1 individual ranking under 1,4-butanediol) (Fig. 2c). Zooming into subcategories of the strains, bioethanol and food strains but not brewing ones had higher robustness against acidic conditions (including low pH, formic acid, and 4−aminobenzoic acid as significant individual rankings), while the performance under nonacidic conditions was led by plant but not soil strains (Kruskal-Wallis test, FDR < 0.1). Different industrial strain subcategories also performed differently in divergent conditions: for instance, bioethanol and food strains showed higher resistances to multiple conditions such as formic acid and 4-aminobenzoic acid, while brewing strains showed high robustness against fumaric acid.

With the objective of presenting the concept of how strains’ physiological traits could be ranked, the above results were based on giving equal weights to the 5 growth parameters in the calculation of the robustness and performance scores. To gain further insights into the sensitivities of the rankings to weighting different parameters, parameter influence analysis was performed (Supplemental Note S2). A case study showed that 2 strains with significant growth curve differences could still have close rank positions when giving specific parameter weights (e.g., the performance ranks of Y55 and YPS128 under 1,4-butanediol; Fig. S2). Such information can be useful when selecting a host strain for a specific process by setting different weights to different growth parameters, e.g., lag phase would be a less important selection criterion for a continuous fermentation process and thus be given a low weight; however, in a batch fermentation process, lag phase duration becomes critical and could be assigned a higher weight. Besides parameter selection and weighting, our phenotypic scoring method could also be expanded to different industrially relevant processes with customized setups: e.g., different medium composition and aeration condition, which is not limited to the standard medium and condition used in this proof-of-concept study.

### Metabolomic profiling and correlations between metabolomics and stress resistance phenotypes

**Figure 3:**
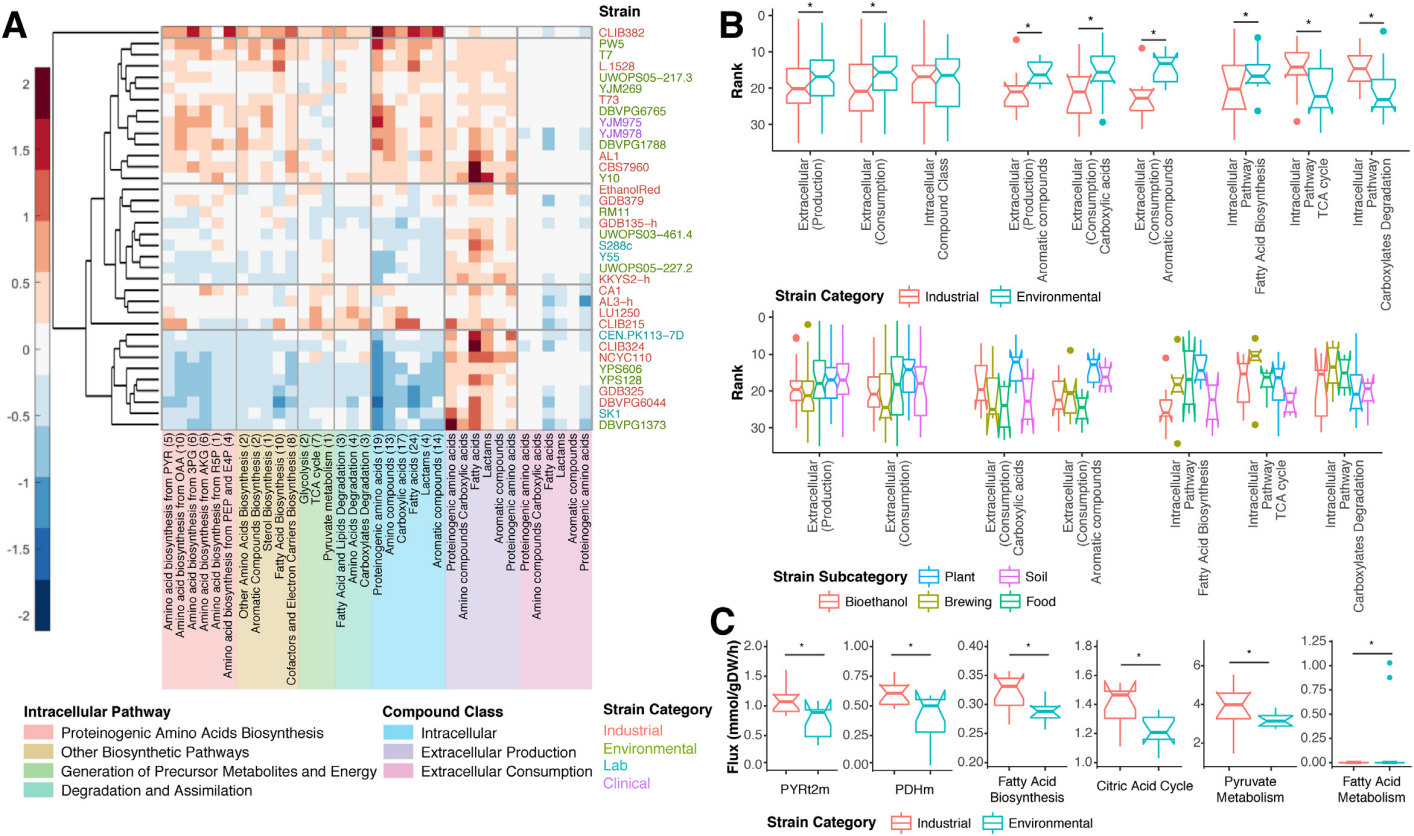
The metabolomic profiles of various isolated strains of *S. cerevisiae*. (**A**) The metabolome of the yeast strains growing exponentially on glucose, and the consumed/excreted metabolites from the spent medium. The colors represent the combined contribution of metabolites in a pathway or compound class (Supplemental Note S3). For intracellular metabolites, red and blue intensities indicate the extent to which the group contains a majority of metabolites that are above or below the mean, respectively. For extracellular metabolites, red indicates a high production level and blue indicates a high degree of consumption. Numbers in parentheses indicate total number of metabolites in the group. Hierarchical clustering was applied to the group variables using Euclidian distance between the strains. For data normalization before visualization, see details in Supplemental Note S3. (**B**) The comparisons of the rankings of metabolome groups, compound classes, or metabolic pathways among different strain categories and subcategories. (**C**) The comparisons of the key fluxes and the fluxes in metabolic subsystems between industrial and environmental strains. PYRt2m: pyruvate mitochondrial transport via proton symport, PDHm: pyruvate dehydrogenase. In the industrial vs. environmental comparisons, the asterisk indicates a significant difference (Wilcoxon rank sum test, FDR < 0.1).

The metabolic characteristics of all strains were investigated by determining both intra- and extracellular metabolomes by GC-MS (Supplemental Note S3). In both analyses, 79 metabolites were identified and quantified in terms of relative abundance (Dataset S2). The metabolomic data were used to group the yeast strains according to metabolite abundance in relevant pathways and/or compound classes (Fig. 3a, Dataset S2). Overall, negative correlations could be observed between the intra- and extracellular metabolite abundances (Fig. 3a and S3). Significant negative correlations were captured between the extracellular abundance of proteinogenic amino acids and all intracellular compound classes except fatty acids (Spearman correlation test with Bonferroni adjustment, FDR < 0.05). The analysis also showed that the industrial strains (particularly bioethanol and brewing strains) had noticeably lower extracellular metabolite levels than the environmental strains, especially for aromatic compounds and carboxylic acids (Wilcoxon rank sum test, FDR < 0.1; Fig. 3b). The greatest difference was actually observed in the carboxylic acids that could be imported and consumed as carbon sources by yeast cells. Industrial strains, and especially brewing strains, show generally high intracellular concentrations in the carboxylate degradation and tricarboxylic acid cycle (TCA cycle) pathways (Wilcoxon rank sum test, FDR < 0.1). Conversely, higher intracellular levels were observed for fatty acid biosynthesis for plant strains (Fig. 3b). Interestingly, as the major membrane lipid component, fatty acids (saturated fatty acids in particular) are highly associated with nonacidic stress response (ethanol, salt, oxidative and thermal stresses) in plants and fungi [32]. Thus, in the resistance to nonacidic conditions such as alcohol and heat, plant strains could outperform the industrial ones due to the biosynthetic activity, cellular abundances, and composition of intracellular fatty acids, which aligns well with our observations in resistance scores (Fig. 2c).

### Genome diversity among industrial and natural *S. cerevisiae* strains

**Figure 4:**
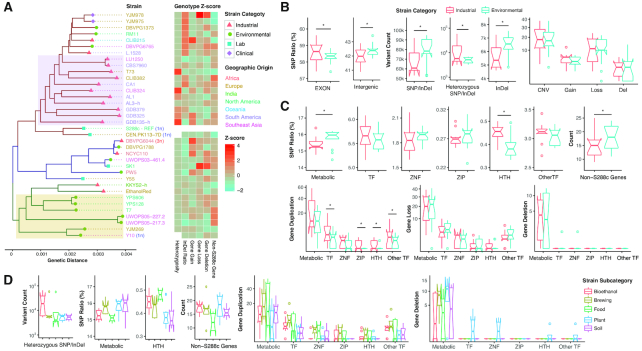
The genetic characterization of various *S. cerevisiae* isolated strains. (**A**) A phylogenetic tree of all the strains was constructed on the basis of identified SNPs/indels and shows 3 main clades. The strain functional class, geographic origin, and determined ploidy (for nondiploid strains) are indicated. The genetic features were normalized to *Z* scores and are illustrated in the heat map. (**B-C**) The comparisons between the industrial and environmental strains on the number of variants **(B)** and the ratio of variants in specific gene sets (for SNPs/indels) or the number of non-S288c genes and genes affected by CNVs. **(C)**. Asterisks indicate a significant difference (Wilcoxon rank sum test, *P* < 0.05). (**D**) The comparisons among different strain subcategories on the number of variants, SNP ratio in different gene sets, number of non-S288c genes, and genes affected by CNVs. ZIP: zipper superfamily; ZNF: zinc finger superfamily.

We sequenced the genomes of all 36 strains to a minimum depth of 52×, with a median of 96×. By using S288c as the reference genome, we identified 342,325 SNP loci, 19,347 small insertion, and 17,457 small deletion (indel) loci among all strains (Table S1 and Dataset S3), with a average SNP/indel count of 68,928 per strain, which was comparable to previous studies [33–35]. In the constructed phylogenetic tree, 3 major clades were discovered (Fig. 4a). We could observe an obvious enrichment of industrial strains in the clade of S288c (13 of 20 strains [65%]). Notably, all 7 strains isolated from the Brazilian bioethanol industry with distinct origins and genotypic categories [36] were clustered in this branch (shaded purple in Fig. 4a). This suggests the possibility of common ancestors, while the long pairwise genetic distances also indicate high divergences in their strain-specific genetic makeups and evolutionary paths. All 6 strains with a high heterozygosity rate, including CLIB324, GDB135-h, T73 (∼97.7– 98.3%, brewing or bakery strains), AL1, GDB325, and GDB379 (∼42.1–52.4%, all bioethanol strains), were present in the same subgroup, while all the other nonhaploid strains’ SNPs/indels were generally in homozygous form (heterozygosity rate <15%). These 6 industrial strains showed some unique resistance to acids: CLIB324 was the strain most resistant (in both performance and robustness) to formic acid and the most robust against pyruvic acid; T73 showed the highest performance under pyruvic acid whereas AL1 was the most resistant to ferulic acid (Fig. 2a). More interestingly, all 7 strains in the other subgroup of the same clade (YJM978–L.1528) were also among the top 8 strains with highest indel rates (>9.0%). On the contrary, more environmental strains were found in the clade of T7 (7 of 9 strains [78%], within yellow shade in Fig. 4a). The SK1 clade was the most divergent cluster. Y55 and PW5, 2 of the top 3 strains with universal resistance, locate in this cluster with long genetic distances from other strains. The only 2 industrial strains in this cluster, DBVPG6044 and NCYC110, had generally poor stress resistance (except DBVPG6044’s robustness against succinic acid) (Fig. 2a), distinguishing them from Y55 and PW5 from the same clade.

The analysis of CNVs resulted in the identification of 2916 unique CNV-affected genes in total (Table S1 and Dataset S3), while most of the large-scale CNVs are located in subtelomeric regions, in agreement with a recent study [37]. Chromosome I duplication was discovered in 5 strains (4 environmental and 1 industrial), while all other strain-specific chromosome-scale duplications were captured in industrial strains (Fig. S4a): NCYC110, chrV; CLIB215, chrXII; GDB135-h, chrIII; and GDB325, chrVI. PW5 (environmental) and DBVPG6044 (industrial, triploid) are also the 2 strains that have a large number of duplicated genes (>200). In addition, extremely high copy numbers of tandem repeated segments (>10) were observed in 7 strains, with 4 of them being bioethanol strains (AL1, 82× in chrI; CLIB382, 11× in chrVII; ethanol red, 18× in chrX; GDB325, 34× in chrIX), together with 1 food (KKYS2-h, 13× in chrVI), 1 plant (RM11, 22× in chrVII), and 1 clinical (YJM975, 701× in chrX and 19× in chrXIV) strain. Except NCYC110, all 11 aforementioned strains had good resistance (especially robustness) to 1 or more acidic conditions (Fig. 2a). In comparison, chromosome-scale loss events were only captured in chrI of YJM978, a clinical strain. The laboratory strain SK1 was another strain with a large number of gene losses (>800), and this strain was also the strain most resistant to acetic acid (Fig. 2a).

Our *de novo* assembly and gene prediction suggested 7–25 potential novel non-S288c genes per strain, and most of these genes were nonmetabolic genes and homologous genes from other *Saccharomyces* strains (Dataset S3). Five of the 8 strains with >20 new genes were environmental strains. The strains CLIB382, UWOPS05-227.2, and UWOPS05-217.3 had the largest counts of new genes, whereas CLIB382 was also the strain most resistant to fumaric acid and 4-aminobenzoic acid (Fig. 2a). Ten new genes were shared by UWOPS05-227.2 and UWOPS05-217.3 (both related to the nectar of the Bertram palm), which also shared the high-resistance traits towards ferulic acid and 1,4-butanediol (Fig. 2a). Because these genes were absent in the reference strain S288c and largely uncharacterized, they have not been previously reported to be associated with the aforementioned phenotypes.

In the systematic comparisons between the genetic makeups of industrial and environmental strains (Brazilian bioethanol strains binned as 1 candidate; Supplemental Note S1), significantly more heterozygous SNPs/indels (Wilcoxon rank sum test, *P* = 4.1e–2), especially in bioethanol strains, and fewer indels (*P* = 3.6e–2) were observed in industrial strains (Fig. 4b–d). When investigating the genes influenced by these variants (the ratio of SNPs/indels falling in specific gene categories, or the number of genes affected by CNVs), we found relatively more SNPs/indels in the TF superfamily helix-turn-helix (HTH) in all subcategories of industrial strains (*P* = 1.7e–2; Fig. 4c–d). We also found significantly more duplicated TFs in industrial strains (bioethanol and brewing strains in particular), together with 3 TF superfamilies, zipper, HTH, and other (TFs belonging to neither the zipper, HTH, nor zinc finger superfamilies) (*P* < 0.05; Fig. 4c–d). More gene deletions were observed in plant strains (Kruskal-Wallis test, *P* < 0.05; Fig. 4d), and more interestingly, deletions of TFs were only observed in plant species.

### Phenotypic prediction using reconstructed strain-specific GSMMs

To analyze metabolic differences between the strains and predict metabolic phenotypes, strain-specific GSMMs were reconstructed by incorporating severe mutations, gene deletions, and non-S288c genes (Dataset S4, Supplemental Note S4). The pairwise comparison of the strain-specific models shows that the networks differ by at most only 2% of total reactions (Supplemental Note S4). The models were able to predict strain differences in the utilization of 30 carbon sources and 5 nitrogen sources (Supplemental Note S4) in comparison with the experimental data for selected strains (Dataset S5). Due to a recent study [38], interstrain differences could be observed in key metabolic fluxes simulated by strain-specific models, which could provide more meaningful biological insights than the reaction presence/absence comparisons. Thus, different activated fluxes were simulated by the strain-specific models (Supplemental Note S4). Higher fluxes of pyruvate mitochondrial transport via proton symport and pyruvate dehydrogenase were observed in environmental strains (Wilcoxon rank sum test, FDR = 6.6e–2; Fig. 3c), which are 2 key upstream reactions before the TCA cycle, suggesting that environmental strains have relatively higher energy flux from the pyruvate metabolism to the TCA cycle than industrial strains. When categorizing the energy fluxes by different subsystems, we found that environmental strains also had higher fluxes in fatty acid biosynthesis, the TCA cycle, and pyruvate metabolism, while industrial strains had higher fluxes towards fatty acid metabolism (degradation) (FDR < 0.1; Fig. 3c).

### Identification of the genetic features and patterns associated with different stress resistance

**Figure 5:**
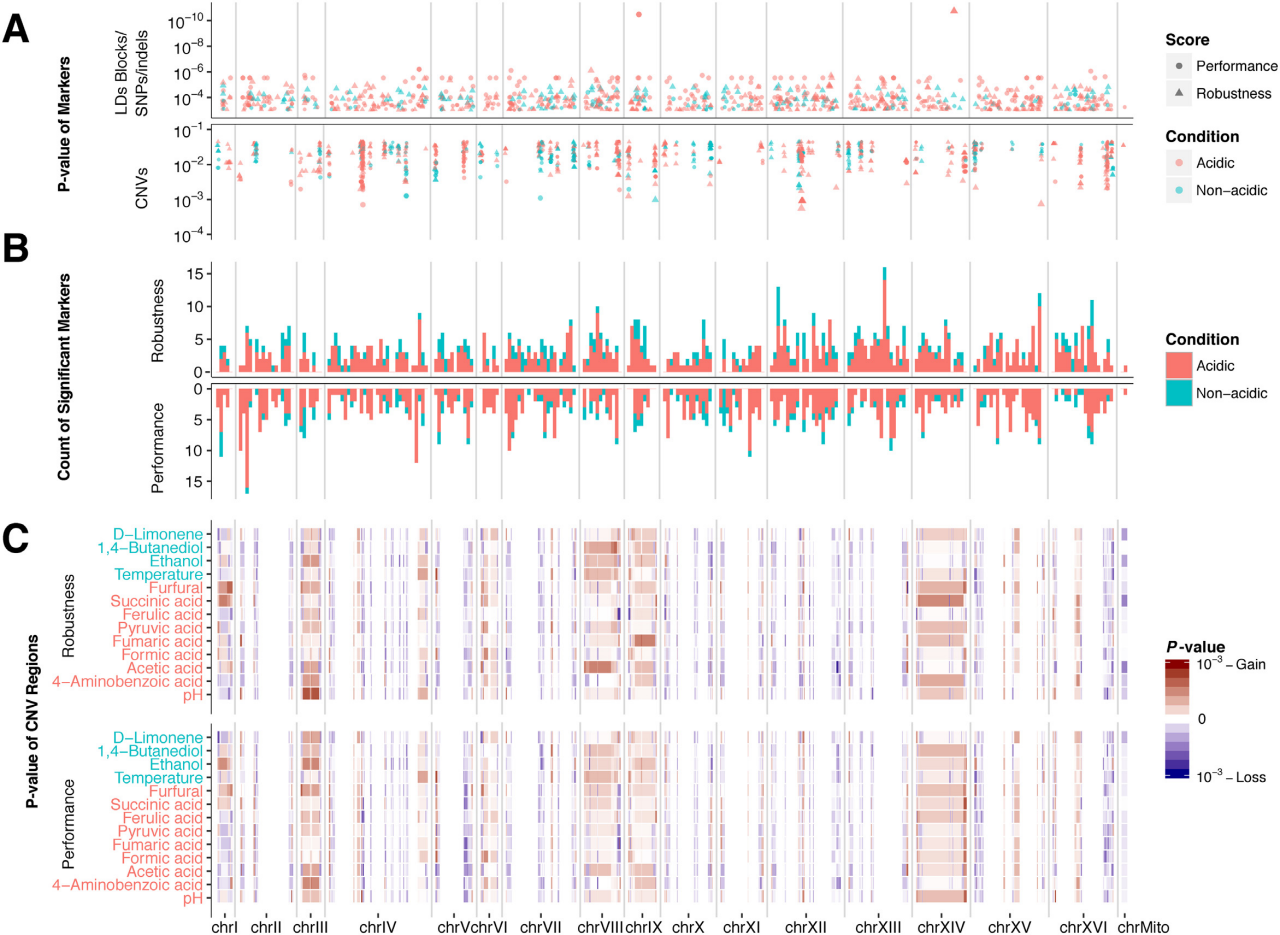
The GWASs for the resistance rankings and the genomic hot spots. (**A**) Genome-wide distributions of significant variants, for LDs or non-LD SNPs/indels and CNVs, respectively. (**B**) The total number of significant LDs and non-LD SNPs/indels in each 50kbp genomic window. (**C**) Genome-wide significance levels for CNV regions. Condition labels were colored by acidic or nonacidic condition classification. A region was colored red if its gain event was more significant than the loss event, or blue if vice versa.

To identify the potential genetic contributors to stress resistance phenotypes, SNP-based and CNV-based GWASs were performed (Fig. 5). For the SNP-based GWAS, to reduce the impact from the false-positive SNPs that were identified from the highly divergent genomic regions, the core genome regions shared by all strains were annotated by *de novo* assembled contiguous sequences, and only the SNPs/indels in the core genome were used as genotype markers (Fig. S4b and Supplemental Note S5). In summary, 3,449 LD blocks were identified, 165,358 SNP/indel markers were used in the SNP-based core genome GWAS (core-GWAS), and 880 CNV markers were used in the CNV-based GWAS. Significance cutoffs were obtained from the quantile-quantile plot of the *P* value distribution (Supplemental Note S5). In the SNP-based GWAS, significant markers for the robustness and performance rankings under nonacidic conditions were evenly distributed along the genome (Fig. 5b), while the 2 rankings under acidic conditions showed different genomic hot spots: e.g., chrII for performance and chrXIII for robustness. In the CNV-based GWAS, the robustness against acidic conditions always presented condition-specific genomic hot spots (especially for gain events): e.g., chrI, furfural; chrXIV, succinic acid; chrIX, fumaric acid; chrVIII, acetic acid; and chrIII, pH, all in gain events (Fig. 5c). In contrast, for performance, hot regions tended to be shared by multiple conditions, e.g., the loss events in chrV and chrVIII and the gain event in chrXIV (Fig. 5c and S4b). In general, the GWAS profiles for the performance under acidic and nonacidic conditions were relatively similar; on the contrary, the profiles for the robustness against acidic and nonacidic conditions were highly disparate (Fig. S4b).

The core-GWAS was successful in avoiding genomic regions with high variabilities (e.g., CNV regions, centromere, chromosome ends, break points of structural variations, mitochondrial DNA); thus, the genes processed by the SNP-based and CNV-based GWAS were not highly overlapping: from the 1,818 and 2,931 genes treated in the SNP-based and CNV-based GWAS, respectively, only 794 genes were in the shared regions, while no gene was deemed significant from both SNP-based and CNV-based GWAS within the 26 individual rankings (Fig. S4b, Table S2, and Dataset S6).

**Figure 6:**
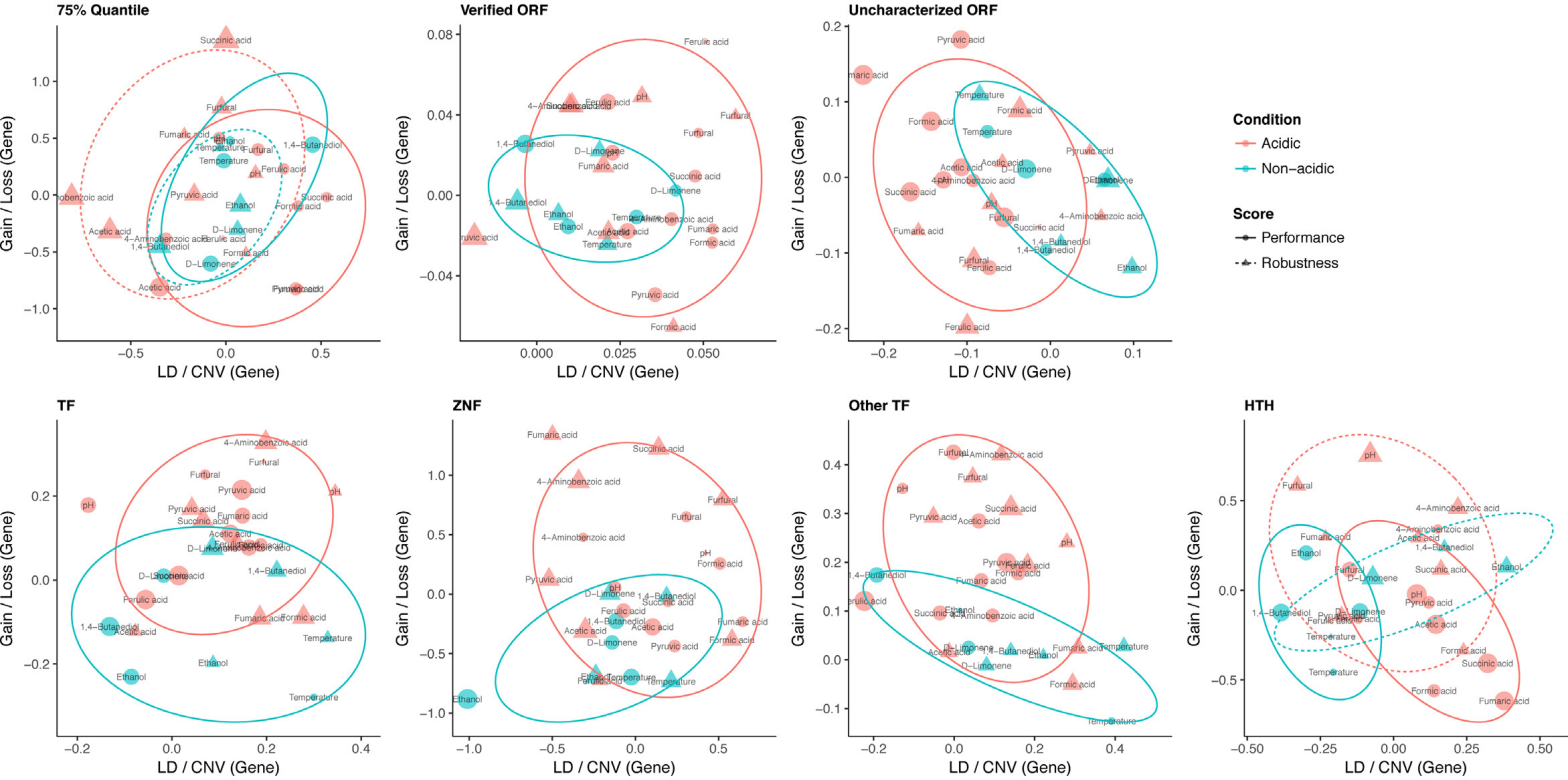
The relative contributions from different variant source (SNP or CNV, gain or loss event) to different inhibitory conditions and scores. The coordinates were calculated following the protocol in Supplemental Note S5. The first panel shows the relative contributions of the 75% quantile of all genes (sorted by *P* values). The other panels show the relative contributions in specific gene categories (verified or uncharacterized ORFs, TFs, and different TF superfamilies). ZNF: zinc finger superfamily.

The patterns of the 4 GWAS profile groups (acidic/nonacidic × robustness/performance) were more obvious when studying the relative contribution from SNP/gain/loss events in specific gene categories (Fig. 6). When looking at all genes sorted by GWAS *P* values and represented by the 75% quantile, the contribution patterns to the robustness and performance under nonacidic conditions were similar, while the robustness against acidic conditions had relatively higher contribution from CNVs, especially gain events. In verified ORFs, the resistance to acidic conditions was slightly skewed to SNPs, while in uncharacterized genes, CNVs presented a much higher contribution. Regarding TFs, in the TF superfamilies zinc finger and other, the resistances to acidic conditions were to a great extent contributed by gain events, which could be linked with the observation that the industrial strains had more TFs duplicated and were generally more resistant to acidic conditions. In the TF family HTH, the performance under acidic conditions was skewed to SNPs, compared with the performance under nonacidic conditions. Meanwhile, a higher SNP/indel rate in the HTH family was discovered in industrial strains compared with environmental strains.

**Figure 7:**
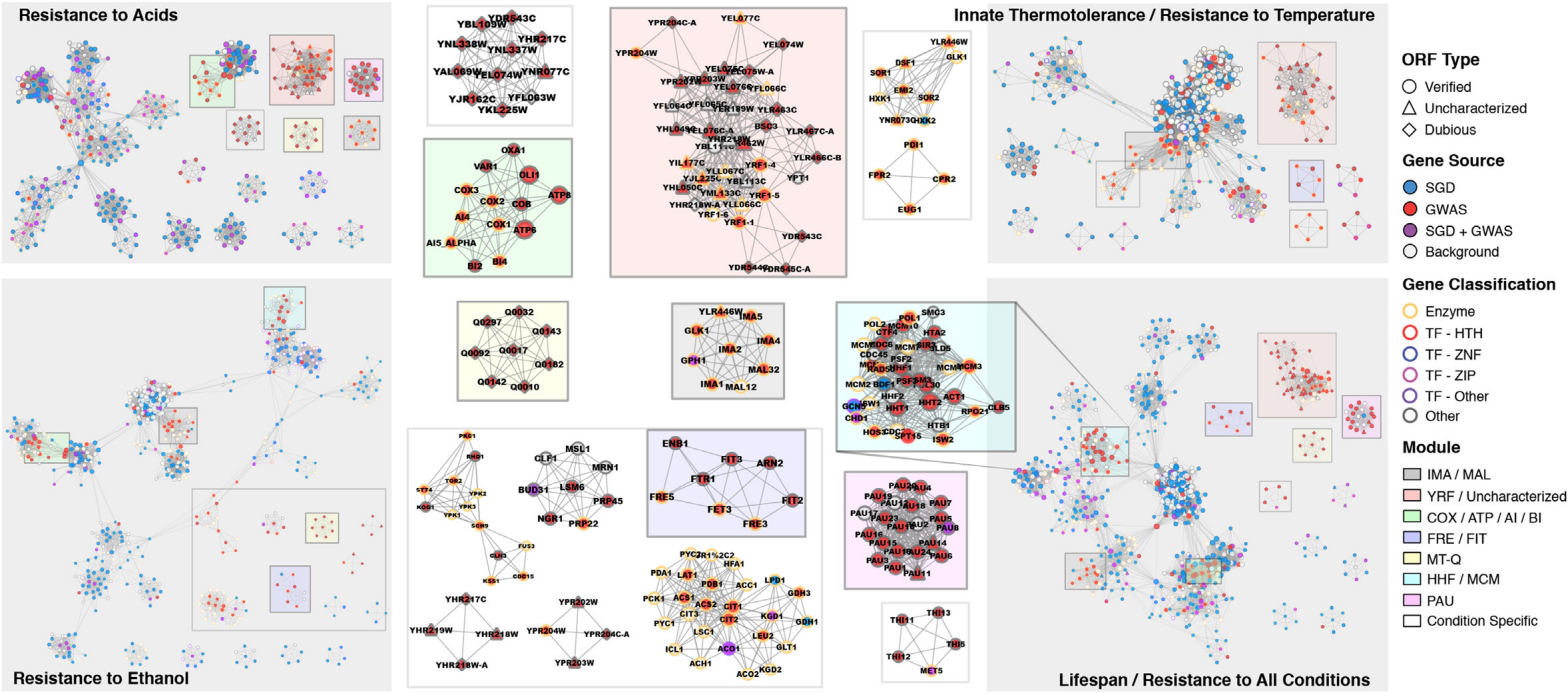
The PPINs of multiple phenotypes, with SGD recorded entries and GWAS observations. The node color indicates whether a gene is from the SGD entry, GWAS profile, or shared by both. The node border color and shape denote different gene categories. The 8 GWAS-specific modules (not identified from the network built from the SGD gene list) that were shared by multiple PPINs are highlighted in different background colors. Network-specific GWAS modules are shown against a white background.

To validate the GWAS results with literature-based knowledge, we compared the GWAS outcomes with the phenotype-associated gene lists in the Saccharomyces Genome Database (SGD) [11] (Supplemental Note S5). The significant gene lists from GWAS are highly noisy because several genes always appear contiguous in significant genomic regions (LD blocks or CNV regions). Therefore, it is possible that most of the genes receiving the same significant *P* values are just “passengers.” To identify “driver” genetic variants in the GWAS gene lists, we mapped the genes to PPINs and identified the core modules in the network by ModuleDiscoverer [28]. In the presented PPINs, the GWAS gene lists associated with resistances to acids, ethanol, thermotolerance, and lifespan (an SGD phenotype associated with the measurement of all our resistance scores) were found to be highly overlapping with the genes already recorded in SGD entries, suggesting a successful literature-based validation (Fig. 7). When making comparisons between the GWAS modules and the modules constructed by randomly selected gene lists with the same lengths (5 random gene lists for each GWAS gene list), GWAS modules constantly showed significantly higher modularity (more nodes in modules, more nodes in cliques, more internal edges, higher proportion of foreground nodes, Wilcoxon signed-rank test, *P* < 1e–2) and higher associations with SGD entries (more edges to SGD nodes, higher overlapping rate, higher overlapping rate of foreground nodes, *P* < 1e–2). Furthermore, additional GWAS-specific modules (SGD genes not highly observed in such modules) were also discovered, which represent valuable candidates for engineering target selection. Some of these modules were shared by multiple networks: the *IMA*/*MAL* module was shared by all 4 networks; the large *YRF*/Uncharacterized gene module was shared by lifespan, thermotolerance, and resistance to acids; the *COX*/*ATP*/Mitochondrial gene module was shared by resistances to acids and ethanol; and the *FRE*/*FIT* module was shared by thermotolerance and lifespan.

## Discussion

The mechanisms influencing stress resistance in yeast strains are complex, and the genetic interpretation of these findings will require more systematic investigations. Our comprehensive omics study, incorporating the discoveries from phenome, genome, metabolome, fluxome, GWAS, and the interactome, has been successful in establishing multidimensional associations among strain metadata, genotype, and resistance to different conditions.

Among the studies with large-scale phenotype screening, previous works focused on the metabolome [33, 39] and basic physiology [33, 40], while in this work we also developed a systematic scoring and ranking protocol to characterize the strains’ industrial potential regarding resistance to multiple industrially relevant stresses with the possibility of setting different weights to parameters according to different engineering purposes. Compared with previous association studies that aimed to establish genotype-to-phenotype links by using only SNPs [33, 41] or performed SNP-based GWAS in both the core and pan-genome region [37], here we introduced SNP-based core genome GWAS and CNV-based GWAS to reduce the rate of false-positive observations introduced by the pan-genome diversity of the yeast population (common GWAS procedure), and we took both SNPs/indels and structural variations into consideration. The reconstruction of strain-specific GSMMs and the prediction of energy fluxes provide a valuable resource for engineers working with different strains for different purposes.

To summarize our evidence in the phenotypic and genotypic screening and make some basic comparisons between industrial and environmental strains and among their subcategories, we have established several interesting associations. Regarding genotype, industrial strains tend to have higher heterozygosity (especially bioethanol strains), which could be a result of genome fusion during fermentation processes, lower indel rate, higher SNP/indel rate in the TF family HTH, higher frequencies of large-scale and high-intensity duplication events (especially affecting TFs), and lower possibility to acquire new genes. At the phenotypic level, industrial strains have relatively low levels of extracellular aromatic compounds and carboxylic acid and lower pathway activity in the TCA cycle but relatively high in ethanol metabolism, which is conducive to their functionality in anaerobic fermentation. Consequently, the accumulation of aliphatic acids from the TCA cycle, such as succinate and fumarate, may therefore contribute to the strains’ high innate resistance (especially robustness) to acidic conditions (bioethanol and food strains in particular). These resistances were found to be highly associated with duplication events, especially in uncharacterized genes and TFs, as well as the SNPs/indels in the HTH TF family. On the other hand, regarding the genotype, environmental strains tended to have lower heterozygosity but a higher indel rate, wider range of phylogenetic diversity, and higher frequencies of small-scale and random loss events, low possibility for large-scale duplications, and a higher tendency to acquire new genes—especially plant strains, in which deletions of TFs were also only observed in this subcategory. To summarize the phenotype observations, environmental strains, especially the plant species, tended to have relatively high extracellular metabolite levels, higher energy flux towards the TCA cycle, and high activity in fatty acid biosynthesis, which also improves the resistances in a wide range. They were in general not outstanding in robustness to extreme conditions (especially the acidic ones) but tended to have wider ranges of resistance (especially in the performance parameter) to multiple conditions, in particular the nonacidic conditions. These phenotypes have relatively high associations with gene loss events.

Regarding the selection of platform strains, our study revealed that the most commonly used *S. cerevisiae* strains, S288c and CEN.PK113-7D, performed rather poorly among all screened strains with regard to bioprocessing-relevant stress resistance. A tremendous amount of engineering work has been performed using these 2 strains as host strains [4, 5], but few evaluations have been performed to assess whether they are the best possible choices to use in industrial settings. Our findings suggest that many environmental strains also have potential industrial applications due to their unique stress resistance patterns when compared with industrial strains. More impressively, none of the top 3 strains with resistance to multiple stress conditions, Y55, RM11, and PW5, was isolated from current industrial yeast fermentations but rather all were initially isolated from plants and thus could serve as promising platform strains for bioprocessing purposes. The recent developments in CRISPR methods have also made it possible to genetically engineer almost any yeast strain [10]; thus, nonstandard or polypoid strains could be introduced in metabolic engineering projects [42].

From the point of view of genetics and evolution, evidence from previous studies matches our observations and assumptions quite well [43, 44]; from the engineering point of view, it has been reported that the duplication or overexpression of several TFs [45] and the mutagenesis of Spt15 [8], a TF from the HTH family, could boost stress resistance during fermentation. Remarkably, Spt15 is also the first priority suggested by our GWAS as a potential engineering target (Dataset S6); thus, we believe that the potential engineering targets and engineering strategies suggested by our study are of high confidence. Besides the previously revealed mechanisms and strategies (overexpression of TFs and transporters, mutagenesis of TFs, chromosomal duplications in industrial strains), there are several new outcomes arising from the present study, namely, the potential genotype resources and engineering targets associated with different stress conditions such as SNPs/indels and loss events of environmental strains, the TFs and significant genes that have not been previously engineered, uncharacterized gene clusters, and tandem repeats. Therefore, we propose several sources to select potential engineering targets, as follows:

1. *The extreme genotypes found in the well-performing strains*.
  For instance, the suggested platform strain Y55 has below average resistance to 4-aminobenzoic acid and ferulic acid. To improve the resistance to these and related compounds, genomic features from AL1, CLIB215, and GDB325, the strains most resistant to the selected conditions, could be introduced. Large-scale duplications of chrXII (∼3–4 copies from CLIB215), chrVI (6 copies from CLIB215), and the repeat region of chrVI: 742212–745751 (82 copies from AL1) could be engineered to modify Y55.
2. *The most significant variants and genes suggested by GWAS*.
  For example, for the resistance to ferulic acid, the LD blocks chrXIV: 605937–607882 (–log *P* = 11.76) and chrIX: 151566–161597 (–log *P* = 11.46) were identified as the most significant markers for robustness and performance, respectively. The SNPs/indels in these blocks and the overlapped genes could be considered as the first priority.
3. *The variants or genes revealed by multiple GWAS profiles to provide resistance to different conditions*.
  The CNV region YHR218W-YHR219 (with both gain and loss events from different rankings) was found to be significantly associated with resistance to 7 different stress conditions. The gain event of YNR059W-YNR062C was associated with resistance to 5 acidic conditions. The putative gene YER138W-A with unknown function was shared by strains resisstant to 6 acidic conditions, while its neighbor YER148W/*SPT15* (also significant in multiple conditions) has been previously reported to be highly associated with ethanol resistance [8]. *SKY1* was also a significant gene shared by variants resistant to 4 conditions; it has been shown to be associated with osmotic tolerance [46].
4. *Specific gene sets that were captured in multidimension studies*.
  Three TFs, Spt15 (HTH) (previously reported [8]), Ecm22 (zinc finger), and Bur6 (other TF), were identified by GWAS, among which Ecm22 and Bur6 were from the CNVs while Spt15 (HTH) was from the SNPs. The gain event of YNR059W-YNR062C was associated with robustness against multiple acidic conditions. These cases from GWAS also match the analyses in the genotyping.
5. *The modules discovered in the phenotype-associated PPIN*.
  The *COX*/*ATP*/mitochondrial gene module is part of the respiratory chain, and the genes were found to provide protection against acetic acid exposure and other stresses [47] owing to this module's association with respiration, redox, and ion balance. This could provide promising engineering targets for ethanol and acid resistance. The *FRE*/*FIT* module is associated with iron transport in the cell wall, whereas it has also been reported that tolerance of exposure to inorganic acid and weak acid is correlated with iron uptake [48, 49]. The *PAU* module (the seripauperin gene family), which encodes yeast cell wall mannoproteins [50], has been shown to be responsible for anaerobiosis and environmental stress tolerance [51]. Because the gain event of uncharacterized genes was discovered to be associated with acid resistance, the modules with uncharacterized and dubious genes (e.g., *YRF*/uncharacterized module, *YPR* and *YHR* modules for ethanol resistance, *SOR*/*HKX* module for thermotolerance) could also be used to characterize the new functions and the association with stress resistance. Other network-specific modules, especially those with multiple uncharacterized genes, could also be considered as engineering targets. For instance, the *PYC/LPD/KGD/CIT/ACS/GDH* module has genes from the Krebs cycle, the *GLK/SOR/HXK* module participates in phosphorylation, and the *SCH/YPK* module has genes from the fermentable growth medium signaling pathways. These genes from carbon metabolism were verified to be associated with resistance to alcohol and acid exposure [47].

## Potential Implications

The present work has not only revealed successfully the phenotypic and genotypic divergence of a representative strain collection and discussed the underlying evolutionary mechanisms but also proposed a practical toolbox for platform strain selection and identification of new engineering targets, a number of which we present here. Technically, the innovative methods used in the present study, including the comprehensive resistance score calculation, the strain-specific GSMM and fluxome construction, the analytically rigorous core-genome and CNV-based GWAS, and the noise-reducing PPIN module discovery, are also applicable to other geno- and phenotyping projects, especially strain-level population studies with high interstrain genetic diversity.

## Availability of supporting data

The whole-genome sequence data have been deposited in the NCBI Sequence Read Archive (http://www.ncbi.nlm.nih.gov/sra) under accession numbers SRR6114116 to SRR6114151 under project ID PRJNA412468.

The metabolomic data have been deposited in the European Bioinformatics Institute's MetaboLights repository with identifier MTBLS780.

Data further supporting this work are available in the GigaDB repository [52].

## Additional files

This article has 13 supplemental items, including the supplemental notes (including 5 sections), 2 tables, 4 figures, and 6 data sets in individual documents.

**Dataset S1**. The physiological characterization results.

**Dataset S2**. The intra- and extracellular metabolomes.

**Dataset S3**. The genetic makeups of the strains, including CNV profiles, SNP/indel profiles and non-S288c genes predicted by YGAP.

**Dataset S4**. The strain-specific genome-scale metabolic models (in .xml files).

**Dataset S5**. The growth data for CEN.PK, ethanol red, and S288c in the utilization of different carbon and nitrogen sources.

**Dataset S6**. The genes significantly associated with robustness and performance rankings discovered by GWAS.

**Dataset S7**. The growth curve raw data for physiological characterization.

**Table S1**. The variant statistics.

**Table S2**. The GWAS profile statistics on significant genes.

**Figure S1**. The performance and robustness ranking values in all conditions for all strains.

**Figure S2**. The parameter sensitivity analysis of the strain Y55. (**A**) Sensitivity of the ranking position of strain Y55 regarding each growth parameter. Positive and negative parameter influence (PI) scores indicate an increase or decrease in ranking position, respectively, when the corresponding parameter is given more weight in the rank variability analysis (Supplemental Note S2). PI scores are normalized to be in the range –1 to 1, representing the extreme scores among all parameters and conditions. (**B**) Significant negative correlations (*P* ≤ 0.05) on PI scores between Y55 and other strains. Larger markers indicate higher probability to fall in the same ranking range.

**Figure S3**. The correlations among phenotypic rankings including the resistance scores and the metabolomic features. Spearman correlation coefficients are shown in a heat map: red for a positive correlation and blue for a negative correlation. Color bars distinguish the acidic or nonacidic inhibitory conditions class and the intra- or extracellular metabolome. Label colors represent the robustness or performance score for the resistance rankings, different pathway or compound class for intracellular metabolome, or production or consumption for extracellular metabolome.

**Figure S4**. The CNV region distribution for 36 strains (A) and the LD blocks and CNV regions used for GWAS (B). **(A)** Gain, loss, and deletion events are indicated by different colors. Each layer stands for an individual strain (excluding S288c). (**B**) The distributions of significant genes and their variant sources. The green layer illustrates the core genome regions used by the SNP-based GWAS (core-GWAS; Supplemental Note S5), while the red layer indicates the CNV regions. The inner layers represent the significant genes for all rankings, robustness against acidic conditions, performance under acidic conditions, robustness against nonacidic conditions, and performance under nonacidic conditions, respectively. Colors indicate variant source: pink symbols were captured in both gain and loss events, while brown symbols were significant in both SNP-based GWAS and CNV-based GWAS.

## Abbreviations

bp: base pairs; chr: chromosome; CNV: copy number variation; core-GWAS: SNP-based core genome GWAS; FDR: false-discovery rate; GC-MS: gas chromatography–mass spectrometry; GSMM: genome-scale metabolic models; GWAS: genome-wide association studies; HTH: helix-turn-helix; indel: insertion and deletion; LD: linkage disequilibrium; OD: optical density; ORF: open reading frame; PI: parameter influence; PPIN: protein-protein interaction network; RVA: rank variability analysis; SGD: *Saccharomyces* Genome Database; SNP: single-nucleotide polymorphism; TCA cycle: tricarboxylic acid cycle; TF: transcription factor; YGAP: yeast genome annotation pipeline; YPD: yeast extract peptone dextrose.

## Competing interests

The authors declare that they have no competing interests.

## Funding

G.P. would like to thank Deutsche Forschungsgemeinschaft (DFG) CRC/Transregio 124 “Pathogenic fungi and their human host: Networks of interaction,” subproject B5. B.B., L.D., M.J.H., and J.F. thank the Novo Nordisk Foundation for financial support.

## Author contributions

B.B. designed the physiological characterization. B.B. and L.D. performed the experiments. K.K. performed the data analyses. B.B., K.K., and D.M. wrote the initial manuscript. G.P., J.F., and M.J.H. supervised this study. D.M. performed the GSMM construction and simulation. H.M. and S.V.B. performed the metabolomic measurements. J.L. advised the bioinformatics analyses. All authors conceived the project and approved the final version of manuscript.

## Supplementary Material

GIGA-D-18-00382_Original_Submission.pdf

GIGA-D-18-00382_Revision_1.pdf

GIGA-D-18-00382_Revision_2.pdf

Response_to_Reviewer_Comments_Original_Submission.pdf

Response_to_Reviewer_Comments_Revision_1.pdf

Reviewer_1_Report_Original_Submission -- Jiazhang Lian 11/23/2018 Reviewed

Reviewer_1_Report_Revision_1 -- Jiazhang Lian 1/6/2019 Reviewed

Reviewer_2_Report_Original_Submission -- yasumune nakayama 12/9/2018 Reviewed

Reviewer_3_Report_Original_Submission -- Amy Lee 12/17/2018 Reviewed

Reviewer_3_Report_Revision_1 -- Amy Lee 1/9/2019 Reviewed

Supplemental Files

## References

[1] de Jong E, Higson A, Walsh P, et al. Bio-based chemicals: value added products from biorefineries. Report. IEA Bioenergy, Task42 Biorefinery; 2012.

[2] Taylor R, Nattrass L, Alberts G, et al. From the sugar platform to biofuels and biochemicals. Final Report for the European Commission Directorate-General Energy, No. ENER/C2/423-2012/SI2 673791; 2015.

[3] Archer CT, Kim JF, Jeong H, et al. The genome sequence of *E. coli* W (ATCC 9637): comparative genome analysis and an improved genome-scale reconstruction of *E. coli*. BMC Genomics. 2011;12:9. 21208457 10.1186/1471-2164-12-9 PMC3032704

[4] van Dijken JP, Bauer J, Brambilla L, et al. An interlaboratory comparison of physiological and genetic properties of four *Saccharomyces cerevisiae* strains. Enzyme Microb Technol. 2000;26(9–10):706–14. 10862876 10.1016/s0141-0229(00)00162-9

[5] Çakar ZP, Turanlı-Yıldız B, Alkım C, et al. Evolutionary engineering of *Saccharomyces cerevisiae* for improved industrially important properties. FEMS Yeast Res. 2012;12(2):171–82. 22136139 10.1111/j.1567-1364.2011.00775.x

[6] Li BZ, Yuan YJ Transcriptome shifts in response to furfural and acetic acid in *Saccharomyces cerevisiae*. Appl Microbiol Biotechnol. 2010;86(6):1915–24. 20309542 10.1007/s00253-010-2518-2

[7] Castle LA, Siehl DL, Gorton R et al. Discovery and directed evolution of a glyphosate tolerance gene. Science. 2004;304(5674):1151–4. 15155947 10.1126/science.1096770

[8] Alper H, Moxley J, Nevoigt E, et al. Engineering yeast transcription machinery for improved ethanol tolerance and production. Science. 2006;314(5805):1565–8. 17158319 10.1126/science.1131969

[9] Verduyn C, Postma E, Scheffers WA et al. Effect of benzoic acid on metabolic fluxes in yeasts: a continuous-culture study on the regulation of respiration and alcoholic fermentation. Yeast. 1992;8(7):501–17. 1523884 10.1002/yea.320080703

[10] Stovicek V, Borodina I, Forster J CRISPR–Cas system enables fast and simple genome editing of industrial *Saccharomyces cerevisiae* strains. Metabol Eng Commun. 2015;2:13–22. 10.1016/j.meteno.2015.03.001 PMC8193243 34150504

[11] Cherry JM, Hong EL, Amundsen C, et al. Saccharomyces Genome Database: the genomics resource of budding yeast. Nucleic Acids Res. 2012;40(Database issue):D700–5. 22110037 10.1093/nar/gkr1029 PMC3245034

[12] Li H, Durbin R Fast and accurate long-read alignment with Burrows-Wheeler transform. Bioinformatics. 2010;26(5):589–95. 20080505 10.1093/bioinformatics/btp698 PMC2828108

[13] DePristo MA, Banks E, Poplin R, et al. A framework for variation discovery and genotyping using next-generation DNA sequencing data. Nat Genet. 2011;43(5):491–8. 21478889 10.1038/ng.806 PMC3083463

[14] McKenna A, Hanna M, Banks E, et al. The Genome Analysis Toolkit: a MapReduce framework for analyzing next-generation DNA sequencing data. Genome Res. 2010;20(9):1297–303. 20644199 10.1101/gr.107524.110 PMC2928508

[15] Cingolani P, Platts A, Wang le L, et al. A program for annotating and predicting the effects of single nucleotide polymorphisms, SnpEff: SNPs in the genome of *Drosophila melanogaster* strain w1118; iso-2; iso-3. Fly. 2012;6(2):80–92. 22728672 10.4161/fly.19695 PMC3679285

[16] Boeva V, Popova T, Bleakley K, et al. Control-FREEC: a tool for assessing copy number and allelic content using next-generation sequencing data. Bioinformatics. 2012;28(3):423–5. 22155870 10.1093/bioinformatics/btr670 PMC3268243

[17] Vilella AJ, Severin J, Ureta-Vidal A, et al. EnsemblCompara GeneTrees: complete, duplication-aware phylogenetic trees in vertebrates. Genome Res. 2009;19(2):327–35. 19029536 10.1101/gr.073585.107 PMC2652215

[18] Proux-Wera E, Armisen D, Byrne KP et al. A pipeline for automated annotation of yeast genome sequences by a conserved-synteny approach. BMC Bioinformatics. 2012;13:237. 22984983 10.1186/1471-2105-13-237 PMC3507789

[19] Pruitt KD, Tatusova T, Maglott DR NCBI reference sequences (RefSeq): a curated non-redundant sequence database of genomes, transcripts and proteins. Nucleic Acids Res. 2007;35(Database issue):D61–5. 17130148 10.1093/nar/gkl842 PMC1716718

[20] Gish W, States DJ Identification of protein coding regions by database similarity search. Nat Genet. 1993;3(3):266–72. 8485583 10.1038/ng0393-266

[21] Mo ML, Palsson BO, Herrgard MJ Connecting extracellular metabolomic measurements to intracellular flux states in yeast. BMC Syst Biol. 2009;3:37. 19321003 10.1186/1752-0509-3-37 PMC2679711

[22] UniProt Consortium. The Universal Protein Resource (UniProt) in 2010. Nucleic Acids Res. 2010;38(Database issue):D142–8. 19843607 10.1093/nar/gkp846 PMC2808944

[23] Moretti S, Martin O, Van Du Tran T, et al. MetaNetX/MNXref–reconciliation of metabolites and biochemical reactions to bring together genome-scale metabolic networks. Nucleic Acids Res. 2016;44(D1):D523–6. 26527720 10.1093/nar/gkv1117 PMC4702813

[24] Kang HM, Zaitlen NA, Wade CM, et al. Efficient control of population structure in model organism association mapping. Genetics. 2008;178(3):1709–23. 18385116 10.1534/genetics.107.080101 PMC2278096

[25] Teixeira MC, Monteiro P, Jain P, et al. The YEASTRACT database: a tool for the analysis of transcription regulatory associations in *Saccharomyces cerevisiae*. Nucleic Acids Res. 2006;34(Database issue):D446–51. 16381908 10.1093/nar/gkj013 PMC1347376

[26] Caspi R, Billington R, Ferrer L, et al. The MetaCyc database of metabolic pathways and enzymes and the BioCyc collection of pathway/genome databases. Nucleic Acids Res. 2016;44(D1):D471–80. 26527732 10.1093/nar/gkv1164 PMC4702838

[27] Jensen LJ, Kuhn M, Stark M, et al. STRING 8–a global view on proteins and their functional interactions in 630 organisms. Nucleic Acids Res. 2009;37(Database issue):D412–6. 18940858 10.1093/nar/gkn760 PMC2686466

[28] Vlaic S, Conrad T, Tokarski-Schnelle C, et al. ModuleDiscoverer: identification of regulatory modules in protein-protein interaction networks. Sci Rep. 2018;8(1):433. 29323246 10.1038/s41598-017-18370-2 PMC5764996

[29] R Core Team. R: a language and environment for statistical computing. Vienna, Austria: R Foundation for Statistical Computing; 2015.

[30] Shannon P, Markiel A, Ozier O, et al. Cytoscape: a software environment for integrated models of biomolecular interaction networks. Genome Res. 2003;13(11):2498–504. 14597658 10.1101/gr.1239303 PMC403769

[31] Houghton-Larsen J, Brandt A Fermentation of high concentrations of maltose by *Saccharomyces cerevisiae* is limited by the COMPASS methylation complex. Appl Environ Microbiol. 2006;72(11):7176–82. 16980427 10.1128/AEM.01704-06 PMC1636176

[32] Upchurch RG Fatty acid unsaturation, mobilization, and regulation in the response of plants to stress. Biotechnol Lett. 2008;30(6):967–77. 18227974 10.1007/s10529-008-9639-z

[33] Skelly DA, Merrihew GE, Riffle M, et al. Integrative phenomics reveals insight into the structure of phenotypic diversity in budding yeast. Genome Res. 2013;23(9):1496–504. 23720455 10.1101/gr.155762.113 PMC3759725

[34] Strope PK, Skelly DA, Kozmin SG, et al. The 100-genomes strains, an S*. cerevisiae* resource that illuminates its natural phenotypic and genotypic variation and emergence as an opportunistic pathogen. Genome Res. 2015;25(5):762–74. 25840857 10.1101/gr.185538.114 PMC4417123

[35] Liti G, Carter DM, Moses AM, et al. Population genomics of domestic and wild yeasts. Nature. 2009;458(7236):337–41. 19212322 10.1038/nature07743 PMC2659681

[36] da Silva-Filho EA, Brito dos Santos SK, Resende Ado M, et al. Yeast population dynamics of industrial fuel-ethanol fermentation process assessed by PCR-fingerprinting. Antonie Van Leeuwenhoek. 2005;88(1):13–23. 15928973 10.1007/s10482-004-7283-8

[37] Peter J, De Chiara M, Friedrich A, et al. Genome evolution across 1,011 *Saccharomyces cerevisiae* isolates. Nature. 2018;556(7701):339–44. 29643504 10.1038/s41586-018-0030-5 PMC6784862

[38] Monk JM, Koza A, Campodonico MA, et al. Multi-omics quantification of species variation of *Escherichia coli* links molecular features with strain phenotypes. Cell Syst. 2016;3(3):238–51.e12. 27667363 10.1016/j.cels.2016.08.013 PMC5058344

[39] Breunig JS, Hackett SR, Rabinowitz JD, et al. Genetic basis of metabolome variation in yeast. PLoS Genet. 2014;10(3):e1004142. 24603560 10.1371/journal.pgen.1004142 PMC3945093

[40] Borneman AR, Desany BA, Riches D, et al. Whole-genome comparison reveals novel genetic elements that characterize the genome of industrial strains of *Saccharomyces cerevisiae*. PLoS Genet. 2011;7(2):e1001287. 21304888 10.1371/journal.pgen.1001287 PMC3033381

[41] Kang K, Li J, Lim BL, et al. MESSI: metabolic engineering target selection and best strain identification tool. Database (Oxford). 2015;2015; doi:10.1093/database/bav076. PMC4529744 26255308

[42] Zhang GC, Kong II, Kim H, et al. Construction of a quadruple auxotrophic mutant of an industrial polyploid *Saccharomyces cerevisiae* strain by using RNA-guided Cas9 nuclease. Appl Environ Microbiol. 2014;80(24):7694–701. 25281382 10.1128/AEM.02310-14 PMC4249234

[43] Bergstrom A, Simpson JT, Salinas F, et al. A high-definition view of functional genetic variation from natural yeast genomes. Mol Biol Evol. 2014;31(4):872–88. 24425782 10.1093/molbev/msu037 PMC3969562

[44] Caspeta L, Chen Y, Ghiaci P, et al. Biofuels. altered sterol composition renders yeast thermotolerant. Science. 2014;346(6205):75–8. 25278608 10.1126/science.1258137

[45] Alriksson B, Horváth IS, Jönsson LJ Overexpression of *Saccharomyces cerevisiae* transcription factor and multidrug resistance genes conveys enhanced resistance to lignocellulose-derived fermentation inhibitors. Process Biochem. 2010;45(2):264–71.

[46] Yoshikawa K, Tanaka T, Furusawa C, et al. Comprehensive phenotypic analysis for identification of genes affecting growth under ethanol stress in *Saccharomyces cerevisiae*. FEMS Yeast Res. 2009;9(1):32–44. 19054128 10.1111/j.1567-1364.2008.00456.x

[47] Henriques SF, Mira NP, Sá-Correia I Genome-wide search for candidate genes for yeast robustness improvement against formic acid reveals novel susceptibility (Trk1 and positive regulators) and resistance (Haa1-regulon) determinants. Biotechnol Biofuels. 2017;10:96. 28428821 10.1186/s13068-017-0781-5 PMC5395885

[48] Mira NP, Teixeira MC, Sá-Correia I Adaptive response and tolerance to weak acids in *Saccharomyces cerevisiae*: a genome-wide view. OMICS. 2010;14(5):525–40. 20955006 10.1089/omi.2010.0072 PMC3129613

[49] Abbott DA, Suir E, van Maris AJ, et al. Physiological and transcriptional responses to high concentrations of lactic acid in anaerobic chemostat cultures of *Saccharomyces cerevisiae*. Appl Environ Microbiol. 2008;74(18):5759–68. 18676708 10.1128/AEM.01030-08 PMC2547041

[50] Marguet D, Guo XJ, Lauquin GJ Yeast gene SRP1 (serine-rich protein). Intragenic repeat structure and identification of a family of SRP1-related DNA sequences. J Mol Biol. 1988;202(3):455–70. 3139887 10.1016/0022-2836(88)90278-1

[51] Rivero D, Berna L, Stefanini I, et al. Hsp12p and PAU genes are involved in ecological interactions between natural yeast strains. Environ Microbiol. 2015;17(8):3069–81. 26079802 10.1111/1462-2920.12950

[52] Kang K, Bergdahl B, Machado D, et al. Supporting data for “Linking genetic, metabolic, and phenotypic diversity among *Saccharomyces cerevisiae* strains using multi-omics associations”. GigaScience Database. 2019 10.5524/100558. PMC6446221 30715293

