# Supplementary material for: Linking genetic, metabolic, and phenotypic diversity among Saccharomyces cerevisiae strains using multi-omics associations
Source: Gigascience. 2019 Jan 31;8(4):giz015. doi: 10.1093/gigascience/giz015 (PMC6446221; doi:10.1093/gigascience/giz015)
Supplement: GIGA-D-18-00382_Revision_1.pdf [file giz015_giga-d-18-00382_revision_1.pdf]

GigaScience

# Linking genetic, metabolic and phenotypic diversity among S. cerevisiae strains using multi-omics associations

--Manuscript Draft--

| | |
|---|---|
| Manuscript Number: | GIGA-D-18-00382R1 |
| Full Title: | Linking genetic, metabolic and phenotypic diversity among S. cerevisiae strains using multi-omics associations |
| Article Type: | Research |
| Funding Information: | Novo Nordisk Fonden — Dr. Jochen Förster<br>Deutsche Forschungsgemeinschaft (DE) (CRC/Transregio 124) — Dr. Gianni Panagiotou |
| Abstract: | The selection of bioengineering platform strains and engineering strategies to improve the stress resistance of Saccharomyces cerevisiae remains a pressing need in bio-based chemical production. Thus, a systematic effort to exploit the genotypic and phenotypic diversity to boost yeast's industrial value is still urgently needed. Here, we analyzed 5400 growth curves obtained from 36 S. cerevisiae strains and comprehensively profiled their resistances against 13 industrially relevant stresses. We observed that bioethanol and brewing strains exhibit higher resistance against acidic conditions, however, plant isolates tend to have wider range of resistance, which may be associated with their metabolome and fluxome signatures in TCA cycle and fatty acid metabolism. By deep genomic sequencing we found that industrial strains have more genomic duplications especially affecting transcription factors, presenting disparate evolutionary paths in comparison to the environmental strains which have more InDels, gene deletions and strain-specific genes. Genome-wide association studies coupled with protein-protein interaction networks uncovered novel genetic determinants of stress resistances. These resistance-related engineering targets and strain rankings provide a valuable source for engineering significantly improved industrial platform strains. |
| Corresponding Author: | Gianni Panagiotou, Ph.D.<br>Leibniz Institute for Natural Product and Infection Biology - Hans Knoell Institute (HKI)<br>Jena, Thuringia GERMANY |
| Corresponding Author Secondary Information: | |
| Corresponding Author's Institution: | Leibniz Institute for Natural Product and Infection Biology - Hans Knoell Institute (HKI) |
| Corresponding Author's Secondary Institution: | |
| First Author: | Kang Kang |
| First Author Secondary Information: | |
| Order of Authors: | Kang Kang<br>Basti Bergdahl<br>Daniel Machado<br>Laura Dato<br>Ting-Li Han<br>Jun Li<br>Silas Villas-Boas<br>Markus J. Herrgård<br>Jochen Förster<br>Gianni Panagiotou, Ph.D. |

Powered by Editorial Manager® and ProduXion Manager® from Aries Systems Corporation

| Order of Authors Secondary Information: | |
|---|---|
| Response to Reviewers: | ear Dr. Nicole Nogoy,<br><br>Thank you very much for the opportunity to revise our manuscript, “Linking genetic, metabolic and phenotypic diversity among S. cerevisiae strains using multi-omics associations” for publication in GigaScience. We are pleased to submit the revised version, in which we have addressed all suggestions of the three reviewers, and a point-to-point response to their comments could be found below. We have also adjusted some of our author’s name and order, please be noticed.<br><br>We are looking forward to hearing from you and we are hoping a positive evaluation.<br><br>Sincerely,<br><br>Gianni Panagiotou, PhD<br><br><br>Respond to Editor’s note:<br>Overall, a better description of the data analysis of metabolome and fluxome is required and reviewer #2 highlighted that some important procedures are missing for others to replicate the experiment. We strongly suggest you add your methods into Protocols.io - and the Protocols.io DOI cited in them manuscript. Note that our journal has a strong focus on reproducible research and we require such information is added for reviewers to adequately replicate your work. Furthermore, the quality of your results is not sufficiently discussed and needs to be improved to convince the reviewers.<br>We have made all suggested revisions to better describe the metabolome and fluxome analysis and prepared point-to-point responses to all reviewers’ questions. Required raw data (Dataset S7) and scripts are all available now. However, the submission systems automatically unzip all zip files. We alternatively uploaded Datasets S4 & S7 into the ftp folder.<br>Please also ensure that the genomic data associated with the SRA accession number is accessible, and add the MetaboLights accession number in the paper under "Availability of Supporting Data".<br>We have prepared the standard MetaboLights submission, with accession number: MTBLS780. The public date was set to 28/12/2018, which is the most recent date we can choose, as the administrators may manually curate the data. And our SRA submission is open to public now (SRR6114130 to SRR6114127).<br>In addition, please register any new software application in the SciCrunch.org database to receive a RRID (Research Resource Identification Initiative ID) number, and include this in your manuscript. This will facilitate tracking, reproducibility and re-use of your tool.<br>We have uploaded two self-developed pipelines to Github and SciCrunch: the metabolomic data processing pipeline, Metab 1.0 (https://github.com/reinkk/Metab, SCR_016877) and the growth curve processing pipeline, Growth-Profiling-Toolbox 1.0 (https://github.com/reinkk/Growth-Profiling-Toolbox, SCR_016878)<br><br>Answers to reviewers:<br><br>Reviewer #1:<br>In this manuscript, the authors did large-scale comparative genomics studies on various yeast species. Based on the growth phenotypes in the presence of different inhibitors or harsh industrial conditions, the authors tried to find the genetic determinants of several industrially desirable phenotypes. Although the topic covered in this manuscript is important for industrial biotechnology, unfortunately, the reviewer is not convinced that the authors have achieved what they claimed. Following please find a few suggestions to improve this manuscript.<br>We would like to thank this reviewer for his/her comments.<br>1. The stress response is precisely controlled by the regulatory networks, which means that transcriptomics may be more reliable than genomics studies for the purpose of this study.<br>Thank you for your suggestion. Transcriptomics would be definitely expand the value of such a multi-omic study. The current study has focused more on the identification of industrial phenotype associated genotypes, thus ,we believe ultra-depth sequencing, in particular covering the genomic variants affecting transcription factor and non-coding |

Powered by Editorial Manager® and ProduXion Manager® from Aries Systems Corporation

regions such as promoters, are in higher priority. To fill the gap in the RNA-level, we performed sufficient work in GWAS followed by protein-protein interaction networks, which has successfully associated the industrial phenotypes with regulatory networks.
2. The most important data that the reviewer expect to have is the list of genes associated with the desirable phenotypes. Unfortunately, it is not provided in this manuscript.
Due to the length limitation, the regarding gene lists were summarized in Dataset S6 but not listed in the main text. We have inserted more clear quotations in the main text to guide the reader.
3. All bioinformatics derived data should be verified. Otherwise, such data can be wrong or misleading and may be useless for practical biotechnology applications.
Thank you for your comment. The pipelines we used for the bioinformatic analyses are the state-of-the-art used for population genomics (e.g., BWA for mapping, GATK for SNP/InDel calling, FreeC for CNV calling, EMMA for GWAS) or specifically developed for S. cerevisiae (YGAP gene annotation pipeline). The tools and pipelines are also in line with the ones applied in recent publications in the most prestigious journals of the field of yeast population genomics (Liti et al., 2009a; Peter et al., 2018). Thus we are confident that our results are technically reliable and reproducible. Furthermore, our study is a systems level analysis linking genotypes with phenotypes therefore we focused on confirming holistically the accuracy of our findings using extensively public available phenotypic databases (such as SGD) and other bioinformatics approaches (PPI networks). Verifying one association experimentally will not in our opinion add to our study, which serves as a very important source to experimental groups for future metabolic engineering endeavours.

Reviewer #2:
The manuscript titled "Linking genetic, metabolic and phenotypic diversity among S.cerevisiae strains using multi-omics association" details the characteristics of various budding yeast strains with multi omics. Authors analyzed phenotypes, metabolome, fluxome and genome of the strains. They demonstrated the possible strategy of strain selection and improvement.
This study includes several useful information, such as widely analyzed stress tolerance and genome-wide association study. However, manuscript of several part should be improved. Especially the description of data analysis of metabolome and fluxome is missing some important procedures for others to replicate the experiment. And quality of result is not sufficiently discussed.
We would like to thank this reviewer for his/her positive comments.
Major point is below.
Line 86-88.
Maximum OD is also important parameter for industrial usage. Please, consider including the parameter in your study. At least, include the maximum OD of each strain under the general condition in this study.
Thank you for this valuable comment. The total number of generations was chosen as the measure instead of the final OD as it also takes into account the starting OD which could vary among different experiments, in which the cells were under extreme stress. When coming to industrial production, considering that the initial OD would be stable, then the parameter “number of generations” could be definitely replaced by final OD. Some selected cases were also compared with the conventional procedure which showed that the two methods actually correspond quite well. Actually, we now shared our pipeline in Matlab on Github (https://github.com/reinkk/Growth-Profiling-Toolbox) and SciCrunch (SCR_016878), which allows user to modify some simple codes to use max OD as a parameter to replace “number of generations.
Line 142-151.
The part of Parameter Influence Analysis makes me confusing. I could not understand why it is discussed here. What the advantage of this analysis. For example, we all realize that lag phase and average growth rate are less important for continuous fermentation without the analysis.
Since we know before the analysis that lag phase and average growth rate are less important for continuous fermentation, and that is the intended use of the strain, we do not want to select a strain which ranks high due to these parameters. Therefore, PIA is introduced as a tool to understand in depth which are the parameters driving the phenotypic differences. The main advantage of PIA is that it can distinguish strains with similar rank (when giving equal weight to all five growth parameters) from each other and thereby assist in selecting the strain most suited for a specific application, and

therefore allows researchers to adjust parameters' weights for different industrial purposes. When selecting a platform strain for a continuous fermentation process, low weight could be given to parameters like "lag phase". In contrast in a batch fermentation, "lag phase" becomes a determinant parameter which can influence the fermentation cycle dramatically; in that case a higher weight could be given to "lag phase". To make this clearer, we have modified related descriptions in the main text as: "Such information can be useful when selecting a host strain for specific process by setting different weights to different growth parameters, e.g. lag phase would be less important selection criteria for a continuous fermentation process and subsequently be given a low weight, however, in a batch fermentation process, lag phase duration becomes critical and could be assigned with a higher weight".
Line 154-156.
Show the score plot of principal component analysis of metabolome data as supplemental figure to show the data quality of biological and technical replicates. "intracellular" in Dataset S2 can be applied to directly to PCA. Unit variance is probably enough for preprocessing. Also, show the PCA of "extracellular" data.
Thank you for this excellent suggestion. We have applied such analyses and place the new figures in Supplementary Note S3 as Figures SN 6 & 7, under a new section named "Metabolite profile similarity by Principal Component Analysis (PCA) analysis".
Line 240-254.
Please discuss the reliability of the fluxes calculated in this study.
We cited a recent paper used similar simulation technology showing that, strain-specific models can simulate differences in fluxes and give more biological insights than comparing metabolic reactions. So we added "Due to a recent study (Monk et al., 2016), inter-strain differences could be observed in key metabolic fluxes simulated by strain-specific models, which could provide more meaningful biological insights than the reaction presence/absence comparisons.".
Line 251-254.
Comparison of flux of limiting steps is probably more suitable to compare the flux of pathways instead using the summed fluxes.
We fully agree. So we also presented the fluxes of key limited steps (e.g., Pyruvate Mitochondrial Transport) before the summed fluxes. However, the pathways we listed here, e.g., Citric Acid Cycle and Pyruvate Metabolism, have non-linear complex network structures which are consisted of multiple parallel reactions. Thus we think the overall flux description may give a different view from the key limiting step.
Line 257-316.
Are the sample number and sequence depth enough to perform SNP-based and CNV based GWAS?
The genomes were sequenced at a minimal depth of 52X, which is sufficient for SNP and CNV calling and GWAS analysis. Previous studies have also conducted GWAS on a strain cluster with similar number (35 ~ 38) of yeast strains (Connelly et al., 2013; Liti et al., 2009a; Warringer et al., 2011). Also, there is systematic evaluation of GWAS on S. cerevisiae strains of such sample size (Diao and Chen, 2012), suggesting that biologically meaningful results could be observed. Also, our PPIN analysis assisted dramatically in the reduction of false-positive noises in the GWAS analysis.
Figure 1.
In this study, growth phenotypes are mostly discussed as relative values. However absolute values are also important. Especially the variance of the phenotypic scores under each stress. Please show them as supplemental figures. Box plots may be suitable.
Thank you for this suggestion. All raw parameter scores were summarized in Dataset S1. However, considering our data size, we have 36 strains, 13 conditions with 4~7 levels and five parameter scores for each condition. To have sufficient resolution, in total there should be 36 x 13 x 5 = 2,340 box plot panels with 5~7 columns each. So we did not prepare all the figures for raw parameter scores but listed them in tables.
Figure 3A. and line 368-375 of supplemental items.
The preprocessing and transformation of HCA is not sufficiently described. Please describe in detail. Because data of MS is relative abundance, this process is quite important. And, what the "scaled to the biological range" mean?
The definition of "the biological range" was introduced by a previous literature in the metabolomic data normalization (van den Berg et al., 2006). We now added this citation and modified our description in detail under the section "Compound class and pathway assignment" as: "Metabolite data was transformed before calculating the contribution to each group of pathways and/or metabolite classes. Both intracellular

and extracellular metabolites were scaled to the biological range [5]. Intracellular metabolite data was transformed by subtracting the mean of the data set, followed by division with the difference between the maximum and minimum value of the particular metabolite. This difference is referred to as the biological range and makes all metabolites equally important [5]. Extracellular metabolites were first centered by the starting amount prior to scaling to the biological range, and were grouped according to compound classes as well as if they were produced (scaled value > 0) or consumed (< 0). Metabolites belonging to one of the selected classes and had positive or negative values were added together after scaling, respectively, to show production and consumption. For both intracellular and extracellular metabolites, scaled values of metabolites belonging to a pathway or compound class were added together for each strain to create group variables.The sum was subsequently divided by the square root of the number of metabolites to be comparable with the other classes. Hierarchical clustering was applied to the group variables using Euclidian distance between the strains."

Figure 3A.

How the compound class determined? And, what do metabolites belong to the classes?

To describe this, a new section names "Compound class and pathway assignment" was inserted to Supplemental Note S3: "Compounds identified were assigned to different classes and pathways by the pathway information in YMDB (Yeast Metabolic Database) (Jewison et al., 2012). These pathway and compound class assignment were summarized in Dataset S2.".

Line 309-325 of supplemental items.

I think the metabolome data of ethanol is not mentioned in this study.

Thank you for your correction. We did not use the ethanol media so we have removed all regarding descriptions in Supplemental Note S3 now.

Line 379-380 of supplemental items.

In Ref.4 you mentioned, the level of extracellular metabolites used. Your study also uses them? If so, please add the data used to calculate the flux as supplemental material.

Ref. 4 refers the starting model of yeast we used for the strain-specific models, which did not actually used extra metabolomic data. The metabolomics data used for our models are already included in Dataset S2. And the regarding raw data are now available on MetaboLights with accession number: MTBLS780. The public date was set to 28/12/2018, which is the most recent date we can choose.

Minor point

Line 97-115.

The stress responses are sometimes affected by growth condition such as aeration condition and medium composition. I recommend describing the conditions briefly in body text, and if possible, discuss about it in discussion.

Thank you for this suggestion. The growth medium used in the current study is a standard defined mineral medium extensively used in yeast studies. Aeration was the same in all conditions for phenotypic characterization by using the Growth Profiler. As the size of the study was already considered quite large, it was actually not within the scope of the study to include variation in the growth medium nor the aeration conditions. But sure, expanded studies with altered aeration condition or medium composition will be interesting, following our technical route. Now we added the following statement in the main text: "Besides parameter selection and weighting, our phenotypic scoring method could also be expanded to different industrially relevant processes with customized set-ups: e.g., different medium composition and aeration condition, which is not limited to the standard medium and condition used in this proof-of-concept study."

line 154 -156.

Please show the level of compound identification based on Metabolomics Standards Initiative (see table1 of Metabolites2018,8,31)

We have prepared the standard MetaboLights submission, with accession number: MTBLS780. The public date was set to 28/12/2018, which is the most recent date we can choose. And we also included an MAF table (Metabolite Assignment File) in Dataset S2, so readers can find ChEBI ids for those metabolites.

Line 383-423

If possible, compare the genes you selected with previous study of single gene deletion collection, such as the study below.

(FEMS Yeast Research, Volume 9, Issue 1, 1 February 2009, Pages 32-44)

Powered by Editorial Manager® and ProduXion Manager® from Aries Systems Corporation

We do observe genes mentioned in the literature to appear as significant in our analysis, thus we added the following sentence -as such an example- in our discussion: “SKY1 was also a significant gene shared by 4 conditions, which has been proved to be associated with osmotic tolerance (Yoshikawa et al., 2009)”.
Figure 3A. In HCA what type of clustering method is used?
We added the following statement to the figure 3 legend: “Hierarchical clustering was applied to the group variables using Euclidian distance between the strains. For data normalization before visualization, see details in Supplemental Note S3.”
Figure 3C.
Please add the unit of vertical axis.
The unit is mmol/gDW/h and we have now added it to the figure.

Reviewer #3:
In this study, Kang et al. performed whole genome sequencing, characterized growth under 13 industrially relevant conditions, performed metabolomics and GWAS analyses on 36 S. cerevisiae strains to identify potentially important genes implicated in stress tolerance/ resistance. The rationale for this study is well-defined. While other studies that utilize natural genetic variation between strains have a larger collection of strains across a wide geographic and phylogenetic distribution (e.g. 165 strains in Sardi M et al 2018, PMC5849340), Kang et al. utilizes a multi-OMIC approach to characterize these 36 strains deeply. Additionally, these authors performed a detailed growth characterization of these 36 strains, with thoughtful considerations outlined in the Parameter Influence Analysis. Overall, the paper seems appropriate for GigaScience given its editorial guidelines. Below, I have a few comments that can be addressed with minor revisions to the manuscript.
We would like to thank this reviewer for his/her positive comments.
Major points:
1. Given the importance of the growth data that defines strain robustness and performance, where will the raw growth data underlying the 5400 growth curves along with the in-house MatLab scripts deposited?
We have uploaded two self-developed pipelines to Github and SciCrunch: the metabolomic data processing pipeline, Metab 1.0 (https://github.com/reinkk/Metab, SCR_016877) and the growth curve processing pipeline, Growth-Profiling-Toolbox 1.0 (https://github.com/reinkk/Growth-Profiling-Toolbox, SCR_016878). And the raw OD data were also included as Dataset S7. The software repositories were now mentioned in the manuscript as “Raw data for growth curves in G-values (Dataset S7) were analyzed automatically using self-developed Matlab® scripts: Growth Profiling Toolbox (GitHub repository: https://github.com/reinkk/Growth-Profiling-Toolbox, with SciCrunch ID SCR_016878).”.
2. Along the same line of inquiries, I was not able to access the genomic data associated with the SRA accession number that was provided in the manuscript.
We now made our submission public.
3. Furthermore, while I am not a metabolomics expert, I know that the metabolomics field has developed Metabolomics Standards Initiative to improve reproducibility. It is not clear whether the authors follow this guideline. There were also no accession number associated with the metabolomics data. Have the authors deposited the data to databases such as MetaboLights?
We have prepared the standard MetaboLights submission, with accession number: MTBLS780. The public date was set to 28/12/2018, which is the most recent date we can choose. And we also included an MAF table (Metabolite Assignment File) in Dataset S2, so readers can find ChEBI ids for those metabolites.
4. The use of base modifications (BMs) to describe genetic variations such as SNPs or INDELs is inappropriate. In general, base modification is used to describe methylation, and as far as I could gather from the methods section, the authors did not perform epigenetic analyses or PacBio sequencing.
We modified it as “SNPs/InDels” throughout the text.
5. Since accurate SNP calling is important for the GWAS, I have a few comments and questions about the choice of these filters in GATK:
a. It's worthwhile to explicit state the filtering strategy (I did not see it in the supplemental file) and had to dig it out by looking up the reference to a previous paper: pubmed/26255308
b. Is there a reason why the authors chose to use UnifiedGenotyper as GATK has deprecated this tool due to this caller being very "aggressive in calling variants in order to be very sensitive"? The current recommendation by GATK is to use HaplotypeCaller

| | |
|---|---|
| | that reduces false positives, especially on INDELs.<br>c. This is related to the concerns of false positive SNPs, where the authors use VariantFiltration (parameters:-filterExpression 'ReadPosRankSum<−8.0 \|\| FS>10.0'). What is the rationale for lowering the FisherStrand (FS) value below recommendations by GATK? According to GATK, most of the variants that fail have an FS value greater than 55, hence the recommendation of failing variants with an FS value less than 60.<br>We are sorry that we have made a mistake here and thank you so much for your correction. We checked our script and we indeed used a new pipeline since GATK suggested HaplotypeCaller after 2014, which is different with the cited paper when data were processed before HaplotypeCaller becoming a golden standard. The section has been rewritten now as: "SNPs/InDels were called and filtered using the Genome Analysis Toolkit (GATK) 3.5 (DePristo et al., 2011; McKenna et al., 2010), with the sequential steps to include RealignerTargetCreator, IndelRealigner, HaplotypeCaller (with parameter "-rf BadCigar") and VariantFiltration (with parameter "--filterExpression "DP < 10 \|\| QD < 2.0 \|\| FS > 60.0"")".<br>d. The authors cite Strope PK et al (2015) as a support that the numbers of genetic variants they identified in this study is comparable to previous studies (p9, lines 178-180). However, Strope PK observed an average of 78,184 SNPs and 7840 INDELs from 100 genomes, compared to the current study of 342,325 SNPs and 19,347 INDELs. Given the relaxed filter used for variant calling and the 2-4X more variants identified, I think it's important to make sure that these variants are not false positives.<br>S. cerevisiae strains are quite divergent in the genome. The reason why Strope PK et al (2015) has a lower number of SNPs/InDels is that the 100 strains they selected are extremely close to the reference strain S288c, and 84 of the 100 strains are exactly in the same clade with S288c in the phylogenetic tree and were called "S288c type". Also, their number indicates the SNP count per strain, while our 342,325 are all SNPs loci identified in the whole strain collection. For each strain, we have 68,928 SNPs/InDels in average, which is quite close to Strope PK et. al (78,184 + 7,840, as they did not applied the strict filtering as we did). Now we cited a more comparable study (similar strain collection but with lower sequencing depth) where they have 235,127 SNPs (Liti et al., 2009b) and modified our description to "By using S288c as the reference genome, we identified 342,325 SNP loci, 19,347 small insertion and 17,457 small deletion (InDel) loci among all strains (Table S1 & Dataset S3), with an average SNP/InDel count of 68,928 per strain".<br>Furthermore, as described in our manuscript, we applied MAF > 5% (at least 4 alleles in our case) before LD block building and GWAS. Also, we selected only core-genome SNPs/InDels for GWAS, which resulted in only 165,358 loci left. We believe these SNPs/InDels are confident for down-stream GWAS.<br>6. What is the rationale for choosing a low MAF filtration (MAF > 0.5%) for the core-genome GWAS (pg 34, line 483)? What does the MAF distribution look like for these identified core-genome SNPs? In general, the GWAS studies in yeast tend to use MAF > 2% - 25%, as the power to detect association with rare variation is limited.<br>Sorry for this typo in Note S5 and now we corrected it. Actually we used MAF > 0.05, which is 5% but not 0.5%. We now modified all sections mentioning MAF to the format "5%" to avoid misunderstanding.<br>Minor points:<br>1. In Supplemental Note S2, the authors describe how they extract growth parameters of interest in great details. Could the authors add in the rationale for calculating the average growth rate using a time point between 50 to 501 time points (pg 14, line 165)? More specifically, does this time point capture the linear range of the growth curve and time point 50 is after the lag phase of even the most stressed cells?<br>The time point was selected during exponential phase. So for the most stressed cell, the time point was much later than the cells under less stress. For the least stressed cells, the time point is close to 50, otherwise that would be a much later time point. We added "to ensure the time point was in exponential phase" in the relevant description.<br>2. In Supplemental Dataset S1, the authors need to define the color coding for parameter influence.<br>We added "P1~P5 stand for: 1. Lag phase; 2. Growth duration; 3. Number of generations during the growth phase; 4. Average specific growth rate during the growth phase; 5. Maximum specific growth rate." to the figure legend.<br>3. In general, the figures are aesthetically pleasing and well designed. However, it is important to keep in mind of color blindness (e.g. Fig 2A uses red and green colours to denote the strains)<br>Thank you for your suggestion. We admit that we have not thought about colour |

Powered by Editorial Manager® and ProduXion Manager® from Aries Systems Corporation

| | |
|---|---|
| | blindness and we will pay more attention in the future.<br>4. Need to properly cite R as per CRAN's guideline https://cran.r-project.org/doc/FAQ/R-FAQ.html#Citing-R<br>Thank you for pointing this out, the R Core Team has been cited now.<br><br>References<br><br>Connelly, C.F., Skelly, D.A., Dunham, M.J., and Akey, J.M. (2013). Population Genomics and Transcriptional Consequences of Regulatory Motif Variation in Globally Diverse Saccharomyces cerevisiae Strains. Molecular Biology and Evolution 30, 1605-1613.<br>DePristo, M.A., Banks, E., Poplin, R., Garimella, K.V., Maguire, J.R., Hartl, C., Philippakis, A.A., del Angel, G., Rivas, M.A., Hanna, M., et al. (2011). A framework for variation discovery and genotyping using next-generation DNA sequencing data. Nature genetics 43, 491-498.<br>Diao, L., and Chen, K.C. (2012). Local ancestry corrects for population structure in Saccharomyces cerevisiae genome-wide association studies. Genetics 192, 1503-1511.<br>Jewison, T., Knox, C., Neveu, V., Djoumbou, Y., Guo, A.C., Lee, J., Liu, P., Mandal, R., Krishnamurthy, R., Sinelnikov, I., et al. (2012). YMDB: the Yeast Metabolome Database. Nucleic Acids Res 40, D815-820.<br>Liti, G., Carter, D.M., Moses, A.M., Warringer, J., Parts, L., James, S.A., Davey, R.P., Roberts, I.N., Burt, A., Koufopanou, V., et al. (2009a). Population genomics of domestic and wild yeasts. Nature 458, 337-341.<br>Liti, G., Carter, D.M., Moses, A.M., Warringer, J., Parts, L., James, S.A., Davey, R.P., Roberts, I.N., Burt, A., Koufopanou, V., et al. (2009b). Population genomics of domestic and wild yeasts. Nature 458, 337-341.<br>McKenna, A., Hanna, M., Banks, E., Sivachenko, A., Cibulskis, K., Kernytsky, A., Garimella, K., Altshuler, D., Gabriel, S., Daly, M., et al. (2010). The Genome Analysis Toolkit: a MapReduce framework for analyzing next-generation DNA sequencing data. Genome research 20, 1297-1303.<br>Monk, J.M., Koza, A., Campodonico, M.A., Machado, D., Seoane, J.M., Palsson, B.O., Herrgard, M.J., and Feist, A.M. (2016). Multi-omics Quantification of Species Variation of Escherichia coli Links Molecular Features with Strain Phenotypes. Cell Syst 3, 238-251 e212.<br>Peter, J., De Chiara, M., Friedrich, A., Yue, J.X., Pflieger, D., Bergstrom, A., Sigwalt, A., Barre, B., Freel, K., Llored, A., et al. (2018). Genome evolution across 1,011 Saccharomyces cerevisiae isolates. Nature 556, 339-344.<br>van den Berg, R.A., Hoefsloot, H.C., Westerhuis, J.A., Smilde, A.K., and van der Werf, M.J. (2006). Centering, scaling, and transformations: improving the biological information content of metabolomics data. BMC Genomics 7, 142.<br>Warringer, J., Zörgö, E., Cubillos, F.A., Zia, A., Gjuvsland, A., Simpson, J.T., Forsmark, A., Durbin, R., Omholt, S.W., Louis, E.J., et al. (2011). Trait Variation in Yeast Is Defined by Population History. PLoS Genetics 7, e1002111.<br>Yoshikawa, K., Tanaka, T., Furusawa, C., Nagahisa, K., Hirasawa, T., and Shimizu, H. (2009). Comprehensive phenotypic analysis for identification of genes affecting growth under ethanol stress in Saccharomyces cerevisiae. FEMS Yeast Res 9, 32-44. |
| **Additional Information:** | |
| **Question** | **Response** |
| Are you submitting this manuscript to a special series or article collection? | No |
| **Experimental design and statistics**<br><br>Full details of the experimental design and statistical methods used should be given in the Methods section, as detailed in our Minimum Standards Reporting Checklist. Information essential to interpreting the | Yes |

| | |
|---|---|
| data presented should be made available in the figure legends.<br><br>Have you included all the information requested in your manuscript? | |
| **Resources**<br><br>A description of all resources used, including antibodies, cell lines, animals and software tools, with enough information to allow them to be uniquely identified, should be included in the Methods section. Authors are strongly encouraged to cite Research Resource Identifiers (RRIDs) for antibodies, model organisms and tools, where possible.<br><br>Have you included the information requested as detailed in our Minimum Standards Reporting Checklist? | Yes |
| **Availability of data and materials**<br><br>All datasets and code on which the conclusions of the paper rely must be either included in your submission or deposited in publicly available repositories (where available and ethically appropriate), referencing such data using a unique identifier in the references and in the "Availability of Data and Materials" section of your manuscript.<br><br>Have you have met the above requirement as detailed in our Minimum Standards Reporting Checklist? | Yes |

Revised Manuscript with Track Changes Click here to access/download;Manuscript;SC_Main_tracing.docx

Click here to view linked References

# Linking genetic, metabolic and phenotypic diversity among *S. cerevisiae* strains using multi-omics associations

Kang Kang[1,7†], Basti Bergdahl[2†], Daniel Machado[3,4], Laura Dato[2], Ting-Li Han~~Han Morgan~~[5], Jun Li[1,7], ~~Laura Dato[2], ~~Silas Villas-Boas[5], Markus J. Herrgård[2*], Jochen Förster[2*,#] and Gianni Panagiotou[1,6,7*]

† Authors contributed equally to this work

* Corresponding author: herrgard@biosustain.dtu.dk , Jochen.Forster@carlsberg.com , Gianni.Panagiotou@hki-jena.de

Author affiliations:

1. Systems Biology & Bioinformatics Group, School of Biological Sciences, The University of Hong Kong, Hong Kong S.A.R., China
2. The Novo Nordisk Foundation Center for Biosustainability, Technical University of Denmark, Kgs.Lyngby, Denmark
3. Department of Biological Engineering, School of Engineering, University of Minho, Braga, Portugal
4. The European Molecular Biology Laboratory (EMBL), Heidelberg, Germany
5. Centre for Microbial Innovation, School of Biological Sciences, University of Auckland, Auckland, New Zealand
6. Department of Microbiology, Li Ka Shing Faculty of Medicine, The University of Hong Kong, Hong Kong S.A.R., China

7. Systems Biology & Bioinformatics Unit, Leibniz Institute for Natural Product Research and Infection Biology – Hans Knöll Institute, Jena, Germany

# Present address: Carlsberg A/S, Carlsberg Research Laboratory, 1799, Copenhagen V, Denmark

* Correspondence:

Gianni Panagiotou, Systems Biology & Bioinformatics Unit, Leibniz Institute for Natural Product Research and Infection Biology, Hans Knöll Institute, Jena, Germany. Email: gianni.panagiotou@hki-jena.de. Address: Adolf-Reichwein-Straße 23, 07745 Jena, Germany. Phone: +49 3641 532-1759

## ABSTRACT

The selection of bioengineering platform strains and engineering strategies to improve the stress resistance of *Saccharomyces cerevisiae* remains a pressing need in bio-based chemical production. Thus, a systematic effort to exploit the genotypic and phenotypic diversity to boost yeast's industrial value is still urgently needed. Here, we analyzed 5400 growth curves obtained from 36 *S. cerevisiae* strains and comprehensively profiled their resistances against 13 industrially relevant stresses. We observed that bioethanol and brewing strains exhibit higher resistance against acidic conditions, however, plant isolates tend to have wider range of resistance, which may be associated with their metabolome and fluxome signatures in TCA cycle and fatty acid metabolism. By deep genomic sequencing we found that industrial strains have more genomic duplications especially affecting transcription factors, presenting disparate evolutionary paths in comparison to the environmental strains which have more InDels, gene deletions and strain-specific genes. Genome-wide association studies coupled with protein-protein interaction networks uncovered novel genetic determinants of stress resistances. These resistance-related engineering targets and strain rankings provide a valuable source for engineering significantly improved industrial platform strains.

## KEYWORDS

*S. cerevisiae*, Multi-omic study, Platform strain, Stress resistance, Geno-to-phenotype association

## BACKGROUND

We are facing a paradigm shift, where our economy and industrial processes need to exchange current oil-based technologies with new and sustainable biotechnologies. The transition to a bio-based economy has already begun as several products have reached commercial production scale, e.g. cellulosic ethanol, succinic acid, lactic acid, acetic acid, itaconic acid, 1,3-propanediol, 1,4-butanediol, farnesene and ABE (Acetone, n-Butanol, Ethanol) [1, 2]. In order to build a microbial cell factory to convert sugars or other carbon sources into bio-products, the first step is to select a host organism with metabolic and physiological properties suitable for the intended bioprocess. These properties include tolerance to substrates, products and byproducts in high concentration, and resistance to the abiotic stresses including low/high pH, high temperature, osmotic stress etc. during the industrial fermentation.

Most current metabolic engineering projects are carried out using laboratory strains of bacteria [3] or yeast [4] as the starting host. Although such strains are easily manipulated genetically, they do not always meet the requirements set by stressful industrial fermentation conditions [5]. In order to improve the stress resistance of the host organism, different strategies have been adopted: (1) Comparative transcriptomic or proteomic studies [6]; (2) Directed evolution of the host genome under extreme conditions [7]; (3) Knowledge-based engineering [8]. Nevertheless, all these methods are neglecting the abundant genetic diversity present in environmental or industrial strains by focusing on narrow genetic resources (limited gene candidates, random mutations, and simple genetic changes) in a single host laboratory strain. Global screening incorporating genetic variation present in a broader strain collection could significantly speed up development of improved industrial production strains.

Here, we aim to address the scientific and engineering challenges outlined above through a *Sacharomyces cerevisiae* population multi-omics approach. We explored the diversity of 36 industrial, environmental, clinical and laboratory strains of *S. cerevisiae* regarding the genetic composition,

metabolic properties -metabolite levels and predicted flux distributions- and resistance to 13 industrially relevant stress conditions. To characterize and rank the strains' industrial values in stress resistance, we defined two scores – Robustness and Performance, calculated from five growth parameters with variable weights to meet different bioengineering purposes. The outcomes of this study include: (1) Identification of strains with multiple or specific stress-resistance as potential platform strains for cell factory construction; (2) Construction of strain-specific metabolic models explaining the divergence in metabolic phenotypes; (3) Establishing novel resistance phenotype-genotype links including suggestions of potential engineering targets to boost industrially relevant phenotypic properties.

## DATA DESCRIPTION

We performed a multi-omic study on a collection of 36 *S. cerevisiae* strains, including natural and industrial strains with different geographical origins. The genomes of these strains were sequenced with high depths (> 50x) and the variant profiles and phylogenetic tree were constructed. Besides, intra- and extracellular metabolomes were measured. Strain-specific Genome-scale Metabolic Models (GSMM) were constructed to predict the fluxomes of all the strains. Regarding phenome, the strain collection was exposed to 13 industrially relevant stresses (including low pH, high temperature and various of inhibitory compounds) in different inhibitory levels. We analyzed over 5400 growth curves to score the strains in two phenotypic scores: Robustness and Performance. Genome-wide association studies (GWAS) were performed to establish the geno-to-phenotype associations and the Protein-Protein Interaction Network (PPIN) modules associated to the stress resistance were built. Suggestions for potential engineering targets to improve the stress resistances of the bioengineering platform strain were given based on the multi-omic study.

## ANALYSES

### Systematic screening of resistance to multiple bioprocessing relevant stress conditions

In this study, we used a collection of 36 *S. cerevisiae* strains from various geographical and isolation origins (**Supplemental Note S1**). Strains were classified into four types: 17 industrial strains (from ethanol production, food and brewing industries), 13 environmental strains (isolated from soil, plant and animals), 4 laboratorial and 2 clinical strains (vaginal isolates). To investigate the phenotypic diversity in the strain collection, all strains were exposed to 13 different stress conditions including 11 inhibitory compounds, acidic pH and high temperature (**Figure 1A**). Acidic pH and the conditions using acids as inhibitory compounds were classified as acidic conditions. In each condition, the cells were exposed to the inhibitory compound at between four and seven distinct levels resulting in different numbers of strains that exhibit measurable growth (**Figure 1A & Supplemental Note S2**). In total, we analyzed more than 5400 growth curves and five growth parameters were extracted from each growth curve to score the strains in two phenotypic traits: Robustness and Performance (**Figure 1B, Supplemental Note S2 & Dataset S1**). Briefly, Robustness measures the ability of the strain to withstand increasing inhibitory levels whereas Performance measures how well a particular strain compares to other strains in a particular stress condition. The resistance scores were calculated in each inhibitory level and then integrated as final strain rankings for each condition (**Figures 2A-B & Figure S1, Supplemental Note S2**). For instance, strain DBVPG1373 had poor Performance scores under 1,4-butanediol compared to other strains but has high Robustness according to its ability to maintain its growth parameters when inhibitory levels increase; while PW5 is a counter-example with high Performance but low Robustness score due to the sharp decrease in fitness when the inhibitory levels increased (**Figure 1C**).

The strain Y55, a laboratory strain isolated from grape, was identified as the most resistant strain to multiple conditions, followed by two plant strains, RM11 and PW5 (**Figure 2A**). These strains with

plant origins could be potentially used as platform strain candidates but seldom considered in previous bioengineering endeavors [9]. Interestingly, six out of the top ten strains are industrial strains (3 bioethanol strains), but nonetheless, the industrial strains are not significantly better than environmental strains overall in multiple stress resistances. The two most commonly used laboratory strains CEN.PK113-7D and S288c, obtained low ranking values (> 6) in most conditions, with notable exceptions only the resistance to fumaric and pyruvic acids, respectively (**Figure 2B**).

Furthermore, diverse resistance patterns were observed for different strains (**Figure 2B**), which could also assist the host selection for specific production purposes: e.g., S288c and Y55 are two laboratory strains with overall bad and good universal resistance, respectively; CA1, a Brazilian bioethanol strain, shows high resistance to most acidic conditions, while the plant strain T7 is only resistant to non-acidic conditions; another bioethanol strain, CLIB215, has much higher Robustness than Performance rankings in most conditions; on the contrary, the Performance overweighs Robustness for the plant strains PW5 and RM11. When comparisons were made between industrial and environmental strains (Brazilian bioethanol strains binned as one candidate, **Supplemental Note S1**; Wilcoxon rank-sum test), the industrial strains show noticeably higher rankings in Robustness against acidic conditions (*P* value = 6.5e-4, also significant in 4 individual rankings), while the environmental strains score better in the Performance under non-acidic conditions (*P* value = 4.0e-2, and also significant in one individual ranking under 1,4-butanediol) (**Figure 2C**). Zooming into subcategories of the strains, bioethanol and food strains but not brewing ones have higher Robustness against acidic conditions (including low pH, formic acid and 4−aminobenzoic acid as significant individual rankings), while the Performance under non-acidic conditions was led by plant but not soil strains (Kruskal-Wallis test, *FDR* < 0.1). Different industrial strain subcategories also performed differently in divergent conditions: for instance, bioethanol

and food strains showed higher resistances to multiple conditions such as formic acid and 4−Aminobenzoic acid, while brewing strains showed high Robustness against fumaric acid.

With the objective to present the concept of how strains' physiological traits could be ranked, the above results were based on giving equal weights to the five growth parameters in the calculation of the Robustness and Performance scores. To gain further insights into the sensitivities of the rankings to weighting different parameters, Parameter Influence Analysis (PIA) was performed (**Supplemental Note S2**). A case study shows that two strains with significant growth curve differences could still have close rank positions when giving specific parameter weights (e.g., the Performance ranks of Y55 and YPS128 under 1,4-butanediol) (**Figure S2**). Such information can be useful when selecting a host strain for specific process by setting different weights to different growth parameters, e.g. lag phase would be less important selection criteria for a continuous fermentation process and subsequently be given a low weight, however, in a batch fermentation process, lag phase duration becomes critical and could be assigned with a higher weight. ~~Such information can be useful when selecting a host strain for specific process by setting different weights to different growth parameters, e.g. lag phase and average growth rate would be less important selection criteria for a continuous fermentation process~~Besides parameter selection and weighting, our phenotypic scoring method could also be expanded to different industrially relevant processes with customized set-ups: e.g., different medium composition and aeration condition, which is not limited to the standard medium and condition used in this proof-of-concept study. ~~than the other three parameters.~~

**Formatted:** Font: Not Italic

**Formatted:** Font: Not Italic

**Metabolomic profiling and correlations between metabolomics and stress resistance phenotypes**

The metabolic characteristics of all strains were investigated by determining both intra- and extracellular metabolomes by GC-MS (**Supplemental Note S3**). In both analyses, 79 metabolites were

identified and quantified in terms of relative abundance (**Dataset S2**). The metabolomic data were used to group the yeast strains according to metabolite abundance in relevant pathways and/or compound classes (**Figure 3A, Dataset S2**). Overall, negative correlations could be observed between the intra- and extracellular metabolite abundances (**Figures 3A & S3**). Significant negative correlations were captured between the extracellular abundance of proteinogenic amino acids and all intracellular compound classes except fatty acids (Spearman's correlation test with Bonferroni adjustment, $FDR < 0.05$). The analysis also showed that the industrial strains (particularly bioethanol and brewing strains) have noticeably lower extracellular metabolite levels than the environmental strains, especially for aromatic compounds and carboxylic acids (Wilcoxon rank-sum test, $FDR < 0.1$, **Figure 3B**). The greatest difference was actually observed in the carboxylic acids that could be imported and consumed as carbon sources by yeast cells. Industrial strains, and especially brewing strains show generally high intracellular concentrations in Carboxylates Degradation and TCA Cycle pathways (Wilcoxon rank-sum test, $FDR < 0.1$). While conversely, higher intracellular levels were observed for Fatty Acid Biosynthesis for plant strains (**Figure 3B**). Interestingly, as the major membrane lipid component, fatty acids (saturated fatty acids in particular) are highly associated with non-acidic stress response (ethanol, salt, oxidative and thermal stresses) in plants and fungi [10]. Thus, in the resistance to non-acidic conditions such as alcohol and heat, plant strains could outperform the industrial ones due to the biosynthetic activity, cellular abundances and composition of intracellular fatty acids, which aligns with our observations in resistance scores very well (**Figure 2C**).

**Genome diversity among industrial and natural *S. cerevisiae* strains**

We sequenced the genomes of all 36 strains to a minimum depth of 52x, with a median of 96x. By using S288c as the reference genome, we identified 342,325 SNP locis, 19,347 small insertions and

17,457 small deletions (InDels) loci as unique base modifications (BMs) among all strains (**Table S1** & **Dataset S3**), with an average SNP/InDel count of 68,928 per strain, which were comparable to previous studies [11-13]. In the constructed phylogenetic tree, three major clades were discovered (**Figure 4A**). We could observe an obvious enrichment of industrial strains in the clade of S288c (13 out of 20 strains, 65.0%). Notably, all the seven strains isolated from Brazilian bioethanol industry with distinct origins and genotypic categories [14] were clustered in this branch (within purple shade in **Figure 4A**). This suggests the possibility of common ancestors, while the long pairwise genetic distances also indicate high divergences in their strain-specific genetic makeups and evolutionary paths. All the six strains with high heterozygosity rate, including CLIB324, GDB135-h, T73 (97.7 ~ 98.3%, brewing or bakery strains), AL1, GDB325 and GDB379 (42.1 ~ 52.4%, all bioethanol strains), were present in the same subgroup, while all the other non-haploid strains' base modificationsSNPs/InDels were generally in homozygous form (heterozygosity rate < 15%). These six industrial strains have shown some unique resistance to acids: CLIB324 was the most resistant strain (in both Performance and Robustness) to formic acid and the most Robust against pyruvic acid; T73 shows the highest Performance under pyruvic acid whereas AL1 was the most resistant to ferulic acid (**Figure 2A**). More interestingly, all seven strains in the other subgroup of the same clade (YJM978 – L.1528), were also among the top eight strains with highest InDel rates (> 9.0%). On the contrary, more environmental strains were found in the clade of T7 (7 out of 9 strains, 77.8%, within yellow shade in **Figure 4A**). The SK1 clade was the most divergent cluster. Y55 and PW5, two of the top three strains with universal resistance, locate in this cluster with long genetic distances from other strains. The only two industrial strains in this cluster, DBVPG6044 and NCYC110, had generally poor stress resistance (except DBVPG6044's Robustness against succinic acid) (**Figure 2A**) distinguishing them from Y55 and PW5 from the same clade.

The analysis of copy-number variations (CNVs) resulted in the identification of 2,916 unique CNV-affected genes in total (**Table S1** & **Dataset S3**), while most of the large-scale CNVs are located in subtelomeric regions, in agreement with a recent study [15]. Chromosome I duplication was discovered in five strains (four environmental and one industrial), while all other strain-specific chromosome-scale duplications were captured in industrial strains (**Figure S4A**): NCYC110 – chrV, CLIB215 – chrXII, GDB135-h – chrIII and GDB325 – chrVI. PW5 (environmental) and DBVPG6044 (industrial, triploid) are also the two strains that have large number of duplicated genes (> 200). In addition, extremely high copy-numbers of tandem repeat segments (> 10) were observed in seven strains, with four of them being bioethanol strains (AL1 – 82x in chrI, CLIB382 – 11x in chrVII, Ethanol Red – 18x in chrX, GDB325 – 34x in chrIX), together with one food (KKYS2-h – 13x in chrVI), one plant (RM11 – 22x in chrVII) and one clinical (YJM975 – 701x in chrX and 19x in chrXIV) strain. Except NCYC110, all the eleven aforementioned strains have good resistance (especially Robustness) to one or more acidic conditions (**Figure 2A**). In comparison, chromosome-scale loss events were only captured in chrI of YJM978, a clinical strain. The laboratory strain SK1 was another strain with large number of gene losses (> 800) and this strain was also the most resistant strain to acetic acid (**Figure 2A**).

Our *de novo* assembly and gene prediction suggested 7 to 25 potential novel non-S288c genes per strain, and most of these genes were non-metabolic genes and homologous genes from other *Saccharomyces* strains (**Dataset S3**). Five out of the eight strains with over 20 new genes were environmental strains. The strains CLIB382, UWOPS05-227.2 and UWOPS05-217.3 had the largest counts of new genes, whereas CLIB382 was also the most resistant strain to fumaric acid and 4-aminobenzoic acid (**Figure 2A**). Ten new genes were shared by UWOPS05-227.2 and UWOPS05-217.3 (both related with nectar of Bertram palm), which were also sharing the high resistance traits towards ferulic acid and 1,4-butanediol (**Figure 2A**). As these genes were absent in the reference strain S288c

and largely uncharacterized, they have not been previously reported to be associated with the aforementioned phenotypes.

In the systematic comparisons between the genetic makeups of industrial and environmental strains (Brazilian bioethanol strains binned as one candidate, **Supplemental Note S1**), significantly more heterozygous ~~BMs~~ SNPs/InDels (Wilcoxon rank-sum test, *P* value = 4.1e-2), especially in bioethanol strains, and less InDels (*P* value = 3.6e-2) were observed in industrial strains (**Figure 4B-D**). When investigating the genes influenced by these variants (the ratio of ~~BMs~~ SNPs/InDels falling in specific gene categories, or the number of genes affected by CNVs), we found relatively more ~~BMs~~ SNPs/InDels in the transcription factor (TF) super family Helix-turn-helix (HTH) in all subcategories of industrial strains (*P* value = 1.7e-2) (**Figure 4C-D**). We also found significantly more duplicated TFs in industrial strains (bioethanol and brewing strains in particular), together with three TF super families, Zipper (ZIP), HTH and Other (TF not belonging to any of the ZIP, HTH nor Zinc finger (ZNF) super families) (**Figures 4C-D**, *P* value < 0.05). More gene deletions were observed in plant strains (Kruskal-Wallis test, *P* value < 0.05, **Figure 4D**), and more interestingly, deletions of TFs were only observed in plant species.

**Phenotypic prediction using reconstructed strain-specific genome-scale metabolic models**

In order to analyze metabolic differences between the strains and predict metabolic phenotypes, strain-specific Genome-scale Metabolic Models (GSMMs) were reconstructed by incorporating severe mutations, gene deletions and non-S288c genes (**Dataset S4, Supplemental Note S4**). The pairwise comparison of the strain-specific models shows that the networks differ by at most only 2% of total reactions (**Supplemental Note S4**). The models were able to predict strain differences in the utilization of 30 carbon sources and 5 nitrogen sources (**Supplemental Note S4**) in comparison with the experimental data for selected strains (**Dataset S5**). Due to a recent study [16], inter-strain differences

could be observed in key metabolic fluxes simulated by strain-specific models, which could provide more meaningful biological insights than the reaction presence/absence comparisons. Thus, dDifferent activated fluxes were simulated by the strain-specific models (**Supplemental Note S4**). Higher fluxes of Pyruvate Mitochondrial Transport via Proton Symport and Pyruvate Dehydrogenase were observed in environmental strains (Wilcoxon rank-sum test, *FDR* = 6.6e-2, **Figure 3C**), which are two key up-stream reactions before the TCA cycle, suggesting that environmental strains have relative higher energy flux from the pyruvate metabolism to the TCA cycle than industrial strains. When summing up the energy fluxes by different subsystems, we found that environmental strains also have higher fluxes in Fatty Acid Biosynthesis, Citric Acid Cycle (TCA Cycle) and Pyruvate Metabolism, while industrial strains have higher fluxes towards Fatty Acid Metabolism (degradation) (*FDR* < 0.1, **Figure 3C**).

**Identification of the genetic features and patterns associated with different stress resistance**

To identify the potential genetic contributors to stress resistance phenotypes, SNP-based and CNV-based genome-wide association studies (GWAS) were performed (**Figure 5**). For the SNP-based GWAS, to reduce the impact from the false positive SNPs that were called from the highly divergent genomic regions, the core genome regions shared by all strains were annotated by *de novo* assembled contigs, and only the base modificationsSNPs/InDels in the core genome were used as genotype markers (**Figure S4B & Supplemental Note S5**). In summary, 3,449 linkage disequilibrium (LD) blocks were identified, 165,358 base modification markers were used in the SNP-based core genome GWAS (Core-GWAS) and 880 CNV markers were used in the CNV-based GWAS. Significance cut-offs were obtained from the quantile-quantile plot of the *P* value distribution (**Supplemental Note S5**). In the SNP-based GWAS, significant markers for the Robustness and Performance rankings under non-acidic conditions were evenly distributed along the genome (**Figure 5B**), while the two rankings under acidic conditions

showed different genomic hotspots: e.g., chrII for Performance and chrXIII for Robustness. In the CNV-based GWAS, the Robustness against acidic conditions always presented condition-specific genomic hotspots (especially for gain events): e.g., chrI – furfural, chrXIV – succinic acid, chrIX – fumaric acid, chrVIII – acetic acid and chrIII – pH, all in gain events (**Figure 5C**). While for Performance, hot regions tend to be shared by multiple conditions: e.g., the loss events in chrV and chrVIII, and the gain event in chrXIV (**Figure 5C & Figure S4B**). In general, the GWAS profiles for the Performance under acidic and non-acidic conditions were relatively similar; on the contrary the profiles for the Robustness against acidic and non-acidic conditions are highly disparate (**Figure S4B**).

The Core-GWAS was successful in avoiding genomic regions with high variabilities (CNV regions, centromere, chromosome ends, break points of structural variations, mitochondrial DNA, etc.), thus the genes processed by the SNP-based and CNV-based GWAS were not highly overlapping: from the 1,818 and 2,931 genes treated in the SNP-based and CNV-based GWAS, respectively, only 794 genes were in the shared regions, while no gene was deemed significant from both SNP-based and CNV-based GWAS within the 26 individual rankings (**Figure S4B, Table S2 & Dataset S6**).

The patterns of the four GWAS profile groups (Acidic/Non-acidic × Robustness/Performance) were more obvious when studying the relative contribution from SNP/gain/loss events on specific gene categories (**Figure 6**). When looking at all genes sorted by GWAS *P* values and represented by the 75% quantile, the contribution patterns to the Robustness and Performance under non-acidic conditions were similar, while the Robustness against acidic conditions has relatively higher contribution from CNVs, especially gain events. In verified ORFs, the resistance to acidic conditions was slightly skewed to SNPs, while in uncharacterized genes, CNVs were presenting much higher contribution. Regarding transcription factors (TFs), in the TF super families ZNF and Other the resistances to acidic conditions were all highly contributed by gain events, which could be linked with the observation that the industrial

strains have more TFs duplicated and are generally more resistant to acidic conditions. In the TF family HTH, the Performance under acidic conditions was skewed to SNPs, compared with the Performance under non-acidic conditions. Meanwhile, higher base modification rate in the HTH family was discovered in industrial strains compared to environmental strains.

To validate the GWAS results with literature-based knowledge, we compared the GWAS outcomes with the phenotype-associated gene lists in Saccharomyces Genome Database (SGD) [17] (**Supplemental Note S5**). The significant gene lists from GWAS are highly noisy since several genes always appear contiguous in significant genomic regions (LD blocks or CNV regions). Therefore it is possible that most of the genes receiving the same significant *P* values are just “passengers”. In order to identify “driver” genetic variants in the GWAS gene lists, we mapped the genes to protein-protein interaction networks (PPIN) and identified the core modules in the network by ModuleDiscoverer [18]. In the presented PPI networks, the GWAS gene lists associated to resistances to acids, ethanol, thermotolerance and lifespan (an SGD phenotype associated with the measurement of all our resistance scores), were found to be highly overlapping with the genes already recorded in SGD entries, suggesting a successful literature-based validation (**Figure 7**). When making comparisons between the GWAS modules with the modules constructed by randomly selected gene lists with the same lengths (5 random gene lists for each GWAS gene list), GWAS modules constantly show significantly higher modularity (more nodes in modules, more nodes in cliques, more internal edges, higher proportion of foreground nodes, Wilcoxon signed-rank test, $P < 1e\text{-}2$) and higher associations with SGD entries (more edges to SGD nodes, higher overlapping rate, higher overlapping rate of foreground nodes, $P < 1e\text{-}2$). Furthermore, additional GWAS-specific modules (SGD genes not highly observed in such modules) were also discovered, which represent valuable candidates for engineering target selection. Some of these modules were shared by multiple networks: the *IMA*/*MAL* module was shared by all four networks; the large

*YRF*/Uncharacterized gene module was shared by lifespan, thermotolerance and resistance to acids; the *COX*/*ATP*/Mitochondrial gene module was shared by resistances to acids and ethanol; the *FRE*/*FIT* module was shared by thermotolerance and lifespan.

## DISCUSSION

The mechanisms influencing the yeast stress resistance are complex and the genetic interpretation require more systematical investigations. Our comprehensive omics study, incorporating the discoveries from phenome, genome, metabolome, fluxome, GWAS and the interactome, has been successful in establishing multi-dimensional associations among strain metadata, genotype and resistance to different conditions.

Among the studies with large-scale phenotype screening, previous works focused on the metabolome [11, 19] and basic physiology [11, 20], while in this work we also developed a systematic scoring and ranking protocol, to characterize the strains' industrial potential regarding resistance to multiple industrially relevant stresses with the possibility to set different weights to parameters according to different engineering purposes. Compared with previous association studies that aimed to establish genotype-to-phenotype links by using only SNPs [11, 21], or performed SNP-based GWAS in both core- and pan-genome region [15], here we introduced SNP-based core genome GWAS and CNV-based GWAS to reduce false positive observations introduced by the pan-genome diversity of the yeast population (common GWAS procedure), and take both ~~base modifications~~SNPs/InDels and structural variations into consideration. The reconstruction of strain-specific GSMMs and the prediction of energy fluxes provide a valuable resource for engineers working with different strains for different purposes.

To summarize our evidences in the phenotypic and genotypic screening and make some basic comparisons between industrial and environmental strains and among their subcategories, we have

established several interesting associations. Regarding genotype, industrial strains tend to have higher heterozygosity (especially bioethanol strains), which could be a result of genome fusion during fermentation processes, lower InDel rate, higher SNPs/InDels rate in the TF family HTH, and higher frequencies in large-scale and high-intensity duplication events (especially affecting transcription factors), and lower possibility to acquire new genes. At the phenotypic level, industrial strains have relatively low extracellular aromatic compound and carboxylic acid levels and lower pathway activity in TCA cycle but relatively high in ethanol metabolism, which meets their functionality in anaerobic fermentation. Consequently, the accumulation of aliphatic acids from TCA cycle, such as succinate and fumarate, may therefor contribute to the strains' high innate resistance (especially Robustness) to acidic conditions (bioethanol and food strains in particular). These resistances were found to be highly associated with duplication events, especially in uncharacterized genes and TFs, as well as the SNPs/InDels in the helix-turn-helix TF family. On the other hand, regarding the genotype, environmental strains tend to have lower heterozygosity but higher InDel rate, wider range of phylogenetic diversity, and higher frequencies in small-scale and random loss events, low possibility in large-scale duplications and higher tendency to acquire new genes – especially plant strains, where deletions of TFs were also only observed in this subcategory. Summarizing the phenotype, environmental strains, especially the plant species, tend to have relatively high extracellular metabolite levels, higher energy flux towards TCA cycle and high activity in fatty acid biosynthesis, which also improves the resistances in wide range. They are in general not outstanding in the Robustness to extreme conditions (especially the acidic ones) but tend to have wider ranges of resistance (especially the Performance) to multiple conditions, in particular the non-acidic conditions. These phenotypes have relatively high associations with gene loss events.

Regarding the selection of platform strains our study revealed that the most commonly used *S. cerevisiae* strains, S288c and CEN.PK113-7D, performed rather poorly among all screened strains with regard to the bioprocessing-relevant stress resistance. Tremendous amount of engineering work has been performed using these two strains as host strains [4, 5], but few evaluations have been made on whether they are the best possible ones to use in industrial settings. Our findings suggest that many environmental strains also have potential industrial applications due to their unique stress resistance patterns when compared with industrial strains. More impressively, none of the top three strains with resistance to multiple stress conditions, Y55, RM11 and PW5, was isolated from current industrial yeast fermentations but all were initially isolated from plants, and thus could serve as promising platform strains for bioprocessing purposes. The recent developments in CRISPR methods have also made it possible to genetically engineer almost any yeast strain [22], thus non-standard or polypoid strains could be introduced in metabolic engineering projects [23].

From the point of view of genetics and evolution, evidences from previous studies match our observations and assumptions quite well [24, 25]; from the engineering point of view, it has been reported that the duplication or overexpression of several TFs [26] and the mutagenesis of Spt15 [8], a TF from the HTH family, could boost the stress resistance during fermentation. Remarkably, Spt15 is also the first priority suggested by our GWAS as potential engineering target (**Dataset S6**), thus we believe that the potential engineering targets and engineering strategies suggested by our study are of high confidence. Besides the previously revealed mechanisms and strategies (overexpression of TFs and transporters, mutagenesis of TFs, chromosomal duplications in industrial strains), the potential genotype resources and engineering targets associated with different stress conditions such as SNPs/InDels and loss events of environmental strains, the TFs and significant genes which have not been previously engineered,

uncharacterized gene clusters, tandem repeats, are new outcomes arising from this study. Therefore, we propose here several sources to select potential engineering targets as indicated below:

1. *The extreme genotypes found in the well-performing strains*.

   For instance, the suggested platform strain Y55 has below average resistance to 4-aminobenzoic acid and ferulic acid. To improve the resistance to these and related compounds, genomic features from AL1, CLIB215 and GDB325, the most resistant strains to the selected conditions, could be introduced. Large-scale duplications of chrXII (3~4 copies from CLIB215), chrVI (6 copies from CLIB215), and the repeat region of chrVI:742212-745751 (82 copies from AL1), could be engineered to Y55.

2. *The most significant variants and genes suggested by GWAS*.

   For example, for the resistance to ferulic acid, the LD blocks chrXIV: 605937-607882 (–lg (*P* value) = 11.76) and chrIX:151566-161597 (–lg (*P* value) = 11.46) were identified as the most significant markers for Robustness and Performance respectively. The SNPs/InDels in these blocks and the overlapped genes could be considered as the first priority.

3. *The variants or genes by multiple GWAS profiles regarding resistance to different conditions*.

   The CNV region YHR218W-YHR219 (with both gain and loss events from different rankings) was found significantly associated with seven different stress conditions. The gain event of YNR059W-YNR062C was associated with five acidic conditions. The putative gene YER138W-A with unknown function was shared by six acidic conditions, while its neighbor YER148W/*SPT15* (also significant in multiple conditions) has been previously reported to be highly associated with ethanol resistance [8]. *SKY1* was also a significant gene shared by 4 conditions, which has been proved to be associated with osmotic tolerance [27].

4. *Specific gene sets that were captured in multi-dimension studies*.

Three transcription factors, Spt15 (HTH) (previously reported [8]), Ecm22 (ZNF), Bur6 (Other TF) were identified by GWAS, among which, Ecm22 and Bur6 were from the CNVs while Spt15 (HTH) was from the SNPs. The gain event of YNR059W-YNR062C was associated with the Robustness against multiple acidic conditions. These cases from GWAS can also match the analyses in the genotyping.

5. *The modules discovered in the phenotype associated PPIN*.

The *COX*/*ATP*/Mitochondrial Gene module is part of the respiratory chain and the genes were found to provide protection against acetic acid and other stresses [28], due to its association to respiration, redox and ion balance, which could serve as promising engineering targets for the ethanol and acid resistance. The *FRE*/*FIT* module is associated with the iron transport in cell wall, whereas it is also reported that the tolerance to inorganic acid and weak acid is correlated with iron uptake [29, 30]. The *PAU* module (the seripauperin gene family) which encodes yeast cell wall mannoproteins [31], was proved to be responsible for anaerobiosis and environmental stress [32]. As the gain event of uncharacterized genes was discovered to be associated to the acid resistance, the modules with uncharacterized and dubious genes (*YRF*/Uncharacterized module, *YPR* and *YHR* modules for ethanol resistance, *SOR*/*HKX* module for thermotolerance, etc.) could also be used to characterize the new functions and the association with stress resistance. Other network-specific modules, especially the ones with multiple uncharacterized genes, could also be considered as engineering targets. For instance, the *PYC/LPD/KGD/CIT/ACS/GDH* module has genes from Krebs cycle, the *GLK/SOR/HXK* module participates the phosphorylation, and the *SCH/YPK* module has genes from the Fermentable Growth Medium (FGM) signalling pathways. These genes from carbon metabolism were verified to be associated with the resistance to alcohols and acids [28].

## POTENTIAL IMMPLICATIONS

The present work has not only revealed successfully the phenotypic and genotypic divergence of a representative strain collection and discussed the underlying evolutionary mechanisms, but also proposed a practical toolbox for platform strain selection and identification of new engineering targets, a consistent number of which were already presented here. Technically, the innovative methods used in this study, including the comprehensive resistance score calculation, the strain-specific GSMM and fluxome construction, the analytically rigorous core-genome and CNV-based GWAS, and the noise-reducing PPIN module discovery, are also applicable to other geno- and phenotyping projects, especially the strain level population studies with high inter-strain genetic diversity.

## METHODS

### Yeast strain collection storage

A collection of 36 *S. cerevisiae* strains were studied in this research, and the details were summarized in **Supplemental Note S1**. The strains were stored at -80°C in cryogenic tubes containing YPD medium with 20% (vol/vol) glycerol. From the stock tubes, a sterile inoculation loop was used to transfer cells onto YPD plates. Plates were incubated for 48 h before cells were used for pre-cultivation.

### Medium for pre-cultures and under various stress conditions

Pre-cultures of yeast strains were grown in a defined mineral medium containing 7.5 g/L $(NH_4)_2SO_4$, 14.4 g/L $KH_2PO_4$, 0.5 g/L $MgSO_4{\bullet}7H_2O$, 2 mL/L of trace element solution, 1 mL/L vitamin solution (prepared according to Verduyn *et al.* [33]) and 20 g/L glucose. The pH of the salts together with trace elements was adjusted to 6.0 with NaOH before autoclaving. The glucose solution was autoclaved separately before being added to the salt solution together with the filter-sterilized vitamin solution. Pre-cultures were made in 24-deepwell plates (CR1424, Enzyscreen, The Netherlands) containing 1 mL

mineral medium in each well. A single yeast colony was inoculated from an YPD agar plate and grown for 20 h at 30°C and 300 rpm in an incubator with 51 mm shaking orbit. Media for cultivation under various stress conditions were introduced in detail in **Supplemental Note S2**.

**Cultivation in the Growth Profiler 1152 and data processing**

Yeast strains were pre-cultivated as described above and harvested by centrifugation then inoculated to 96-weell microplates. The inoculated plates were then placed in the Growth Profiler 1152 and growth was monitored for ca. 66 hours. Details of cultivation, biological replicates and data processing were described in detail in **Supplemental Note S2**.

**Investigation of carbon and nitrogen source utilization with Biolog Phenotype Microarrays**

Yeast strains CEN.PK113-7D, S288C and Ethanol Red were pre-grown in 50 mL conical tubes using 5 mL YPD medium at 30°C and 280 rpm for 16 h. These pre-cultures were used to inoculate 250 mL shake flasks with 25 mL YPD medium at an initial OD of 0.2. The strains were cultivated until the OD reached ~1 (ca. 5 h) at which point the cells were washed twice in sterile water. After the final wash the cells were concentrated to an OD of 4.2 by diluting with an appropriate volume of sterile water. The cell suspensions were diluted 48-fold when added to the media specific for PM1-3, resulting in a starting OD of 0.0875. After inoculation, the PM plates were placed in the OmniLog incubator at 30°C and the development of the colored dye was measured every 15 min for 83 h. Data files were converted and exported to Excel using the dedicated software from the supplier.

**Intra- and extracellular metabolome screening**

Intracellular and extracellular metabolites of the yeast strains growing in glucose media were profiled using GC-MS. Metabolite identification and normalization of GC-MS data was performed using the AMDIS (Automated Mass Spectral Deconvolution and Identification System) software. Intra- and extracellular metabolites were assigned to different compound classes, and the intracellular metabolites

were also assigned to different pathways and pathway groups. The experimental protocol was described in detail in **Supplemental Note S3**.

**Genome sequencing and estimation of strain ploidy**

The genomes of the 36 *S. cerevisiae* strains were sequenced using the Illumina MiSeq or HiSeq 2000 platform. Paired-end sequencing libraries with 350 bp insert size were prepared with the TruSeq Nano DNA kit and sequenced with either 150 nt or 250 nt read length. Data quality control and filtering were performed by FastQC. Strain ploidy was determined by relative comparison of the DNA amount of the $G_0$-$G_1$ gated population of the target strains with reference *S. cerevisiae* strains of known ploidy, measured by flow cytometry following the procedure previously described [22]. To avoid the misidentification of aneuploid strains as polyploid strain in the flow cytometry analysis, the ploidy estimation results were verified by the allele frequencies of the heterozygous ~~base modifications~~SNPs/InDels.

**Reads mapping, variant calling and annotation**

Reads were mapped to the S288c reference genome (SGD release 64 [17]) using BWA (v0.7.12, module *mem*) [34]. A minimum coverage of 50x, after filtration, was set as requirement for each strain. ~~Base modifications~~SNPs/InDels ~~, including SNPs and small InDels,~~ were called and filtered using the Genome Analysis Toolkit (GATK) 3.4 [35, 36], with the sequential steps to include RealignerTargetCreator, IndelRealigner, HaplotypeCaller (with parameter “-rf BadCigar”) ~~UnifiedGenotyper~~ and VariantFiltration (with parameter “--filterExpression "DP < 10 || QD < 2.0 || FS > 60.0"”)~~, with the parameters previously described [20]~~. SNPs and InDels were annotated by SnpEff using the *S. cerevisiae* database version EF4.69 [37]. The 800 bp upstream regions of the genes were included as potential regulatory sequences. CNVs were detected using Control-FreeC [38], whereas the reads of the S288c haploid strain were used as the reference genome. Gene fully covered by the CNV regions were labeled

as affected genes. If a gene was partially overlapped with a CNV region, gain event would not be assigned due to the incompleteness of the obtained copies, while loss or deletion event (when the copy-number of the CNV region is zero) will be assigned to this gene, as at least one copy of the gene was truncated.

**Population structure analysis**

The consensus sequences of the 36 strains were generated using GATK [36] based on the SNP set. Protein sequences were translated from the ORFs and used for the Neighbor-Joining tree building by TreeBest [39].

Formatted: English (United States)

Formatted: English (United States)

***De novo* assembly and ORF prediction**

*De novo* assemblies were performed with Newbler version 2.8 using the default parameters. To identify the potential novel genes in the yeast population, compared to the reference genome S288c, ORF predictions were performed with YGAP [40] based on the *de novo* assemblies. For the genes of which no S288c homologous gene was annotated in YGAP, we further extracted the sequences and searched against NCBI nr protein data set [41] using BLASTX [42]. The ORFs with at least one valid hit to S288c (identity $\geq$ 95% and *E* value < 1e-5) were removed from the potential novel gene list and treated as miss-identifications of the YGAP pipeline.

**Strain-specific Genome-scale Metabolic Model (GSMM) construction**

Strain-specific GSMMs were constructed from the starting model iMM904 [43]. Severe mutations of consequences of gene truncation, elongation or deletion were considered in different severity levels, as well as the new reactions introduced by non-S288c genes (**Supplemental Note S4**). Reactions were annotated by the UniProt database [44] and MetaNetX database [45]. The mixed integer linear programming (MILP) algorithm was applied to build the strain-specific models (**Supplemental Note S4**). Carbon and nitrogen source utilization and fluxes were simulated with the FRAMED package using Gurobi 6.5 (**Supplemental Note S4**).

**Genome-wide association studies (GWAS) for resistance rankings**

GWAS were carried out for ~~base modifications~~SNPs/InDels ~~(SNPs/InDels)~~ and CNVs, respectively (as called SNP-based and CNV-based GWAS). Only SNPs/InDels in core-genome regions and with MAF > ~~0.0~~5% were used in the SNP-based GWAS. CNV markers were defined due to the overlap relationships and were used for gain and loss event separately. Strain rankings were used as phenotypic values. The mixed-model based method Efficient Mixed Model Association (EMMA [46]) was applied as the main algorithm in GWAS. Details of the core-GWAS markers, CNV marker identification, transformation of genotypic values, linkage disequilibrium (LD) block identification and *P* value assignment for genes were described in detail in **Supplemental Note S5**. Different significance cut-offs for SNPs/gain/loss markers were set according to the departure of observed *P* value from the predicted *P* value distribution (**Supplemental Note S5**).

**Gene categories used in genotyping and GWAS**

The basic gene information was acquired from SGD [17], including gene ID, symbol name, the ORF type (verified, uncharacterized and dubious), EC number. The transcription factor list, with the super family classification, and the regulatory relationships, were collected from SGD [17] and YEASTRACT [47]. The GO-Slim Terms were acquired from SGD [17]. The metabolic pathways (YeastCyc) were downloaded from SGD [17] and MetaCyc [48]. The phenotype-associated gene lists were grasped from the Yeast Phenotype Ontology from SGD [17]. Among the gene and phenotype association entries, only the entries with positive or negative effects to phenotype were reserved, and the ones with neutral or unclear consequence were removed. When applied with GWAS profiles, the SGD entries were re-classified according to the phenotype classification and used chemicals (**Supplemental Note S5**).

**The discovery of the PPI modules from SGD gene list and GWAS profile**

The PPI network of *S. cerevisiae* was acquired from the STRING [49] database and regulatory modules were computed by ModuleDiscoverer [18] for both SGD gene lists and GWAS profiles (see details in **Supplemental Note S5**).

**Statistical analysis**

All statistical analyses were performed in R. For comparative analysis between industrial and environmental strains, Wilcoxon rank-sum tests were performed. For comparative analysis among different strain subcategorises, Kruskal-Wallis tests were performed. For multiple comparisons, Benjamini–Hochberg procedure was used to calculate the False Discovery Rate (FDR), *FDR* < 0.05 or *FDR* < 0.1 was used as the significance cut-off.

Spearman's tests with Bonferroni adjustment were performed to correlate the genotypic or phenotypic features, where *FDR* < 0.05 was used as significance cut-off.

The mixed-model based method Efficient Mixed Model Association (EMMA) [46] was applied as the main algorithm in GWAS.

**Data visualization**

R [50] and corresponding packages including ggplot2, ggtree, heatmap.plus and matplotlib were used for illustration of statistical results. Cytoscape 3.6.0 [51] was used to visualize the analyses incorporating network and topology.

**Availability of data**

The whole-genome sequence data have been deposited in the NCBI Sequence Read Archive (SRA; http://www.ncbi.nlm.nih.gov/sra) under accession number SRR6114130 to SRR6114127.

The metabolomic data have been deposited in EBI MetaboLights with identifier MTBLS780.

**DECLARATIONS**

**List of abbreviations**

ABE: Acetone, n-Butanol, Ethanol

AMDIS: Automated Mass Spectral Deconvolution and Identification System

~~BM: base modification~~

CI: confidence interval

CNV: copy number variation

Core-GWAS: SNP-based core genome GWAS

EC: Enzyme Commission

EMMA: Efficient Mixed Model Association

FDR: false discovery rate

FGM: fermentable growth medium

GC-MS: Gas Chromatography-Mass Spectrometer

GO: Gene Ontology

GPR: gene-protein-reaction

GSMM: Genome-scale metabolic models

GWAS: Genome-wide association studies

HTH: Helix-turn-helix

InDel: insertion and deletion

KEGG: Kyoto Encyclopedia of Genes and Genomes

LD: linkage disequilibrium

MAF: minor allele frequency

MCF: methyl chloroformate

MILP: mixed integer linear programming algorithm

NCBI: National Center of Biotechnology Information

OD: optical density

ORF: open reading frame

*pdf*: probability density function

PI: Parameter Influence

PIA: Parameter Influence Analysis

PPIN: protein-protein interaction networks

RVA: Rank Variability Analysis

*S. cerevisiae*: *Saccharomyces cerevisiae*

SD: standard deviation

SGD: *Saccharomyces* Genome Database

SLM: Shape Language Model

SNP: single nucleotide polymorphism

SRA: Sequence Read Archive

TCA cycle: tricarboxylic acid cycle

TF: transcription factor

YGAP: Yeast Genome Annotation Pipeline

ZIP: Zipper

ZNF: Zinc finger

**Competing interests**

The authors declare no competing interests.

**Funding**

GP would like to thank Deutsche Forschungsgemeinschaft (DFG) CRC/Transregio 124 'Pathogenic fungi and their human host: Networks of interaction', subproject B5. BB, LD, MJH and JF thank the Novo Nordisk Foundation for financial support.

**Authors' contributions**

BB designed the physiological characterization. BB and LD performed the experiments. KK performed the data analyses. BB, KK and DM wrote the initial manuscript. GP, JF and MJH supervised this study. DM performed the GSMM construction and simulation. HM and SVB performed the metabolomic measurements. JL advised the bioinformatics analyses. All authors conceived the project and approved the final version of manuscript.

**Acknowledgements**

The authors would like to thank Dr. Sebastian Vlaic from HKI, Germany for the development of ModuleDiscoverer and the assistance in PPIN analysis.

## SUPPLEMENTAL ITEMS

This article has 13 supplemental items, including the supplemental notes (including 5 sections), 2 tables, 4 figures and 6 datasets in individual documents.

**Supplemental Dataset Captions**

**Dataset S1**. The physiological characterization results.

**Dataset S2**. The intra- and extracellular metabolomes.

**Dataset S3**. The genetic makeups of the strains, including copy number variation profiles, ~~base modification~~SNP/InDel profiles and none-S288c genes predicted by YGAP.

**Dataset S4**. The strain-specific Genome-scale Metabolic Models (in .xml files).

**Dataset S5**. The growth data for CEN.PK, Ethanol Red and S288c in the utilization of different carbon and nitrogen sources.

**Dataset S6**. The genes significantly associated with Robustness and Performance rankings discovered by GWAS.

## REFERENCES


1. de Jong E, Higson A, Walsh P and Wellisch M. Bio-based chemicals value added products from biorefineries. IEA Bioenergy, Task42 Biorefinery. 2012.
2. Taylor R, Nattrass L, Alberts G, Robson P, Chudziak C, Bauen A, et al. From the sugar platform to biofuels and biochemicals. Final Report for the European Commission Directorate-General Energy N (ENER/C2/423-2012/SI2 673791). 2015.
3. Archer CT, Kim JF, Jeong H, Park JH, Vickers CE, Lee SY, et al. The genome sequence of E. coli W (ATCC 9637): comparative genome analysis and an improved genome-scale reconstruction of E. coli. BMC genomics. 2011;12:9. doi:10.1186/1471-2164-12-9.
4. van Dijken JP, Bauer J, Brambilla L, Duboc P, Francois JM, Gancedo C, et al. An interlaboratory comparison of physiological and genetic properties of four Saccharomyces cerevisiae strains. Enzyme Microb Technol. 2000;26 9-10:706-14.
5. Cakar ZP, Turanli-Yildiz B, Alkim C and Yilmaz U. Evolutionary engineering of Saccharomyces cerevisiae for improved industrially important properties. FEMS yeast research. 2012;12 2:171-82. doi:10.1111/j.1567-1364.2011.00775.x.
6. Li BZ and Yuan YJ. Transcriptome shifts in response to furfural and acetic acid in Saccharomyces cerevisiae. Applied microbiology and biotechnology. 2010;86 6:1915-24. doi:10.1007/s00253-010-2518-2.
7. Castle LA, Siehl DL, Gorton R, Patten PA, Chen YH, Bertain S, et al. Discovery and directed evolution of a glyphosate tolerance gene. Science. 2004;304 5674:1151-4. doi:10.1126/science.1096770.
8. Alper H, Moxley J, Nevoigt E, Fink GR and Stephanopoulos G. Engineering yeast transcription machinery for improved ethanol tolerance and production. Science. 2006;314 5805:1565-8. doi:10.1126/science.1131969.
9. Houghton-Larsen J and Brandt A. Fermentation of high concentrations of maltose by Saccharomyces cerevisiae is limited by the COMPASS methylation complex. Appl Environ Microbiol. 2006;72 11:7176-82. doi:10.1128/AEM.01704-06.
10. Upchurch RG. Fatty acid unsaturation, mobilization, and regulation in the response of plants to stress. Biotechnol Lett. 2008;30 6:967-77. doi:10.1007/s10529-008-9639-z.
11. Skelly DA, Merrihew GE, Riffle M, Connelly CF, Kerr EO, Johansson M, et al. Integrative phenomics reveals insight into the structure of phenotypic diversity in budding yeast. Genome research. 2013;23 9:1496-504. doi:10.1101/gr.155762.113.
12. Strope PK, Skelly DA, Kozmin SG, Mahadevan G, Stone EA, Magwene PM, et al. The 100-genomes strains, an S. cerevisiae resource that illuminates its natural phenotypic and genotypic variation and emergence as an opportunistic pathogen. Genome research. 2015;25 5:762-74. doi:10.1101/gr.185538.114.
13. Liti G, Carter DM, Moses AM, Warringer J, Parts L, James SA, et al. Population genomics of domestic and wild yeasts. Nature. 2009;458 7236:337-41. doi:10.1038/nature07743.
14. da Silva-Filho EA, Brito dos Santos SK, Resende Ado M, de Morais JO, de Morais MA, Jr. and Ardaillon Simoes D. Yeast population dynamics of industrial fuel-ethanol fermentation process assessed by PCR-fingerprinting. Antonie Van Leeuwenhoek. 2005;88 1:13-23. doi:10.1007/s10482-004-7283-8.

15. Peter J, De Chiara M, Friedrich A, Yue JX, Pflieger D, Bergstrom A, et al. Genome evolution across 1,011 Saccharomyces cerevisiae isolates. Nature. 2018;556 7701:339-44. doi:10.1038/s41586-018-0030-5.
16. Monk JM, Koza A, Campodonico MA, Machado D, Seoane JM, Palsson BO, et al. Multi-omics Quantification of Species Variation of Escherichia coli Links Molecular Features with Strain Phenotypes. Cell Syst. 2016;3 3:238-51 e12. doi:10.1016/j.cels.2016.08.013.
17. Cherry JM, Hong EL, Amundsen C, Balakrishnan R, Binkley G, Chan ET, et al. Saccharomyces Genome Database: the genomics resource of budding yeast. Nucleic acids research. 2012;40 Database issue:D700-5. doi:10.1093/nar/gkr1029.
18. Vlaic S, Conrad T, Tokarski-Schnelle C, Gustafsson M, Dahmen U, Guthke R, et al. ModuleDiscoverer: Identification of regulatory modules in protein-protein interaction networks. Sci Rep. 2018;8 1:433. doi:10.1038/s41598-017-18370-2.
19. Breunig JS, Hackett SR, Rabinowitz JD and Kruglyak L. Genetic basis of metabolome variation in yeast. PLoS Genet. 2014;10 3:e1004142. doi:10.1371/journal.pgen.1004142.
20. Borneman AR, Desany BA, Riches D, Affourtit JP, Forgan AH, Pretorius IS, et al. Whole-Genome Comparison Reveals Novel Genetic Elements That Characterize the Genome of Industrial Strains of Saccharomyces cerevisiae. PLoS Genetics. 2011;7 2:e1001287. doi:10.1371/journal.pgen.1001287.
21. Kang K, Li J, Lim BL and Panagiotou G. MESSI: metabolic engineering target selection and best strain identification tool. Database : the journal of biological databases and curation. 2015;2015 doi:10.1093/database/bav076.
22. Stovicek V, Borodina I and Forster J. CRISPR–Cas system enables fast and simple genome editing of industrial Saccharomyces cerevisiae strains. Metabolic Engineering Communications. 2015;2:13-22.
23. Zhang GC, Kong, II, Kim H, Liu JJ, Cate JH and Jin YS. Construction of a quadruple auxotrophic mutant of an industrial polyploid saccharomyces cerevisiae strain by using RNA-guided Cas9 nuclease. Appl Environ Microbiol. 2014;80 24:7694-701. doi:10.1128/AEM.02310-14.
24. Bergstrom A, Simpson JT, Salinas F, Barre B, Parts L, Zia A, et al. A high-definition view of functional genetic variation from natural yeast genomes. Mol Biol Evol. 2014;31 4:872-88. doi:10.1093/molbev/msu037.
25. Caspeta L, Chen Y, Ghiaci P, Feizi A, Buskov S, Hallstrom BM, et al. Biofuels. Altered sterol composition renders yeast thermotolerant. Science. 2014;346 6205:75-8. doi:10.1126/science.1258137.
26. Alriksson B, Horváth IS and Jönsson LJ. Overexpression of Saccharomyces cerevisiae transcription factor and multidrug resistance genes conveys enhanced resistance to lignocellulose-derived fermentation inhibitors. Process Biochemistry. 2010;45 2:264-71.
27. Yoshikawa K, Tanaka T, Furusawa C, Nagahisa K, Hirasawa T and Shimizu H. Comprehensive phenotypic analysis for identification of genes affecting growth under ethanol stress in Saccharomyces cerevisiae. FEMS Yeast Res. 2009;9 1:32-44. doi:10.1111/j.1567-1364.2008.00456.x.
28. Henriques SF, Mira NP and Sa-Correia I. Genome-wide search for candidate genes for yeast robustness improvement against formic acid reveals novel susceptibility (Trk1 and positive regulators) and resistance (Haa1-regulon) determinants. Biotechnology for biofuels. 2017;10:96. doi:10.1186/s13068-017-0781-5.

29. Mira NP, Teixeira MC and Sa-Correia I. Adaptive response and tolerance to weak acids in Saccharomyces cerevisiae: a genome-wide view. OMICS. 2010;14 5:525-40. doi:10.1089/omi.2010.0072.
30. Abbott DA, Suir E, van Maris AJ and Pronk JT. Physiological and transcriptional responses to high concentrations of lactic acid in anaerobic chemostat cultures of Saccharomyces cerevisiae. Appl Environ Microbiol. 2008;74 18:5759-68. doi:10.1128/AEM.01030-08.
31. Marguet D, Guo XJ and Lauquin GJ. Yeast gene SRP1 (serine-rich protein). Intragenic repeat structure and identification of a family of SRP1-related DNA sequences. Journal of molecular biology. 1988;202 3:455-70.
32. Rivero D, Berna L, Stefanini I, Baruffini E, Bergerat A, Csikasz-Nagy A, et al. Hsp12p and PAU genes are involved in ecological interactions between natural yeast strains. Environ Microbiol. 2015;17 8:3069-81. doi:10.1111/1462-2920.12950.
33. Verduyn C, Postma E, Scheffers WA and Van Dijken JP. Effect of benzoic acid on metabolic fluxes in yeasts: a continuous-culture study on the regulation of respiration and alcoholic fermentation. Yeast. 1992;8 7:501-17. doi:10.1002/yea.320080703.
34. Li H and Durbin R. Fast and accurate long-read alignment with Burrows-Wheeler transform. Bioinformatics. 2010;26 5:589-95. doi:10.1093/bioinformatics/btp698.
35. DePristo MA, Banks E, Poplin R, Garimella KV, Maguire JR, Hartl C, et al. A framework for variation discovery and genotyping using next-generation DNA sequencing data. Nature genetics. 2011;43 5:491-8. doi:10.1038/ng.806.
36. McKenna A, Hanna M, Banks E, Sivachenko A, Cibulskis K, Kernytsky A, et al. The Genome Analysis Toolkit: a MapReduce framework for analyzing next-generation DNA sequencing data. Genome research. 2010;20 9:1297-303. doi:10.1101/gr.107524.110.
37. Cingolani P, Platts A, Wang le L, Coon M, Nguyen T, Wang L, et al. A program for annotating and predicting the effects of single nucleotide polymorphisms, SnpEff: SNPs in the genome of Drosophila melanogaster strain w1118; iso-2; iso-3. Fly. 2012;6 2:80-92. doi:10.4161/fly.19695.
38. Boeva V, Popova T, Bleakley K, Chiche P, Cappo J, Schleiermacher G, et al. Control-FREEC: a tool for assessing copy number and allelic content using next-generation sequencing data. Bioinformatics. 2012;28 3:423-5. doi:10.1093/bioinformatics/btr670.
39. Vilella AJ, Severin J, Ureta-Vidal A, Heng L, Durbin R and Birney E. EnsemblCompara GeneTrees: Complete, duplication-aware phylogenetic trees in vertebrates. Genome research. 2009;19 2:327-35. doi:10.1101/gr.073585.107.
40. Proux-Wera E, Armisen D, Byrne KP and Wolfe KH. A pipeline for automated annotation of yeast genome sequences by a conserved-synteny approach. BMC bioinformatics. 2012;13:237. doi:10.1186/1471-2105-13-237.
41. Pruitt KD, Tatusova T and Maglott DR. NCBI reference sequences (RefSeq): a curated non-redundant sequence database of genomes, transcripts and proteins. Nucleic acids research. 2007;35 Database issue:D61-5. doi:10.1093/nar/gkl842.
42. Gish W and States DJ. Identification of protein coding regions by database similarity search. Nature genetics. 1993;3 3:266-72. doi:10.1038/ng0393-266.
43. Mo ML, Palsson BO and Herrgard MJ. Connecting extracellular metabolomic measurements to intracellular flux states in yeast. Bmc Syst Biol. 2009;3:37. doi:10.1186/1752-0509-3-37.
44. UniProt C. The Universal Protein Resource (UniProt) in 2010. Nucleic acids research. 2010;38 Database issue:D142-8. doi:10.1093/nar/gkp846.

45. Moretti S, Martin O, Van Du Tran T, Bridge A, Morgat A and Pagni M. MetaNetX/MNXref--reconciliation of metabolites and biochemical reactions to bring together genome-scale metabolic networks. Nucleic acids research. 2016;44 D1:D523-6. doi:10.1093/nar/gkv1117.
46. Kang HM, Zaitlen NA, Wade CM, Kirby A, Heckerman D, Daly MJ, et al. Efficient control of population structure in model organism association mapping. Genetics. 2008;178 3:1709-23. doi:10.1534/genetics.107.080101.
47. Teixeira MC, Monteiro P, Jain P, Tenreiro S, Fernandes AR, Mira NP, et al. The YEASTRACT database: a tool for the analysis of transcription regulatory associations in Saccharomyces cerevisiae. Nucleic acids research. 2006;34 Database issue:D446-51. doi:10.1093/nar/gkj013.
48. Caspi R, Billington R, Ferrer L, Foerster H, Fulcher CA, Keseler IM, et al. The MetaCyc database of metabolic pathways and enzymes and the BioCyc collection of pathway/genome databases. Nucleic acids research. 2016;44 D1:D471-80. doi:10.1093/nar/gkv1164.
49. Jensen LJ, Kuhn M, Stark M, Chaffron S, Creevey C, Muller J, et al. STRING 8--a global view on proteins and their functional interactions in 630 organisms. Nucleic acids research. 2009;37 Database issue:D412-6. doi:10.1093/nar/gkn760.
50. R Core Team. R: A Language and Environment for Statistical. Vienna, Austria: R Foundation for Statistical Computing, 2015.
51. Shannon P, Markiel A, Ozier O, Baliga NS, Wang JT, Ramage D, et al. Cytoscape: a software environment for integrated models of biomolecular interaction networks. Genome research. 2003;13 11:2498-504. doi:10.1101/gr.1239303.

## FIGURE LEGENDS

**Figure 1. The stress conditions and resistance scores**. (**A**) The thirteen stress conditions and four-to-seven inhibitory levels in each condition. The colored cells indicate the number of strains that could grow in each experimental setting. (**B**) Calculation of the resistance scores (Performance and Robustness) based on five growth parameters. (**C**) The inhibitory level-specific Performance and Robustness scores of three strains under 1,4-butanediol condition: Y55, high ranking in both scores; DBVPG1373: good Robustness but poor Performance; PW5: good Performance but bad Robustness.

**Figure 2. The physiological characterization of various *S. cerevisiae* strains**. (**A**) The Performance and Robustness ranking values in all conditions from selected cases with different patterns. (**B**) The Performance and Robustness rankings for all strains in all conditions. Strains were sorted by their frequencies to be ranked in top 10 in all rankings. (**C**) The comparisons of different strain categories and subcategories in multiple resistance rankings. In the industrial vs. environmental comparisons, star marker suggests a significant difference (Wilcoxon rank-sum test, $FDR < 0.1$).

**Figure 3. The metabolomic profiles of various isolated strains of *Saccharomyces cerevisiae*.** (**A**) The metabolome of the yeast strains growing exponentially on glucose, and the consumed/excreted metabolites from the spent medium. The colors represent the combined contribution of metabolites in a pathway or compound class **(Supplemental Note S3)**. For intracellular metabolites, a red color indicates that the group contains a majority of metabolites that are higher above the mean (and *vice versa* for blue). For extracellular metabolites, red indicates a high production level and blue indicates a high degree of consumption. Numbers in parenthesis indicate total number of metabolites in the group. Hierarchical

clustering was applied to the group variables using Euclidian distance between the strains. For data normalization before visualization, see details in **Supplemental Note S3**. (**B**) The comparisons of the rankings of metabolome groups, compound classes or metabolic pathways among different strain categories and subcategories. (**C**) The comparisons of the key fluxes and the fluxes in metabolic subsystems between industrial and environmental strains. PYRt2m: Pyruvate Mitochondrial Transport via Proton Symport, PDHm: Pyruvate Dehydrogenase. In the industrial vs. environmental comparisons, star marker suggests a significant difference (Wilcoxon rank-sum test, $FDR < 0.1$).

**Figure 4. The genetic characterization of various *Saccharomyces cerevisiae* isolated strains.** (**A**) A phylogenetic tree of all the strains was constructed based on identified ~~base modifications~~SNPs/InDels ~~(SNPs/InDels)~~ and shows three main clades. The strain functional class, geographical origin and determined ploidy (for non-diploid strains) were marked. The genetic features were normalized to *Z* scores and illustrated in heat map. (**B-C**) The comparisons between the industrial and environmental strains on the number of variants **(B)** and the ratio of variants in specific gene sets (for SNPs/InDels) or the number of non-S288c genes and genes affected by CNVs) **(C)**. Star marker suggests a significant difference (Wilcoxon rank-sum test, $P < 0.05$). (**D**) The comparisons among different strain subcategories on the number of variants, SNP ratio in different gene sets, number of non-S288c genes and gene affected by CNVs.

**Figure 5. The genome-wide associations for the resistance rankings and the genomic hotspots**. (**A**) Genome-wide distributions of significant variants, for LDs or non-LD SNPs/InDels and CNVs respectively. (**B**) The total number of significant LDs and non-LD SNPs/InDels in each 50k bp genomic windows. (**C**) Genome-wide significance levels for CNV regions. Condition labels were colored by

acidic or non-acidic condition classification. A region was colored in red if its gain event was more significant than the loss event, or *vice versa*.

**Figure 6. The relative contributions from different variant source (SNP or CNV, gain or loss event) to different inhibitory conditions and scores**. The coordinates were calculated following the protocol in **Supplemental Note S5**. The first sub figure shows the relative contributions of the 75% quantile of all genes (sorted by *P* values). The other sub figures show the relative contributions in specific gene categories (verified or uncharacterized ORFs, TFs and different TF super families).

**Figure 7. The protein-protein interaction networks of multiple phenotypes, with SGD recorded entries and GWAS observations.** The node color indicates if a gene is from the SGD entry, GWAS profile or shared by both. The node border color and shape denote different gene categories. The eight GWAS-specific modules (not identified from the network built from the SGD gene list) that were shared by multiple PPI networks are highlighted in different background colors. Network-specific GWAS modules are shown in white background.

Revised Manuscript
Click here to access/download;Manuscript;SC_Main.docx
Click here to view linked References

# Linking genetic, metabolic and phenotypic diversity among *S. cerevisiae* strains using multi-omics associations

Kang Kang[1,7†], Basti Bergdahl[2†], Daniel Machado[3,4], Laura Dato[2], Ting-Li Han[5], Jun Li[1,7], Silas Villas-Boas[5], Markus J. Herrgård[2*], Jochen Förster[2*,#] and Gianni Panagiotou[1,6,7*]

† Authors contributed equally to this work

* Corresponding author: herrgard@biosustain.dtu.dk , Jochen.Forster@carlsberg.com , Gianni.Panagiotou@hki-jena.de

Author affiliations:

1. Systems Biology & Bioinformatics Group, School of Biological Sciences, The University of Hong Kong, Hong Kong S.A.R., China
2. The Novo Nordisk Foundation Center for Biosustainability, Technical University of Denmark, Kgs.Lyngby, Denmark
3. Department of Biological Engineering, School of Engineering, University of Minho, Braga, Portugal
4. The European Molecular Biology Laboratory (EMBL), Heidelberg, Germany
5. Centre for Microbial Innovation, School of Biological Sciences, University of Auckland, Auckland, New Zealand
6. Department of Microbiology, Li Ka Shing Faculty of Medicine, The University of Hong Kong, Hong Kong S.A.R., China

7. Systems Biology & Bioinformatics Unit, Leibniz Institute for Natural Product Research and Infection Biology – Hans Knöll Institute, Jena, Germany

# Present address: Carlsberg A/S, Carlsberg Research Laboratory, 1799, Copenhagen V, Denmark

* Correspondence:

Gianni Panagiotou, Systems Biology & Bioinformatics Unit, Leibniz Institute for Natural Product Research and Infection Biology, Hans Knöll Institute, Jena, Germany. Email: gianni.panagiotou@hki-jena.de. Address: Adolf-Reichwein-Straße 23, 07745 Jena, Germany. Phone: +49 3641 532-1759

## ABSTRACT

The selection of bioengineering platform strains and engineering strategies to improve the stress resistance of *Saccharomyces cerevisiae* remains a pressing need in bio-based chemical production. Thus, a systematic effort to exploit the genotypic and phenotypic diversity to boost yeast's industrial value is still urgently needed. Here, we analyzed 5400 growth curves obtained from 36 *S. cerevisiae* strains and comprehensively profiled their resistances against 13 industrially relevant stresses. We observed that bioethanol and brewing strains exhibit higher resistance against acidic conditions, however, plant isolates tend to have wider range of resistance, which may be associated with their metabolome and fluxome signatures in TCA cycle and fatty acid metabolism. By deep genomic sequencing we found that industrial strains have more genomic duplications especially affecting transcription factors, presenting disparate evolutionary paths in comparison to the environmental strains which have more InDels, gene deletions and strain-specific genes. Genome-wide association studies coupled with protein-protein interaction networks uncovered novel genetic determinants of stress resistances. These resistance-related engineering targets and strain rankings provide a valuable source for engineering significantly improved industrial platform strains.



## KEYWORDS

*S. cerevisiae*, Multi-omic study, Platform strain, Stress resistance, Geno-to-phenotype association

## BACKGROUND

We are facing a paradigm shift, where our economy and industrial processes need to exchange current oil-based technologies with new and sustainable biotechnologies. The transition to a bio-based economy has already begun as several products have reached commercial production scale, e.g. cellulosic ethanol, succinic acid, lactic acid, acetic acid, itaconic acid, 1,3-propanediol, 1,4-butanediol, farnesene and ABE (Acetone, n-Butanol, Ethanol) [1, 2]. In order to build a microbial cell factory to convert sugars or other carbon sources into bio-products, the first step is to select a host organism with metabolic and physiological properties suitable for the intended bioprocess. These properties include tolerance to substrates, products and byproducts in high concentration, and resistance to the abiotic stresses including low/high pH, high temperature, osmotic stress etc. during the industrial fermentation.

Most current metabolic engineering projects are carried out using laboratory strains of bacteria [3] or yeast [4] as the starting host. Although such strains are easily manipulated genetically, they do not always meet the requirements set by stressful industrial fermentation conditions [5]. In order to improve the stress resistance of the host organism, different strategies have been adopted: (1) Comparative transcriptomic or proteomic studies [6]; (2) Directed evolution of the host genome under extreme conditions [7]; (3) Knowledge-based engineering [8]. Nevertheless, all these methods are neglecting the abundant genetic diversity present in environmental or industrial strains by focusing on narrow genetic resources (limited gene candidates, random mutations, and simple genetic changes) in a single host laboratory strain. Global screening incorporating genetic variation present in a broader strain collection could significantly speed up development of improved industrial production strains.

Here, we aim to address the scientific and engineering challenges outlined above through a *Sacharomyces cerevisiae* population multi-omics approach. We explored the diversity of 36 industrial, environmental, clinical and laboratory strains of *S. cerevisiae* regarding the genetic composition,

metabolic properties -metabolite levels and predicted flux distributions- and resistance to 13 industrially relevant stress conditions. To characterize and rank the strains' industrial values in stress resistance, we defined two scores – Robustness and Performance, calculated from five growth parameters with variable weights to meet different bioengineering purposes. The outcomes of this study include: (1) Identification of strains with multiple or specific stress-resistance as potential platform strains for cell factory construction; (2) Construction of strain-specific metabolic models explaining the divergence in metabolic phenotypes; (3) Establishing novel resistance phenotype-genotype links including suggestions of potential engineering targets to boost industrially relevant phenotypic properties.

## DATA DESCRIPTION

We performed a multi-omic study on a collection of 36 *S. cerevisiae* strains, including natural and industrial strains with different geographical origins. The genomes of these strains were sequenced with high depths (> 50x) and the variant profiles and phylogenetic tree were constructed. Besides, intra- and extracellular metabolomes were measured. Strain-specific Genome-scale Metabolic Models (GSMM) were constructed to predict the fluxomes of all the strains. Regarding phenome, the strain collection was exposed to 13 industrially relevant stresses (including low pH, high temperature and various of inhibitory compounds) in different inhibitory levels. We analyzed over 5400 growth curves to score the strains in two phenotypic scores: Robustness and Performance. Genome-wide association studies (GWAS) were performed to establish the geno-to-phenotype associations and the Protein-Protein Interaction Network (PPIN) modules associated to the stress resistance were built. Suggestions for potential engineering targets to improve the stress resistances of the bioengineering platform strain were given based on the multi-omic study.

## ANALYSES

### Systematic screening of resistance to multiple bioprocessing relevant stress conditions

In this study, we used a collection of 36 *S. cerevisiae* strains from various geographical and isolation origins (**Supplemental Note S1**). Strains were classified into four types: 17 industrial strains (from ethanol production, food and brewing industries), 13 environmental strains (isolated from soil, plant and animals), 4 laboratorial and 2 clinical strains (vaginal isolates). To investigate the phenotypic diversity in the strain collection, all strains were exposed to 13 different stress conditions including 11 inhibitory compounds, acidic pH and high temperature (**Figure 1A**). Acidic pH and the conditions using acids as inhibitory compounds were classified as acidic conditions. In each condition, the cells were exposed to the inhibitory compound at between four and seven distinct levels resulting in different numbers of strains that exhibit measurable growth (**Figure 1A & Supplemental Note S2**). In total, we analyzed more than 5400 growth curves and five growth parameters were extracted from each growth curve to score the strains in two phenotypic traits: Robustness and Performance (**Figure 1B, Supplemental Note S2 & Dataset S1**). Briefly, Robustness measures the ability of the strain to withstand increasing inhibitory levels whereas Performance measures how well a particular strain compares to other strains in a particular stress condition. The resistance scores were calculated in each inhibitory level and then integrated as final strain rankings for each condition (**Figures 2A-B & Figure S1, Supplemental Note S2**). For instance, strain DBVPG1373 had poor Performance scores under 1,4-butanediol compared to other strains but has high Robustness according to its ability to maintain its growth parameters when inhibitory levels increase; while PW5 is a counter-example with high Performance but low Robustness score due to the sharp decrease in fitness when the inhibitory levels increased (**Figure 1C**).

The strain Y55, a laboratory strain isolated from grape, was identified as the most resistant strain to multiple conditions, followed by two plant strains, RM11 and PW5 (**Figure 2A**). These strains with

plant origins could be potentially used as platform strain candidates but seldom considered in previous bioengineering endeavors [9]. Interestingly, six out of the top ten strains are industrial strains (3 bioethanol strains), but nonetheless, the industrial strains are not significantly better than environmental strains overall in multiple stress resistances. The two most commonly used laboratory strains CEN.PK113-7D and S288c, obtained low ranking values (> 6) in most conditions, with notable exceptions only the resistance to fumaric and pyruvic acids, respectively (**Figure 2B**).

Furthermore, diverse resistance patterns were observed for different strains (**Figure 2B**), which could also assist the host selection for specific production purposes: e.g., S288c and Y55 are two laboratory strains with overall bad and good universal resistance, respectively; CA1, a Brazilian bioethanol strain, shows high resistance to most acidic conditions, while the plant strain T7 is only resistant to non-acidic conditions; another bioethanol strain, CLIB215, has much higher Robustness than Performance rankings in most conditions; on the contrary, the Performance overweighs Robustness for the plant strains PW5 and RM11. When comparisons were made between industrial and environmental strains (Brazilian bioethanol strains binned as one candidate, **Supplemental Note S1**; Wilcoxon rank-sum test), the industrial strains show noticeably higher rankings in Robustness against acidic conditions ($P$ value = 6.5e-4, also significant in 4 individual rankings), while the environmental strains score better in the Performance under non-acidic conditions ($P$ value = 4.0e-2, and also significant in one individual ranking under 1,4-butanediol) (**Figure 2C**). Zooming into subcategories of the strains, bioethanol and food strains but not brewing ones have higher Robustness against acidic conditions (including low pH, formic acid and 4−aminobenzoic acid as significant individual rankings), while the Performance under non-acidic conditions was led by plant but not soil strains (Kruskal-Wallis test, $FDR < 0.1$). Different industrial strain subcategories also performed differently in divergent conditions: for instance, bioethanol

and food strains showed higher resistances to multiple conditions such as formic acid and 4−Aminobenzoic acid, while brewing strains showed high Robustness against fumaric acid.

With the objective to present the concept of how strains' physiological traits could be ranked, the above results were based on giving equal weights to the five growth parameters in the calculation of the Robustness and Performance scores. To gain further insights into the sensitivities of the rankings to weighting different parameters, Parameter Influence Analysis (PIA) was performed (**Supplemental Note S2**). A case study shows that two strains with significant growth curve differences could still have close rank positions when giving specific parameter weights (e.g., the Performance ranks of Y55 and YPS128 under 1,4-butanediol) (**Figure S2**). Such information can be useful when selecting a host strain for specific process by setting different weights to different growth parameters, e.g. lag phase would be less important selection criteria for a continuous fermentation process and subsequently be given a low weight, however, in a batch fermentation process, lag phase duration becomes critical and could be assigned with a higher weight. Besides parameter selection and weighting, our phenotypic scoring method could also be expanded to different industrially relevant processes with customized set-ups: e.g., different medium composition and aeration condition, which is not limited to the standard medium and condition used in this proof-of-concept study.

**Metabolomic profiling and correlations between metabolomics and stress resistance phenotypes**

The metabolic characteristics of all strains were investigated by determining both intra- and extracellular metabolomes by GC-MS (**Supplemental Note S3**). In both analyses, 79 metabolites were identified and quantified in terms of relative abundance (**Dataset S2**). The metabolomic data were used to group the yeast strains according to metabolite abundance in relevant pathways and/or compound classes (**Figure 3A, Dataset S2**). Overall, negative correlations could be observed between the intra- and

extracellular metabolite abundances (**Figures 3A & S3**). Significant negative correlations were captured between the extracellular abundance of proteinogenic amino acids and all intracellular compound classes except fatty acids (Spearman's correlation test with Bonferroni adjustment, $FDR < 0.05$). The analysis also showed that the industrial strains (particularly bioethanol and brewing strains) have noticeably lower extracellular metabolite levels than the environmental strains, especially for aromatic compounds and carboxylic acids (Wilcoxon rank-sum test, $FDR < 0.1$, **Figure 3B**). The greatest difference was actually observed in the carboxylic acids that could be imported and consumed as carbon sources by yeast cells. Industrial strains, and especially brewing strains show generally high intracellular concentrations in Carboxylates Degradation and TCA Cycle pathways (Wilcoxon rank-sum test, $FDR < 0.1$). While conversely, higher intracellular levels were observed for Fatty Acid Biosynthesis for plant strains (**Figure 3B**). Interestingly, as the major membrane lipid component, fatty acids (saturated fatty acids in particular) are highly associated with non-acidic stress response (ethanol, salt, oxidative and thermal stresses) in plants and fungi [10]. Thus, in the resistance to non-acidic conditions such as alcohol and heat, plant strains could outperform the industrial ones due to the biosynthetic activity, cellular abundances and composition of intracellular fatty acids, which aligns with our observations in resistance scores very well (**Figure 2C**).

### Genome diversity among industrial and natural *S. cerevisiae* strains

We sequenced the genomes of all 36 strains to a minimum depth of 52x, with a median of 96x. By using S288c as the reference genome, we identified 342,325 SNP loci, 19,347 small insertion and 17,457 small deletion (InDel) loci among all strains (**Table S1** & **Dataset S3**), with an average SNP/InDel count of 68,928 per strain, which were comparable to previous studies [11-13]. In the constructed phylogenetic tree, three major clades were discovered (**Figure 4A**). We could observe an

obvious enrichment of industrial strains in the clade of S288c (13 out of 20 strains, 65.0%). Notably, all the seven strains isolated from Brazilian bioethanol industry with distinct origins and genotypic categories [14] were clustered in this branch (within purple shade in **Figure 4A**). This suggests the possibility of common ancestors, while the long pairwise genetic distances also indicate high divergences in their strain-specific genetic makeups and evolutionary paths. All the six strains with high heterozygosity rate, including CLIB324, GDB135-h, T73 (97.7 ~ 98.3%, brewing or bakery strains), AL1, GDB325 and GDB379 (42.1 ~ 52.4%, all bioethanol strains), were present in the same subgroup, while all the other non-haploid strains' SNPs/InDels were generally in homozygous form (heterozygosity rate < 15%). These six industrial strains have shown some unique resistance to acids: CLIB324 was the most resistant strain (in both Performance and Robustness) to formic acid and the most Robust against pyruvic acid; T73 shows the highest Performance under pyruvic acid whereas AL1 was the most resistant to ferulic acid (**Figure 2A**). More interestingly, all seven strains in the other subgroup of the same clade (YJM978 – L.1528), were also among the top eight strains with highest InDel rates (> 9.0%). On the contrary, more environmental strains were found in the clade of T7 (7 out of 9 strains, 77.8%, within yellow shade in **Figure 4A**). The SK1 clade was the most divergent cluster. Y55 and PW5, two of the top three strains with universal resistance, locate in this cluster with long genetic distances from other strains. The only two industrial strains in this cluster, DBVPG6044 and NCYC110, had generally poor stress resistance (except DBVPG6044's Robustness against succinic acid) (**Figure 2A**) distinguishing them from Y55 and PW5 from the same clade.

The analysis of copy-number variations (CNVs) resulted in the identification of 2,916 unique CNV-affected genes in total (**Table S1** & **Dataset S3**), while most of the large-scale CNVs are located in subtelomeric regions, in agreement with a recent study [15]. Chromosome I duplication was discovered in five strains (four environmental and one industrial), while all other strain-specific

chromosome-scale duplications were captured in industrial strains (**Figure S4A**): NCYC110 – chrV, CLIB215 – chrXII, GDB135-h – chrIII and GDB325 – chrVI. PW5 (environmental) and DBVPG6044 (industrial, triploid) are also the two strains that have large number of duplicated genes (> 200). In addition, extremely high copy-numbers of tandem repeat segments (> 10) were observed in seven strains, with four of them being bioethanol strains (AL1 – 82x in chrI, CLIB382 – 11x in chrVII, Ethanol Red – 18x in chrX, GDB325 – 34x in chrIX), together with one food (KKYS2-h – 13x in chrVI), one plant (RM11 – 22x in chrVII) and one clinical (YJM975 – 701x in chrX and 19x in chrXIV) strain. Except NCYC110, all the eleven aforementioned strains have good resistance (especially Robustness) to one or more acidic conditions (**Figure 2A**). In comparison, chromosome-scale loss events were only captured in chrI of YJM978, a clinical strain. The laboratory strain SK1 was another strain with large number of gene losses (> 800) and this strain was also the most resistant strain to acetic acid (**Figure 2A**).

Our *de novo* assembly and gene prediction suggested 7 to 25 potential novel non-S288c genes per strain, and most of these genes were non-metabolic genes and homologous genes from other *Saccharomyces* strains (**Dataset S3**). Five out of the eight strains with over 20 new genes were environmental strains. The strains CLIB382, UWOPS05-227.2 and UWOPS05-217.3 had the largest counts of new genes, whereas CLIB382 was also the most resistant strain to fumaric acid and 4-aminobenzoic acid (**Figure 2A**). Ten new genes were shared by UWOPS05-227.2 and UWOPS05-217.3 (both related with nectar of Bertram palm), which were also sharing the high resistance traits towards ferulic acid and 1,4-butanediol (**Figure 2A**). As these genes were absent in the reference strain S288c and largely uncharacterized, they have not been previously reported to be associated with the aforementioned phenotypes.

In the systematic comparisons between the genetic makeups of industrial and environmental strains (Brazilian bioethanol strains binned as one candidate, **Supplemental Note S1**), significantly more

heterozygous SNPs/InDels (Wilcoxon rank-sum test, *P* value = 4.1e-2), especially in bioethanol strains, and less InDels (*P* value = 3.6e-2) were observed in industrial strains (**Figure 4B-D**). When investigating the genes influenced by these variants (the ratio of SNPs/InDels falling in specific gene categories, or the number of genes affected by CNVs), we found relatively more SNPs/InDels in the transcription factor (TF) super family Helix-turn-helix (HTH) in all subcategories of industrial strains (*P* value = 1.7e-2) (**Figure 4C-D**). We also found significantly more duplicated TFs in industrial strains (bioethanol and brewing strains in particular), together with three TF super families, Zipper (ZIP), HTH and Other (TF not belonging to any of the ZIP, HTH nor Zinc finger (ZNF) super families) (**Figures 4C-D**, *P* value < 0.05). More gene deletions were observed in plant strains (Kruskal-Wallis test, *P* value < 0.05, **Figure 4D**), and more interestingly, deletions of TFs were only observed in plant species.

**Phenotypic prediction using reconstructed strain-specific genome-scale metabolic models**

In order to analyze metabolic differences between the strains and predict metabolic phenotypes, strain-specific Genome-scale Metabolic Models (GSMMs) were reconstructed by incorporating severe mutations, gene deletions and non-S288c genes (**Dataset S4, Supplemental Note S4**). The pairwise comparison of the strain-specific models shows that the networks differ by at most only 2% of total reactions (**Supplemental Note S4**). The models were able to predict strain differences in the utilization of 30 carbon sources and 5 nitrogen sources (**Supplemental Note S4**) in comparison with the experimental data for selected strains (**Dataset S5**). Due to a recent study [16], inter-strain differences could be observed in key metabolic fluxes simulated by strain-specific models, which could provide more meaningful biological insights than the reaction presence/absence comparisons. Thus, different activated fluxes were simulated by the strain-specific models (**Supplemental Note S4**). Higher fluxes of Pyruvate Mitochondrial Transport via Proton Symport and Pyruvate Dehydrogenase were observed in

environmental strains (Wilcoxon rank-sum test, *FDR* = 6.6e-2, **Figure 3C**), which are two key up-stream reactions before the TCA cycle, suggesting that environmental strains have relative higher energy flux from the pyruvate metabolism to the TCA cycle than industrial strains. When summing up the energy fluxes by different subsystems, we found that environmental strains also have higher fluxes in Fatty Acid Biosynthesis, Citric Acid Cycle (TCA Cycle) and Pyruvate Metabolism, while industrial strains have higher fluxes towards Fatty Acid Metabolism (degradation) (*FDR* < 0.1, **Figure 3C**).

**Identification of the genetic features and patterns associated with different stress resistance**

To identify the potential genetic contributors to stress resistance phenotypes, SNP-based and CNV-based genome-wide association studies (GWAS) were performed (**Figure 5**). For the SNP-based GWAS, to reduce the impact from the false positive SNPs that were called from the highly divergent genomic regions, the core genome regions shared by all strains were annotated by *de novo* assembled contigs, and only the SNPs/InDels in the core genome were used as genotype markers (**Figure S4B & Supplemental Note S5**). In summary, 3,449 linkage disequilibrium (LD) blocks were identified, 165,358 base modification markers were used in the SNP-based core genome GWAS (Core-GWAS) and 880 CNV markers were used in the CNV-based GWAS. Significance cut-offs were obtained from the quantile-quantile plot of the *P* value distribution (**Supplemental Note S5**). In the SNP-based GWAS, significant markers for the Robustness and Performance rankings under non-acidic conditions were evenly distributed along the genome (**Figure 5B**), while the two rankings under acidic conditions showed different genomic hotspots: e.g., chrII for Performance and chrXIII for Robustness. In the CNV-based GWAS, the Robustness against acidic conditions always presented condition-specific genomic hotspots (especially for gain events): e.g., chrI – furfural, chrXIV – succinic acid, chrIX – fumaric acid, chrVIII – acetic acid and chrIII – pH, all in gain events (**Figure 5C**). While for Performance, hot regions tend to

be shared by multiple conditions: e.g., the loss events in chrV and chrVIII, and the gain event in chrXIV (**Figure 5C & Figure S4B**). In general, the GWAS profiles for the Performance under acidic and non-acidic conditions were relatively similar; on the contrary the profiles for the Robustness against acidic and non-acidic conditions are highly disparate (**Figure S4B**).

The Core-GWAS was successful in avoiding genomic regions with high variabilities (CNV regions, centromere, chromosome ends, break points of structural variations, mitochondrial DNA, etc.), thus the genes processed by the SNP-based and CNV-based GWAS were not highly overlapping: from the 1,818 and 2,931 genes treated in the SNP-based and CNV-based GWAS, respectively, only 794 genes were in the shared regions, while no gene was deemed significant from both SNP-based and CNV-based GWAS within the 26 individual rankings (**Figure S4B, Table S2 & Dataset S6**).

The patterns of the four GWAS profile groups (Acidic/Non-acidic × Robustness/Performance) were more obvious when studying the relative contribution from SNP/gain/loss events on specific gene categories (**Figure 6**). When looking at all genes sorted by GWAS *P* values and represented by the 75% quantile, the contribution patterns to the Robustness and Performance under non-acidic conditions were similar, while the Robustness against acidic conditions has relatively higher contribution from CNVs, especially gain events. In verified ORFs, the resistance to acidic conditions was slightly skewed to SNPs, while in uncharacterized genes, CNVs were presenting much higher contribution. Regarding transcription factors (TFs), in the TF super families ZNF and Other the resistances to acidic conditions were all highly contributed by gain events, which could be linked with the observation that the industrial strains have more TFs duplicated and are generally more resistant to acidic conditions. In the TF family HTH, the Performance under acidic conditions was skewed to SNPs, compared with the Performance under non-acidic conditions. Meanwhile, higher base modification rate in the HTH family was discovered in industrial strains compared to environmental strains.

To validate the GWAS results with literature-based knowledge, we compared the GWAS outcomes with the phenotype-associated gene lists in Saccharomyces Genome Database (SGD) [17] (**Supplemental Note S5**). The significant gene lists from GWAS are highly noisy since several genes always appear contiguous in significant genomic regions (LD blocks or CNV regions). Therefore it is possible that most of the genes receiving the same significant *P* values are just “passengers”. In order to identify “driver” genetic variants in the GWAS gene lists, we mapped the genes to protein-protein interaction networks (PPIN) and identified the core modules in the network by ModuleDiscoverer [18]. In the presented PPI networks, the GWAS gene lists associated to resistances to acids, ethanol, thermotolerance and lifespan (an SGD phenotype associated with the measurement of all our resistance scores), were found to be highly overlapping with the genes already recorded in SGD entries, suggesting a successful literature-based validation (**Figure 7**). When making comparisons between the GWAS modules with the modules constructed by randomly selected gene lists with the same lengths (5 random gene lists for each GWAS gene list), GWAS modules constantly show significantly higher modularity (more nodes in modules, more nodes in cliques, more internal edges, higher proportion of foreground nodes, Wilcoxon signed-rank test, $P < 1e-2$) and higher associations with SGD entries (more edges to SGD nodes, higher overlapping rate, higher overlapping rate of foreground nodes, $P < 1e-2$). Furthermore, additional GWAS-specific modules (SGD genes not highly observed in such modules) were also discovered, which represent valuable candidates for engineering target selection. Some of these modules were shared by multiple networks: the *IMA*/*MAL* module was shared by all four networks; the large *YRF*/Uncharacterized gene module was shared by lifespan, thermotolerance and resistance to acids; the *COX*/*ATP*/Mitochondrial gene module was shared by resistances to acids and ethanol; the *FRE*/*FIT* module was shared by thermotolerance and lifespan.

## DISCUSSION

The mechanisms influencing the yeast stress resistance are complex and the genetic interpretation require more systematical investigations. Our comprehensive omics study, incorporating the discoveries from phenome, genome, metabolome, fluxome, GWAS and the interactome, has been successful in establishing multi-dimensional associations among strain metadata, genotype and resistance to different conditions.

Among the studies with large-scale phenotype screening, previous works focused on the metabolome [11, 19] and basic physiology [11, 20], while in this work we also developed a systematic scoring and ranking protocol, to characterize the strains' industrial potential regarding resistance to multiple industrially relevant stresses with the possibility to set different weights to parameters according to different engineering purposes. Compared with previous association studies that aimed to establish genotype-to-phenotype links by using only SNPs [11, 21], or performed SNP-based GWAS in both core- and pan-genome region [15], here we introduced SNP-based core genome GWAS and CNV-based GWAS to reduce false positive observations introduced by the pan-genome diversity of the yeast population (common GWAS procedure), and take both SNPs/InDels and structural variations into consideration. The reconstruction of strain-specific GSMMs and the prediction of energy fluxes provide a valuable resource for engineers working with different strains for different purposes.

To summarize our evidences in the phenotypic and genotypic screening and make some basic comparisons between industrial and environmental strains and among their subcategories, we have established several interesting associations. Regarding genotype, industrial strains tend to have higher heterozygosity (especially bioethanol strains), which could be a result of genome fusion during fermentation processes, lower InDel rate, higher SNPs/InDels rate in the TF family HTH, and higher frequencies in large-scale and high-intensity duplication events (especially affecting transcription

factors), and lower possibility to acquire new genes. At the phenotypic level, industrial strains have relatively low extracellular aromatic compound and carboxylic acid levels and lower pathway activity in TCA cycle but relatively high in ethanol metabolism, which meets their functionality in anaerobic fermentation. Consequently, the accumulation of aliphatic acids from TCA cycle, such as succinate and fumarate, may therefor contribute to the strains' high innate resistance (especially Robustness) to acidic conditions (bioethanol and food strains in particular). These resistances were found to be highly associated with duplication events, especially in uncharacterized genes and TFs, as well as the SNPs/InDels in the helix-turn-helix TF family. On the other hand, regarding the genotype, environmental strains tend to have lower heterozygosity but higher InDel rate, wider range of phylogenetic diversity, and higher frequencies in small-scale and random loss events, low possibility in large-scale duplications and higher tendency to acquire new genes – especially plant strains, where deletions of TFs were also only observed in this subcategory. Summarizing the phenotype, environmental strains, especially the plant species, tend to have relatively high extracellular metabolite levels, higher energy flux towards TCA cycle and high activity in fatty acid biosynthesis, which also improves the resistances in wide range. They are in general not outstanding in the Robustness to extreme conditions (especially the acidic ones) but tend to have wider ranges of resistance (especially the Performance) to multiple conditions, in particular the non-acidic conditions. These phenotypes have relatively high associations with gene loss events.

Regarding the selection of platform strains our study revealed that the most commonly used *S. cerevisiae* strains, S288c and CEN.PK113-7D, performed rather poorly among all screened strains with regard to the bioprocessing-relevant stress resistance. Tremendous amount of engineering work has been performed using these two strains as host strains [4, 5], but few evaluations have been made on whether they are the best possible ones to use in industrial settings. Our findings suggest that many environmental

strains also have potential industrial applications due to their unique stress resistance patterns when compared with industrial strains. More impressively, none of the top three strains with resistance to multiple stress conditions, Y55, RM11 and PW5, was isolated from current industrial yeast fermentations but all were initially isolated from plants, and thus could serve as promising platform strains for bioprocessing purposes. The recent developments in CRISPR methods have also made it possible to genetically engineer almost any yeast strain [22], thus non-standard or polypoid strains could be introduced in metabolic engineering projects [23].

From the point of view of genetics and evolution, evidences from previous studies match our observations and assumptions quite well [24, 25]; from the engineering point of view, it has been reported that the duplication or overexpression of several TFs [26] and the mutagenesis of Spt15 [8], a TF from the HTH family, could boost the stress resistance during fermentation. Remarkably, Spt15 is also the first priority suggested by our GWAS as potential engineering target (**Dataset S6**), thus we believe that the potential engineering targets and engineering strategies suggested by our study are of high confidence. Besides the previously revealed mechanisms and strategies (overexpression of TFs and transporters, mutagenesis of TFs, chromosomal duplications in industrial strains), the potential genotype resources and engineering targets associated with different stress conditions such as SNPs/InDels and loss events of environmental strains, the TFs and significant genes which have not been previously engineered, uncharacterized gene clusters, tandem repeats, are new outcomes arising from this study. Therefore, we propose here several sources to select potential engineering targets as indicated below:

1. *The extreme genotypes found in the well-performing strains*.

   For instance, the suggested platform strain Y55 has below average resistance to 4-aminobenzoic acid and ferulic acid. To improve the resistance to these and related compounds, genomic features from AL1, CLIB215 and GDB325, the most resistant strains to the selected conditions, could be introduced.

Large-scale duplications of chrXII (3~4 copies from CLIB215), chrVI (6 copies from CLIB215), and the repeat region of chrVI:742212-745751 (82 copies from AL1), could be engineered to Y55.

2. *The most significant variants and genes suggested by GWAS*.

For example, for the resistance to ferulic acid, the LD blocks chrXIV: 605937-607882 (–lg (*P* value) = 11.76) and chrIX:151566-161597 (–lg (*P* value) = 11.46) were identified as the most significant markers for Robustness and Performance respectively. The SNPs/InDels in these blocks and the overlapped genes could be considered as the first priority.

3. *The variants or genes by multiple GWAS profiles regarding resistance to different conditions*.

The CNV region YHR218W-YHR219 (with both gain and loss events from different rankings) was found significantly associated with seven different stress conditions. The gain event of YNR059W-YNR062C was associated with five acidic conditions. The putative gene YER138W-A with unknown function was shared by six acidic conditions, while its neighbor YER148W/*SPT15* (also significant in multiple conditions) has been previously reported to be highly associated with ethanol resistance [8]. *SKY1* was also a significant gene shared by 4 conditions, which has been proved to be associated with osmotic tolerance [27].

4. *Specific gene sets that were captured in multi-dimension studies*.

Three transcription factors, Spt15 (HTH) (previously reported [8]), Ecm22 (ZNF), Bur6 (Other TF) were identified by GWAS, among which, Ecm22 and Bur6 were from the CNVs while Spt15 (HTH) was from the SNPs. The gain event of YNR059W-YNR062C was associated with the Robustness against multiple acidic conditions. These cases from GWAS can also match the analyses in the genotyping.

5. *The modules discovered in the phenotype associated PPIN*.

The *COX*/*ATP*/Mitochondrial Gene module is part of the respiratory chain and the genes were found to provide protection against acetic acid and other stresses [28], due to its association to respiration, redox and ion balance, which could serve as promising engineering targets for the ethanol and acid resistance. The *FRE*/*FIT* module is associated with the iron transport in cell wall, whereas it is also reported that the tolerance to inorganic acid and weak acid is correlated with iron uptake [29, 30]. The *PAU* module (the seripauperin gene family) which encodes yeast cell wall mannoproteins [31], was proved to be responsible for anaerobiosis and environmental stress [32]. As the gain event of uncharacterized genes was discovered to be associated to the acid resistance, the modules with uncharacterized and dubious genes (*YRF*/Uncharacterized module, *YPR* and *YHR* modules for ethanol resistance, *SOR*/*HKX* module for thermotolerance, etc.) could also be used to characterize the new functions and the association with stress resistance. Other network-specific modules, especially the ones with multiple uncharacterized genes, could also be considered as engineering targets. For instance, the *PYC/LPD/KGD/CIT/ACS/GDH* module has genes from Krebs cycle, the *GLK/SOR/HXK* module participates the phosphorylation, and the *SCH/YPK* module has genes from the Fermentable Growth Medium (FGM) signalling pathways. These genes from carbon metabolism were verified to be associated with the resistance to alcohols and acids [28].

**POTENTIAL IMMPLICATIONS**

The present work has not only revealed successfully the phenotypic and genotypic divergence of a representative strain collection and discussed the underlying evolutionary mechanisms, but also proposed a practical toolbox for platform strain selection and identification of new engineering targets, a consistent number of which were already presented here. Technically, the innovative methods used in this study, including the comprehensive resistance score calculation, the strain-specific GSMM and fluxome

construction, the analytically rigorous core-genome and CNV-based GWAS, and the noise-reducing PPIN module discovery, are also applicable to other geno- and phenotyping projects, especially the strain level population studies with high inter-strain genetic diversity.

## METHODS

### Yeast strain collection storage

A collection of 36 *S. cerevisiae* strains were studied in this research, and the details were summarized in **Supplemental Note S1**. The strains were stored at -80°C in cryogenic tubes containing YPD medium with 20% (vol/vol) glycerol. From the stock tubes, a sterile inoculation loop was used to transfer cells onto YPD plates. Plates were incubated for 48 h before cells were used for pre-cultivation.

### Medium for pre-cultures and under various stress conditions

Pre-cultures of yeast strains were grown in a defined mineral medium containing 7.5 g/L $(NH_4)_2SO_4$, 14.4 g/L $KH_2PO_4$, 0.5 g/L $MgSO_4{\bullet}7H_2O$, 2 mL/L of trace element solution, 1 mL/L vitamin solution (prepared according to Verduyn *et al.* [33]) and 20 g/L glucose. The pH of the salts together with trace elements was adjusted to 6.0 with NaOH before autoclaving. The glucose solution was autoclaved separately before being added to the salt solution together with the filter-sterilized vitamin solution. Pre-cultures were made in 24-deepwell plates (CR1424, Enzyscreen, The Netherlands) containing 1 mL mineral medium in each well. A single yeast colony was inoculated from an YPD agar plate and grown for 20 h at 30°C and 300 rpm in an incubator with 51 mm shaking orbit. Media for cultivation under various stress conditions were introduced in detail in **Supplemental Note S2**.

### Cultivation in the Growth Profiler 1152 and data processing

Yeast strains were pre-cultivated as described above and harvested by centrifugation then inoculated to 96-weell microplates. The inoculated plates were then placed in the Growth Profiler 1152 and growth

was monitored for ca. 66 hours. Details of cultivation, biological replicates and data processing were described in detail in **Supplemental Note S2**.

**Investigation of carbon and nitrogen source utilization with Biolog Phenotype Microarrays**

Yeast strains CEN.PK113-7D, S288C and Ethanol Red were pre-grown in 50 mL conical tubes using 5 mL YPD medium at 30°C and 280 rpm for 16 h. These pre-cultures were used to inoculate 250 mL shake flasks with 25 mL YPD medium at an initial OD of 0.2. The strains were cultivated until the OD reached ~1 (ca. 5 h) at which point the cells were washed twice in sterile water. After the final wash the cells were concentrated to an OD of 4.2 by diluting with an appropriate volume of sterile water. The cell suspensions were diluted 48-fold when added to the media specific for PM1-3, resulting in a starting OD of 0.0875. After inoculation, the PM plates were placed in the OmniLog incubator at 30°C and the development of the colored dye was measured every 15 min for 83 h. Data files were converted and exported to Excel using the dedicated software from the supplier.

**Intra- and extracellular metabolome screening**

Intracellular and extracellular metabolites of the yeast strains growing in glucose media were profiled using GC-MS. Metabolite identification and normalization of GC-MS data was performed using the AMDIS (Automated Mass Spectral Deconvolution and Identification System) software. Intra- and extracellular metabolites were assigned to different compound classes, and the intracellular metabolites were also assigned to different pathways and pathway groups. The experimental protocol was described in detail in **Supplemental Note S3**.

**Genome sequencing and estimation of strain ploidy**

The genomes of the 36 *S. cerevisiae* strains were sequenced using the Illumina MiSeq or HiSeq 2000 platform. Paired-end sequencing libraries with 350 bp insert size were prepared with the TruSeq Nano DNA kit and sequenced with either 150 nt or 250 nt read length. Data quality control and filtering were

performed by FastQC. Strain ploidy was determined by relative comparison of the DNA amount of the $G_0$-$G_1$ gated population of the target strains with reference *S. cerevisiae* strains of known ploidy, measured by flow cytometry following the procedure previously described [22]. To avoid the misidentification of aneuploid strains as polyploid strain in the flow cytometry analysis, the ploidy estimation results were verified by the allele frequencies of the heterozygous SNPs/InDels.

**Reads mapping, variant calling and annotation**

Reads were mapped to the S288c reference genome (SGD release 64 [17]) using BWA (v0.7.12, module *mem*) [34]. A minimum coverage of 50x, after filtration, was set as requirement for each strain. SNPs/InDels were called and filtered using the Genome Analysis Toolkit (GATK) 3.4 [35, 36], with the sequential steps to include RealignerTargetCreator, IndelRealigner, HaplotypeCaller (with parameter "-rf BadCigar") and VariantFiltration (with parameter "--filterExpression "DP < 10 || QD < 2.0 || FS > 60.0""). SNPs and InDels were annotated by SnpEff using the *S. cerevisiae* database version EF4.69 [37]. The 800 bp upstream regions of the genes were included as potential regulatory sequences. CNVs were detected using Control-FreeC [38], whereas the reads of the S288c haploid strain were used as the reference genome. Gene fully covered by the CNV regions were labeled as affected genes. If a gene was partially overlapped with a CNV region, gain event would not be assigned due to the incompleteness of the obtained copies, while loss or deletion event (when the copy-number of the CNV region is zero) will be assigned to this gene, as at least one copy of the gene was truncated.

**Population structure analysis**

The consensus sequences of the 36 strains were generated using GATK [36] based on the SNP set. Protein sequences were translated from the ORFs and used for the Neighbor-Joining tree building by TreeBest [39].

***De novo* assembly and ORF prediction**

*De novo* assemblies were performed with Newbler version 2.8 using the default parameters. To identify the potential novel genes in the yeast population, compared to the reference genome S288c, ORF predictions were performed with YGAP [40] based on the *de novo* assemblies. For the genes of which no S288c homologous gene was annotated in YGAP, we further extracted the sequences and searched against NCBI nr protein data set [41] using BLASTX [42]. The ORFs with at least one valid hit to S288c (identity ≥ 95% and *E* value < 1e-5) were removed from the potential novel gene list and treated as miss-identifications of the YGAP pipeline.

**Strain-specific Genome-scale Metabolic Model (GSMM) construction**

Strain-specific GSMMs were constructed from the starting model iMM904 [43]. Severe mutations of consequences of gene truncation, elongation or deletion were considered in different severity levels, as well as the new reactions introduced by non-S288c genes (**Supplemental Note S4**). Reactions were annotated by the UniProt database [44] and MetaNetX database [45]. The mixed integer linear programming (MILP) algorithm was applied to build the strain-specific models (**Supplemental Note S4**). Carbon and nitrogen source utilization and fluxes were simulated with the FRAMED package using Gurobi 6.5 (**Supplemental Note S4**).

**Genome-wide association studies (GWAS) for resistance rankings**

GWAS were carried out for SNPs/InDels and CNVs, respectively (as called SNP-based and CNV-based GWAS). Only SNPs/InDels in core-genome regions and with MAF > 5% were used in the SNP-based GWAS. CNV markers were defined due to the overlap relationships and were used for gain and loss event separately. Strain rankings were used as phenotypic values. The mixed-model based method Efficient Mixed Model Association (EMMA [46]) was applied as the main algorithm in GWAS. Details of the core-GWAS markers, CNV marker identification, transformation of genotypic values, linkage disequilibrium (LD) block identification and *P* value assignment for genes were described in detail in

**Supplemental Note S5**. Different significance cut-offs for SNPs/gain/loss markers were set according to the departure of observed *P* value from the predicted *P* value distribution (**Supplemental Note S5**).

**Gene categories used in genotyping and GWAS**

The basic gene information was acquired from SGD [17], including gene ID, symbol name, the ORF type (verified, uncharacterized and dubious), EC number. The transcription factor list, with the super family classification, and the regulatory relationships, were collected from SGD [17] and YEASTRACT [47]. The GO-Slim Terms were acquired from SGD [17]. The metabolic pathways (YeastCyc) were downloaded from SGD [17] and MetaCyc [48]. The phenotype-associated gene lists were grasped from the Yeast Phenotype Ontology from SGD [17]. Among the gene and phenotype association entries, only the entries with positive or negative effects to phenotype were reserved, and the ones with neutral or unclear consequence were removed. When applied with GWAS profiles, the SGD entries were re-classified according to the phenotype classification and used chemicals (**Supplemental Note S5**).

**The discovery of the PPI modules from SGD gene list and GWAS profile**

The PPI network of *S. cerevisiae* was acquired from the STRING [49] database and regulatory modules were computed by ModuleDiscoverer [18] for both SGD gene lists and GWAS profiles (see details in **Supplemental Note S5**).

**Statistical analysis**

All statistical analyses were performed in R. For comparative analysis between industrial and environmental strains, Wilcoxon rank-sum tests were performed. For comparative analysis among different strain subcategorises, Kruskal-Wallis tests were performed. For multiple comparisons, Benjamini–Hochberg procedure was used to calculate the False Discovery Rate (FDR), $FDR < 0.05$ or $FDR < 0.1$ was used as the significance cut-off.

Spearman's tests with Bonferroni adjustment were performed to correlate the genotypic or phenotypic features, where *FDR* < 0.05 was used as significance cut-off.

The mixed-model based method Efficient Mixed Model Association (EMMA) [46] was applied as the main algorithm in GWAS.

**Data visualization**

R [50] and corresponding packages including ggplot2, ggtree, heatmap.plus and matplotlib were used for illustration of statistical results. Cytoscape 3.6.0 [51] was used to visualize the analyses incorporating network and topology.

**Availability of data**

The whole-genome sequence data have been deposited in the NCBI Sequence Read Archive (SRA; http://www.ncbi.nlm.nih.gov/sra) under accession number SRR6114130 to SRR6114127.

The metabolomic data have been deposited in EBI MetaboLights with identifier MTBLS780.

**DECLARATIONS**

**List of abbreviations**

ABE: Acetone, n-Butanol, Ethanol

AMDIS: Automated Mass Spectral Deconvolution and Identification System

CI: confidence interval

CNV: copy number variation

Core-GWAS: SNP-based core genome GWAS

EC: Enzyme Commission

EMMA: Efficient Mixed Model Association

FDR: false discovery rate

FGM: fermentable growth medium

GC-MS: Gas Chromatography-Mass Spectrometer

GO: Gene Ontology

GPR: gene-protein-reaction

GSMM: Genome-scale metabolic models

GWAS: Genome-wide association studies

HTH: Helix-turn-helix

InDel: insertion and deletion

KEGG: Kyoto Encyclopedia of Genes and Genomes

LD: linkage disequilibrium

MAF: minor allele frequency

MCF: methyl chloroformate

MILP: mixed integer linear programming algorithm

NCBI: National Center of Biotechnology Information

OD: optical density

ORF: open reading frame

*pdf*: probability density function

PI: Parameter Influence

PIA: Parameter Influence Analysis

PPIN: protein-protein interaction networks

RVA: Rank Variability Analysis

*S. cerevisiae*: *Saccharomyces cerevisiae*

SD: standard deviation

SGD: *Saccharomyces* Genome Database

SLM: Shape Language Model

SNP: single nucleotide polymorphism

SRA: Sequence Read Archive

TCA cycle: tricarboxylic acid cycle

TF: transcription factor

YGAP: Yeast Genome Annotation Pipeline

ZIP: Zipper

ZNF: Zinc finger

**Competing interests**

The authors declare no competing interests.

**Funding**

GP would like to thank Deutsche Forschungsgemeinschaft (DFG) CRC/Transregio 124 ‘Pathogenic fungi and their human host: Networks of interaction’, subproject B5. BB, LD, MJH and JF thank the Novo Nordisk Foundation for financial support.

**Authors' contributions**

BB designed the physiological characterization. BB and LD performed the experiments. KK performed the data analyses. BB, KK and DM wrote the initial manuscript. GP, JF and MJH supervised this study. DM performed the GSMM construction and simulation. HM and SVB performed the metabolomic measurements. JL advised the bioinformatics analyses. All authors conceived the project and approved the final version of manuscript.

**Acknowledgements**

The authors would like to thank Dr. Sebastian Vlaic from HKI, Germany for the development of ModuleDiscoverer and the assistance in PPIN analysis.

## SUPPLEMENTAL ITEMS

This article has 13 supplemental items, including the supplemental notes (including 5 sections), 2 tables, 4 figures and 6 datasets in individual documents.

**Supplemental Dataset Captions**

**Dataset S1**. The physiological characterization results.

**Dataset S2**. The intra- and extracellular metabolomes.

**Dataset S3**. The genetic makeups of the strains, including copy number variation profiles, SNP/InDel profiles and none-S288c genes predicted by YGAP.

**Dataset S4**. The strain-specific Genome-scale Metabolic Models (in .xml files).

**Dataset S5**. The growth data for CEN.PK, Ethanol Red and S288c in the utilization of different carbon and nitrogen sources.

**Dataset S6**. The genes significantly associated with Robustness and Performance rankings discovered by GWAS.

## REFERENCES


1. de Jong E, Higson A, Walsh P and Wellisch M. Bio-based chemicals value added products from biorefineries. IEA Bioenergy, Task42 Biorefinery. 2012.
2. Taylor R, Nattrass L, Alberts G, Robson P, Chudziak C, Bauen A, et al. From the sugar platform to biofuels and biochemicals. Final Report for the European Commission Directorate-General Energy N (ENER/C2/423-2012/SI2 673791). 2015.
3. Archer CT, Kim JF, Jeong H, Park JH, Vickers CE, Lee SY, et al. The genome sequence of E. coli W (ATCC 9637): comparative genome analysis and an improved genome-scale reconstruction of E. coli. BMC genomics. 2011;12:9. doi:10.1186/1471-2164-12-9.
4. van Dijken JP, Bauer J, Brambilla L, Duboc P, Francois JM, Gancedo C, et al. An interlaboratory comparison of physiological and genetic properties of four Saccharomyces cerevisiae strains. Enzyme Microb Technol. 2000;26 9-10:706-14.
5. Cakar ZP, Turanli-Yildiz B, Alkim C and Yilmaz U. Evolutionary engineering of Saccharomyces cerevisiae for improved industrially important properties. FEMS yeast research. 2012;12 2:171-82. doi:10.1111/j.1567-1364.2011.00775.x.
6. Li BZ and Yuan YJ. Transcriptome shifts in response to furfural and acetic acid in Saccharomyces cerevisiae. Applied microbiology and biotechnology. 2010;86 6:1915-24. doi:10.1007/s00253-010-2518-2.
7. Castle LA, Siehl DL, Gorton R, Patten PA, Chen YH, Bertain S, et al. Discovery and directed evolution of a glyphosate tolerance gene. Science. 2004;304 5674:1151-4. doi:10.1126/science.1096770.
8. Alper H, Moxley J, Nevoigt E, Fink GR and Stephanopoulos G. Engineering yeast transcription machinery for improved ethanol tolerance and production. Science. 2006;314 5805:1565-8. doi:10.1126/science.1131969.
9. Houghton-Larsen J and Brandt A. Fermentation of high concentrations of maltose by Saccharomyces cerevisiae is limited by the COMPASS methylation complex. Appl Environ Microbiol. 2006;72 11:7176-82. doi:10.1128/AEM.01704-06.
10. Upchurch RG. Fatty acid unsaturation, mobilization, and regulation in the response of plants to stress. Biotechnol Lett. 2008;30 6:967-77. doi:10.1007/s10529-008-9639-z.
11. Skelly DA, Merrihew GE, Riffle M, Connelly CF, Kerr EO, Johansson M, et al. Integrative phenomics reveals insight into the structure of phenotypic diversity in budding yeast. Genome research. 2013;23 9:1496-504. doi:10.1101/gr.155762.113.
12. Strope PK, Skelly DA, Kozmin SG, Mahadevan G, Stone EA, Magwene PM, et al. The 100-genomes strains, an S. cerevisiae resource that illuminates its natural phenotypic and genotypic variation and emergence as an opportunistic pathogen. Genome research. 2015;25 5:762-74. doi:10.1101/gr.185538.114.
13. Liti G, Carter DM, Moses AM, Warringer J, Parts L, James SA, et al. Population genomics of domestic and wild yeasts. Nature. 2009;458 7236:337-41. doi:10.1038/nature07743.
14. da Silva-Filho EA, Brito dos Santos SK, Resende Ado M, de Morais JO, de Morais MA, Jr. and Ardaillon Simoes D. Yeast population dynamics of industrial fuel-ethanol fermentation process assessed by PCR-fingerprinting. Antonie Van Leeuwenhoek. 2005;88 1:13-23. doi:10.1007/s10482-004-7283-8.

15. Peter J, De Chiara M, Friedrich A, Yue JX, Pflieger D, Bergstrom A, et al. Genome evolution across 1,011 Saccharomyces cerevisiae isolates. Nature. 2018;556 7701:339-44. doi:10.1038/s41586-018-0030-5.
16. Monk JM, Koza A, Campodonico MA, Machado D, Seoane JM, Palsson BO, et al. Multi-omics Quantification of Species Variation of Escherichia coli Links Molecular Features with Strain Phenotypes. Cell Syst. 2016;3 3:238-51 e12. doi:10.1016/j.cels.2016.08.013.
17. Cherry JM, Hong EL, Amundsen C, Balakrishnan R, Binkley G, Chan ET, et al. Saccharomyces Genome Database: the genomics resource of budding yeast. Nucleic acids research. 2012;40 Database issue:D700-5. doi:10.1093/nar/gkr1029.
18. Vlaic S, Conrad T, Tokarski-Schnelle C, Gustafsson M, Dahmen U, Guthke R, et al. ModuleDiscoverer: Identification of regulatory modules in protein-protein interaction networks. Sci Rep. 2018;8 1:433. doi:10.1038/s41598-017-18370-2.
19. Breunig JS, Hackett SR, Rabinowitz JD and Kruglyak L. Genetic basis of metabolome variation in yeast. PLoS Genet. 2014;10 3:e1004142. doi:10.1371/journal.pgen.1004142.
20. Borneman AR, Desany BA, Riches D, Affourtit JP, Forgan AH, Pretorius IS, et al. Whole-Genome Comparison Reveals Novel Genetic Elements That Characterize the Genome of Industrial Strains of Saccharomyces cerevisiae. PLoS Genetics. 2011;7 2:e1001287. doi:10.1371/journal.pgen.1001287.
21. Kang K, Li J, Lim BL and Panagiotou G. MESSI: metabolic engineering target selection and best strain identification tool. Database : the journal of biological databases and curation. 2015;2015 doi:10.1093/database/bav076.
22. Stovicek V, Borodina I and Forster J. CRISPR–Cas system enables fast and simple genome editing of industrial Saccharomyces cerevisiae strains. Metabolic Engineering Communications. 2015;2:13-22.
23. Zhang GC, Kong, II, Kim H, Liu JJ, Cate JH and Jin YS. Construction of a quadruple auxotrophic mutant of an industrial polyploid saccharomyces cerevisiae strain by using RNA-guided Cas9 nuclease. Appl Environ Microbiol. 2014;80 24:7694-701. doi:10.1128/AEM.02310-14.
24. Bergstrom A, Simpson JT, Salinas F, Barre B, Parts L, Zia A, et al. A high-definition view of functional genetic variation from natural yeast genomes. Mol Biol Evol. 2014;31 4:872-88. doi:10.1093/molbev/msu037.
25. Caspeta L, Chen Y, Ghiaci P, Feizi A, Buskov S, Hallstrom BM, et al. Biofuels. Altered sterol composition renders yeast thermotolerant. Science. 2014;346 6205:75-8. doi:10.1126/science.1258137.
26. Alriksson B, Horváth IS and Jönsson LJ. Overexpression of Saccharomyces cerevisiae transcription factor and multidrug resistance genes conveys enhanced resistance to lignocellulose-derived fermentation inhibitors. Process Biochemistry. 2010;45 2:264-71.
27. Yoshikawa K, Tanaka T, Furusawa C, Nagahisa K, Hirasawa T and Shimizu H. Comprehensive phenotypic analysis for identification of genes affecting growth under ethanol stress in Saccharomyces cerevisiae. FEMS Yeast Res. 2009;9 1:32-44. doi:10.1111/j.1567-1364.2008.00456.x.
28. Henriques SF, Mira NP and Sa-Correia I. Genome-wide search for candidate genes for yeast robustness improvement against formic acid reveals novel susceptibility (Trk1 and positive regulators) and resistance (Haa1-regulon) determinants. Biotechnology for biofuels. 2017;10:96. doi:10.1186/s13068-017-0781-5.

29. Mira NP, Teixeira MC and Sa-Correia I. Adaptive response and tolerance to weak acids in Saccharomyces cerevisiae: a genome-wide view. OMICS. 2010;14 5:525-40. doi:10.1089/omi.2010.0072.
30. Abbott DA, Suir E, van Maris AJ and Pronk JT. Physiological and transcriptional responses to high concentrations of lactic acid in anaerobic chemostat cultures of Saccharomyces cerevisiae. Appl Environ Microbiol. 2008;74 18:5759-68. doi:10.1128/AEM.01030-08.
31. Marguet D, Guo XJ and Lauquin GJ. Yeast gene SRP1 (serine-rich protein). Intragenic repeat structure and identification of a family of SRP1-related DNA sequences. Journal of molecular biology. 1988;202 3:455-70.
32. Rivero D, Berna L, Stefanini I, Baruffini E, Bergerat A, Csikasz-Nagy A, et al. Hsp12p and PAU genes are involved in ecological interactions between natural yeast strains. Environ Microbiol. 2015;17 8:3069-81. doi:10.1111/1462-2920.12950.
33. Verduyn C, Postma E, Scheffers WA and Van Dijken JP. Effect of benzoic acid on metabolic fluxes in yeasts: a continuous-culture study on the regulation of respiration and alcoholic fermentation. Yeast. 1992;8 7:501-17. doi:10.1002/yea.320080703.
34. Li H and Durbin R. Fast and accurate long-read alignment with Burrows-Wheeler transform. Bioinformatics. 2010;26 5:589-95. doi:10.1093/bioinformatics/btp698.
35. DePristo MA, Banks E, Poplin R, Garimella KV, Maguire JR, Hartl C, et al. A framework for variation discovery and genotyping using next-generation DNA sequencing data. Nature genetics. 2011;43 5:491-8. doi:10.1038/ng.806.
36. McKenna A, Hanna M, Banks E, Sivachenko A, Cibulskis K, Kernytsky A, et al. The Genome Analysis Toolkit: a MapReduce framework for analyzing next-generation DNA sequencing data. Genome research. 2010;20 9:1297-303. doi:10.1101/gr.107524.110.
37. Cingolani P, Platts A, Wang le L, Coon M, Nguyen T, Wang L, et al. A program for annotating and predicting the effects of single nucleotide polymorphisms, SnpEff: SNPs in the genome of Drosophila melanogaster strain w1118; iso-2; iso-3. Fly. 2012;6 2:80-92. doi:10.4161/fly.19695.
38. Boeva V, Popova T, Bleakley K, Chiche P, Cappo J, Schleiermacher G, et al. Control-FREEC: a tool for assessing copy number and allelic content using next-generation sequencing data. Bioinformatics. 2012;28 3:423-5. doi:10.1093/bioinformatics/btr670.
39. Vilella AJ, Severin J, Ureta-Vidal A, Heng L, Durbin R and Birney E. EnsemblCompara GeneTrees: Complete, duplication-aware phylogenetic trees in vertebrates. Genome research. 2009;19 2:327-35. doi:10.1101/gr.073585.107.
40. Proux-Wera E, Armisen D, Byrne KP and Wolfe KH. A pipeline for automated annotation of yeast genome sequences by a conserved-synteny approach. BMC bioinformatics. 2012;13:237. doi:10.1186/1471-2105-13-237.
41. Pruitt KD, Tatusova T and Maglott DR. NCBI reference sequences (RefSeq): a curated non-redundant sequence database of genomes, transcripts and proteins. Nucleic acids research. 2007;35 Database issue:D61-5. doi:10.1093/nar/gkl842.
42. Gish W and States DJ. Identification of protein coding regions by database similarity search. Nature genetics. 1993;3 3:266-72. doi:10.1038/ng0393-266.
43. Mo ML, Palsson BO and Herrgard MJ. Connecting extracellular metabolomic measurements to intracellular flux states in yeast. Bmc Syst Biol. 2009;3:37. doi:10.1186/1752-0509-3-37.
44. UniProt C. The Universal Protein Resource (UniProt) in 2010. Nucleic acids research. 2010;38 Database issue:D142-8. doi:10.1093/nar/gkp846.

45. Moretti S, Martin O, Van Du Tran T, Bridge A, Morgat A and Pagni M. MetaNetX/MNXref--reconciliation of metabolites and biochemical reactions to bring together genome-scale metabolic networks. Nucleic acids research. 2016;44 D1:D523-6. doi:10.1093/nar/gkv1117.
46. Kang HM, Zaitlen NA, Wade CM, Kirby A, Heckerman D, Daly MJ, et al. Efficient control of population structure in model organism association mapping. Genetics. 2008;178 3:1709-23. doi:10.1534/genetics.107.080101.
47. Teixeira MC, Monteiro P, Jain P, Tenreiro S, Fernandes AR, Mira NP, et al. The YEASTRACT database: a tool for the analysis of transcription regulatory associations in Saccharomyces cerevisiae. Nucleic acids research. 2006;34 Database issue:D446-51. doi:10.1093/nar/gkj013.
48. Caspi R, Billington R, Ferrer L, Foerster H, Fulcher CA, Keseler IM, et al. The MetaCyc database of metabolic pathways and enzymes and the BioCyc collection of pathway/genome databases. Nucleic acids research. 2016;44 D1:D471-80. doi:10.1093/nar/gkv1164.
49. Jensen LJ, Kuhn M, Stark M, Chaffron S, Creevey C, Muller J, et al. STRING 8--a global view on proteins and their functional interactions in 630 organisms. Nucleic acids research. 2009;37 Database issue:D412-6. doi:10.1093/nar/gkn760.
50. R Core Team. R: A Language and Environment for Statistical. Vienna, Austria: R Foundation for Statistical Computing, 2015.
51. Shannon P, Markiel A, Ozier O, Baliga NS, Wang JT, Ramage D, et al. Cytoscape: a software environment for integrated models of biomolecular interaction networks. Genome research. 2003;13 11:2498-504. doi:10.1101/gr.1239303.

## FIGURE LEGENDS

**Figure 1. The stress conditions and resistance scores**. (**A**) The thirteen stress conditions and four-to-seven inhibitory levels in each condition. The colored cells indicate the number of strains that could grow in each experimental setting. (**B**) Calculation of the resistance scores (Performance and Robustness) based on five growth parameters. (**C**) The inhibitory level-specific Performance and Robustness scores of three strains under 1,4-butanediol condition: Y55, high ranking in both scores; DBVPG1373: good Robustness but poor Performance; PW5: good Performance but bad Robustness.

**Figure 2. The physiological characterization of various *S. cerevisiae* strains**. (**A**) The Performance and Robustness ranking values in all conditions from selected cases with different patterns. (**B**) The Performance and Robustness rankings for all strains in all conditions. Strains were sorted by their frequencies to be ranked in top 10 in all rankings. (**C**) The comparisons of different strain categories and subcategories in multiple resistance rankings. In the industrial vs. environmental comparisons, star marker suggests a significant difference (Wilcoxon rank-sum test, $FDR < 0.1$).

**Figure 3. The metabolomic profiles of various isolated strains of *Saccharomyces cerevisiae*.** (**A**) The metabolome of the yeast strains growing exponentially on glucose, and the consumed/excreted metabolites from the spent medium. The colors represent the combined contribution of metabolites in a pathway or compound class (**Supplemental Note S3**). For intracellular metabolites, a red color indicates that the group contains a majority of metabolites that are higher above the mean (and *vice versa* for blue). For extracellular metabolites, red indicates a high production level and blue indicates a high degree of consumption. Numbers in parenthesis indicate total number of metabolites in the group. Hierarchical

clustering was applied to the group variables using Euclidian distance between the strains. For data normalization before visualization, see details in **Supplemental Note S3**. (**B**) The comparisons of the rankings of metabolome groups, compound classes or metabolic pathways among different strain categories and subcategories. (**C**) The comparisons of the key fluxes and the fluxes in metabolic subsystems between industrial and environmental strains. PYRt2m: Pyruvate Mitochondrial Transport via Proton Symport, PDHm: Pyruvate Dehydrogenase. In the industrial vs. environmental comparisons, star marker suggests a significant difference (Wilcoxon rank-sum test, $FDR < 0.1$).

**Figure 4. The genetic characterization of various *Saccharomyces cerevisiae* isolated strains.** (**A**) A phylogenetic tree of all the strains was constructed based on identified SNPs/InDels and shows three main clades. The strain functional class, geographical origin and determined ploidy (for non-diploid strains) were marked. The genetic features were normalized to *Z* scores and illustrated in heat map. (**B-C**) The comparisons between the industrial and environmental strains on the number of variants **(B)** and the ratio of variants in specific gene sets (for SNPs/InDels) or the number of non-S288c genes and genes affected by CNVs) **(C)**. Star marker suggests a significant difference (Wilcoxon rank-sum test, $P < 0.05$). (**D**) The comparisons among different strain subcategories on the number of variants, SNP ratio in different gene sets, number of non-S288c genes and gene affected by CNVs.

**Figure 5. The genome-wide associations for the resistance rankings and the genomic hotspots**. (**A**) Genome-wide distributions of significant variants, for LDs or non-LD SNPs/InDels and CNVs respectively. (**B**) The total number of significant LDs and non-LD SNPs/InDels in each 50k bp genomic windows. (**C**) Genome-wide significance levels for CNV regions. Condition labels were colored by

acidic or non-acidic condition classification. A region was colored in red if its gain event was more significant than the loss event, or *vice versa*.

**Figure 6. The relative contributions from different variant source (SNP or CNV, gain or loss event) to different inhibitory conditions and scores**. The coordinates were calculated following the protocol in **Supplemental Note S5**. The first sub figure shows the relative contributions of the 75% quantile of all genes (sorted by *P* values). The other sub figures show the relative contributions in specific gene categories (verified or uncharacterized ORFs, TFs and different TF super families).

**Figure 7. The protein-protein interaction networks of multiple phenotypes, with SGD recorded entries and GWAS observations.** The node color indicates if a gene is from the SGD entry, GWAS profile or shared by both. The node border color and shape denote different gene categories. The eight GWAS-specific modules (not identified from the network built from the SGD gene list) that were shared by multiple PPI networks are highlighted in different background colors. Network-specific GWAS modules are shown in white background.

Figure 1

Click here to access/download;Figure;Figure_1.pdf

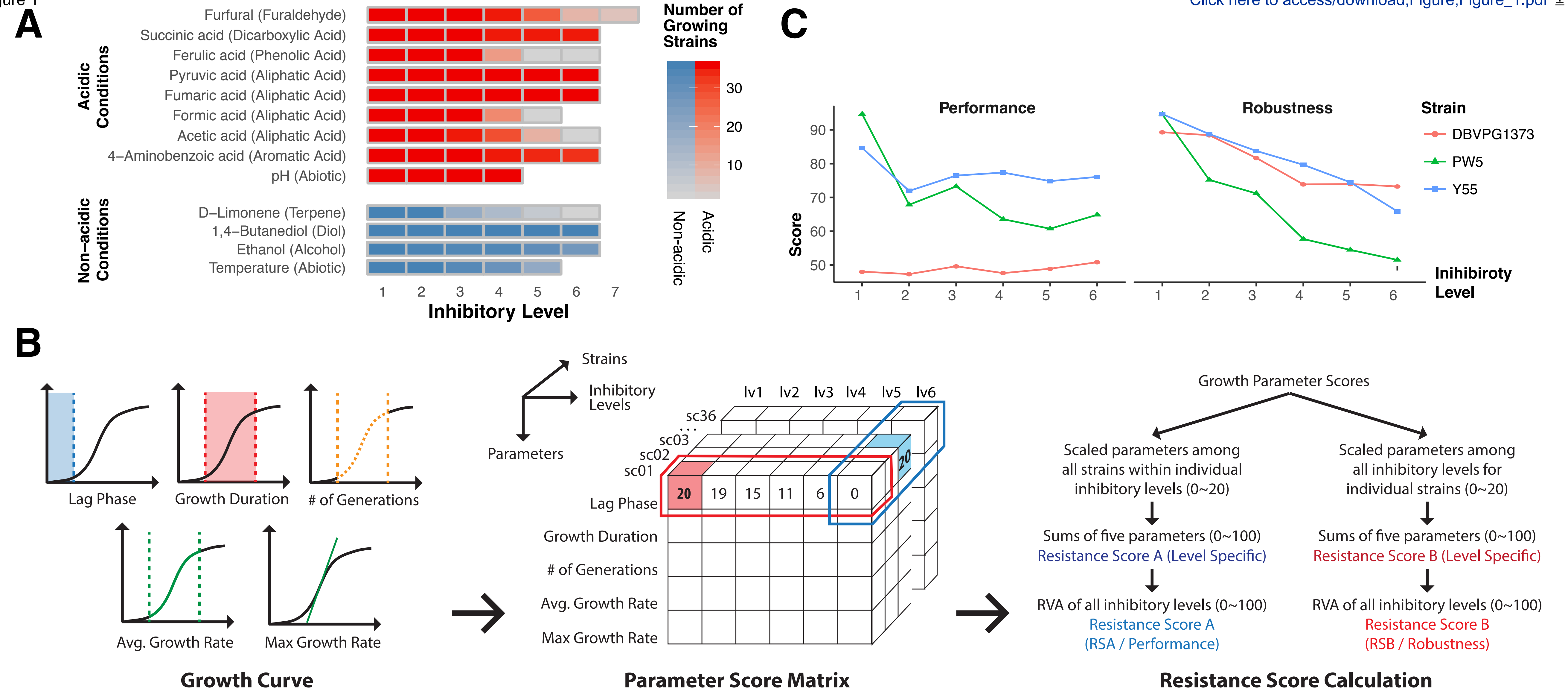

Figure 2

Click here to access/download;Figure;Figure_2.pdf

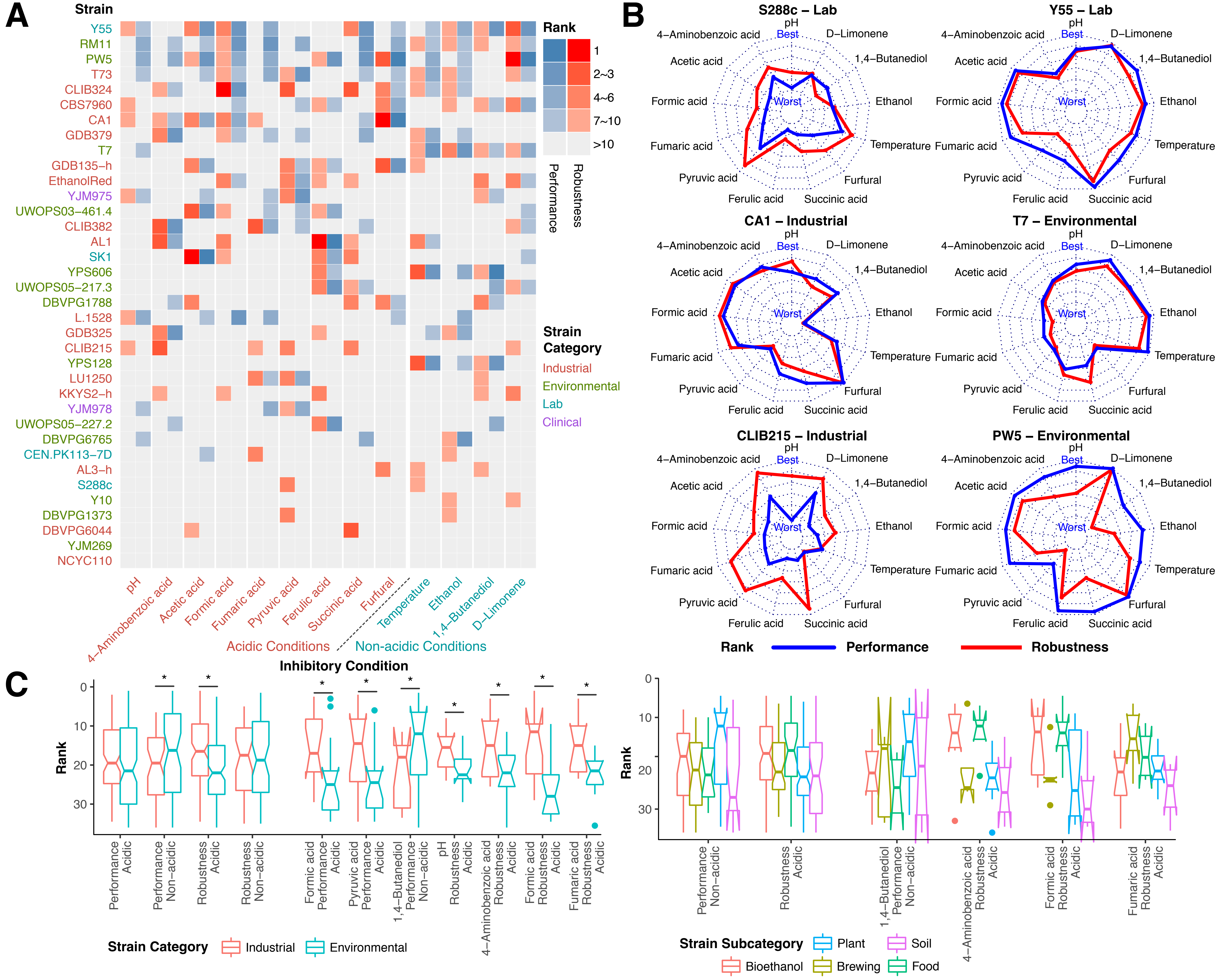

Figure 4

Click here to access/download;Figure;Figure_4.pdf

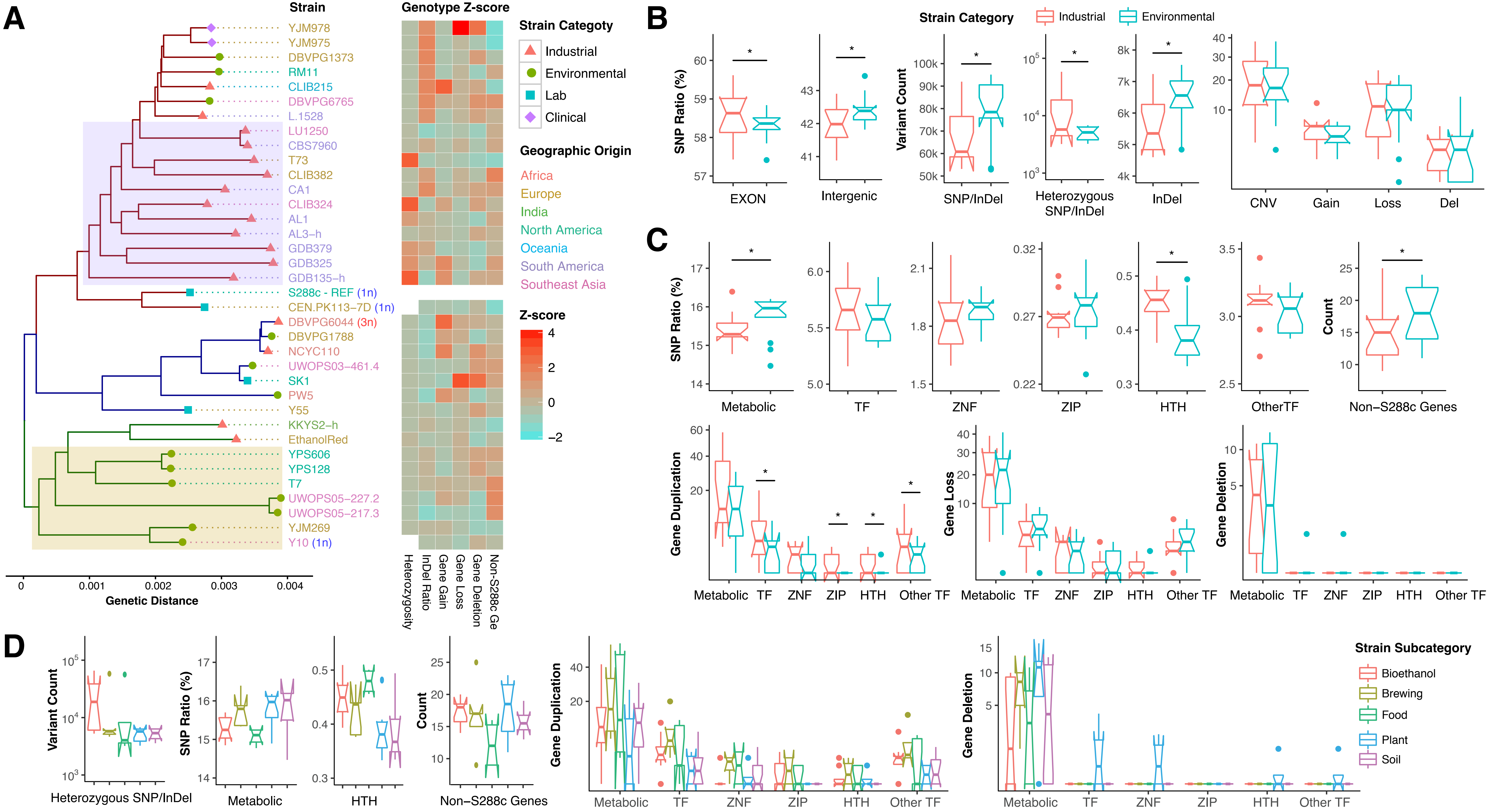

Figure 5

Click here to access/download;Figure;Figure_5.pdf

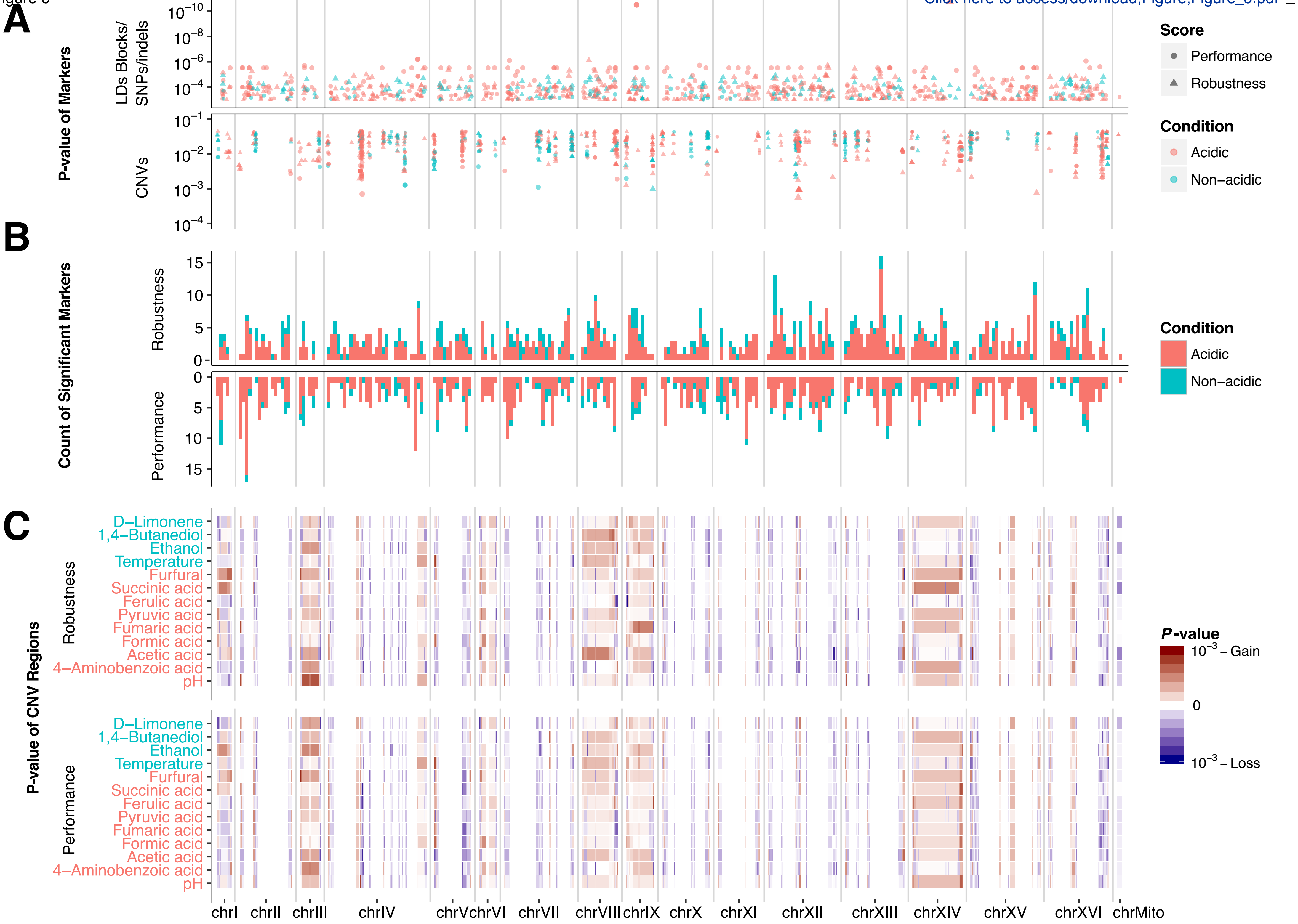

Click here to access/download;Figure;Figure_6.pdf

Figure 6

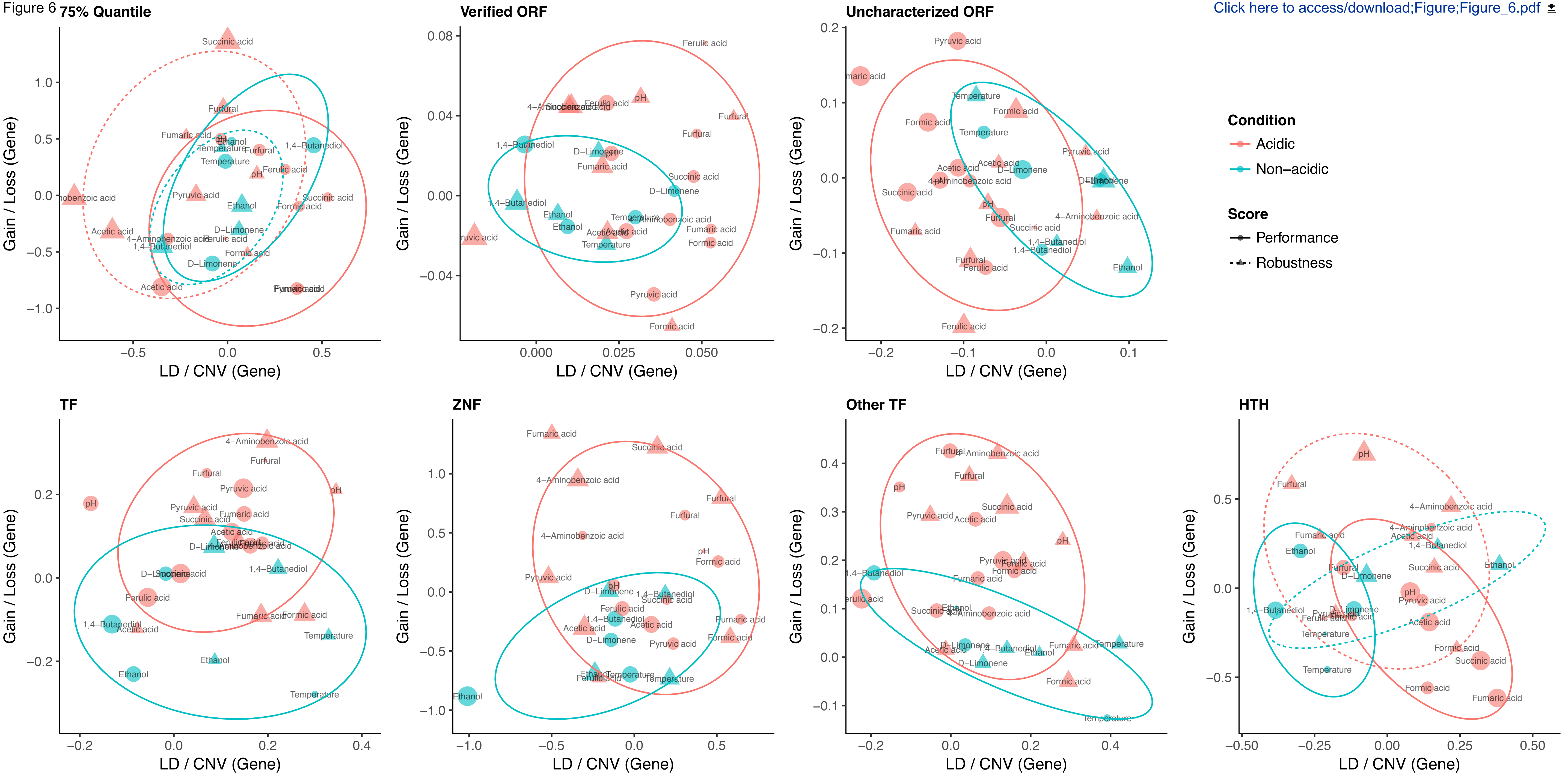

Figure 7

Click here to access/download;Figure;Figure_7.pdf

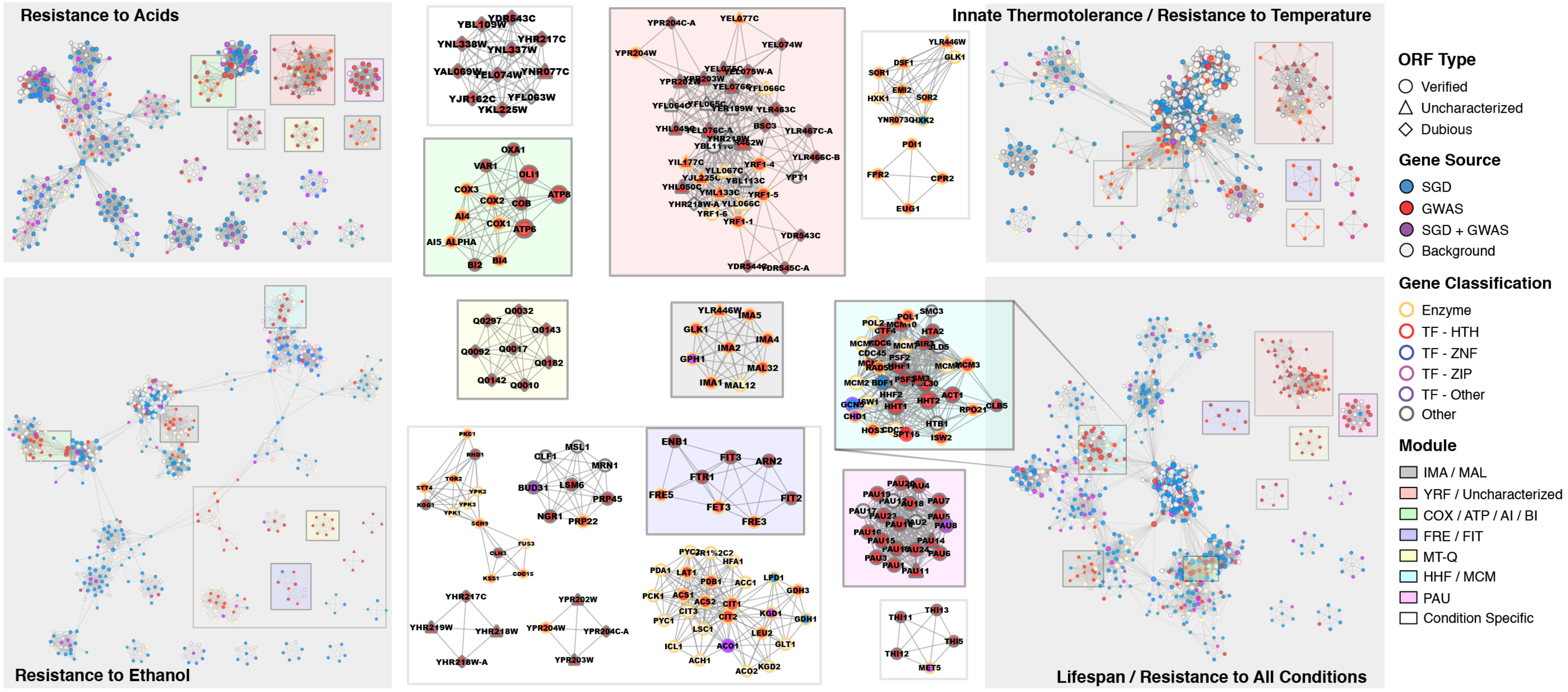

Figure 3

Click here to access/download;Figure;Figure_3.pdf

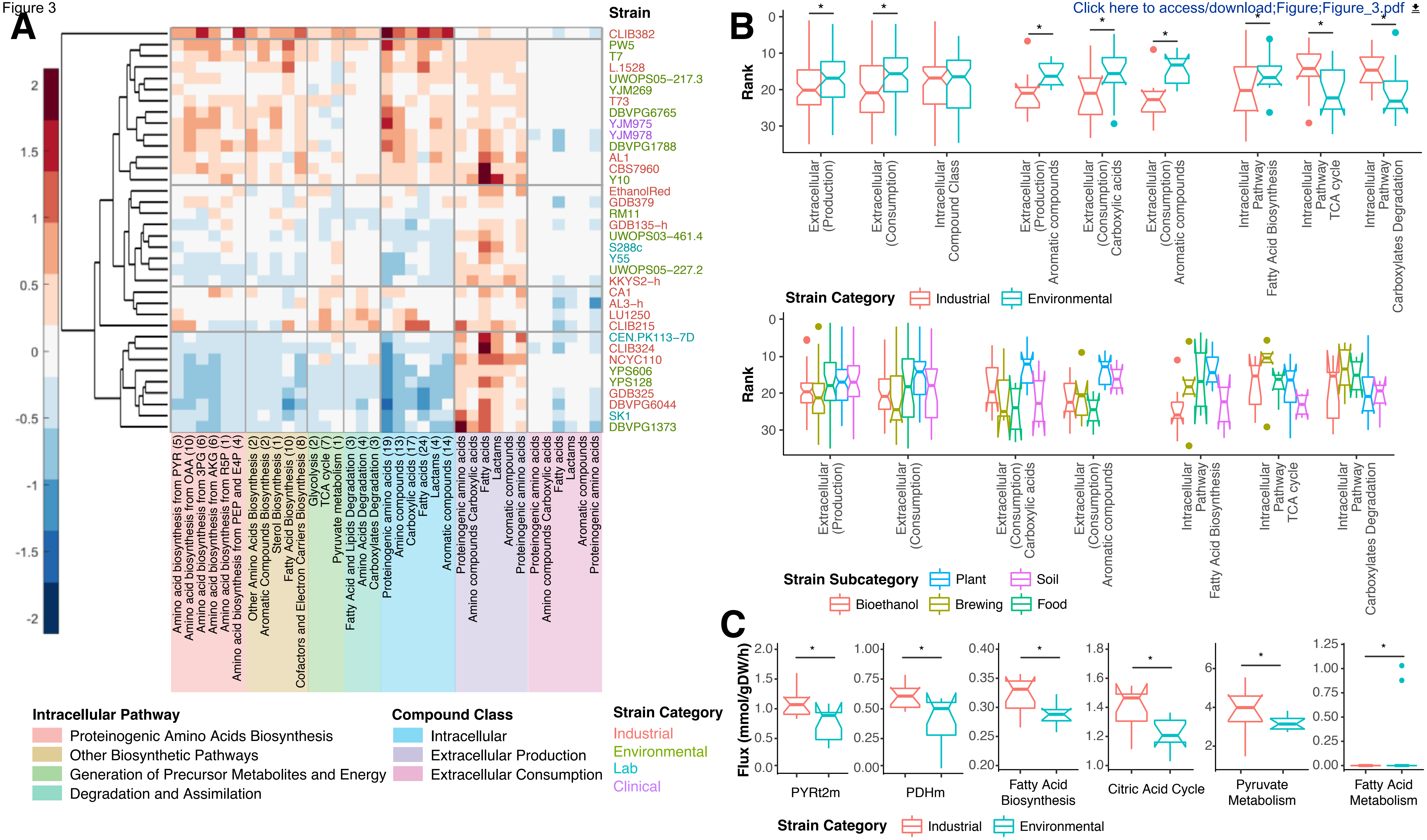

Dataset S1

Click here to access/download
**Supplementary Material**
Dataset_S1.xlsx

Dataset S3

Click here to access/download
**Supplementary Material**
Dataset_S3.xlsx

Dataset S5

Click here to access/download
**Supplementary Material**
Dataset_S5.xlsx

Dataset S6

Click here to access/download
**Supplementary Material**
Dataset_S6.xlsx

Revised Supplemental Information with Track Changes

Click here to access/download
**Supplementary Material**
SC_SI_tracing.docx

Click here to access/download
**Supplementary Material**
SC_SI.docx

Dataset S2

Click here to access/download
**Supplementary Material**
Dataset_S2.xlsx

Rebuttal Letter
Click here to access/download;Personal Cover;SC_rebuttal_letter.pdf

Leibniz Institute for Natural Product Research and Infection Biology
Hans Knöll Institute

Hans-Knöll-Institut | Beutenbergstraße 11a | 07745 Jena

Gianni Panagiotou, PhD

Head of Systems Biology and Bioinformatics
Leibniz Institute for Natural Product Research and Infection Biology
Hans Knöll Institute

+49 3641 532-1759 (T)
+49 3641 532-2759 (F)
gianni.panagiotou@leibniz-hki.de
www.leibniz-hki.de
Jena, Germany

**Visiting address:**
Beutenbergstraße 11a
07745 Jena

**Postal address:**
Adolf-Reichwein-Straße 23
07745 Jena

Honorary Associate Professor
School of Biological Sciences
The University of Hong Kong
gipa@hku.hk
sbb.hku.hk
Hong Kong

Editor-in-Chief
Computational and Structural Biotechnology Journal
Elsevier
gianni.panagiotou@csbj-rncsb.org
www.csbj.org
Gothenburg, Sweden

Dear Dr. Nicole Nogoy,

Thank you very much for the opportunity to revise our manuscript, "Linking genetic, metabolic and phenotypic diversity among *S. cerevisiae* strains using multi-omics associations" for publication in GigaScience. We are pleased to submit the revised version, in which we have addressed all suggestions of the three reviewers, and a point-to-point response to their comments could be found below. We have also adjusted some of our author's name and order, please be noticed.

We are looking forward to hearing from you and we are hoping a positive evaluation.

Sincerely,

Gianni Panagiotou, PhD

**Respond to Editor's note:**

Overall, a better description of the data analysis of metabolome and fluxome is required and reviewer #2 highlighted that some important procedures are missing for others to replicate the experiment. We strongly suggest you add your methods into Protocols.io - and the Protocols.io DOI cited in them manuscript. Note that our journal has a strong focus on reproducible research and we require such information is added for reviewers to adequately replicate your work. Furthermore, the quality of your results is not sufficiently discussed and needs to be improved to convince the reviewers.

We have made all suggested revisions to better describe the metabolome and fluxome analysis and prepared point-to-point responses to all reviewers' questions. Required raw data (**Dataset S7**) and scripts are all available now. However, the submission systems automatically unzip all zip files. We alternatively uploaded **Datasets S4 & S7** into the ftp folder.

Please also ensure that the genomic data associated with the SRA accession number is accessible, and add the MetaboLights accession number in the paper under "Availability of Supporting Data".

We have prepared the standard MetaboLights submission, with accession number: **MTBLS780**. The public date was set to 28/12/2018, which is the most recent date we can choose, as the administrators may manually curate the data. And our SRA submission is open to public now (**SRR6114130** to **SRR6114127**).

In addition, please register any new software application in the SciCrunch.org database to receive a RRID (Research Resource Identification Initiative ID) number, and include this in your manuscript. This will facilitate tracking, reproducibility and re-use of your tool.

We have uploaded two self-developed pipelines to Github and SciCrunch: the metabolomic data processing pipeline, Metab 1.0 (https://github.com/reinkk/Metab, **SCR_016877**) and the growth curve processing pipeline, Growth-Profiling-Toolbox 1.0 (https://github.com/reinkk/Growth-Profiling-Toolbox, **SCR_016878**)

**Answers to reviewers:**

**Reviewer #1:**

---

In this manuscript, the authors did large-scale comparative genomics studies on various yeast species. Based on the growth phenotypes in the presence of different inhibitors or harsh industrial conditions, the authors tried to find the genetic determinants of several industrially desirable phenotypes. Although the topic covered in this manuscript is important for industrial biotechnology, unfortunately, the reviewer is not convinced that the authors have achieved what they claimed. Following please find a few suggestions to improve this manuscript.

---

We would like to thank this reviewer for his/her comments.

---

1. The stress response is precisely controlled by the regulatory networks, which means that transcriptomics may be more reliable than genomics studies for the purpose of this study.

---

Thank you for your suggestion. Transcriptomics would be definitely expand the value of such a multi-omic study. The current study has focused more on the identification of industrial phenotype associated genotypes, thus ,we believe ultra-depth sequencing, in particular covering the genomic variants affecting transcription factor and non-coding regions such as promoters, are in higher priority. To fill the gap in the RNA-level, we performed sufficient work in GWAS followed by protein-protein interaction networks, which has successfully associated the industrial phenotypes with regulatory networks.

---

2. The most important data that the reviewer expect to have is the list of genes associated with the desirable phenotypes. Unfortunately, it is not provided in this manuscript.

---

Due to the length limitation, the regarding gene lists were summarized in **Dataset S6** but not listed in the main text. We have inserted more clear quotations in the main text to guide the reader.

3. All bioinformatics derived data should be verified. Otherwise, such data can be wrong or misleading and may be useless for practical biotechnology applications.

Thank you for your comment. The pipelines we used for the bioinformatic analyses are the state-of-the-art used for population genomics (e.g., BWA for mapping, GATK for SNP/InDel calling, FreeC for CNV calling, EMMA for GWAS) or specifically developed for *S. cerevisiae* (YGAP gene annotation pipeline). The tools and pipelines are also in line with the ones applied in recent publications in the most prestigious journals of the field of yeast population genomics (Liti et al., 2009a; Peter et al., 2018). Thus we are confident that our results are technically reliable and reproducible. Furthermore, our study is a systems level analysis linking genotypes with phenotypes therefore we focused on confirming holistically the accuracy of our findings using extensively public available phenotypic databases (such as SGD) and other bioinformatics approaches (PPI networks). Verifying one association experimentally will not in our opinion add to our study, which serves as a very important source to experimental groups for future metabolic engineering endeavours.

**Reviewer #2:**

The manuscript titled "Linking genetic, metabolic and phenotypic diversity among S.cerevisiae strains using multi-omics association" details the characteristics of various budding yeast strains with multi omics. Authors analyzed phenotypes, metabolome, fluxome and genome of the strains. They demonstrated the possible strategy of strain selection and improvement.

This study includes several useful information, such as widely analyzed stress tolerance and genome-wide association study. However, manuscript of several part should be improved. Especially the description of data analysis of metabolome and fluxome is missing some important procedures for others to replicate the experiment. And quality of result is not sufficiently discussed.

We would like to thank this reviewer for his/her positive comments.

Major point is below.

Line 86-88.

Maximum OD is also important parameter for industrial usage. Please, consider including the parameter in your study. At least, include the maximum OD of each strain under the general condition in this study.

---

Thank you for this valuable comment. The total number of generations was chosen as the measure instead of the final OD as it also takes into account the starting OD which could vary among different experiments, in which the cells were under extreme stress. When coming to industrial production, considering that the initial OD would be stable, then the parameter “number of generations” could be definitely replaced by final OD. Some selected cases were also compared with the conventional procedure which showed that the two methods actually correspond quite well. Actually, we now shared our pipeline in Matlab on Github (https://github.com/reinkk/Growth-Profiling-Toolbox) and SciCrunch (**SCR_016878**), which allows user to modify some simple codes to use max OD as a parameter to replace “number of generations.

---

Line 142-151.

The part of Parameter Influence Analysis makes me confusing. I could not understand why it is discussed here. What the advantage of this analysis. For example, we all realize that lag phase and average growth rate are less important for continuous fermentation without the analysis.

---

Since we know before the analysis that lag phase and average growth rate are less important for continuous fermentation, and that is the intended use of the strain, we do not want to select a strain which ranks high due to these parameters. Therefore, PIA is introduced as a tool to understand in depth which are the parameters driving the phenotypic differences. The main advantage of PIA is that it can distinguish strains with similar rank (when giving equal weight to all five growth parameters) from each other and thereby assist in selecting the strain most suited for a specific application, and therefore allows researchers to adjust parameters’ weights for different industrial purposes. When selecting a platform strain for a continuous fermentation process, low weight could be given to parameters like “lag phase”. In contrast in a batch fermentation, “lag phase” becomes a determinant parameter which can influence the fermentation cycle dramatically; in that case a higher weight could be given to “lag phase”. To make this clearer, we have modified related descriptions in the main text

as: “*Such information can be useful when selecting a host strain for specific process by setting different weights to different growth parameters, e.g. lag phase would be less important selection criteria for a continuous fermentation process and subsequently be given a low weight, however, in a batch fermentation process, lag phase duration becomes critical and could be assigned with a higher weight*”.

---

Line 154-156.

Show the score plot of principal component analysis of metabolome data as supplemental figure to show the data quality of biological and technical replicates. "intracellular" in Dataset S2 can be applied to directly to PCA. Unit variance is probably enough for preprocessing. Also, show the PCA of "extracellular" data.

---

Thank you for this excellent suggestion. We have applied such analyses and place the new figures in **Supplementary Note S3** as **Figures SN 6 & 7**, under a new section named “*Metabolite profile similarity by Principal Component Analysis (PCA) analysis*”.

---

Line 240-254.

Please discuss the reliability of the fluxes calculated in this study.

---

We cited a recent paper used similar simulation technology showing that, strain-specific models can simulate differences in fluxes and give more biological insights than comparing metabolic reactions. So we added “*Due to a recent study (Monk et al., 2016), inter-strain differences could be observed in key metabolic fluxes simulated by strain-specific models, which could provide more meaningful biological insights than the reaction presence/absence comparisons.*”.

---

Line 251-254.

Comparison of flux of limiting steps is probably more suitable to compare the flux of pathways instead using the summed fluxes.

---

We fully agree. So we also presented the fluxes of key limited steps (e.g., Pyruvate Mitochondrial Transport) before the summed fluxes. However, the pathways we listed

here, e.g., Citric Acid Cycle and Pyruvate Metabolism, have non-linear complex network structures which are consisted of multiple parallel reactions. Thus we think the overall flux description may give a different view from the key limiting step.

---

Line 257-316.

Are the sample number and sequence depth enough to perform SNP-based and CNV based GWAS?

---

The genomes were sequenced at a minimal depth of 52X, which is sufficient for SNP and CNV calling and GWAS analysis. Previous studies have also conducted GWAS on a strain cluster with similar number (35 ~ 38) of yeast strains (Connelly et al., 2013; Liti et al., 2009a; Warringer et al., 2011). Also, there is systematic evaluation of GWAS on *S. cerevisiae* strains of such sample size (Diao and Chen, 2012), suggesting that biologically meaningful results could be observed. Also, our PPIN analysis assisted dramatically in the reduction of false-positive noises in the GWAS analysis.

---

Figure 1.

In this study, growth phenotypes are mostly discussed as relative values. However absolute values are also important. Especially the variance of the phenotypic scores under each stress. Please show them as supplemental figures. Box plots may be suitable.

---

Thank you for this suggestion. All raw parameter scores were summarized in Dataset S1. However, considering our data size, we have 36 strains, 13 conditions with 4~7 levels and five parameter scores for each condition. To have sufficient resolution, in total there should be 36 x 13 x 5 = 2,340 box plot panels with 5~7 columns each. So we did not prepare all the figures for raw parameter scores but listed them in tables.

---

Figure 3A. and line 368-375 of supplemental items.

The preprocessing and transformation of HCA is not sufficiently described. Please describe in detail. Because data of MS is relative abundance, this process is quite important. And, what the "scaled to the biological range" mean?

---

The definition of “the biological range” was introduced by a previous literature in the metabolomic data normalization (van den Berg et al., 2006). We now added this citation and modified our description in detail under the section “Compound class and pathway assignment” as: “*Metabolite data was transformed before calculating the contribution to each group of pathways and/or metabolite classes. Both intracellular and extracellular metabolites were scaled to the biological range [5]. Intracellular metabolite data was transformed by subtracting the mean of the data set, followed by division with the difference between the maximum and minimum value of the particular metabolite. This difference is referred to as the biological range and makes all metabolites equally important [5]. Extracellular metabolites were first centered by the starting amount prior to scaling to the biological range, and were grouped according to compound classes as well as if they were produced (scaled value > 0) or consumed (< 0). Metabolites belonging to one of the selected classes and had positive or negative values were added together after scaling, respectively, to show production and consumption. For both intracellular and extracellular metabolites, scaled values of metabolites belonging to a pathway or compound class were added together for each strain to create group variables.The sum was subsequently divided by the square root of the number of metabolites to be comparable with the other classes. Hierarchical clustering was applied to the group variables using Euclidian distance between the strains.*”

---

Figure 3A.

How the compound class determined? And, what do metabolites belong to the classes?

---

To describe this, a new section names “*Compound class and pathway assignment*” was inserted to **Supplemental Note S3**: “*Compounds identified were assigned to different classes and pathways by the pathway information in YMDB (Yeast Metabolic Database) (Jewison et al., 2012). These pathway and compound class assignment were summarized in* ***Dataset S2***.”.

---

Line 309-325 of supplemental items.

I think the metabolome data of ethanol is not mentioned in this study.

---

Thank you for your correction. We did not use the ethanol media so we have removed all regarding descriptions in **Supplemental Note S3** now.

---

Line 379-380 of supplemental items.

In Ref.4 you mentioned, the level of extracellular metabolites used. Your study also uses them? If so, please add the data used to calculate the flux as supplemental material.

---

Ref. 4 refers the starting model of yeast we used for the strain-specific models, which did not actually used extra metabolomic data. The metabolomics data used for our models are already included in **Dataset S2**. And the regarding raw data are now available on MetaboLights with accession number: **MTBLS780**. The public date was set to 28/12/2018, which is the most recent date we can choose.

---

Minor point

Line 97-115.

The stress responses are sometimes affected by growth condition such as aeration condition and medium composition. I recommend describing the conditions briefly in body text, and if possible, discuss about it in discussion.

---

Thank you for this suggestion. The growth medium used in the current study is a standard defined mineral medium extensively used in yeast studies. Aeration was the same in all conditions for phenotypic characterization by using the Growth Profiler. As the size of the study was already considered quite large, it was actually not within the scope of the study to include variation in the growth medium nor the aeration conditions. But sure, expanded studies with altered aeration condition or medium composition will be interesting, following our technical route. Now we added the following statement in the main text: "*Besides parameter selection and weighting, our phenotypic scoring method could also be expanded to different industrially relevant processes with*

*customized set-ups: e.g., different medium composition and aeration condition, which is not limited to the standard medium and condition used in this proof-of-concept study*."

---

line 154 -156.

Please show the level of compound identification based on Metabolomics Standards Initiative (see table1 of Metabolites2018,8,31)

---

We have prepared the standard MetaboLights submission, with accession number: **MTBLS780**. The public date was set to 28/12/2018, which is the most recent date we can choose. And we also included an MAF table (Metabolite Assignment File) in **Dataset S2**, so readers can find ChEBI ids for those metabolites.

---

Line 383-423

If possible, compare the genes you selected with previous study of single gene deletion collection, such as the study below.

(FEMS Yeast Research, Volume 9, Issue 1, 1 February 2009, Pages 32-44)

---

We do observe genes mentioned in the literature to appear as significant in our analysis, thus we added the following sentence -as such an example- in our discussion: "*SKY1 was also a significant gene shared by 4 conditions, which has been proved to be associated with osmotic tolerance (Yoshikawa et al., 2009)*".

---

Figure 3A. In HCA what type of clustering method is used?

---

We added the following statement to the figure 3 legend: "*Hierarchical clustering was applied to the group variables using Euclidian distance between the strains. For data normalization before visualization, see details in **Supplemental Note S3**.*"

---

Figure 3C.

Please add the unit of vertical axis.

---

The unit is mmol/gDW/h and we have now added it to the figure.

**Reviewer #3:**

---

In this study, Kang et al. performed whole genome sequencing, characterized growth under 13 industrially relevant conditions, performed metabolomics and GWAS analyses on 36 S. cerevisiae strains to identify potentially important genes implicated in stress tolerance/ resistance. The rationale for this study is well-defined. While other studies that utilize natural genetic variation between strains have a larger collection of strains across a wide geographic and phylogenetic distribution (e.g. 165 strains in Sardi M et al 2018, PMC5849340), Kang et al. utilizes a multi-OMIC approach to characterize these 36 strains deeply. Additionally, these authors performed a detailed growth characterization of these 36 strains, with thoughtful considerations outlined in the Parameter Influence Analysis. Overall, the paper seems appropriate for GigaScience given its editorial guidelines. Below, I have a few comments that can be addressed with minor revisions to the manuscript.

---

We would like to thank this reviewer for his/her positive comments.

---

Major points:

1. Given the importance of the growth data that defines strain robustness and performance, where will the raw growth data underlying the 5400 growth curves along with the in-house MatLab scripts deposited?

---

We have uploaded two self-developed pipelines to Github and SciCrunch: the metabolomic data processing pipeline, Metab 1.0 (https://github.com/reinkk/Metab, **SCR_016877**) and the growth curve processing pipeline, Growth-Profiling-Toolbox 1.0 (https://github.com/reinkk/Growth-Profiling-Toolbox, **SCR_016878**). And the raw OD data were also included as Dataset S7. The software repositories were now mentioned in the manuscript as "*Raw data for growth curves in G-values (**Dataset S7**) were analyzed automatically using self-developed Matlab® scripts: Growth Profiling Toolbox (GitHub repository: https://github.com/reinkk/Growth-Profiling-Toolbox, with SciCrunch ID **SCR_016878**).*".

2. Along the same line of inquiries, I was not able to access the genomic data associated with the SRA accession number that was provided in the manuscript.

We now made our submission public.

3. Furthermore, while I am not a metabolomics expert, I know that the metabolomics field has developed Metabolomics Standards Initiative to improve reproducibility. It is not clear whether the authors follow this guideline. There were also no accession number associated with the metabolomics data. Have the authors deposited the data to databases such as MetaboLights?

We have prepared the standard MetaboLights submission, with accession number: **MTBLS780**. The public date was set to 28/12/2018, which is the most recent date we can choose. And we also included an MAF table (Metabolite Assignment File) in **Dataset S2**, so readers can find ChEBI ids for those metabolites.

4. The use of base modifications (BMs) to describe genetic variations such as SNPs or INDELs is inappropriate. In general, base modification is used to describe methylation, and as far as I could gather from the methods section, the authors did not perform epigenetic analyses or PacBio sequencing.

We modified it as “SNPs/InDels” throughout the text.

5. Since accurate SNP calling is important for the GWAS, I have a few comments and questions about the choice of these filters in GATK:
a. It's worthwhile to explicit state the filtering strategy (I did not see it in the supplemental file) and had to dig it out by looking up the reference to a previous paper: pubmed/26255308

b. Is there a reason why the authors chose to use UnifiedGenotyper as GATK has deprecated this tool due to this caller being very "aggressive in calling variants in order to be very sensitive"? The current recommendation by GATK is to use HaplotypeCaller that reduces false positives, especially on INDELs.
c. This is related to the concerns of false positive SNPs, where the authors use VariantFiltration (parameters:-filterExpression 'ReadPosRankSum < −8.0 || FS > 10.0'). What is the rationale for lowering the FisherStrand (FS) value below recommendations by GATK? According to GATK, most of the variants that fail have an FS value greater than 55, hence the recommendation of failing variants with an FS value less than 60.

We are sorry that we have made a mistake here and thank you so much for your correction. We checked our script and we indeed used a new pipeline since GATK suggested HaplotypeCaller after 2014, which is different with the cited paper when data were processed before HaplotypeCaller becoming a golden standard. The section has been rewritten now as: "*SNPs/InDels were called and filtered using the Genome Analysis Toolkit (GATK) 3.5 (DePristo et al., 2011; McKenna et al., 2010), with the sequential steps to include RealignerTargetCreator, IndelRealigner, HaplotypeCaller (with parameter "-rf BadCigar") and VariantFiltration (with parameter "--filterExpression "DP < 10 || QD < 2.0 || FS > 60.0"")*".

---

d. The authors cite Strope PK et al (2015) as a support that the numbers of genetic variants they identified in this study is comparable to previous studies (p9, lines 178-180). However, Strope PK observed an average of 78,184 SNPs and 7840 INDELs from 100 genomes, compared to the current study of 342,325 SNPs and 19,347 INDELs. Given the relaxed filter used for variant calling and the 2-4X more variants identified, I think it's important to make sure that these variants are not false positives.

---

*S. cerevisiae* strains are quite divergent in the genome. The reason why Strope PK et al (2015) has a lower number of SNPs/InDels is that the 100 strains they selected are extremely close to the reference strain S288c, and 84 of the 100 strains are exactly in the same clade with S288c in the phylogenetic tree and were called "S288c type". Also, their number indicates the SNP count per strain, while our 342,325 are all SNPs loci identified in the whole strain collection. For each strain, we have 68,928 SNPs/InDels in average, which is quite close to Strope PK et. al (78,184 + 7,840, as they did not applied the strict filtering as we did). Now we cited a more comparable study (similar strain collection but with lower sequencing depth) where they have 235,127 SNPs (Liti et al., 2009b) and modified our description to "*By using S288c as the reference genome, we identified 342,325 SNP loci, 19,347 small insertion and 17,457 small deletion (InDel) loci among all strains (**Table S1** & **Dataset S3**), with an average SNP/InDel count of 68,928 per strain*".

Furthermore, as described in our manuscript, we applied MAF > 5% (at least 4 alleles in our case) before LD block building and GWAS. Also, we selected only core-genome SNPs/InDels for GWAS, which resulted in only 165,358 loci left. We believe these SNPs/InDels are confident for down-stream GWAS.

6. What is the rationale for choosing a low MAF filtration (MAF > 0.5%) for the core-genome GWAS (pg 34, line 483)? What does the MAF distribution look like for these identified core-genome SNPs? In general, the GWAS studies in yeast tend to use MAF > 2% - 25%, as the power to detect association with rare variation is limited.

Sorry for this typo in Note S5 and now we corrected it. Actually we used MAF > 0.05, which is 5% but not 0.5%. We now modified all sections mentioning MAF to the format "5%" to avoid misunderstanding.

Minor points:

1. In Supplemental Note S2, the authors describe how they extract growth parameters of interest in great details. Could the authors add in the rationale for calculating the average growth rate using a time point between 50 to 501 time points (pg 14, line 165)? More specifically, does this time point capture the linear range of the growth curve and time point 50 is after the lag phase of even the most stressed cells?

The time point was selected during exponential phase. So for the most stressed cell, the time point was much later than the cells under less stress. For the least stressed cells, the time point is close to 50, otherwise that would be a much later time point. We added "*to ensure the time point was in exponential phase*" in the relevant description.

2. In Supplemental Dataset S1, the authors need to define the color coding for parameter influence.

We added "*P1~P5 stand for: 1. Lag phase; 2. Growth duration; 3. Number of generations during the growth phase; 4. Average specific growth rate during the growth phase; 5. Maximum specific growth rate.*" to the figure legend.

3. In general, the figures are aesthetically pleasing and well designed. However, it is important to keep in mind of color blindness (e.g. Fig 2A uses red and green colours to denote the strains)

Thank you for your suggestion. We admit that we have not thought about colour blindness and we will pay more attention in the future.

4. Need to properly cite R as per CRAN's guideline https://cran.r-project.org/doc/FAQ/R-FAQ.html#Citing-R

Thank you for pointing this out, the R Core Team has been cited now.

**References**


Connelly, C.F., Skelly, D.A., Dunham, M.J., and Akey, J.M. (2013). Population Genomics and Transcriptional Consequences of Regulatory Motif Variation in Globally Diverse Saccharomyces cerevisiae Strains. Molecular Biology and Evolution *30*, 1605-1613.

DePristo, M.A., Banks, E., Poplin, R., Garimella, K.V., Maguire, J.R., Hartl, C., Philippakis, A.A., del Angel, G., Rivas, M.A., Hanna, M., *et al.* (2011). A framework for variation discovery and genotyping using next-generation DNA sequencing data. Nature genetics *43*, 491-498.

Diao, L., and Chen, K.C. (2012). Local ancestry corrects for population structure in Saccharomyces cerevisiae genome-wide association studies. Genetics *192*, 1503-1511.

Jewison, T., Knox, C., Neveu, V., Djoumbou, Y., Guo, A.C., Lee, J., Liu, P., Mandal, R., Krishnamurthy, R., Sinelnikov, I., *et al.* (2012). YMDB: the Yeast Metabolome Database. Nucleic Acids Res *40*, D815-820.

Liti, G., Carter, D.M., Moses, A.M., Warringer, J., Parts, L., James, S.A., Davey, R.P., Roberts, I.N., Burt, A., Koufopanou, V., *et al.* (2009a). Population genomics of domestic and wild yeasts. Nature *458*, 337-341.

Liti, G., Carter, D.M., Moses, A.M., Warringer, J., Parts, L., James, S.A., Davey, R.P., Roberts, I.N., Burt, A., Koufopanou, V., *et al.* (2009b). Population genomics of domestic and wild yeasts. Nature *458*, 337-341.

McKenna, A., Hanna, M., Banks, E., Sivachenko, A., Cibulskis, K., Kernytsky, A., Garimella, K., Altshuler, D., Gabriel, S., Daly, M., *et al.* (2010). The Genome Analysis Toolkit: a MapReduce framework for analyzing next-generation DNA sequencing data. Genome research *20*, 1297-1303.

Monk, J.M., Koza, A., Campodonico, M.A., Machado, D., Seoane, J.M., Palsson, B.O., Herrgard, M.J., and Feist, A.M. (2016). Multi-omics Quantification of Species Variation of Escherichia coli Links Molecular Features with Strain Phenotypes. Cell Syst *3*, 238-251 e212.

Peter, J., De Chiara, M., Friedrich, A., Yue, J.X., Pflieger, D., Bergstrom, A., Sigwalt, A., Barre, B., Freel, K., Llored, A., *et al.* (2018). Genome evolution across 1,011 Saccharomyces cerevisiae isolates. Nature *556*, 339-344.

van den Berg, R.A., Hoefsloot, H.C., Westerhuis, J.A., Smilde, A.K., and van der Werf, M.J. (2006). Centering, scaling, and transformations: improving the biological information content of metabolomics data. BMC Genomics *7*, 142.

Warringer, J., Zörgö, E., Cubillos, F.A., Zia, A., Gjuvsland, A., Simpson, J.T., Forsmark, A., Durbin, R., Omholt, S.W., Louis, E.J., *et al.* (2011). Trait Variation in Yeast Is Defined by Population History. PLoS Genetics *7*, e1002111.

Yoshikawa, K., Tanaka, T., Furusawa, C., Nagahisa, K., Hirasawa, T., and Shimizu, H. (2009). Comprehensive phenotypic analysis for identification of genes affecting growth under ethanol stress in Saccharomyces cerevisiae. FEMS Yeast Res *9*, 32-44.

Cover Letter
Click here to access/download;Personal Cover;SC_cover_letter.pdf

**Leibniz Institute for Natural Product Research and Infection Biology**
Hans Knöll Institute

Hans-Knöll-Institut | Beutenbergstraße 11a | 07745 Jena

Gianni Panagiotou, PhD

Head of Systems Biology and Bioinformatics
Leibniz Institute for Natural Product Research and Infection Biology
Hans Knöll Institute

+49 3641 532-1759 (T)
+49 3641 532-2759 (F)
gianni.panagiotou@leibniz-hki.de
www.leibniz-hki.de
Jena, Germany

**Visiting address:**
Beutenbergstraße 11a
07745 Jena

**Postal address:**
Adolf-Reichwein-Straße 23
07745 Jena

Associate Professor
School of Biological Sciences
The University of Hong Kong
gipa@hku.hk
sbb.hku.hk
Hong Kong

Editor-in-Chief
Computational and Structural Biotechnology Journal
Elsevier
gianni.panagiotou@csbj-rncsb.org
www.csbj.org
Gothenburg, Sweden

Dear Editor,

We are pleased to submit the enclosed paper entitled "Linking genetic, metabolic and phenotypic diversity among *S. cerevisiae* strains using multi-omics associations" for publication in the journal *GigaScience*. In the present work, we investigated the genetic and phenotypic diversity of 36 industrial, environmental, clinical and laboratory *S. cerevisiae* strains, by an integrative study covering genome, metabolome, fluxome, phenome and interactome focusing on the resistance against 13 industrially relevant stress conditions. In total, we analyzed more than 5400 growth curves, five growth parameters were extracted and used to score the strains in two phenotypic scores: Robustness and Performance. Differences in the genetic makeups and stress resistances between industrial and environmental strains were revealed (e.g., high duplication rate and high heterozygosity rate were associated with higher acidic condition resistance for industrial strains), and the differentiated evolutionary paths and stress-response strategies were discussed. Gene-to-phenotype links were established by the SNP-based and CNV-based genome-wide association studies (GWAS). Finally, new platform strains with multiple stress resistances (e.g., Y55, RM11 and PW5) and novel potential engineering targets were suggested; for instance, the duplication events found in AL1, CLIB215 and GDB325, the CNV regions YHR218W-YHR219, YNR059W-YNR062C and the transcription factors Spt15, Ecm22 and Bur6 revealed by GWAS, and the *FRE*/*FIT*, *COX*/*ATP* and *SOR*/*HKX* modules discovered in the protein-protein interaction network (PPIN) analysis.

Previous large-scale genomics investigations have revealed the genetic diversity of *S. cerevisiae*, however, the differences between natural and industrial strains and the respective associations to the industrial values were missing. On the other hand, traditional bioengineering endeavors have also neglected the genetic diversity of yeast populations, and were in lack of global screening technics for engineering targets. In our work, the multi-omics integrative strategy was successful for both scientific questions and engineering purposes. Moreover, the innovative technical methods, including the resistance score calculation, the core genome SNP-based GWAS and CNV-based GWAS, and the noise-reducing PPIN module discovery, are also applicable to further geno- and phenotyping studies.

We would like to suggest the following experts in the fields of industrial microbiology and yeast systems and synthetic biology as the potential reviewers of our manuscript: Prof. Jens Nilsen (Chalmers University of Technology), Prof. Yingjin Yuan (Tianjin University) and Prof. Huanming Yang (BGI-Shenzhen).

The materials and data are original in this study, have not been previously published and have not been submitted for other publications. The authors declare no conflict of interest. We, therefore, hope and look forward to the opportunity for our work to be peer-reviewed, and should any questions or concerns arise, please do not hesitate to contact us.

Sincerely,

Gianni Panagiotou, PhD
