# Supplementary material for: Linking genetic, metabolic, and phenotypic diversity among Saccharomyces cerevisiae strains using multi-omics associations
Source: Gigascience. 2019 Jan 31;8(4):giz015. doi: 10.1093/gigascience/giz015 (PMC6446221; doi:10.1093/gigascience/giz015)
Supplement: Supplemental Files [file giz015_supplemental_files.zip › SC_SI.docx]

# Linking genetic, metabolic and phenotypic diversity among *S. cerevisiae* strains using multi-omics associations

Kang Kang^1,6†^, Basti Bergdahl^2†^, Daniel Machado^3,4^, Laura Dato^2^, Ting-Li Han^5^, Jun Li^1,6^, Silas Villas-Boas^5^, Markus J. Herrgård^2*^, Jochen Förster^2*,#^ and Gianni Panagiotou^1,6*^

* Corresponding author: [herrgard@biosustain.dtu.dk](mailto:herrgard@biosustain.dtu.dk) , [Jochen.Forster@carlsberg.com](mailto:Jochen.Forster@carlsberg.com) , [Gianni.Panagiotou@hki-jena.de](mailto:Gianni.Panagiotou@hki-jena.de)

**SUPPLEMENTAL ITEMS**

This article has 13 supplemental items, including the supplemental notes (including 5 sections), 2 tables, 4 figures and 6 datasets in individual documents.

**Supplemental Tables**

**Table S1. The variant statistics.**

| **Strain** | **SNP/InDel (k)** | **Heterozygosity (%)** | **InDel Rate (%)** | **Gain event / Gene** | **Loss event / Gene** | **Deletion event / Gene** | **non-S288c Gene** |
| --- | --- | --- | --- | --- | --- | --- | --- |
| AL1 | 74.51 | 42.1 | 8.36 | 6 / 61 | 4 / 45 | 0 / 0 | 18 |
| AL3-h | 64.39 | 8.65 | 8.32 | 5 / 76 | 5 / 57 | 2 / 42 | 16 |
| CA1 | 61.38 | 8.56 | 9.2 | 9 / 54 | 22 / 196 | 3 / 46 | 20 |
| CBS7960 | 62.65 | 9.91 | 8.54 | 4 / 49 | 21 / 187 | 1 / 11 | 18 |
| CEN.PK113-7D | 25.1 | 0 | 8.02 | 5 / 39 | 0 / 0 | 3 / 25 | 8 |
| CLIB215 | 53.15 | 6.04 | 9.29 | 7 / 460 | 18 / 184 | 3 / 43 | 9 |
| CLIB324 | 57.57 | 97.93 | 7.99 | 5 / 194 | 3 / 39 | 3 / 51 | 15 |
| CLIB382 | 60.75 | 8.45 | 8.81 | 3 / 31 | 21 / 183 | 4 / 16 | 25 |
| DBVPG1373 | 54.25 | 6.58 | 9.29 | 4 / 40 | 20 / 99 | 14 / 55 | 13 |
| DBVPG1788 | 90.55 | 5.04 | 7.75 | 6 / 193 | 10 / 222 | 0 / 0 | 15 |
| DBVPG6044 | 91.97 | 6.48 | 7.87 | 12 / 429 | 24 / 250 | 2 / 35 | 15 |
| DBVPG6765 | 53.44 | 6.49 | 9.22 | 4 / 132 | 18 / 180 | 3 / 65 | 22 |
| Ethanol Red | 78.01 | 14.41 | 8.06 | 6 / 59 | 13 / 195 | 0 / 0 | 14 |
| GDB135-h | 66.92 | 98.25 | 7.8 | 7 / 276 | 2 / 13 | 2 / 40 | 18 |
| GDB325 | 85.45 | 52.41 | 8.36 | 8 / 304 | 6 / 100 | 0 / 0 | 20 |
| GDB379 | 80.25 | 45.79 | 8.65 | 1 / 9 | 3 / 33 | 0 / 0 | 15 |
| KKYS2-h | 74.98 | 5.76 | 8.35 | 6 / 59 | 11 / 139 | 1 / 9 | 9 |
| L.1528 | 54.51 | 6.89 | 9.06 | 3 / 34 | 19 / 155 | 6 / 48 | 16 |
| LU1250 | 60.81 | 9.45 | 7.71 | 1 / 17 | 2 / 55 | 0 / 0 | 17 |
| NCYC110 | 90.44 | 5.14 | 7.78 | 4 / 333 | 8 / 93 | 4 / 65 | 17 |
| PW5 | 91.71 | 6.25 | 7.7 | 7 / 257 | 10 / 247 | 0 / 0 | 14 |
| RM11 | 52.9 | 6.12 | 9.15 | 3 / 22 | 10 / 154 | 0 / 0 | 18 |
| S288c | 0.28 | 0 | 34.51 | NA | NA | NA | 3 |
| SK1 | 86.54 | 7.28 | 8.06 | 0 / 0 | 32 / 843 | 5 / 100 | 19 |
| T7 | 78.45 | 8.04 | 8.33 | 7 / 124 | 22 / 150 | 8 / 30 | 23 |
| T73 | 59.47 | 97.85 | 7.97 | 6 / 58 | 1 / 19 | 0 / 0 | 9 |
| UWOPS03-461.4 | 86.89 | 7.26 | 8.01 | 3 / 44 | 10 / 69 | 3 / 55 | 20 |
| UWOPS05-217.3 | 93.7 | 4.14 | 7.63 | 1 / 4 | 1 / 10 | 0 / 0 | 23 |
| UWOPS05-227.2 | 95.12 | 4.33 | 7.91 | 6 / 166 | 15 / 173 | 1 / 0 | 24 |
| Y10 | 75.83 | 0 | 8.13 | 4 / 62 | 0 / 0 | 2 / 32 | 14 |
| Y55 | 76.93 | 7.29 | 8.17 | 2 / 25 | 14 / 132 | 4 / 52 | 15 |
| YJM269 | 79.39 | 8.58 | 8.29 | 7 / 104 | 3 / 60 | 0 / 0 | 11 |
| YJM975 | 53.68 | 6.23 | 9.32 | 5 / 28 | 13 / 141 | 2 / 10 | 7 |
| YJM978 | 53.34 | 6.35 | 9.16 | 1 / 48 | 22 / 1052 | 3 / 96 | 7 |
| YPS128 | 77.88 | 8.29 | 8.38 | 1 / 7 | 14 / 150 | 4 / 51 | 16 |
| YPS606 | 78.22 | 8.7 | 8.38 | 3 / 36 | 19 / 198 | 4 / 46 | 19 |
|  |  |  |  |  |  |  |  |

**Table S2. The GWAS profile statistics on significant genes.**

| **Item** | **Score** | **Condition** | **SNP** | **Gain** | **Loss** | **CNV *** | **SNP and CNV** | **SNP or CNV** |
| --- | --- | --- | --- | --- | --- | --- | --- | --- |
| pH | Robustness | Acidic | 0 | 184 | 53 | 237 | 0 | 237 |
| Temperature | Robustness | Non-acidic | 19 | 23 | 58 | 81 | 0 | 100 |
| Ethanol | Robustness | Non-acidic | 2 | 6 | 31 | 37 | 0 | 39 |
| 1,4-Butanediol | Robustness | Non-acidic | 4 | 30 | 40 | 70 | 0 | 74 |
| D-Limonene | Robustness | Non-acidic | 0 | 3 | 50 | 53 | 0 | 53 |
| 4-Aminobenzoic acid | Robustness | Acidic | 4 | 4 | 69 | 73 | 0 | 77 |
| Acetic acid | Robustness | Acidic | 3 | 24 | 118 | 142 | 0 | 145 |
| Formic acid | Robustness | Acidic | 7 | 3 | 26 | 29 | 0 | 36 |
| Fumaric acid | Robustness | Acidic | 0 | 25 | 52 | 77 | 0 | 77 |
| Pyruvic acid | Robustness | Acidic | 37 | 13 | 28 | 41 | 0 | 78 |
| Ferulic acid | Robustness | Acidic | 22 | 5 | 76 | 78 | 0 | 100 |
| Succinic acid | Robustness | Acidic | 0 | 4 | 71 | 75 | 0 | 75 |
| Furfural | Robustness | Acidic | 2 | 102 | 56 | 143 | 0 | 145 |
| pH | Performance | Acidic | 24 | 30 | 18 | 48 | 0 | 72 |
| Temperature | Performance | Non-acidic | 17 | 20 | 49 | 69 | 0 | 86 |
| Ethanol | Performance | Non-acidic | 0 | 9 | 65 | 74 | 0 | 74 |
| 1,4-Butanediol | Performance | Non-acidic | 4 | 8 | 52 | 60 | 0 | 64 |
| D-Limonene | Performance | Non-acidic | 0 | 21 | 46 | 67 | 0 | 67 |
| 4-Aminobenzoic acid | Performance | Acidic | 0 | 4 | 69 | 73 | 0 | 73 |
| Acetic acid | Performance | Acidic | 0 | 21 | 53 | 74 | 0 | 74 |
| Formic acid | Performance | Acidic | 10 | 28 | 74 | 94 | 0 | 104 |
| Fumaric acid | Performance | Acidic | 25 | 3 | 57 | 57 | 0 | 82 |
| Pyruvic acid | Performance | Acidic | 11 | 0 | 22 | 22 | 0 | 33 |
| Ferulic acid | Performance | Acidic | 0 | 12 | 29 | 41 | 0 | 41 |
| Succinic acid | Performance | Acidic | 0 | 20 | 61 | 81 | 0 | 81 |
| Furfural | Performance | Acidic | 0 | 28 | 49 | 77 | 0 | 77 |
| pH | Resistance ** | Acidic | 24 | 214 | 70 | 284 | 0 | 307 |
| Temperature | Resistance | Non-acidic | 24 | 24 | 69 | 93 | 0 | 117 |
| Ethanol | Resistance | Non-acidic | 2 | 11 | 72 | 83 | 0 | 85 |
| 1,4-Butanediol | Resistance | Non-acidic | 8 | 38 | 92 | 130 | 0 | 138 |
| D-Limonene | Resistance | Non-acidic | 0 | 24 | 60 | 84 | 0 | 84 |
| 4-Aminobenzoic acid | Resistance | Acidic | 4 | 4 | 99 | 103 | 0 | 107 |
| Acetic acid | Resistance | Acidic | 3 | 30 | 138 | 168 | 0 | 171 |
| Formic acid | Resistance | Acidic | 16 | 29 | 88 | 109 | 0 | 125 |
| Fumaric acid | Resistance | Acidic | 25 | 28 | 106 | 131 | 0 | 155 |
| Pyruvic acid | Resistance | Acidic | 48 | 13 | 50 | 63 | 0 | 111 |
| Ferulic acid | Resistance | Acidic | 22 | 12 | 91 | 100 | 0 | 122 |
| Succinic acid | Resistance | Acidic | 0 | 24 | 130 | 154 | 0 | 154 |
| Furfural | Resistance | Acidic | 2 | 121 | 94 | 200 | 0 | 202 |
| * CNV: Genes significant with either gain or loss events. | | | | | | | | |
| ** Resistance: Genes significant for either Robustness or Performance. | | | | | | | | |

**Supplemental Figures**

**Figure S1.** **The Performance and Robustness ranking values in all conditions for all strains.**

**Figure S2.** **The parameter sensitivity analysis of the strain Y55.** (**A**) Sensitivity of the ranking position of strain Y55 regarding each growth parameter. Positive and negative Parameter Influence (PI) scores indicate an increase or decrease in ranking position, respectively, when the corresponding parameter is given more weight in the Rank Variability Analysis (RVA) (**Supplemental Note S2**). PI scores are normalized to be in the range -1 to 1, representing the extreme scores among all parameters and conditions. (**B**) Significant negative correlations (*P* value ≤ 0.05) on PI scores between Y55 and other strains. Larger markers indicate higher probability to fall in the same ranking range.

**Figure S3.** **The correlations among phenotypic rankings including the resistance scores and the metabolomic features.** Spearman’s correlation coefficients were shown in heat map: red color for a positive correlation and blue for a negative correlation. Color bars were used to distinguish the acidic or non-acidic inhibitory conditions class and the intra- or extracellular metabolome. Label colors represent the Robustness or Performance score for the resistance rankings, different pathway or compound class for intracellular metabolome, or production or consumption for extracellular metabolome.

**Figure S4.** **The CNV region distribution for 36 strains (A) and the LD-blocks and CNV regions used for GWAS (B).** **(A)** Gain, loss and deletion events were marked in different colors. Each layer stands for an individual strain (excluding S288c). (**B**) The distributions of significant genes and their variant sources. The green layer illustrates the core genome regions used by the SNP-based GWAS (Core-GWAS, **Supplemental Note S5**), while the red layer indicates the CNV regions. The inner layers represent the significant genes for all rankings, Robustness against acidic conditions, Performance under acidic conditions, Robustness against non-acidic conditions and Performance under non-acidic conditions, respectively. Different colors indicate different variant sources: pink markers were captured in both gain and loss events, while brown marks were significant in both SNP-based GWAS and CNV-based GWAS.

**Supplemental Notes**

**Supplemental Note S1. Strain collection and the stress conditions.**

**Strain collection used in this study**

**Table SN1. The strain collection used in this study.**

| **Strain** | **Type** | **Isolation source** | **Geographic origin** |
| --- | --- | --- | --- |
| AL1 | Industrial | Ethanol production | Brazil |
| AL3-h | Industrial | Ethanol production | Brazil |
| CA1 | Industrial | Ethanol production | Brazil |
| CBS7960 | Industrial | Ethanol production | Brazil |
| CEN.PK113-7D | Laboratory | Derived from parental strains ENY.WA-1A and MC996A | Germany |
| CLIB215 | Industrial | Bakery | New Zealand |
| CLIB324 | Industrial | Bakery | Saigon, Viet Nam |
| CLIB382 | Industrial | Beer brewing | Ireland |
| DBVPG1373 | Environmental | Soil | the Netherlands |
| DBVPG1788 | Environmental | Soil | Turku, Finland |
| DBVPG6044 | Industrial | Bili wine, from *Osbeckia grandiflora* | West Africa |
| DBVPG6765 | Environmental | Lici fruit | Indonesia |
| Ethanol Red | Industrial | Ethanol production | France |
| GDB 135-h | Industrial | Ethanol production | Brazil |
| GDB 325 | Industrial | Ethanol production | Brazil |
| GDB 379 | Industrial | Ethanol production | Brazil |
| KK:YS2-h | Industrial | Finger millet fermentation | Northern India |
| L.1528 | Industrial | Fermentation of Cabernet must | Cauquenes, Chile |
| LUI250 | Industrial | Rice wine | Viet Nam |
| NCYC110 | Industrial | Ginger beer from *Zingiber officinale* | West Africa |
| PW5 | Environmental | Raphia palm | Aba, Abia state, Nigeria |
| RM11 | Environmental | Grape | USA |
| S288c | Laboratory | Rotting fig | USA |
| SK1 | Laboratory | Soil | USA |
| T7 | Environmental | Oak tree | Babler State Park, Missouri, USA |
| T73 | Industrial | Wine fermentation | Spain |
| UWOPS03-461.4 | Environmental | Nectar, Bertram palm | Telok Senangin, Malaysia |
| UWOPS05-217.3 | Environmental | Nectar, Bertram palm | Telok Senangin, Malaysia |
| UWOPS05-227.2 | Environmental | *Trigona spp.* (Stingless bee) collected near Bertam palm flower | Telok Senangin, Malaysia |
| Y10 | Environmental | Coconut | Philippines |
| Y55 | Laboratory | Grape | France |
| YJM269 | Environmental | Grape | Austria |
| YJM975 | Clinical | Vaginal isolate | Bergamo, Italy |
| YJM978 | Clinical | Vaginal isolate | Bergamo, Italy |
| YPS128 | Environmental | Soil beneath *Quercus alba* | Pennsylvania, USA |
| YPS606 | Environmental | Bark of *Quercus rubra* | Pennsylvania, USA |

**The Brazilian bioethanol strains used in this study**

Seven industrial strains were collected as Brazilian bioethanol strains. To avoid these strains may have close genetic relationships which could bias the strain collection, these strains were selected from different sources with distinct origins and genotypic categories [1] (**Table SN2**). Although most of these strains could be found in one major phylogenetic cluster (**Figure 4A**), suggesting the possibility that some of these strains may share common ancestors, we could also observe the long pair-wise genetic distances among the strains (**Figure 4A**), indicating that high divergences in their strain-specific genetic makeups and evolutionary paths could be observed. Also, these strains also have diverse physiological features when cultivated under different conditions (e.g. different carbon sources including glucose and ethanol) (**Table SN2**). Thus, the selected Brazilian bioethanol strains are representing extensive diversity genetically and phenotypically.

**Table SN2. The selection of Brazilian Bioethanol strains and their physiological data.**

| **Strain ID** | **Glucose** | | |  | **Ethanol** | | | **Collection Notes** |
| --- | --- | --- | --- | --- | --- | --- | --- | --- |
|  | **Lag phase (Hr)** | **Max OD** | **Max Growth Rate (Hr^-1^)** |  | **Lag phase (Hr)** | **Max OD** | **Max Growth Rate (Hr^-1^)** |  |
| AL1 | 16 | 6.5 | 0.24 |  | 60 | 22.3 | 0.19 | Brazilian bioethanol strain obtained from Rosane Schwan, Federal University of Lavras, Brazil |
| AL3-h | 6 | 15.6 | 0.27 |  | 150 | 11,0 | 0.06 | Homozygous isolate derived from AL3, Brazilian bioethanol strain obtained from Rosane Schwan, Federal University of Lavras, Brazil |
| CA1 | 6 | 15.8 | 0.39 |  | 50 | 22.1 | 0.15 | Brazilian cachaca strain obtained from Rosane Schwan, Federal University of Lavras, Brazil |
| CBS7960 | 5 | 7.8 | 0.39 |  | 93 | 17.5 | 0. 10 | Isolated from in a factory in Sao Paulo, Brazil and obtained from Dr. Justin Fay, Washington University School of Medicine. Produces ethanol from cane-sugar syrup. |
| GDB 135-h | 6 | 13.1 | 0.36 |  | 20 | 19.9 | 0.19 | Obtained from Marcos Morais, LIKA, Recife, Brazil; P6 genotype [1], dominant in molasses refineries. |
| GDB 325 | 16 | 9.7 | 0.24 |  | NA | NA | NA | Obtained from Marcos Morais, LIKA, Recife, Brazil; P25 genotype [1], found mostly in molasses refineries. |
| GDB 379 | 5 | 12.0 | 0.48 |  | 50 | 20.6 | 0.16 | Obtained from Marcos Morais, LIKA, Recife, Brazil; P1 genotype [1], prominent in sugar-cane refineries. |

**Strain binning for comparisons**

In the comparisons between industrial and environmental strains, to avoid the potential bias induced by the strains sharing the same geographical and functional origins, the seven Brazilian bioethanol strains were binned as one strain (ID: Brazilian Bioethanol), and the physiological value (e.g., the Robustness rank) and genetic value (e.g., the heterozygosity rate) were replaced with the median values of the seven strains. Only the comparison results significant for both binned strain collection (11 industrial vs. 12 environmental strains) and the original strain collection (unbinned, 17 industrial vs. 12 environmental strains) were retrained.

In the sub-categories comparisons (bioethanol, brewing, food, plant and soil), the lab strains Y55 and SK11 which were initially isolated from plant and soil respectively, were categorized as plant or soil strain.

**Supplemental Note S2. Physiological traits screening.**

**Definitions of physiological traits**

In comparison with all strains:

Inhibitory level-specific Performance – A strain with high level-specific Performance has better values of the growth parameters (e.g. higher max growth rate, average growth rate and shorter lag phase) than other strains at a specific inhibitory level.

Performance – A strain with high Performance can perform better than other strains in all or most of the inhibitory levels (mean of all inhibitory level-specific Performance scores).

In comparison with one strain’s reference values (physiological scores in inhibitory level 1):

Inhibitory level-specific Robustness – A strain with high level-specific Robustness maintains or improves its own growth parameters in a specific inhibitory level relative to reference values.

Robustness – A strain with high Robustness can grow and maintain its own growth parameters (relative to reference values) in all or most of the inhibitory levels.

**Media for cultivation under various stress conditions**

The media used for cultivation under stress conditions contained the following concentrations of inorganic ions: 50 mM K^+^; 100 mM NH_4_^+^, 52 mM SO_4_^2-^, 50 mM PO_4_^2-^, 2.03 mM Mg^2+^, and 50 mM of appropriate buffer. The salts used to obtain these concentrations varied depending on the buffer added: medium at pH 3 contained 6.6 g/L (NH_4_)_2_SO_4_, 6.8 g/L KH_2_PO_4_, 0.5 g/L MgSO_4_•7H_2_O and 7.5 g/L Tartaric acid (pK_a_ 2.98); medium at pH 4.5 contained 7.1 g/L Na_2_SO_4_, 6.8 g/L KH_2_PO_4_, 0.5 g/L MgSO_4_•7H_2_O and 4.6 g/L Ammonium tartrate dibasic (pK_a_ 4.25); medium at pH 5.5 contained 6.6 g/L (NH_4_)_2_SO_4_, 6.0 g/L NaH_2_PO_4_, 0.5 g/L MgSO_4_•7H_2_O and 5.1 g/L Potassium hydrogen phthalate (pK_a_ 5.4); medium at pH 7 contained 6.6 g/L (NH_4_)_2_SO_4_, 6.8 g/L KH_2_PO_4_, 0.5 g/L MgSO_4_•7H_2_O and 5.2 g/L BES (pK_a_ 7.1). Every medium contained 20 g/L glucose, 2 mL/L trace element solution and 1 mL/L vitamin solution. The growth of yeast strains was investigated in the presence of the following compounds at pH 5.5: Ethanol (11.4, 25, 45, 65, 78.6 g/L), 1,4-Butanediol (53.2, 60, 70, 80, 86.8 g/L), D-Limonene (4.9, 9, 15, 21, 25.1 g/L), 4-Aminobenzoic acid (PABA; 0.08, 0.25, 0.50, 0.75, 0.92 g/L); and at pH 3: Acetic acid (1.3, 2, 3, 4, 4.7 g/L), Formic acid (0.16, 0.30, 0.50, 0.70 g/L), Fumaric acid (0.48, 1.5, 3, 4.5, 5.2 g/L), Pyruvic acid (13.2, 20, 30, 40, 46.8 g/L), Ferulic acid (0.06, 0.19, 0.38, 0.57, 0.70 g/L), Succinic acid (13.2, 20, 30, 40, 46.8 g/L), Furfural (0.4, 0.83, 1.25, 2.5, 3.75, 4.6 g/L). A concentrated stock solution of each medium was prepared fresh the day before usage and the pH was adjusted using either NaOH or tartaric acid. The stock solution was filter sterilized and diluted using appropriate salt/buffer/glucose solution to yield different concentrations in the ranges indicated above.

**Cultivation in the Growth Profiler 1152**

Yeast strains were pre-cultivated as described above and harvested by centrifugation of the 24-deepwell microplates at 4°C and 4600 rpm for 10 min using a swing-out rotor. Spent medium was removed and cells were resuspended in 500-900 µL sterile water. Samples of the cell suspensions were diluted 10- and 20-fold and the OD values were measured in a 96-well microtiter plate using a Synergy H1 microplate reader (BioTek Instruments Inc., Winooski, VT, USA) at 600 nm wavelength. Each strain was then individually diluted in a 2 mL microtube to an OD = 4.5 (equivalent to a 1 cm light path length) and a volume of at least 1 mL. One cell suspension at a time was vortexed and poured into a liquid reservoir; using a multichannel pipette, 20 µl was inoculated in 6 wells on a 96-squarewell microplate (CR1496d, Enzyscreen, the Netherlands) containing 280 µL of medium, resulting in a starting OD of 0.3. The inoculated plates (up to 12 96-well microplates containing 912 experimental conditions) were then placed in the Growth Profiler 1152 and growth was monitored for ca. 66 hours. Evaluation of growth at temperatures 36°C, 38°C, 40°C, 41°C and 42°C was performed using 24-roundwell microplates (CR1424f, Enzyscreen, the Netherlands) containing 700 µL of medium at pH 5.5. These plates were inoculated with 50 µL cell suspensions at OD = 4.5, resulting in a starting OD of 0.3. Experiments at elevated temperatures were performed in biological duplicates and all other cultivations in at least three biological replicates (inoculated on different days with new starter cultures).

**Processing of data generated by the Growth Profiler 1152**

The green pixel values (G-values) indicating the biomass obtained from the Growth Profiler 1152 were converted to OD-equivalents prior to feature extraction from the growth curves. The calibration curve was generated by measuring the G-values of 24 cell suspensions with specific OD-values in the range from 0.2 to 95. These cell suspensions were obtained by concentrating the cells from eight cultures of yeast strain CEN-PK113-7D grown over-night in 25 mL YPD medium using 250 mL shake flask at 30°C and 280 rpm, and subsequently diluted appropriately to obtain the desired OD-values. The cell suspensions were used to fill the wells of both a 24-roundwell and a 96-squarewell microplate and the G-values were determined in all tray positions in the machine for both plate types. The G-values were averaged for each scanner and the SLM (Shape Language Model) toolbox for Matlab^®^ was used to fit spline models to the data (one for each scanner and plate type). These models were used as the calibration curves for all experiments.

**Extraction of features from growth curves**

Raw data for growth curves in G-values (**Dataset S7**) were analyzed automatically using self-developed Matlab® scripts: Growth Profiling Toolbox (GitHub repository: <https://github.com/reinkk/Growth-Profiling-Toolbox>, with SciCrunch ID SCR_016878). From each growth curve, five growth parameters were determined:

1. Lag phase (defined as the time required to reach 25% of total number of generations). When using the common definition of lag phase (the intercept of the start value (in OD) with the extrapolated steepest slope during the growth phase), the approach did not work well for the growth curves derived from very stressed cells as observed in our study. Thus the chosen definition was used to facilitate the automatic analysis of the growth curves. The total number of generations was chosen as the measure instead of the final OD as it also takes into account the starting OD which could vary between the different experiments;
2. Growth duration (defined as the time it takes to go from 25% to 100% of the total number of generations). The end of growth phase was defined at which the cells could no longer sustain an exponential growth;
3. Number of generations during the growth phase (defined as (ln(OD_t = end_) - ln(OD_t = lag phase_)/ln(2), where t = end is the end of the growth phase);
4. Average specific growth rate during the growth phase;
5. Maximum specific growth rate.

Features were identified using spline models fitted to both linear and log-transformed data using the SLM toolbox. The average growth rate was calculated from the simulated data generated by the fitted spline model which was evaluated at 501 time points; and the average growth rate was calculated using a time point between 50 to 501 time points depending on the growth characteristics, to ensure the time point was in exponential phase. The maximum specific growth rate was calculated by linear regression of at least four log-transformed data points. The outcome of the automatic feature detection was verified manually for each growth curve to ensure consistent results between biological replicates, or the parameters of the fitting algorithm could be modified. Outliers were detected using a Hampel Filter and excluded from the data before analysis. The five growth parameters extracted from the growth curves were used to score the strains according to the two physiological traits: Performance and Robustness.

The five parameters are not totally independent. As a proof-of-concept study, we kept all these parameters as researchers could adjust the weights according the intended purpose and process. For instance, the number of generations is associated with the average growth rate and the growth duration. While two strains can reach the same number of generations (i.e. biomass produced) by using two different strategies, i) slow average growth for a long time or ii) high average growth during shorter time. For different fermentation purposes (i.e. high final biomass or quick biomass production), high weights could be given to different parameters.

**Calculation of scores in the physiological traits**

The starting point for the calculation is the 3d data matrix containing the mean of the five growth parameter values extracted from replicate experiments. The three dimensions are: 5 growth parameters, 4~7 inhibitory levels, and 36 strains. Raw parameter scores are calculated from the growth curves and rescaled to a value between 0 and 20, where the best value will be 20 and bad values will be close to zero (non-growing strains are given a score of zero in all growth parameters). When calculating Robustness, the raw parameter scores will be rescaled over the 4~7 inhibitory levels. For example, for strain AL1, the Lag Phase score in level 1 is the best, and strain is not growing in level 6, then the Lag Phase score for levels 1 and 6 will be rescaled to 20 and 0, respectively. By summing up the rescaled five parameter scores in each inhibitory level, the level-specific Robustness score is obtained. After calculating the means of the scores in all inhibitory levels and the Rank Variability Analysis (RVA), the Robustness score and ranking for the individual strain will be obtained for each specific stress condition. When calculating Performance, the raw parameter scores will be rescaled over the 36 strains, instead. The best and worst raw scores among all strains will be rescaled to 20 and 0, respectively. By summing up the rescaled five parameter scores in each inhibitory level, the level-specific Performance score is obtained. After calculating the means of the scores in all inhibitory levels and the Rank Variability Analysis (RVA), the Performance score and ranking for the individual strain will be obtained for the specific stress condition. The normalized strain rankings were summarized for Robustness **(Table SN3)** and Performance **(Table SN4)** respectively.

**Table SN3. The normalized Robustness rankings.**

| **Strain** | **pH** | **Temperature** | **Ethanol** | **1,4-Butanediol** | **D-Limonene** | **4-Amino-benzoic acid** | **Acetic acid** | **Formic acid** | **Fumaric acid** | **Pyruvic acid** | **Ferulic acid** | **Succinic acid** | **Furfural** |
| --- | --- | --- | --- | --- | --- | --- | --- | --- | --- | --- | --- | --- | --- |
| AL1 | 14.0 | 6.5 | 11.5 | 14.5 | 29.5 | 3.0 | 14.5 | 5.5 | 17.0 | 30.5 | 1.0 | 10.5 | 14.5 |
| AL3-h | 30.0 | 9.0 | 13.5 | 8.0 | 29.5 | 11.0 | 19.5 | 24.5 | 28.0 | 17.0 | 34.5 | 27.5 | 5.5 |
| CA1 | 9.0 | 20.0 | 36.0 | 16.5 | 19.5 | 10.0 | 4.5 | 4.0 | 8.0 | 27.0 | 21.0 | 17.0 | 1.5 |
| CBS7960 | 10.0 | 22.5 | 7.5 | 10.5 | 6.5 | 14.0 | 16.0 | 11.5 | 26.0 | 15.5 | 8.5 | 25.5 | 4.0 |
| CEN.PK 113-7D | 22.0 | 11.0 | 8.5 | 31.0 | 32.0 | 12.5 | 13.0 | 26.0 | 5.5 | 16.5 | 17.5 | 18.5 | 29.5 |
| CLIB215 | 9.5 | 26.0 | 19.0 | 21.0 | 7.0 | 3.5 | 18.0 | 22.0 | 8.0 | 5.5 | 20.5 | 4.0 | 32.5 |
| CLIB324 | 19.0 | 8.5 | 7.5 | 17.5 | 27.5 | 9.0 | 13.5 | 1.0 | 21.0 | 2.5 | 17.5 | 3.5 | 6.5 |
| CLIB382 | 14.5 | 26.5 | 11.5 | 25.5 | 9.5 | 3.0 | 25.5 | 23.0 | 3.0 | 23.5 | 30.0 | 20.5 | 25.5 |
| DBVPG1373 | 29.5 | 21.5 | 9.0 | 11.5 | 18.5 | 34.5 | 34.5 | 27.0 | 35.5 | 6.0 | 34.0 | 36.0 | 33.0 |
| DBVPG1788 | 17.0 | 27.0 | 19.0 | 5.0 | 27.0 | 11.5 | 6.0 | 33.0 | 18.5 | 21.5 | 19.0 | 5.0 | 5.5 |
| DBVPG6044 | 21.5 | 28.0 | 24.5 | 16.5 | 30.5 | 25.5 | 8.5 | 29.0 | 15.0 | 20.5 | 16.5 | 3.0 | 13.0 |
| DBVPG6765 | 21.0 | 20.0 | 10.0 | 25.5 | 29.5 | 36.0 | 31.5 | 28.0 | 25.0 | 31.5 | 26.5 | 14.0 | 13.5 |
| Ethanol Red | 14.0 | 13.0 | 33.0 | 6.5 | 6.0 | 15.0 | 19.5 | 6.5 | 16.5 | 5.5 | 9.5 | 9.5 | 11.0 |
| GDB 135-h | 36.0 | 9.5 | 35.0 | 11.0 | 29.5 | 33.0 | 4.5 | 22.0 | 22.5 | 5.0 | 7.5 | 21.5 | 2.5 |
| GDB 325 | 21.0 | 17.0 | 7.0 | 31.0 | 29.5 | 6.0 | 31.0 | 21.0 | 35.0 | 16.0 | 10.5 | 22.5 | 29.0 |
| GDB 379 | 19.5 | 9.5 | 14.5 | 30.0 | 29.5 | 5.0 | 22.0 | 9.0 | 18.5 | 16.5 | 28.5 | 23.0 | 26.5 |
| KK:YS2-h | 15.5 | 14.0 | 20.0 | 10.5 | 5.0 | 8.5 | 16.0 | 10.0 | 12.5 | 23.0 | 5.5 | 24.0 | 14.5 |
| L.1528 | 8.0 | 26.5 | 14.0 | 21.5 | 29.5 | 21.5 | 25.0 | 11.0 | 22.5 | 32.5 | 32.5 | 34.5 | 19.5 |
| LUI250 | 24.0 | 19.0 | 27.5 | 10.5 | 18.5 | 25.0 | 32.0 | 22.5 | 6.0 | 6.5 | 27.0 | 20.0 | 20.0 |
| NCYC110 | 22.0 | 35.0 | 31.5 | 34.0 | 15.5 | 19.5 | 31.5 | 22.0 | 23.5 | 26.5 | 18.0 | 17.5 | 27.5 |
| PW5 | 18.5 | 12.0 | 22.0 | 32.0 | 1.5 | 17.5 | 8.0 | 9.5 | 20.0 | 33.5 | 10.0 | 20.5 | 2.5 |
| RM11 | 12.5 | 9.5 | 7.5 | 8.5 | 8.0 | 12.5 | 21.5 | 9.5 | 15.0 | 22.5 | 27.0 | 24.0 | 13.5 |
| S288C | 21.0 | 8.5 | 19.5 | 26.5 | 19.0 | 15.5 | 20.0 | 23.5 | 17.0 | 5.0 | 27.5 | 21.0 | 15.5 |
| SK1 | 21.5 | 35.0 | 30.5 | 35.5 | 11.5 | 29.5 | 1.0 | 10.0 | 20.5 | 18.0 | 5.5 | 6.5 | 28.0 |
| T7 | 14.0 | 7.0 | 5.0 | 9.0 | 7.5 | 17.0 | 22.0 | 29.5 | 27.5 | 26.0 | 15.0 | 11.0 | 27.5 |
| T73 | 11.5 | 8.0 | 10.5 | 17.5 | 10.0 | 24.5 | 16.0 | 9.0 | 12.0 | 7.5 | 27.5 | 24.0 | 13.0 |
| UWOPS03-461.4 | 22.5 | 35.0 | 33.0 | 35.0 | 13.5 | 27.5 | 2.5 | 10.5 | 15.5 | 16.0 | 7.0 | 21.0 | 27.0 |
| UWOPS05-217.3 | 25.0 | 31.0 | 26.0 | 18.5 | 9.5 | 25.5 | 14.5 | 22.5 | 19.0 | 23.0 | 4.5 | 8.0 | 15.0 |
| UWOPS05-227.2 | 25.5 | 31.5 | 28.0 | 27.0 | 19.0 | 20.5 | 17.5 | 22.5 | 21.5 | 22.5 | 4.0 | 17.0 | 17.5 |
| Y10 | 23.0 | 31.0 | 8.5 | 22.0 | 8.0 | 23.0 | 31.0 | 32.5 | 22.5 | 15.0 | 29.0 | 26.5 | 35.0 |
| Y55 | 10.0 | 11.5 | 7.0 | 8.5 | 2.5 | 21.5 | 5.5 | 5.5 | 12.0 | 27.0 | 24.5 | 5.0 | 15.5 |
| YJM269 | 23.5 | 30.5 | 26.5 | 20.0 | 17.0 | 20.5 | 36.0 | 34.0 | 22.0 | 19.0 | 22.5 | 34.5 | 34.5 |
| YJM975 | 8.5 | 20.0 | 26.0 | 10.0 | 19.0 | 30.5 | 16.5 | 14.5 | 15.0 | 6.5 | 29.0 | 26.5 | 17.5 |
| YJM978 | 13.0 | 21.0 | 22.5 | 19.5 | 29.5 | 29.0 | 16.0 | 12.0 | 12.0 | 8.0 | 27.5 | 26.0 | 15.0 |
| YPS128 | 21.0 | 2.0 | 19.5 | 10.5 | 30.5 | 22.0 | 25.0 | 34.5 | 27.5 | 33.0 | 13.5 | 16.0 | 30.5 |
| YPS606 | 22.5 | 2.5 | 15.5 | 8.0 | 29.5 | 22.5 | 25.5 | 32.5 | 20.5 | 33.5 | 6.5 | 21.5 | 22.5 |

**Table SN4. The normalized Performance rankings.**

| **Strain** | **pH** | **Temperature** | **Ethanol** | **1,4-Butanediol** | **D-Limonene** | **4-Amino-benzoic acid** | **Acetic acid** | **Formic acid** | **Fumaric acid** | **Pyruvic acid** | **Ferulic acid** | **Succinic acid** | **Furfural** |
| --- | --- | --- | --- | --- | --- | --- | --- | --- | --- | --- | --- | --- | --- |
| AL1 | 23.5 | 9.0 | 16.0 | 21.0 | 31.5 | 5.5 | 16.5 | 12.5 | 21.5 | 30.5 | 1.0 | 18.5 | 19.0 |
| AL3-h | 33.5 | 16.0 | 22.0 | 26.5 | 32.5 | 18.0 | 33.5 | 34.5 | 36.0 | 36.0 | 36.0 | 34.5 | 18.5 |
| CA1 | 14.5 | 17.0 | 35.5 | 13.0 | 15.0 | 8.0 | 6.0 | 6.0 | 12.0 | 25.0 | 15.5 | 10.5 | 1.5 |
| CBS7960 | 18.0 | 22.0 | 15.5 | 9.5 | 4.5 | 16.0 | 17.5 | 10.0 | 15.5 | 10.5 | 7.5 | 21.0 | 5.0 |
| CEN.PK113-7D | 28.5 | 16.0 | 13.5 | 27.5 | 34.0 | 18.0 | 10.5 | 22.5 | 11.5 | 17.0 | 15.5 | 16.0 | 26.5 |
| CLIB215 | 32.5 | 25.0 | 28.5 | 31.0 | 15.5 | 17.5 | 27.0 | 27.5 | 27.0 | 24.0 | 31.0 | 30.0 | 32.0 |
| CLIB324 | 24.0 | 7.5 | 9.0 | 18.0 | 26.5 | 8.0 | 21.5 | 2.5 | 24.0 | 12.0 | 30.0 | 25.5 | 8.0 |
| CLIB382 | 15.5 | 23.5 | 15.0 | 14.5 | 9.0 | 2.5 | 17.0 | 17.0 | 4.5 | 10.5 | 23.5 | 8.5 | 18.5 |
| DBVPG1373 | 36.0 | 31.5 | 32.5 | 36.0 | 27.0 | 35.5 | 34.0 | 34.5 | 32.5 | 34.5 | 33.0 | 36.0 | 35.5 |
| DBVPG1788 | 18.5 | 27.0 | 17.5 | 8.0 | 27.0 | 9.0 | 13.5 | 29.0 | 23.0 | 21.5 | 13.5 | 17.5 | 7.5 |
| DBVPG6044 | 21.0 | 27.5 | 16.5 | 13.5 | 28.0 | 25.5 | 19.0 | 29.5 | 23.5 | 24.5 | 28.5 | 15.5 | 19.5 |
| DBVPG6765 | 10.0 | 17.0 | 3.5 | 19.0 | 25.5 | 34.0 | 26.0 | 21.5 | 12.0 | 24.5 | 25.5 | 4.5 | 12.5 |
| Ethanol Red | 22.0 | 15.0 | 32.5 | 16.0 | 7.0 | 19.5 | 21.0 | 9.0 | 19.0 | 6.0 | 11.0 | 22.0 | 12.5 |
| GDB 135-h | 34.0 | 11.0 | 35.5 | 34.0 | 36.0 | 35.0 | 6.0 | 20.0 | 17.5 | 7.0 | 7.5 | 25.5 | 3.0 |
| GDB 325 | 13.5 | 9.5 | 4.5 | 25.0 | 26.5 | 3.0 | 31.0 | 17.5 | 21.5 | 15.5 | 11.0 | 19.5 | 26.5 |
| GDB 379 | 11.5 | 6.5 | 9.0 | 20.5 | 25.5 | 2.0 | 12.5 | 7.5 | 7.5 | 7.0 | 23.5 | 6.0 | 18.5 |
| KK:YS2-h | 31.0 | 21.5 | 25.5 | 31.0 | 11.0 | 23.0 | 29.0 | 21.0 | 32.5 | 34.5 | 14.0 | 32.5 | 31.0 |
| L.1528 | 4.5 | 21.0 | 6.5 | 15.5 | 28.0 | 16.0 | 10.0 | 3.0 | 5.0 | 14.5 | 20.0 | 21.0 | 8.0 |
| LUI250 | 22.5 | 26.5 | 32.5 | 33.5 | 23.5 | 32.5 | 28.5 | 18.5 | 7.5 | 4.5 | 23.5 | 13.0 | 13.0 |
| NCYC110 | 26.0 | 36.0 | 32.5 | 32.0 | 16.5 | 20.5 | 29.5 | 22.5 | 22.5 | 25.0 | 23.5 | 29.0 | 31.5 |
| PW5 | 4.5 | 6.5 | 7.0 | 12.0 | 1.0 | 6.0 | 3.0 | 5.0 | 5.0 | 24.0 | 3.0 | 2.5 | 1.0 |
| RM11 | 5.5 | 8.0 | 9.0 | 13.5 | 8.0 | 9.0 | 7.0 | 3.0 | 3.0 | 6.0 | 14.0 | 2.5 | 5.0 |
| S288C | 29.0 | 14.0 | 20.5 | 19.5 | 20.0 | 21.0 | 28.0 | 28.5 | 26.0 | 17.0 | 32.5 | 30.0 | 26.0 |
| SK1 | 14.0 | 34.5 | 28.0 | 30.0 | 9.5 | 28.5 | 1.0 | 15.5 | 19.0 | 15.5 | 6.0 | 17.0 | 26.5 |
| T7 | 10.5 | 2.0 | 3.5 | 8.0 | 4.0 | 16.0 | 20.5 | 25.0 | 24.0 | 24.5 | 18.0 | 20.0 | 25.5 |
| T73 | 8.0 | 6.5 | 9.5 | 11.5 | 7.5 | 19.0 | 8.5 | 7.5 | 6.5 | 2.0 | 20.0 | 7.5 | 7.5 |
| UWOPS03-461.4 | 11.5 | 34.5 | 29.5 | 31.5 | 8.0 | 23.0 | 3.5 | 13.0 | 10.5 | 9.0 | 6.5 | 11.5 | 26.0 |
| UWOPS05-217.3 | 15.5 | 27.5 | 22.5 | 5.0 | 7.5 | 21.5 | 18.5 | 23.0 | 23.0 | 23.5 | 3.0 | 9.5 | 20.5 |
| UWOPS05-227.2 | 11.5 | 28.5 | 17.5 | 6.5 | 15.5 | 10.5 | 17.5 | 22.0 | 21.0 | 16.0 | 3.0 | 11.0 | 19.0 |
| Y10 | 30.0 | 32.0 | 23.0 | 26.5 | 13.0 | 29.0 | 34.0 | 34.5 | 32.5 | 31.0 | 34.0 | 32.5 | 35.5 |
| Y55 | 9.0 | 8.5 | 5.5 | 3.5 | 3.0 | 19.0 | 3.5 | 3.0 | 5.5 | 15.5 | 16.0 | 2.0 | 8.5 |
| YJM269 | 29.5 | 31.5 | 28.0 | 22.5 | 17.0 | 25.5 | 36.0 | 34.5 | 32.5 | 32.5 | 34.0 | 34.5 | 32.0 |
| YJM975 | 10.0 | 20.0 | 23.0 | 9.0 | 18.5 | 30.5 | 10.0 | 12.5 | 10.0 | 3.0 | 24.5 | 17.0 | 14.0 |
| YJM978 | 10.0 | 19.5 | 23.0 | 18.0 | 31.0 | 29.5 | 13.5 | 11.0 | 6.5 | 5.5 | 24.5 | 13.5 | 14.0 |
| YPS128 | 12.0 | 2.0 | 8.0 | 2.5 | 26.0 | 13.0 | 28.0 | 31.5 | 32.5 | 31.5 | 24.0 | 23.0 | 32.5 |
| YPS606 | 14.0 | 2.0 | 6.0 | 1.5 | 26.0 | 15.0 | 24.5 | 30.0 | 32.5 | 27.0 | 7.5 | 26.0 | 24.5 |

**Rank Variability Analysis (RVA)**

The Robustness and Performance scores are useful for obtaining a general overview of the strains by ranking them according to descending property scores. However, such a ranking does not consider the associated errors in the parameters. The most likely rank for each strain was estimated through random sampling of the probable space of the level-specific Robustness and Performance scores. For each strain and property (i.e. level-specific Robustness or Performance), 1000 random values were generated from a normal distribution with mean and standard deviation as calculated above. From these values 1000 scores of Robustness or Performance were calculate as described above for each strain. By ranking the strains according to the Robustness or Performance scores (in descending order), an equal number of ranking values was obtained. As an example, histograms of the Robustness and Performance scores as well as the ranking values from the analysis of 1,4-butanediol are shown in **Figure SN1**. This figure shows that the distributions of the ranking values are not always normally distributed, which means that mean and standard deviation cannot be used to describe all the data.

**Figure SN1. Distributions of property scores and ranking values from the analysis of 1,4-butanediol.** Panels (**A**) and (**B**) show the distributions of Performance and Robustness scores, respectively, for all strains. The distributions of the associated ranking values are shown in panels (**C**) and (**D**), respectively.

The property scores on the other hand are always normally distributed (**Figure SN1**) and by fitting a normal probability density function (*pdf*) to the data, the mean and standard deviation can be estimated. The property scores are also normally distributed within each rank value (**Figure SN2**) and hence, the mean score and the 95% CI can be calculated at each rank value. These values were used to i) estimate the most likely range of ranking values for each strain and ii) to build a *pdf* for the rank values for each strain. The most likely range of ranking values for each strain was estimated by determining within which ranking values the mean ± 1σ would fall given the 95% CIs associated with each ranking value. The generation of a *pdf* for the ranking values begins with estimating the probability of observing a score within the 95% CI associated with each rank value. This was done by integrating under the *pdf* fitted to the property scores within that interval. The *pdf* for the ranking distribution was then obtained by normalizing all 36 probabilities so that the sum equals to one (**Figure SN3**). By integrating under this *pdf*, the probability for the raking range can be estimated, as well as the probability for each strain to fall within a rank of 10 and below a rank of 26 (i.e. within the top and bottom 10 strains, respectively).

**
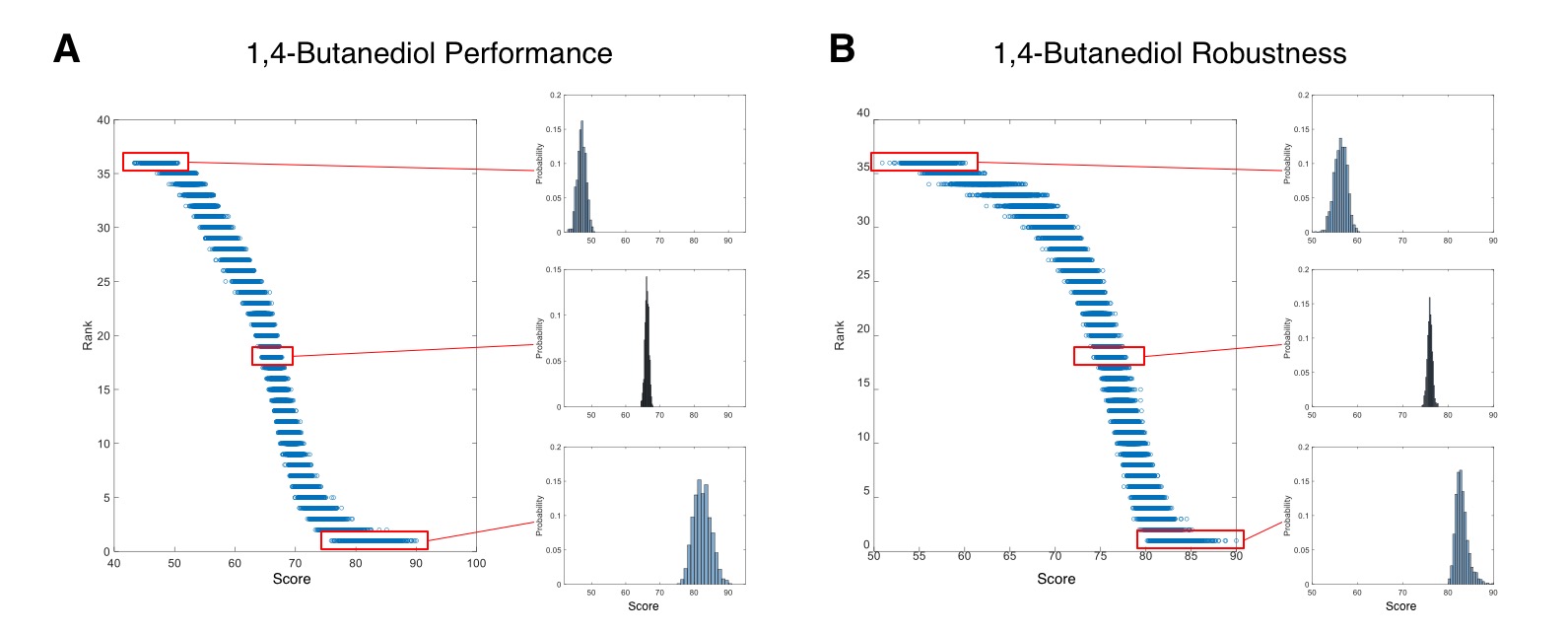
**

**Figure SN2. Distributions of property scores within rank values.** The distributions of Performance (**A**) and Robustness (**B**) scores are shown for ranking values 36, 18 and 1.

**
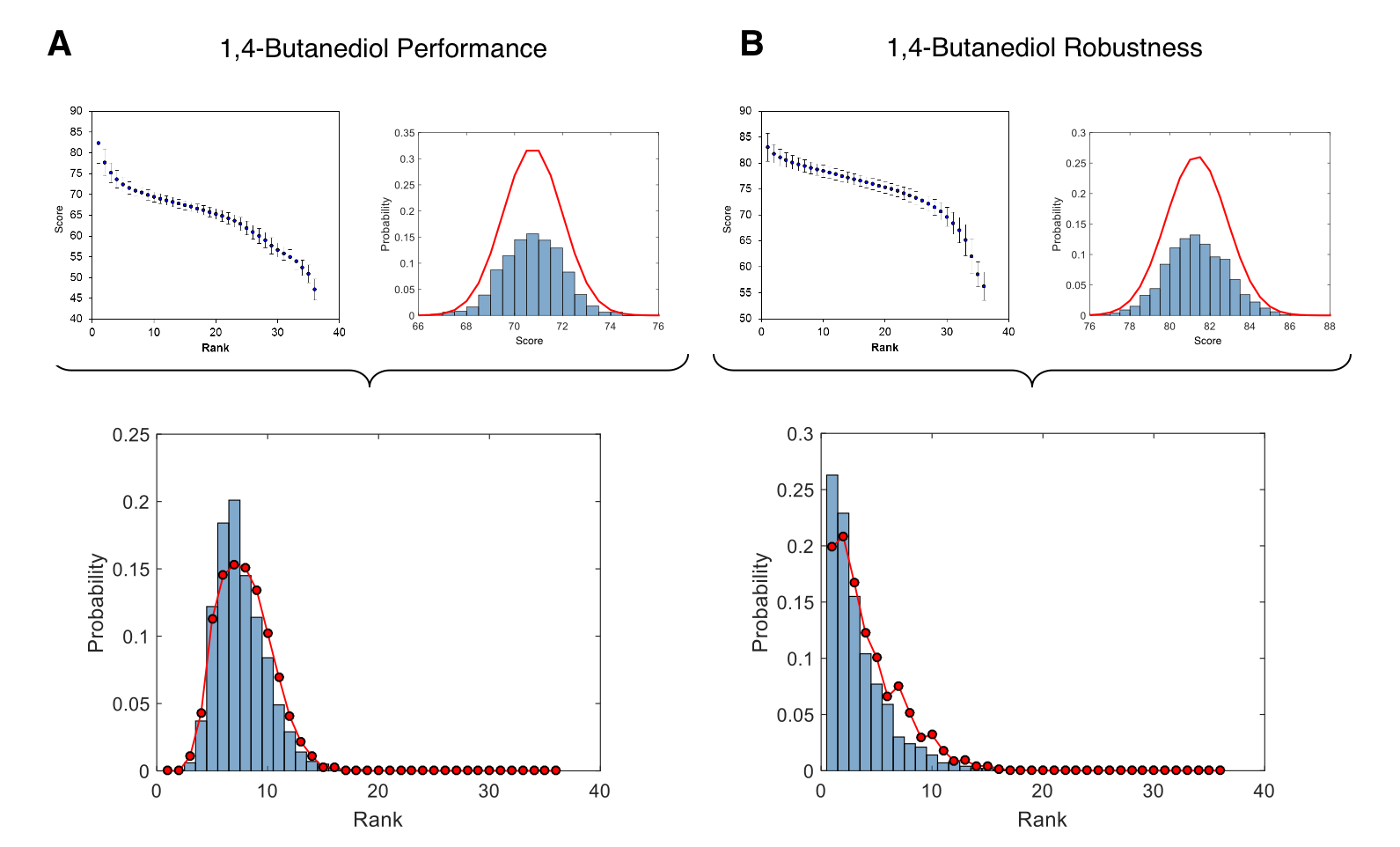
**

**Figure SN3. Estimation of a probability density function for the ranking values.** The probability of observing a property score within the range associated with each ranking value is estimated by integrating under the *pdf* fitted to the property score data for each strain. By normalizing the probabilities to sum to one, the probability for one strain to assume each ranking value is estimated. These values are plotted on top of the ranking distribution of (**A**) Performance and (**B**) Robustness for strain DBVPG1788 using data from 1,4-butanediol experiments.

**Parameter Influence Analysis (PIA)**

The scores obtained following the procedure described above should be considered as general scores since all the growth parameters are given equal weights. Hence, two strains can have the same general score even though they have quite different phenotypes. For example, if strains A and B perform equally in terms of average growth rate, but one excels in lag phase and number of generations whereas the other excels in growth duration and maximum growth rate, both strains would score the same despite very different phenotypic profiles. The influence of the parameters on the ranking of the strains was assessed by performing an RVA at different weights for the parameter of interest. The parameter weights increased from 0 (i.e. the parameter is not used in the calculation of the scores and hence not taken into account when ranking the strains) to 5 (i.e. the scores and the ranking are based solely on the current parameter) with an increment of 0.1 in every iteration. The other parameters were given equal weights such that the total sum of all weights equaled five. For example, if the weight for parameter 1 was set to 3, the other four parameters were all given (5-3)/4 = 0.5 as the weight. The result can be represented as a plot of mid rank value against the parameter weight for all the parameters (**Figure SN4**). Each series is normalized to start at the origin and the influence can be quantified as the area under the normalized values. This area has the following characteristics: i) if the rank value is unaffected by the parameter the area will be close to zero, ii) if the rank value tends to increase with increasing parameter weight the area will be large and positive and iii) if the rank value tends to decrease with increasing parameter weight the area will be large and negative (**Figure SN5**). The parameter influence score is given as the negative value of the area. Hence a positive score means that there is a positive effect on the ranking position of the strain (i.e. the ranking value decreases) and a negative value means that there is a negative effect on the ranking position (i.e. the ranking value increases).

**Figure SN4. Dependency of the mid rank value on the different parameters.** The Parameter Influence Analysis is based on independent evaluations of each growth parameter. An RVA is performed at different weights for the parameter of interest to estimate its influence on the ranking. The figure shows the evaluation of DBVPG1788 in 1,4-butanediol regarding (**A**) Performance and (**B**) Robustness. P1~P5 stand for: 1. Lag phase; 2. Growth duration; 3. Number of generations during the growth phase; 4. Average specific growth rate during the growth phase; 5. Maximum specific growth rate.

**Figure SN5. Quantification of parameter influence.** (**A**) Each parameter series is normalized to start at the origin. The area under these normalized values represents the influence that each parameter has on the ranking of a strain. The figure shows the normalized ranking values and the corresponding area for three parameters with different influence on the ranking of DBVPG1788 in Robustness towards 1,4-butanediol. (**B**) The parameter influence scores are the negative values of the area and are shown for Performance and Robustness of DBVPG1788 in 1,4-butanediol. P1~P5 stand for: 1. Lag phase; 2. Growth duration; 3. Number of generations during the growth phase; 4. Average specific growth rate during the growth phase; 5. Maximum specific growth rate.

**Parameter influence analysis on Y55**

To gain further insights into to the phenotypic characteristics of the strains, we analyzed how the ranking values are affected when each of the five growth parameters is given increasing weight. **Figure S2** shows the results of this analysis for strain Y55, the most versatile strain regarding the resistance traits. The ranking of Y55 is not very sensitive to any particular parameter in D-limonene, acetic acid, formic acid, ferulic acid and furfural as the cumulative scores fall within or just above the average among all strains (**Figure S2**). On the other hand, the ranking in Performance toward temperature and 4-aminobenzoic acid is dependent on which parameter is given more weight. The lag phase has a positive effect on the ranking position whereas the number of generations and maximum growth rate have negative effects leading to lower ranking position. Hence, in these conditions there are other strains with better properties with regard to these parameters. The ranking in Robustness shows a much higher degree of dependency on the parameters than the Performance ranking as the bars extend beyond the average level in several conditions. In nearly all of these conditions, the ranking position is positively affected by increased weight on the maximum growth rate, contrary to the pattern in Performance, indicating that Y55 is able to maintain the value of this particular parameter better than other strains in several conditions. To identify strains that differ significantly from Y55 in terms of ranking sensitivity, we performed a correlation analysis of the parameter influence scores. In total, we found 28 and 32 significant (*P* value ≤ 0.05) negative correlations (*R* ≤ -0.90) in Performance and Robustness over all conditions (**Figure S2**). However, the majority of these (89% and 75% in Performance and Robustness, respectively) were comparisons with strains that have a low probability of falling within the same rank range (*Pr* ≤ 0.48). In Performance, three strains with a significant negative correlation with the parameter influence of Y55 also had a similar ranking range: YPS128 (1,4-butanediol; *Pr* = 0.63), CBS7960 (D-limonene; *Pr* = 0.60) and CEN.PK113-7D (pyruvic acid; *Pr* = 0.72) (**Figure S2**). In Robustness, we found eight such cases in pH (KK:YS2-h; *Pr* = 0.60), temperature (CEN.PK 113-7D; *Pr* = 0.88), ethanol (DBVPG1373; *Pr* = 0.79), 1,4-butanediol (RM11; *Pr* = 0.90), 4-aminobenzoic acid (NCYC110; *Pr* = 0.75), fumaric acid (YJM975; *Pr* = 0.71) and ferulic acid (DBVPG6765 and YJM269; *Pr* = 0.71 for both) (**Figure S2**).

**Supplemental Note S3. Metabolome.**

**Intracellular and extracellular metabolite profiling using GC-MS**

A single colony isolated on YPD agar was used to pre-inoculate 100 mL of minimum mineral (MM) medium (pH 5.5; as described in the media used in measurements of stress conditions [2]) at 30 °C using shake flasks in a rotary shaker at 170 rpm for 24 h. The cells were collected by centrifugation at 2000 x g (4 °C) for 5 min and washed twice in phosphate buffered saline (8 g/L NaCl, 0.2 g/L KCl, 1.44 g/L Na_2_PO_4_, 0.24 g/L KH_2_PO_4_, at pH 7.5). The cell pellets were resuspended in fermentation medium at an initial OD_600_ of 1. The cells were incubated in a rotary shaker-incubator (170 rpm) at 30 °C until reached OD_600_ of 5.

To accommodate the lower OD_600_ for glucose medium, six shake-flask cultures (100 mL) were harvested at late exponential growth phase and two cultures were rapidly pooled together prior sampling. For intracellular metabolomics analysis, five technical replicates (20 mL) for each pooled culture were rapidly filtered under vacuum (Air Cadet vacuum/pressure station, ThermoFisher), quickly washed with cold saline solution (1-2 °C) and quenched in 2.5 mL of cold methanol water (50 % v/v) at -30 °C as described by Smart *et al*. [3]. Three samples (1 mL) of the microbial cultures were filtered (0.2 μm pore size membrane) to remove the *S. cerevisiae* cells, and the filtrate was used for the analysis of extracellular metabolites.

The intracellular metabolites were extracted from the quenched cell pellets using cold methanol/water and freeze-thaw cycles following the protocol optimized by Smart *et al*. [3]. In brief, the internal standard 2,3,3,3-d_4_-alanine was added to each sample (0.3 μmol/sample) before extraction. The solution was mixed vigorously for one minute, frozen at -80 °C, and thawed at 4 °C. After the cycle had been repeated two additional rounds, the samples were centrifuged at -20 °C for 15 min at 20,800 *x* g. The supernatant was collected and the remaining cells were resuspended in 2.5 mL of cold methanol water (70% v/v) at -30 °C. Centrifugation was repeated under the identical conditions and the supernatant collected was pooled with the first collection. The intracellular metabolite extracts and spent culture medium containing extracellular metabolites were lyophilized (BenchTop K manifold freeze dryer, VirTis) before chemical derivatization. The lyophilized samples were derivatized using the methyl chloroformate (MCF) method according to the protocol described by Smart *et al.* [3]. In summary, the lyophilized samples were resuspended in 200 μL of sodium hydroxide solution (1 M) and transferred to a silanized glass tube, then mixed with 167 µL of methanol and 34 µL of pyridine. The derivatization started by adding 20 µL of MCF followed by vigorously mixing for 30 s, and then a further 20 µL of MCF was added followed by vigorously mixing for 30 s. To separate MCF derivatives from the reactive mixture, 400 µL of chloroform was added and vigorously mixed for 10 s followed by the addition of 400 µL of sodium bicarbonate solution (50 mM), and mixing for an additional 10 s. The aqueous layer was removed and dehydrated with anhydrous sodium sulfate before samples were transferred to GC-MS vials.

The MCF derivatives were analyzed in an Agilent GC7890 system coupled to a MSD5975 mass selective detector (EI) operating at 70 eV. The column used for all analyses was a ZB-1701 GC capillary column (30 m x 250 μm id x 0.15 μm with 5 m guard column, Phenomenex). The analysis parameters were conducted in accordance with Smart *et al*. [3]. Samples were injected under pulsed splitless mode with the inlet temperature at 290 °C. The helium gas flow through the GC-column was set at 1.0 mL/min. The GC-oven temperature was initially held at 45 °C for 2 min. Afterward the temperature was raised with a gradient of 9 °C/min until it reached 180 °C and was held for 5 min. Then the temperature ramped at 40 °C/min until it reached 220 °C and was held for 5 min, after which the temperature was ramped at 40 °C/min until it reached 240 °C and was held for 11.5 min. Finally, the temperature was ramped at 40 °C/min until it reached 280 °C and was held for 2 min. The auxiliary temperature was set to 250 °C, the MS quadrupole temperature was 150 °C, and the MS source temperature was 230 °C. The mass spectrometry was operated in scan mode and began after 5.5 min with mass range between 38-550 amu and scan frequency of 2.9 scans/s. The MS detector was turned off after 40 min.

**Metabolite identification and normalization of GC-MS data**

AMDIS (Automated Mass Spectral Deconvolution and Identification System) software was employed for deconvoluting GC-MS chromatograms and identifying metabolites using an in-house MCF mass spectra library. The metabolite identifications were confirmed by both the MS spectrum of the derivatized metabolite and its respective chromatographic retention time. The relative abundance of identified metabolites was extracted by an self-developed algorithm developed in R: Metab (GitHub: https://github.com/reinkk/Metab, SciCrunch ID: SCR_016877), which automatically integrate the GC base-peak value of a selected reference ion. After the false positive and missing values were manually corrected, the abundance values were normalised by the abundance of internal standard (2,3,3,3-d4-alanine) as well as by the biomass content in each samples. In the case of extracellular profile, the abundance values were also normalised by metabolite composition of the culture medium. The biomass content (dry weight) was determined after intracellular metabolite extraction by drying the collected cell debris using a domestic microwave (250 W for 20 min), cooling in a desiccator overnight, followed by weighing of each sample.

**Compound class and pathway assignment**

Compounds identified were assigned to different classes and pathways by the pathway information in YMDB (Yeast Metabolic Database) [4]. These pathway and compound class assignment were summarized in **Dataset S2**.

Metabolite data was transformed before calculating the contribution to each group of pathways and/or metabolite classes. Both intracellular and extracellular metabolites were scaled to the biological range [5]. Intracellular metabolite data was transformed by subtracting the mean of the data set, followed by division with the difference between the maximum and minimum value of the particular metabolite. This difference is referred to as the biological range and makes all metabolites equally important [5]. Extracellular metabolites were first centered by the starting amount prior to scaling to the biological range, and were grouped according to compound classes as well as if they were produced (scaled value > 0) or consumed (< 0). Metabolites belonging to one of the selected classes and had positive or negative values were added together after scaling, respectively, to show production and consumption. For both intracellular and extracellular metabolites, scaled values of metabolites belonging to a pathway or compound class were added together for each strain to create group variables.The sum was subsequently divided by the square root of the number of metabolites to be comparable with the other classes. Hierarchical clustering was applied to the group variables using Euclidian distance between the strains.

**Metabolite profile similarity by Principal Component Analysis (PCA) analysis**

To examine the repeatability of the metabolomic profiling, PCA analyses were performed for intra- and extracellular metabolomes respectively. Metabolomic profiles from the same strain (as biological replicates and technical replicates) clustered well in PCA plots (**Figures SN6 & SN7**).

**Figure SN6.** **The PCA plot for intracellular metabolome**.

**Figure SN7.** **The PCA plot for extracellular metabolome**.

**Supplemental Note S4. Strain-specific GSMM construction.**

**Investigation of differences in metabolic networks using strain-specific genome scale models**

A previously published genome-scale metabolic model for the reference strain S288c [6] was customized using genomic and physiological data to build strain-specific models for all 36 strains. Non-S288c genes acquired from *de novo* assembly and annotation were used as potential candidates of new reactions. For each candidate gene, its respective EC number (when available) was retrieved from UniProt [7]. A total of 18 unique EC numbers were identified among all strains (**Table SN5**). This list includes 7 non-metabolic genes with functions associated with transcription and regulation (e.g., DNA polymerases, ribonucleases, protein kinases). For the remaining EC numbers, all except one were found in the annotations of the reference model, indicating that the associated functions are already present in the genome-scale reconstruction of S288c. The exception is EC 3.1.2.4 (3-hydroxyisobutyryl-CoA hydrolase), which was found in AL1. This enzyme is part of the KEGG pathway for valine degradation. However, the rest of the pathway was not identified in this strain, which could indicate a false positive match. In summary, we could not identify any gain of new metabolic functions among the different strains.

**Table SN5. Candidates for new EC numbers for the strain-specific models.**

| EC Number | # of Strains | Notes |
| --- | --- | --- |
| 1.1.1.1 | 10 | Already in iMM904 |
| 1.11.1.6 | 1 | Already in iMM904 |
| 1.2.1.12 | 2 | Already in iMM904 |
| 1.3.98.1 | 13 | Renamed from 1.3.3.1 (already in iMM904) |
| 2.6.1.62 | 5 | Already in iMM904 |
| 2.7.11.24 | 2 | Protein kinase |
| 2.7.12.1 | 1 | Protein kinase |
| 2.7.7.49 | 18 | DNA Polymerase |
| 2.7.7.7 | 18 | DNA Polymerase |
| 3.1.2.1 | 1 | Already in iMM904 |
| 3.1.2.4 | 1 | 3-hydroxyisobutyryl-CoA hydrolase |
| 3.1.26.4 | 18 | Ribonuclease |
| 3.2.1.22 | 8 | Already in iMM904 |
| 3.5.1.98 | 1 | Histone deacetylase |
| 3.6.4.12 | 3 | DNA Helicase |
| 4.3.1.17 | 27 | Already in iMM904 |
| 5.3.1.23 | 2 | Already in iMM904 |
| 6.1.1.6 | 1 | Already in iMM904 |
|  |  |  |

The genomic data was then used to identify potential gene losses among the strains, including gene deletion (zero copies) and possible loss of function (truncation and/or elongation). We implemented a new model-building algorithm that uses these data in combination with physiological data (**Dataset S5**) to build strain-specific models. The algorithm tries to remove all potentially lost genes except those required to achieve the measured growth and by-product secretion rates. This results in a median value of 23 metabolic genes deleted per strain (**Table SN6**). We performed a pairwise comparison of the strain-specific models in terms of network similarity (Jaccard distance). It can be observed that the networks differ by at most 2% of their total number of reactions (**Figure SN8**).

**Table SN6. Candidates for new EC numbers for the strain-specific models.**

| Strain | Lost Genes | Lost Metabolic Genes | Lost Reactions | Recovered Reactions | Recovered Genes | Growth Rate (*) | |  |
| --- | --- | --- | --- | --- | --- | --- | --- | --- |
| AL1 | 243 | 13 | 5 | 1 | 1 | 0.975418211 | |  |
| AL3-h | 356 | 24 | 13 | 2 | 2 | 0.975418211 | |  |
| CA1 | 286 | 23 | 9 | 3 | 2 | 0.975418211 | |  |
| CBS7960 | 291 | 19 | 9 | 2 | 1 | 0.975418211 | |  |
| CEN.PK113-7D | 183 | 18 | 10 | 1 | 1 | 0.975418211 | |  |
| CLIB215 | 316 | 33 | 19 | 4 | 3 | 0.975418211 | |  |
| CLIB324 | 24 | 4 | 1 | 1 | 1 | 0.975418211 | |  |
| CLIB382 | 309 | 22 | 14 | 0 | 0 | 0.975418211 | |  |
| DBVPG1373 | 316 | 28 | 18 | 2 | 1 | 0.975418211 | |  |
| DBVPG1788 | 468 | 32 | 24 | 4 | 3 | 0.975418211 | |  |
| DBVPG6044 | 490 | 35 | 24 | 5 | 4 | 0.975418211 | |  |
| DBVPG6765 | 268 | 20 | 7 | 2 | 1 | 0.975418211 | |  |
| Ethanol Red | 316 | 17 | 13 | 0 | 0 | 0.867365416 | |  |
| GDB135-h | 19 | 2 | 1 | 1 | 1 | 0.975418211 | |  |
| GDB325 | 215 | 17 | 9 | 0 | 0 | 0.867365416 | |  |
| GDB379 | 236 | 14 | 7 | 0 | 0 | 0.975418211 | |  |
| KKYS2-h | 365 | 26 | 13 | 0 | 0 | 0.867365416 | |  |
| L.1528 | 288 | 23 | 9 | 2 | 1 | 0.975418211 | |  |
| LU1250 | 302 | 20 | 10 | 2 | 1 | 0.975418211 | |  |
| NCYC110 | 467 | 32 | 22 | 5 | 4 | 0.975418211 | |  |
| PW5 | 439 | 26 | 13 | 4 | 3 | 0.975418211 | |  |
| RM11 | 286 | 21 | 8 | 2 | 1 | 0.975418211 | |  |
| S288c | 9 | 0 | 0 | 0 | 0 | 0.975418211 | |  |
| SK1 | 416 | 25 | 19 | 2 | 2 | 0.975418211 | |  |
| T7 | 394 | 25 | 17 | 1 | 1 | 0.975418211 | |  |
| T73 | 7 | 0 | 0 | 0 | 0 | 0.975418211 | |  |
| UWOPS03-461.4 | 451 | 33 | 24 | 3 | 3 | 0.209365937 | |  |
| UWOPS05-217.3 | 415 | 28 | 22 | 2 | 2 | 0.975418211 | |  |
| UWOPS05-227.2 | 414 | 29 | 23 | 1 | 1 | 0.975418211 | |  |
| Y10 | 428 | 27 | 13 | 3 | 2 | 0.975418211 | |  |
| Y55 | 383 | 26 | 21 | 4 | 3 | 0.975418211 | |  |
| YJM269 | 367 | 20 | 8 | 3 | 2 | 0.975418211 | |  |
| YJM975 | 271 | 18 | 7 | 2 | 1 | 0.975418211 | |  |
| YJM978 | 313 | 26 | 11 | 4 | 3 | 0.975418211 | |  |
| YPS128 | 356 | 22 | 16 | 2 | 2 | 0.975418211 | |  |
| YPS606 | 355 | 23 | 15 | 1 | 1 | 0.975418211 | |  |
| (*) for a glucose uptake rate of 10 mmol/gDW/h under aerobic growth | | | | | | |  | |

**Figure SN8.** **The dissimilarities among strain-specific Genome-scale Metabolic Models.** The numbers of the disparate reactions in pairwise comparisons were shown in color gradient.

**Discovery of new metabolic reactions**

The UniProt database [7] was used to search the gene accession numbers from the candidate gene list and retrieve the respective EC numbers. All genes where the top blast hit matched the S288c strain were discarded. The MetaNetX database [8] was used to search metabolic reactions associated with each EC number.

**Strain-specific model building**

Copy-number variation and mutation data were used to determine potential gene deletions in each strain. The severity of the mutations was classified as follows: HIGH for zero gene copies; MEDIUM for truncated genes (*start lost*, *stop gained* or *frame shift* events); and LOW for elongated genes (*stop lost* events). For polyploid strains the mutation of heterologous genes was disregarded if another version of the gene remained functional. This information was used to build strain-specific models using the following mixed integer linear programming (MILP) formulation:

$$\min\sum_{i=1}^{n} w_{i}\cdot y_{i}$$

$$s.t.$$

$$S\cdot v=0$$

$lb\leq v\leq ub$

$v_{j}^{*} - \sigma_{j}\leq v_{j}\leq v_{j}^{*}+ \sigma_{j}$ $\forall_{j} \in experimental$

$-y_{i}M\leq v_{i}\leq y_{i}M$ $\forall_{i} \in\{1,\ldots,n\}$

$$y_{i} \in\left\{ 0,1 \right\}$$

where *S* is the stoichiometric matrix, *n* is the total number of reactions in the original model, *v* is the network flux distribution at steady-state, *lb* and *ub* are the lower an upper flux bounds, *v^*^* is a vector of experimentally determined rates (glucose uptake rate, growth rate, and glycerol, acetate, ethanol, pyruvate, and succinate secretion rates), σ are the respective standard deviations, *M* is a large integer (1000), *y_i_* are binary variables indicating if reaction *i* is used, and *w_i_* are weighting factors that represent the priority to disable a particular reaction. The weighting factors are calculated from the gene-protein-reaction (GPR) associations in the model. For reactions catalyzed by single genes, the weighting factors for gene deletions classified as HIGH, MEDIUM or LOW are 10, 5, and 1, respectively. For reactions catalyzed by protein complexes, the deletion priority is the maximum of all subunits, and for reactions catalyzed by multiple isozymes the priority is the lowest of all respective genes. This algorithm was used to build customized models for each strain using iMM904 as the reference model [6]. The environmental conditions were defined according to the medium composition used for strain cultivation.

**Strain-specific simulation of carbon and nitrogen source utilization**

The simulation of growth on different carbon and nitrogen sources was performed by setting a maximum uptake rate of 10 mmol/gDW/h for the respective substrate and unlimited uptake of other compounds present in the cultivation medium. The carbon and nitrogen sources used for simulation correspond to all sources available in the Biolog plates for which a suitable transporter was present in the iMM904 model.

The models were able to predict strain differences in the utilization of 30 carbon sources and 5 nitrogen sources (**Figure SN9**), however, the in-silico prediction data do not fully meet the experimental data for selected strains (CEN.PK, Ethanol Red and S288c) (**Dataset S5**). This also fits our expectation that the strain-specific phenotypes may largely arise from the differences in regulatory rather than metabolic network, thus could be hardly characterized by GSMMs which take the genotypes of enzymes into consideration. Regulatory network introduced to metabolic models may ultimately improve the construction and utility of strain-specific models.

**Figure SN9.** **The growth rates on different carbon and nitrogen sources predicted by the strain-specific GSMMs.** (**A**) The growth rates on 30 different carbon sources, for which differences could be observed among the strains. (**B**) The growth rates on 5 different nitrogen sources, for which differences could be observed among the strains. The strain UWOPS03−461.4 was removed as an outlier in the prediction.

**Strain-specific simulation of intracellular fluxes**

The simulation of intracellular fluxes was performed by setting a maximum uptake rate of 10 mmol/gDW/h for glucose and unlimited uptake of other compounds present in the cultivation medium. The relative flux values represent a percentage of the glucose uptake rate (starting at 100%). The absolute flux rates were calculated by combining the yield data with growth rate measurements. All the carbon and nitrogen source utilization and flux simulations were performed with the FRAMED package (<https://github.com/cdanielmachado/framed>) using Gurobi 6.5. The differentially activated fluxes were illustrated in **Figure SN10**.

**Figure SN10.** **The differentially activated fluxes among all the strains, predicted by the strain-specific GSMMs.** (**A**) The flux values normalized by glucose intake. (**B**) The absolute flux values from *in silico* prediction. Only the most significantly differing fluxes were shown (SD > mean value).

**Supplemental Note S5. Genome-wide association study.**

**Core-genome GWAS**

Compared with human or crop genomic studies where GWAS were usually applied, high genetic diversity was observed in the *S. cerevisiae* population, with relatively small population size from this study. To increase the accuracy of the GWAS results and remove the potential false positive findings caused by the structural divergence (such as pan-genome regions, CNV regions and transposons) or low sequencing quality areas (centromere, chromosome ends, etc.), we performed the SNP-based GWAS (including markers from SNPs and small InDels) focusing on only core-genome region shared by all strains, and called it Core-GWAS.

To identify the core-genome regions, comparative genomic analysis was performed using a similar strategy to the one described by Oh *et al.* [9], by applying MUSCLE [10] and Bowtie2 [11] alignments. As an assembly-assisted method, all long contigs (> 200 bp) from *de novo* assembly were aligned to the reference genome of S288c. Core genome regions shared by all strain genome assemblies with high sequencing quality were extracted. Finally, 9.8M of the 12.1M genome (81.1%) was identified as the core-genome. In the 379,129 called SNPs/InDels, 245,923 passed the minor allele frequency (MAF) filtration (MAF > 5%), and 165,358 (67.2%) were in the core-genome and analyzed by the SNP-based GWAS. The proportion of the retained SNPs/InDels was much lower than the proportion of the length in core-genome, suggesting that the pan-genome regions were of greater variability with high SNP density (2.1x to core-genome region). Excluding such SNPs/InDels could improve the confidence of the GWAS statistics, by eliminating the influence from non-SNP factors. Meanwhile, most of the pan-genome regions were processed by the CNV-based GWAS, incorporating the presence/absence and copy-number information from the various strains.

**Linkage disequilibrium (LD) block identification**

To identify the independent variants and accommodate the gene-level GWAS analyses, LD-based SNP pruning was performed. LD blocks were generated with Haploview [12] by applying the Four Gamete Rule. Homologous SNPs and InDels with Minor Allele Frequency (MAF) > 5% were used to construct the linkage disequilibrium map. The variants not located in LD blocks were identified as unlinked SNPs/InDels. In visualization, LDs and unlinked SNPs/InDels were used, and individual SNPs/InDels located in LD blocks were omitted.

**The preparation of CNV markers for CNV-based GWAS**

All CNV regions from all strains were aligned and split into non-overlapping fragments, following the procedure described in **Figure SN11**. For each segment, two markers for gain and loss events respectively were generated. If the gain or loss event was not happening in any strain, this marker (with genotype value 0 for all strains) was removed in CNV-based GWAS.

**Figure SN11. The CNV marker preparation for the CNV-based GWAS.**

**Genotype value transformation in GWAS**

To apply the EMMA algorithm to GWAS, genotypic values for the genetic markers should be transformed to 0, 0.5 or 1 for each genetic marker. In the SNP-based GWAS, due to the non-uniformity of the strain ploidies, we used the genotype value 1 to represent all homozygous variants and 0.5 for all heterozygous variants (e.g., for a triploid strain, variants with the actual genotype of 0/0/1 or 0/1/1 were transformed to 0.5). In CNV-based GWAS, different transformations for gain events (the actual copy-number is greater than the original strain ploidy) and loss events (the actual copy-number is less than the original strain ploidy) were applied. For a gain event, the genotype value was set to 1 if the copy-number is equal or greater than twice of the strain’s ploidy, or else 0.5. For a loss event, the genotype value was set to 1 when the copy-number is zero (a region deletion), or else 0.5.

**Gene-level association assignment**

Variant (LD blocks, unlinked SNPs/InDels or CNVs) level association P values were assigned to related genes. In the SNP-based GWAS, variant level P values were first assigned to the respective LD blocks. Gene-level P values (with 800 bp upstream sequences included in gene regions) were calculated from overlapped LD blocks and unlinked SNPs/InDels, by applying the ProxyGeneLD (72) method. For CNVs, the gene-level P values were assigned from the most significant P values of its overlapped CNV regions. Significantly associated genes were further identified using the significance cut-offs described in **Figure SN12**.

**
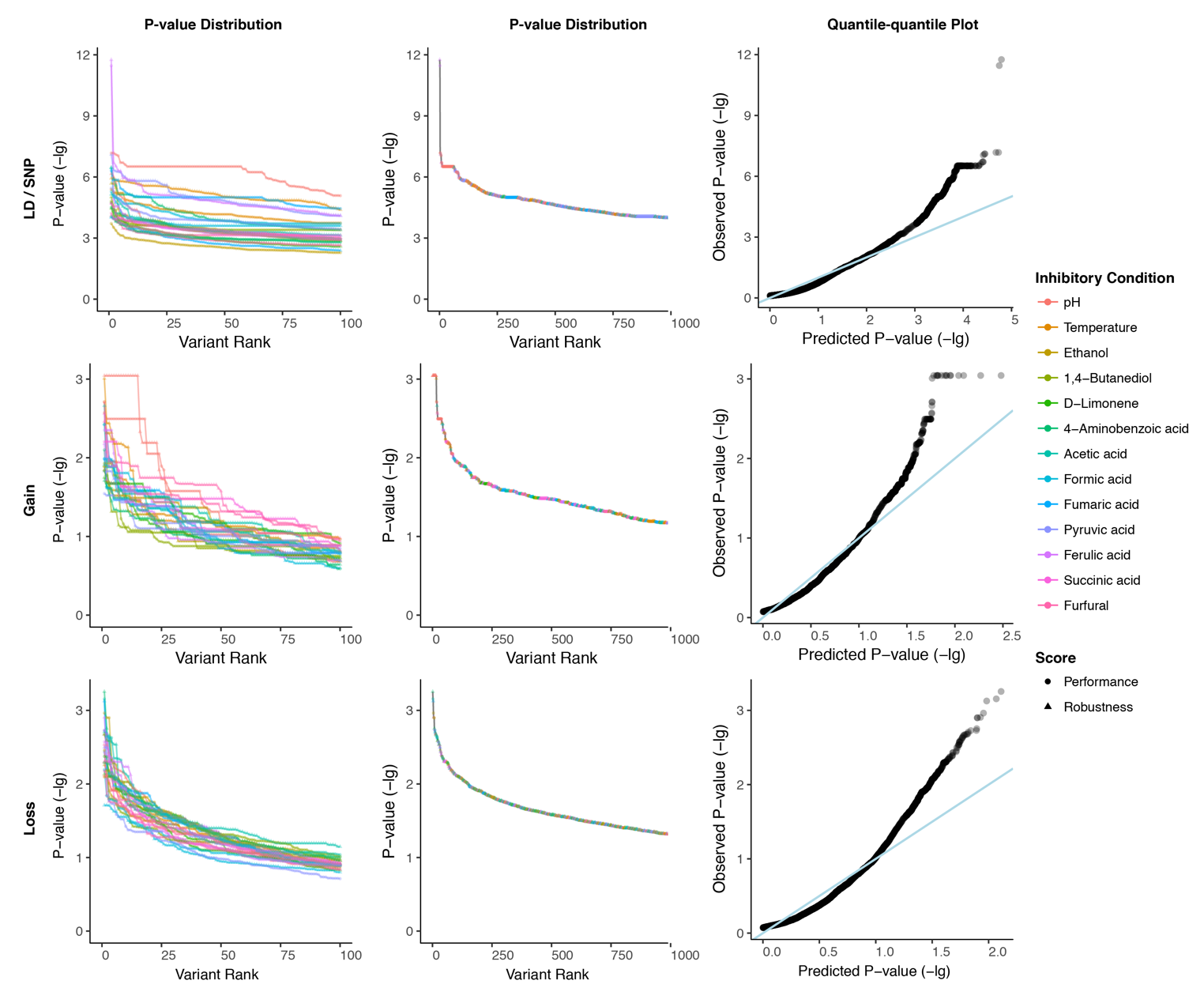
**

**Figure SN12.** **The *P* value distribution of the GWAS results.** The *P* value distributions for individual score rankings, the *P* value distribution for all score rankings, and the quantile-quantile plot for the predicted and observed –lg (*P* value), were illustrated for LDs or non-LD SNPs/InDels, gain events and loss events respectively. Significance cutoffs were set respectively according to the departures of observed *P* values (z (–lg (*P* value observed) – z (–lg (*P* value predicted) > 0.5) as: *Z*_LDs/SNPs/InDels_ = 3.608, *Z*_gain events_ = 1.632, *Z*_loss events_ = 1.342.

**Calculation of the relative contribution of SNP/gain/loss events in GWAS**

For the GWAS profile of each physiological score ranking, a 3,955 × 3 matrix *G* was generated, where the rows represent 3,955 genes used in GWAS studies, and the 3 columns represent 3 GWAS profiles: SNP, gain event and loss event, respectively. For the *P* value of each gene *i* in GWAS profile *j* (P*_i, j_*):

$G_{i,j}= -\lg P_{i,j}$ , or $G_{i,j}= 0$ if gene *i* was not present in the gene list of GWAS profile *j*

To eliminate the differences in the statistical power between SNP-based and CNV-based GWAS profiles, we defined a normalized matrix *G’*, where:

$${G'}_{i,j}=\frac{G_{i,j} \times\bar{G_{j}}}{\bar{G_{SNP}}}$$

For a gene set *k* with *m* genes (for the 75% quantile gene set, choose the top *m* genes with –lgP in the top 25% quantile from 3 GWAS profiles respectively), the SNP / CNV relative contribution *RC_SNP/CNV_* was calculated as:

$${RC}_{SNP/CNV}= \frac{\sum_{i}^{k} {(G'}_{i,SNP}-\frac{{G^{'}}_{i,Gain}+{G^{'}}_{i,Loss}}{2})}{m}$$

The Gain / Loss relative contribution *RC_Gain/Loss_* was calculated as:

$${RC}_{Gain/Loss}= \frac{\sum_{i}^{k} ({G^{'}}_{i,Gain}-{G^{'}}_{i,Loss})}{m}$$

The statistical power *PW* (illustrated by the point size in **Figure 6**) was calculated as:

$$PW= \frac{\sum_{i}^{k} {(G'}_{i,SNP}+\frac{{G^{'}}_{i,Gain}+{G^{'}}_{i,Loss}}{2})}{2m}$$

**The SGD gene lists that were used to be compared with the GWAS profiles**

The SGD phenotype associated gene lists were generated according to relevant stress conditions. For each stress condition, the selected SGD phenotypes are:

Temperature: Temperature sensitivity, Heat sensitivity, Innate thermotolerance, Acquired thermotolerance.

1,4-Butanediol (diol) (few entries): Resistance to chemicals (chemical: butan1ol, butan2ol, isobutanol).

Ethanol: Resistance to chemicals (chemical: ethanol).

pH: Acid pH resistance.

Acetic acid: Resistance to chemicals (chemical: acetate, acetic acid), Acid pH resistance (chemical: acetate, acetic acid).

Ferulic acid (few entries): Resistance to chemicals (chemical: ferulic acid).

Furfural (few entries): Resistance to chemicals (chemical: furfural).

All acids: Resistance to chemicals (chemical: all acids).

All conditions except pH and Temperature: Resistance to chemicals.

All conditions: All entries above, Viability (huge gene set), Competitive fitness (huge gene set), Vegetative growth (huge gene set), Growth in exponential phase, Survival rate in stationary phase, Lifespan, Replicative lifespan, Chronological lifespan, Stress resistance.

**The discovery of the PPI modules from SGD gene list and GWAS profile**

The PPI network of *S. cerevisiae* was acquired from the STRING [13] database. For each SGD gene list that mentioned above, regulatory modules were computed by ModuleDiscoverer [14] (module discovery *P* value cut-off set to 0.001). Meanwhile, the modules from the GWAS profile were computed in parallel. The gene *P* values from the GWAS profiles with SNP, gain and loss events were rescaled to *Z* scores respectively. A unique ranked gene list was generated by selecting the highest *Z* score for each gene from its SNP, gain and loss events. Top N genes were selected to compute the GWAS-based modules in PPIN, where N is the length of the corresponding SGD gene list. Two networks from SGD genes and GWAS genes were merged for illustration and analysis. The modules with most genes from only the GWAS gene list but not SGD gene list, were considered as GWAS-specific modules.

**SUPPLEMENTAL REFERENCES**

1. da Silva-Filho EA, Brito dos Santos SK, Resende Ado M, de Morais JO, de Morais MA, Jr. and Ardaillon Simoes D. Yeast population dynamics of industrial fuel-ethanol fermentation process assessed by PCR-fingerprinting. Antonie Van Leeuwenhoek. 2005;88 1:13-23. doi:10.1007/s10482-004-7283-8.

2. Verduyn C, Postma E, Scheffers WA and Van Dijken JP. Effect of benzoic acid on metabolic fluxes in yeasts: a continuous-culture study on the regulation of respiration and alcoholic fermentation. Yeast. 1992;8 7:501-17. doi:10.1002/yea.320080703.

3. Smart KF, Aggio RBM, Van Houtte JR and Villas-Bôas SG. Analytical platform for metabolome analysis of microbial cells using methyl chloroformate derivatization followed by gas chromatography-mass spectrometry. Nature Protocols. 2010;5 10:1709-29.

4. Jewison T, Knox C, Neveu V, Djoumbou Y, Guo AC, Lee J, et al. YMDB: the Yeast Metabolome Database. Nucleic Acids Res. 2012;40 Database issue:D815-20. doi:10.1093/nar/gkr916.

5. van den Berg RA, Hoefsloot HC, Westerhuis JA, Smilde AK and van der Werf MJ. Centering, scaling, and transformations: improving the biological information content of metabolomics data. BMC Genomics. 2006;7:142. doi:10.1186/1471-2164-7-142.

6. Mo ML, Palsson BO and Herrgard MJ. Connecting extracellular metabolomic measurements to intracellular flux states in yeast. Bmc Syst Biol. 2009;3:37. doi:10.1186/1752-0509-3-37.

7. UniProt C. The Universal Protein Resource (UniProt) in 2010. Nucleic acids research. 2010;38 Database issue:D142-8. doi:10.1093/nar/gkp846.

8. Moretti S, Martin O, Van Du Tran T, Bridge A, Morgat A and Pagni M. MetaNetX/MNXref--reconciliation of metabolites and biochemical reactions to bring together genome-scale metabolic networks. Nucleic acids research. 2016;44 D1:D523-6. doi:10.1093/nar/gkv1117.

9. Oh J, Byrd AL, Deming C, Conlan S, Barnabas B, Blakesley R, et al. Biogeography and individuality shape function in the human skin metagenome. Nature. 2014;514 7520:59-64. doi:10.1038/nature13786.

10. Edgar RC. MUSCLE: multiple sequence alignment with high accuracy and high throughput. Nucleic acids research. 2004;32 5:1792-7. doi:10.1093/nar/gkh340.

11. Langmead B and Salzberg SL. Fast gapped-read alignment with Bowtie 2. Nat Methods. 2012;9 4:357-9. doi:10.1038/nmeth.1923.

12. Barrett JC, Fry B, Maller J and Daly MJ. Haploview: analysis and visualization of LD and haplotype maps. Bioinformatics. 2005;21 2:263-5. doi:10.1093/bioinformatics/bth457.

13. Jensen LJ, Kuhn M, Stark M, Chaffron S, Creevey C, Muller J, et al. STRING 8--a global view on proteins and their functional interactions in 630 organisms. Nucleic acids research. 2009;37 Database issue:D412-6. doi:10.1093/nar/gkn760.

14. Vlaic S, Conrad T, Tokarski-Schnelle C, Gustafsson M, Dahmen U, Guthke R, et al. ModuleDiscoverer: Identification of regulatory modules in protein-protein interaction networks. Sci Rep. 2018;8 1:433. doi:10.1038/s41598-017-18370-2.
