# Supplementary material for: Linking genetic, metabolic, and phenotypic diversity among Saccharomyces cerevisiae strains using multi-omics associations
Source: Gigascience. 2019 Jan 31;8(4):giz015. doi: 10.1093/gigascience/giz015 (PMC6446221; doi:10.1093/gigascience/giz015)
Supplement: Response_to_Reviewer_Comments_Original_Submission.pdf [file giz015_response_to_reviewer_comments_original_submission.pdf]

Author's Response To Reviewer Comments

Close

ear Dr. Nicole Nogoy,

Thank you very much for the opportunity to revise our manuscript, “Linking genetic, metabolic and phenotypic diversity among S. cerevisiae strains using multi-omics associations” for publication in GigaScience. We are pleased to submit the revised version, in which we have addressed all suggestions of the three reviewers, and a point-to-point response to their comments could be found below. We have also adjusted some of our author’s name and order, please be noticed.

We are looking forward to hearing from you and we are hoping a positive evaluation.

Sincerely,

Gianni Panagiotou, PhD

Respond to Editor’s note:
Overall, a better description of the data analysis of metabolome and fluxome is required and reviewer #2 highlighted that some important procedures are missing for others to replicate the experiment. We strongly suggest you add your methods into Protocols.io - and the Protocols.io DOI cited in them manuscript. Note that our journal has a strong focus on reproducible research and we require such information is added for reviewers to adequately replicate your work. Furthermore, the quality of your results is not sufficiently discussed and needs to be improved to convince the reviewers.
We have made all suggested revisions to better describe the metabolome and fluxome analysis and prepared point-to-point responses to all reviewers’ questions. Required raw data (Dataset S7) and scripts are all available now. However, the submission systems automatically unzip all zip files. We alternatively uploaded Datasets S4 & S7 into the ftp folder.
Please also ensure that the genomic data associated with the SRA accession number is accessible, and add the MetaboLights accession number in the paper under "Availability of Supporting Data".
We have prepared the standard MetaboLights submission, with accession number: MTBLS780. The public date was set to 28/12/2018, which is the most recent date we can choose, as the administrators may manually curate the data. And our SRA submission is open to public now (SRR6114130 to SRR6114127).
In addition, please register any new software application in the SciCrunch.org database to receive a RRID (Research Resource Identification Initiative ID) number, and include this in your manuscript. This will facilitate tracking, reproducibility and re-use of your tool.
We have uploaded two self-developed pipelines to Github and SciCrunch: the metabolomic data processing pipeline, Metab 1.0 (https://github.com/reinkk/Metab, SCR_016877) and the growth curve processing pipeline, Growth-Profiling-Toolbox 1.0 (https://github.com/reinkk/Growth-Profiling-Toolbox, SCR_016878)

Answers to reviewers:

Reviewer #1:
In this manuscript, the authors did large-scale comparative genomics studies on various yeast species. Based on the growth phenotypes in the presence of different inhibitors or harsh industrial conditions, the authors tried to find the genetic determinants of several industrially desirable phenotypes. Although the topic covered in this manuscript is important for industrial biotechnology, unfortunately, the reviewer is not convinced that the authors have achieved what they claimed. Following please find a few suggestions to improve this manuscript.
We would like to thank this reviewer for his/her comments.
1. The stress response is precisely controlled by the regulatory networks, which means that transcriptomics

may be more reliable than genomics studies for the purpose of this study.
Thank you for your suggestion. Transcriptomics would be definitely expand the value of such a multi-omic study. The current study has focused more on the identification of industrial phenotype associated genotypes, thus ,we believe ultra-depth sequencing, in particular covering the genomic variants affecting transcription factor and non-coding regions such as promoters, are in higher priority. To fill the gap in the RNA-level, we performed sufficient work in GWAS followed by protein-protein interaction networks, which has successfully associated the industrial phenotypes with regulatory networks.
2. The most important data that the reviewer expect to have is the list of genes associated with the desirable phenotypes. Unfortunately, it is not provided in this manuscript.
Due to the length limitation, the regarding gene lists were summarized in Dataset S6 but not listed in the main text. We have inserted more clear quotations in the main text to guide the reader.
3. All bioinformatics derived data should be verified. Otherwise, such data can be wrong or misleading and may be useless for practical biotechnology applications.
Thank you for your comment. The pipelines we used for the bioinformatic analyses are the state-of-the-art used for population genomics (e.g., BWA for mapping, GATK for SNP/InDel calling, FreeC for CNV calling, EMMA for GWAS) or specifically developed for S. cerevisiae (YGAP gene annotation pipeline). The tools and pipelines are also in line with the ones applied in recent publications in the most prestigious journals of the field of yeast population genomics (Liti et al., 2009a; Peter et al., 2018). Thus we are confident that our results are technically reliable and reproducible. Furthermore, our study is a systems level analysis linking genotypes with phenotypes therefore we focused on confirming holistically the accuracy of our findings using extensively public available phenotypic databases (such as SGD) and other bioinformatics approaches (PPI networks). Verifying one association experimentally will not in our opinion add to our study, which serves as a very important source to experimental groups for future metabolic engineering endeavours.

Reviewer #2:
The manuscript titled "Linking genetic, metabolic and phenotypic diversity among S.cerevisiae strains using multi-omics association" details the characteristics of various budding yeast strains with multi omics. Authors analyzed phenotypes, metabolome, fluxome and genome of the strains. They demonstrated the possible strategy of strain selection and improvement.
This study includes several useful information, such as widely analyzed stress tolerance and genome-wide association study. However, manuscript of several part should be improved. Especially the description of data analysis of metabolome and fluxome is missing some important procedures for others to replicate the experiment. And quality of result is not sufficiently discussed.
We would like to thank this reviewer for his/her positive comments.
Major point is below.
Line 86-88.
Maximum OD is also important parameter for industrial usage. Please, consider including the parameter in your study. At least, include the maximum OD of each strain under the general condition in this study.
Thank you for this valuable comment. The total number of generations was chosen as the measure instead of the final OD as it also takes into account the starting OD which could vary among different experiments, in which the cells were under extreme stress. When coming to industrial production, considering that the initial OD would be stable, then the parameter "number of generations" could be definitely replaced by final OD. Some selected cases were also compared with the conventional procedure which showed that the two methods actually correspond quite well. Actually, we now shared our pipeline in Matlab on Github (https://github.com/reinkk/Growth-Profiling-Toolbox) and SciCrunch (SCR_016878), which allows user to modify some simple codes to use max OD as a parameter to replace "number of generations.
Line 142-151.
The part of Parameter Influence Analysis makes me confusing. I could not understand why it is discussed here. What the advantage of this analysis. For example, we all realize that lag phase and average growth rate are less important for continuous fermentation without the analysis.
Since we know before the analysis that lag phase and average growth rate are less important for continuous fermentation, and that is the intended use of the strain, we do not want to select a strain which ranks high due to these parameters. Therefore, PIA is introduced as a tool to understand in depth which are the parameters driving the phenotypic differences. The main advantage of PIA is that it can distinguish strains with similar rank (when giving equal weight to all five growth parameters) from each other and thereby assist in selecting the strain most suited for a specific application, and therefore allows researchers to adjust parameters' weights for different industrial purposes. When selecting a platform strain for a

continuous fermentation process, low weight could be given to parameters like “lag phase”. In contrast in a batch fermentation, “lag phase” becomes a determinant parameter which can influence the fermentation cycle dramatically; in that case a higher weight could be given to “lag phase”. To make this clearer, we have modified related descriptions in the main text as: “Such information can be useful when selecting a host strain for specific process by setting different weights to different growth parameters, e.g. lag phase would be less important selection criteria for a continuous fermentation process and subsequently be given a low weight, however, in a batch fermentation process, lag phase duration becomes critical and could be assigned with a higher weight”.

Line 154-156.

Show the score plot of principal component analysis of metabolome data as supplemental figure to show the data quality of biological and technical replicates. "intracellular" in Dataset S2 can be applied to directly to PCA. Unit variance is probably enough for preprocessing. Also, show the PCA of "extracellular" data.

Thank you for this excellent suggestion. We have applied such analyses and place the new figures in Supplementary Note S3 as Figures SN 6 & 7, under a new section named “Metabolite profile similarity by Principal Component Analysis (PCA) analysis”.

Line 240-254.

Please discuss the reliability of the fluxes calculated in this study.

We cited a recent paper used similar simulation technology showing that, strain-specific models can simulate differences in fluxes and give more biological insights than comparing metabolic reactions. So we added “Due to a recent study (Monk et al., 2016), inter-strain differences could be observed in key metabolic fluxes simulated by strain-specific models, which could provide more meaningful biological insights than the reaction presence/absence comparisons.”.

Line 251-254.

Comparison of flux of limiting steps is probably more suitable to compare the flux of pathways instead using the summed fluxes.

We fully agree. So we also presented the fluxes of key limited steps (e.g., Pyruvate Mitochondrial Transport) before the summed fluxes. However, the pathways we listed here, e.g., Citric Acid Cycle and Pyruvate Metabolism, have non-linear complex network structures which are consisted of multiple parallel reactions. Thus we think the overall flux description may give a different view from the key limiting step.

Line 257-316.

Are the sample number and sequence depth enough to perform SNP-based and CNV based GWAS?

The genomes were sequenced at a minimal depth of 52X, which is sufficient for SNP and CNV calling and GWAS analysis. Previous studies have also conducted GWAS on a strain cluster with similar number (35 ~ 38) of yeast strains (Connelly et al., 2013; Liti et al., 2009a; Warringer et al., 2011). Also, there is systematic evaluation of GWAS on S. cerevisiae strains of such sample size (Diao and Chen, 2012), suggesting that biologically meaningful results could be observed. Also, our PPIN analysis assisted dramatically in the reduction of false-positive noises in the GWAS analysis.

Figure 1.

In this study, growth phenotypes are mostly discussed as relative values. However absolute values are also important. Especially the variance of the phenotypic scores under each stress. Please show them as supplemental figures. Box plots may be suitable.

Thank you for this suggestion. All raw parameter scores were summarized in Dataset S1. However, considering our data size, we have 36 strains, 13 conditions with 4~7 levels and five parameter scores for each condition. To have sufficient resolution, in total there should be 36 x 13 x 5 = 2,340 box plot panels with 5~7 columns each. So we did not prepare all the figures for raw parameter scores but listed them in tables.

Figure 3A. and line 368-375 of supplemental items.

The preprocessing and transformation of HCA is not sufficiently described. Please describe in detail. Because data of MS is relative abundance, this process is quite important. And, what the "scaled to the biological range" mean?

The definition of “the biological range” was introduced by a previous literature in the metabolomic data normalization (van den Berg et al., 2006). We now added this citation and modified our description in detail under the section “Compound class and pathway assignment” as: “Metabolite data was transformed before calculating the contribution to each group of pathways and/or metabolite classes. Both intracellular and extracellular metabolites were scaled to the biological range [5]. Intracellular metabolite data was transformed by subtracting the mean of the data set, followed by division with the difference between the maximum and minimum value of the particular metabolite. This difference is referred to as the biological

range and makes all metabolites equally important [5]. Extracellular metabolites were first centered by the starting amount prior to scaling to the biological range, and were grouped according to compound classes as well as if they were produced (scaled value > 0) or consumed (< 0). Metabolites belonging to one of the selected classes and had positive or negative values were added together after scaling, respectively, to show production and consumption. For both intracellular and extracellular metabolites, scaled values of metabolites belonging to a pathway or compound class were added together for each strain to create group variables.The sum was subsequently divided by the square root of the number of metabolites to be comparable with the other classes. Hierarchical clustering was applied to the group variables using Euclidian distance between the strains."

Figure 3A.

How the compound class determined? And, what do metabolites belong to the classes?

To describe this, a new section names "Compound class and pathway assignment" was inserted to Supplemental Note S3: "Compounds identified were assigned to different classes and pathways by the pathway information in YMDB (Yeast Metabolic Database) (Jewison et al., 2012). These pathway and compound class assignment were summarized in Dataset S2.".

Line 309-325 of supplemental items.

I think the metabolome data of ethanol is not mentioned in this study.

Thank you for your correction. We did not use the ethanol media so we have removed all regarding descriptions in Supplemental Note S3 now.

Line 379-380 of supplemental items.

In Ref.4 you mentioned, the level of extracellular metabolites used. Your study also uses them? If so, please add the data used to calculate the flux as supplemental material.

Ref. 4 refers the starting model of yeast we used for the strain-specific models, which did not actually used extra metabolomic data. The metabolomics data used for our models are already included in Dataset S2. And the regarding raw data are now available on MetaboLights with accession number: MTBLS780. The public date was set to 28/12/2018, which is the most recent date we can choose.

Minor point

Line 97-115.

The stress responses are sometimes affected by growth condition such as aeration condition and medium composition. I recommend describing the conditions briefly in body text, and if possible, discuss about it in discussion.

Thank you for this suggestion. The growth medium used in the current study is a standard defined mineral medium extensively used in yeast studies. Aeration was the same in all conditions for phenotypic characterization by using the Growth Profiler. As the size of the study was already considered quite large, it was actually not within the scope of the study to include variation in the growth medium nor the aeration conditions. But sure, expanded studies with altered aeration condition or medium composition will be interesting, following our technical route. Now we added the following statement in the main text: "Besides parameter selection and weighting, our phenotypic scoring method could also be expanded to different industrially relevant processes with customized set-ups: e.g., different medium composition and aeration condition, which is not limited to the standard medium and condition used in this proof-of-concept study."

line 154 -156.

Please show the level of compound identification based on Metabolomics Standards Initiative (see table1 of Metabolites2018,8,31)

We have prepared the standard MetaboLights submission, with accession number: MTBLS780. The public date was set to 28/12/2018, which is the most recent date we can choose. And we also included an MAF table (Metabolite Assignment File) in Dataset S2, so readers can find ChEBI ids for those metabolites.

Line 383-423

If possible, compare the genes you selected with previous study of single gene deletion collection, such as the study below.

(FEMS Yeast Research, Volume 9, Issue 1, 1 February 2009, Pages 32-44)

We do observe genes mentioned in the literature to appear as significant in our analysis, thus we added the following sentence -as such an example- in our discussion: "SKY1 was also a significant gene shared by 4 conditions, which has been proved to be associated with osmotic tolerance (Yoshikawa et al., 2009)".

Figure 3A. In HCA what type of clustering method is used?

We added the following statement to the figure 3 legend: "Hierarchical clustering was applied to the group variables using Euclidian distance between the strains. For data normalization before visualization, see details in Supplemental Note S3."

Figure 3C.
Please add the unit of vertical axis.
The unit is mmol/gDW/h and we have now added it to the figure.

Reviewer #3:
In this study, Kang et al. performed whole genome sequencing, characterized growth under 13 industrially relevant conditions, performed metabolomics and GWAS analyses on 36 S. cerevisiae strains to identify potentially important genes implicated in stress tolerance/ resistance. The rationale for this study is well-defined. While other studies that utilize natural genetic variation between strains have a larger collection of strains across a wide geographic and phylogenetic distribution (e.g. 165 strains in Sardi M et al 2018, PMC5849340), Kang et al. utilizes a multi-OMIC approach to characterize these 36 strains deeply. Additionally, these authors performed a detailed growth characterization of these 36 strains, with thoughtful considerations outlined in the Parameter Influence Analysis. Overall, the paper seems appropriate for GigaScience given its editorial guidelines. Below, I have a few comments that can be addressed with minor revisions to the manuscript.
We would like to thank this reviewer for his/her positive comments.
Major points:
1. Given the importance of the growth data that defines strain robustness and performance, where will the raw growth data underlying the 5400 growth curves along with the in-house MatLab scripts deposited?
We have uploaded two self-developed pipelines to Github and SciCrunch: the metabolomic data processing pipeline, Metab 1.0 (https://github.com/reinkk/Metab, SCR_016877) and the growth curve processing pipeline, Growth-Profiling-Toolbox 1.0 (https://github.com/reinkk/Growth-Profiling-Toolbox, SCR_016878). And the raw OD data were also included as Dataset S7. The software repositories were now mentioned in the manuscript as “Raw data for growth curves in G-values (Dataset S7) were analyzed automatically using self-developed Matlab® scripts: Growth Profiling Toolbox (GitHub repository: https://github.com/reinkk/Growth-Profiling-Toolbox, with SciCrunch ID SCR_016878).”.
2. Along the same line of inquiries, I was not able to access the genomic data associated with the SRA accession number that was provided in the manuscript.
We now made our submission public.
3. Furthermore, while I am not a metabolomics expert, I know that the metabolomics field has developed Metabolomics Standards Initiative to improve reproducibility. It is not clear whether the authors follow this guideline. There were also no accession number associated with the metabolomics data. Have the authors deposited the data to databases such as MetaboLights?
We have prepared the standard MetaboLights submission, with accession number: MTBLS780. The public date was set to 28/12/2018, which is the most recent date we can choose. And we also included an MAF table (Metabolite Assignment File) in Dataset S2, so readers can find ChEBI ids for those metabolites.
4. The use of base modifications (BMs) to describe genetic variations such as SNPs or INDELs is inappropriate. In general, base modification is used to describe methylation, and as far as I could gather from the methods section, the authors did not perform epigenetic analyses or PacBio sequencing.
We modified it as “SNPs/InDels” throughout the text.
5. Since accurate SNP calling is important for the GWAS, I have a few comments and questions about the choice of these filters in GATK:
a. It's worthwhile to explicit state the filtering strategy (I did not see it in the supplemental file) and had to dig it out by looking up the reference to a previous paper: pubmed/26255308
b. Is there a reason why the authors chose to use UnifiedGenotyper as GATK has deprecated this tool due to this caller being very "aggressive in calling variants in order to be very sensitive"? The current recommendation by GATK is to use HaplotypeCaller that reduces false positives, especially on INDELs.
c. This is related to the concerns of false positive SNPs, where the authors use VariantFiltration (parameters:-filterExpression 'ReadPosRankSum < −8.0 || FS > 10.0'). What is the rationale for lowering the FisherStrand (FS) value below recommendations by GATK? According to GATK, most of the variants that fail have an FS value greater than 55, hence the recommendation of failing variants with an FS value less than 60.
We are sorry that we have made a mistake here and thank you so much for your correction. We checked our script and we indeed used a new pipeline since GATK suggested HaplotypeCaller after 2014, which is different with the cited paper when data were processed before HaplotypeCaller becoming a golden standard. The section has been rewritten now as: “SNPs/InDels were called and filtered using the Genome Analysis Toolkit (GATK) 3.5 (DePristo et al., 2011; McKenna et al., 2010), with the sequential steps to

include RealignerTargetCreator, IndelRealigner, HaplotypeCaller (with parameter "-rf BadCigar") and VariantFiltration (with parameter "--filterExpression "DP < 10 || QD < 2.0 || FS > 60.0"")".
d. The authors cite Strope PK et al (2015) as a support that the numbers of genetic variants they identified in this study is comparable to previous studies (p9, lines 178-180). However, Strope PK observed an average of 78,184 SNPs and 7840 INDELs from 100 genomes, compared to the current study of 342,325 SNPs and 19,347 INDELs. Given the relaxed filter used for variant calling and the 2-4X more variants identified, I think it's important to make sure that these variants are not false positives.
S. cerevisiae strains are quite divergent in the genome. The reason why Strope PK et al (2015) has a lower number of SNPs/InDels is that the 100 strains they selected are extremely close to the reference strain S288c, and 84 of the 100 strains are exactly in the same clade with S288c in the phylogenetic tree and were called "S288c type". Also, their number indicates the SNP count per strain, while our 342,325 are all SNPs loci identified in the whole strain collection. For each strain, we have 68,928 SNPs/InDels in average, which is quite close to Strope PK et. al (78,184 + 7,840, as they did not applied the strict filtering as we did). Now we cited a more comparable study (similar strain collection but with lower sequencing depth) where they have 235,127 SNPs (Liti et al., 2009b) and modified our description to "By using S288c as the reference genome, we identified 342,325 SNP loci, 19,347 small insertion and 17,457 small deletion (InDel) loci among all strains (Table S1 & Dataset S3), with an average SNP/InDel count of 68,928 per strain".
Furthermore, as described in our manuscript, we applied MAF > 5% (at least 4 alleles in our case) before LD block building and GWAS. Also, we selected only core-genome SNPs/InDels for GWAS, which resulted in only 165,358 loci left. We believe these SNPs/InDels are confident for down-stream GWAS.
6. What is the rationale for choosing a low MAF filtration (MAF > 0.5%) for the core-genome GWAS (pg 34, line 483)? What does the MAF distribution look like for these identified core-genome SNPs? In general, the GWAS studies in yeast tend to use MAF > 2% - 25%, as the power to detect association with rare variation is limited.
Sorry for this typo in Note S5 and now we corrected it. Actually we used MAF > 0.05, which is 5% but not 0.5%. We now modified all sections mentioning MAF to the format "5%" to avoid misunderstanding.
Minor points:
1. In Supplemental Note S2, the authors describe how they extract growth parameters of interest in great details. Could the authors add in the rationale for calculating the average growth rate using a time point between 50 to 501 time points (pg 14, line 165)? More specifically, does this time point capture the linear range of the growth curve and time point 50 is after the lag phase of even the most stressed cells?
The time point was selected during exponential phase. So for the most stressed cell, the time point was much later than the cells under less stress. For the least stressed cells, the time point is close to 50, otherwise that would be a much later time point. We added "to ensure the time point was in exponential phase" in the relevant description.
2. In Supplemental Dataset S1, the authors need to define the color coding for parameter influence.
We added "P1~P5 stand for: 1. Lag phase; 2. Growth duration; 3. Number of generations during the growth phase; 4. Average specific growth rate during the growth phase; 5. Maximum specific growth rate." to the figure legend.
3. In general, the figures are aesthetically pleasing and well designed. However, it is important to keep in mind of color blindness (e.g. Fig 2A uses red and green colours to denote the strains)
Thank you for your suggestion. We admit that we have not thought about colour blindness and we will pay more attention in the future.
4. Need to properly cite R as per CRAN's guideline https://cran.r-project.org/doc/FAQ/R-FAQ.html#Citing-R
Thank you for pointing this out, the R Core Team has been cited now.

References

Connelly, C.F., Skelly, D.A., Dunham, M.J., and Akey, J.M. (2013). Population Genomics and Transcriptional Consequences of Regulatory Motif Variation in Globally Diverse Saccharomyces cerevisiae Strains. Molecular Biology and Evolution 30, 1605-1613.
DePristo, M.A., Banks, E., Poplin, R., Garimella, K.V., Maguire, J.R., Hartl, C., Philippakis, A.A., del Angel, G., Rivas, M.A., Hanna, M., et al. (2011). A framework for variation discovery and genotyping using next-generation DNA sequencing data. Nature genetics 43, 491-498.
Diao, L., and Chen, K.C. (2012). Local ancestry corrects for population structure in Saccharomyces cerevisiae genome-wide association studies. Genetics 192, 1503-1511.

Jewison, T., Knox, C., Neveu, V., Djoumbou, Y., Guo, A.C., Lee, J., Liu, P., Mandal, R., Krishnamurthy, R., Sinelnikov, I., et al. (2012). YMDB: the Yeast Metabolome Database. Nucleic Acids Res 40, D815-820.
Liti, G., Carter, D.M., Moses, A.M., Warringer, J., Parts, L., James, S.A., Davey, R.P., Roberts, I.N., Burt, A., Koufopanou, V., et al. (2009a). Population genomics of domestic and wild yeasts. Nature 458, 337-341.
Liti, G., Carter, D.M., Moses, A.M., Warringer, J., Parts, L., James, S.A., Davey, R.P., Roberts, I.N., Burt, A., Koufopanou, V., et al. (2009b). Population genomics of domestic and wild yeasts. Nature 458, 337-341.
McKenna, A., Hanna, M., Banks, E., Sivachenko, A., Cibulskis, K., Kernytsky, A., Garimella, K., Altshuler, D., Gabriel, S., Daly, M., et al. (2010). The Genome Analysis Toolkit: a MapReduce framework for analyzing next-generation DNA sequencing data. Genome research 20, 1297-1303.
Monk, J.M., Koza, A., Campodonico, M.A., Machado, D., Seoane, J.M., Palsson, B.O., Herrgard, M.J., and Feist, A.M. (2016). Multi-omics Quantification of Species Variation of Escherichia coli Links Molecular Features with Strain Phenotypes. Cell Syst 3, 238-251 e212.
Peter, J., De Chiara, M., Friedrich, A., Yue, J.X., Pflieger, D., Bergstrom, A., Sigwalt, A., Barre, B., Freel, K., Llored, A., et al. (2018). Genome evolution across 1,011 Saccharomyces cerevisiae isolates. Nature 556, 339-344.
van den Berg, R.A., Hoefsloot, H.C., Westerhuis, J.A., Smilde, A.K., and van der Werf, M.J. (2006). Centering, scaling, and transformations: improving the biological information content of metabolomics data. BMC Genomics 7, 142.
Warringer, J., Zörgö, E., Cubillos, F.A., Zia, A., Gjuvsland, A., Simpson, J.T., Forsmark, A., Durbin, R., Omholt, S.W., Louis, E.J., et al. (2011). Trait Variation in Yeast Is Defined by Population History. PLoS Genetics 7, e1002111.
Yoshikawa, K., Tanaka, T., Furusawa, C., Nagahisa, K., Hirasawa, T., and Shimizu, H. (2009). Comprehensive phenotypic analysis for identification of genes affecting growth under ethanol stress in Saccharomyces cerevisiae. FEMS Yeast Res 9, 32-44.

Close
