# Supplementary material for: Linking genetic, metabolic, and phenotypic diversity among Saccharomyces cerevisiae strains using multi-omics associations
Source: Gigascience. 2019 Jan 31;8(4):giz015. doi: 10.1093/gigascience/giz015 (PMC6446221; doi:10.1093/gigascience/giz015)
Supplement: Response_to_Reviewer_Comments_Revision_1.pdf [file giz015_response_to_reviewer_comments_revision_1.pdf]

Author's Response To Reviewer Comments

Close

Reviewer reports:
Reviewer #1: The authors have done a nice job to address all the reviewers' concerns. I think this manuscript can be accepted for publication.

Thank you so much for your suggestions.

Reviewer #3: 1. The authors have added Dataset S7 of raw growth data and associated analysis scripts. However, this needs to be explicitly stated in the main manuscript and not just in the supplemental notes. Also, need to add a legend for Dataset S7 on page 30 of the current manuscript under the heading Supplemental Items.

We have now added a reference of Dataset S7 in the main text and the legend for Dataset S7.

2. Update the SRA accession number to the appropriate Bioproject - PRJNA412468, as the current SRR accession numbers provided (SRR6114130 to SRR6114127) only include 4 strains.

Sorry for this mistake and now we have corrected the IDs.

3. As reviewer #2 and I both pointed out, the metabolomics data need to be submitted to MetaboLights. I still cannot find MTBLS780 (as of Jan 9, 2019)

We have been in contact with MetaboLights regarding our submission. They said that our submission is now in manually curation which may take a week to process. We believe this process won't take too long so our submission could become public soon.

4. In the excel file containing Dataset S1, the colour coding in the table is still not defined. (e.g. does green correspond to P1 and purple correspond to P5?)

We have now filled those cells with actual data.

5. page 15, line 302, need to replace base modification to SNPs/INDELs

Thank you for your correction.

Close
