# Supplementary material for: Linking genetic, metabolic, and phenotypic diversity among Saccharomyces cerevisiae strains using multi-omics associations
Source: Gigascience. 2019 Jan 31;8(4):giz015. doi: 10.1093/gigascience/giz015 (PMC6446221; doi:10.1093/gigascience/giz015)
Supplement: Reviewer_2_Report_Original_Submission -- yasumune nakayama — 12/9/2018 Reviewed [file giz015_reviewer_2_report_original_submission.pdf]

Reviewer Report

Title: Linking genetic, metabolic and phenotypic diversity among S. cerevisiae strains using multi-omics associations

Version: Original Submission **Date:** 12/9/2018

Reviewer name: yasumune nakayama

Reviewer Comments to Author:

The manuscript titled "Linking genetic, metabolic and phenotypic diversity among S.cerevisiae strains using multi-omics association" details the characteristics of various budding yeast strains with multi omics. Authors analyzed phenotypes, metabolome, fluxome and genome of the strains. They demonstrated the possible strategy of strain selection and improvement.
This study includes several useful information, such as widely analyzed stress tolerance and genome-wide association study. However, manuscript of several part should be improved. Especially the description of data analysis of metabolome and fluxome is missing some important procedures for others to replicate the experiment. And quality of result is not sufficiently discussed.
Major point is below.
Line 86-88.
Maximum OD is also important parameter for industrial usage. Please, consider including the parameter in your study. At least, include the maximum OD of each strain under the general condition in this study.
Line 142-151.
The part of Parameter Influence Analysis makes me confusing. I could not understand why it is discussed here. What the advantage of this analysis. For example, we all realize that lag phase and average growth rate are less important for continuous fermentation without the analysis.
Line 154-156.
Show the score plot of principal component analysis of metabolome data as supplemental figure to show the data quality of biological and technical replicates. "intracellular" in Dataset S2 can be applied to directly to PCA. Unit variance is probably enough for preprocessing. Also, show the PCA of "extracellular" data.
Line 240-254.
Please discuss the reliability of the fluxes calculated in this study.
Line 251-254.
Comparison of flux of limiting steps is probably more suitable to compare the flux of pathways instead using the summed fluxes.
Line 257-316.
Are the sample number and sequence depth enough to perform SNP-based and CNV based GWAS?
Figure 1.
In this study, growth phenotypes are mostly discussed as relative values. However absolute values are also important. Especially the variance of the phenotypic scores under each stress. Please show them as supplemental figures. Box plots may be suitable.

Figure 3A. and line 368-375 of supplemental items.
The preprocessing and transformation of HCA is not sufficiently described. Please describe in detail. Because data of MS is relative abundance, this process is quite important. And, what the "scaled to the biological range" mean?
Figure 3A.
How the compound class determined? And, what do metabolites belong to the classes?
Line 309-325 of supplemental items.
I think the metabolome data of ethanol is not mentioned in this study.
Line 379-380 of supplemental items.
In Ref.4 you mentioned, the level of extracellular metabolites used. Your study also uses them? If so, please add the data used to calculate the flux as supplemental material.
Minor point
Line 97-115.
The stress responses are sometimes affected by growth condition such as aeration condition and medium composition. I recommend describing the conditions briefly in body text, and if possible, discuss about it in discussion.
line 154 -156.
Please show the level of compound identification based on Metabolomics Standards Initiative (see table1 of Metabolites2018,8,31)
Line 383-423
If possible, compare the genes you selected with previous study of single gene deletion collection, such as the study below.
(FEMS Yeast Research, Volume 9, Issue 1, 1 February 2009, Pages 32-44)
Figure 3A. In HCA what type of clustering method is used?
Figure 3C.
Please add the unit of vertical axis.

Methods

Are the methods appropriate to the aims of the study, are they well described, and are necessary controls included? Choose an item.

Conclusions

Are the conclusions adequately supported by the data shown? Choose an item.

Reporting Standards

Does the manuscript adhere to the journal's guidelines on minimum standards of reporting? Choose an item.

Choose an item.

Statistics

Are you able to assess all statistics in the manuscript, including the appropriateness of statistical tests used? Choose an item.

Quality of Written English

Please indicate the quality of language in the manuscript: Choose an item.

Declaration of Competing Interests

Please complete a declaration of competing interests, considering the following questions:

- Have you in the past five years received reimbursements, fees, funding, or salary from an organisation that may in any way gain or lose financially from the publication of this manuscript, either now or in the future?
- Do you hold any stocks or shares in an organisation that may in any way gain or lose financially from the publication of this manuscript, either now or in the future?
- Do you hold or are you currently applying for any patents relating to the content of the manuscript?
- Have you received reimbursements, fees, funding, or salary from an organization that holds or has applied for patents relating to the content of the manuscript?
- Do you have any other financial competing interests?
- Do you have any non-financial competing interests in relation to this paper?

If you can answer no to all of the above, write 'I declare that I have no competing interests' below. If your reply is yes to any, please give details below.

I declare that I have no competing interests

I agree to the open peer review policy of the journal. I understand that my name will be included on my report to the authors and, if the manuscript is accepted for publication, my named report including any attachments I upload will be posted on the website along with the authors' responses. I agree for my report to be made available under an Open Access Creative Commons CC-BY license (http://creativecommons.org/licenses/by/4.0/). I understand that any comments which I do not wish to be included in my named report can be included as confidential comments to the editors, which will not be published.

I agree to the open peer review policy of the journal

To further support our reviewers, we have joined with Publons, where you can gain additional credit to further highlight your hard work (see: https://publons.com/journal/530/gigascience). On publication of this paper, your review will be automatically added to Publons, you can then choose whether or not to claim your Publons credit. I understand this statement.

Yes Choose an item.
