# Supplementary material for: Linking genetic, metabolic, and phenotypic diversity among Saccharomyces cerevisiae strains using multi-omics associations
Source: Gigascience. 2019 Jan 31;8(4):giz015. doi: 10.1093/gigascience/giz015 (PMC6446221; doi:10.1093/gigascience/giz015)
Supplement: Reviewer_3_Report_Original_Submission -- Amy Lee — 12/17/2018 Reviewed [file giz015_reviewer_3_report_original_submission.pdf]

Reviewer Report

Title: Linking genetic, metabolic and phenotypic diversity among S. cerevisiae strains using multi-omics associations

Version: Original Submission **Date:** 12/17/2018

Reviewer name: Amy Lee

Reviewer Comments to Author:

In this study, Kang et al. performed whole genome sequencing, characterized growth under 13 industrially relevant conditions, performed metabolomics and GWAS analyses on 36 S. cerevisiae strains to identify potentially important genes implicated in stress tolerance/ resistance. The rationale for this study is well-defined. While other studies that utilize natural genetic variation between strains have a larger collection of strains across a wide geographic and phylogenetic distribution (e.g. 165 strains in Sardi M et al 2018, PMC5849340), Kang et al. utilizes a multi-OMIC approach to characterize these 36 strains deeply. Additionally, these authors performed a detailed growth characterization of these 36 strains, with thoughtful considerations outlined in the Parameter Influence Analysis. Overall, the paper seems appropriate for GigaScience given its editorial guidelines. Below, I have a few comments that can be addressed with minor revisions to the manuscript.
Major points:
1. Given the importance of the growth data that defines strain robustness and performance, where will the raw growth data underlying the 5400 growth curves along with the in-house MatLab scripts deposited?
2. Along the same line of inquiries, I was not able to access the genomic data associated with the SRA accession number that was provided in the manuscript.
3. Furthermore, while I am not a metabolomics expert, I know that the metabolomics field has developed Metabolomics Standards Initiative to improve reproducibility. It is not clear whether the authors follow this guideline. There were also no accession number associated with the metabolomics data. Have the authors deposited the data to databases such as MetaboLights?
4. The use of base modifications (BMs) to describe genetic variations such as SNPs or INDELs is inappropriate. In general, base modification is used to describe methylation, and as far as I could gather from the methods section, the authors did not perform epigenetic analyses or PacBio sequencing.
5. Since accurate SNP calling is important for the GWAS, I have a few comments and questions about the choice of these filters in GATK:
a. It's worthwhile to explicit state the filtering strategy (I did not see it in the supplemental file) and had to dig it out by looking up the reference to a previous paper: pubmed/26255308
b. Is there a reason why the authors chose to use UnifiedGenotyper as GATK has deprecated this tool due to this caller being very "aggressive in calling variants in order to be very sensitive"? The current recommendation by GATK is to use HaplotypeCaller that reduces false positives, especially on INDELs.
c. This is related to the concerns of false positive SNPs, where the authors use VariantFiltration (parameters:-filterExpression 'ReadPosRankSumâ€‰<â€‰âˆ’8.0 || FSâ€‰>â€‰10.0'). What is

the rationale for lowering the FisherStrand (FS) value below recommendations by GATK? According to GATK, most of the variants that fail have an FS value greater than 55, hence the recommendation of failing variants with an FS value less than 60.
d. The authors cite Strope PK et al (2015) as a support that the numbers of genetic variants they identified in this study is comparable to previous studies (p9, lines 178-180). However, Strope PK observed an average of 78,184 SNPs and 7840 INDELs from 100 genomes, compared to the current study of 342,325 SNPs and 19,347 INDELs. Given the relaxed filter used for variant calling and the 2-4X more variants identified, I think it's important to make sure that these variants are not false positives.
6. What is the rationale for choosing a low MAF filtration (MAF > 0.5%) for the core-genome GWAS (pg 34, line 483)? What does the MAF distribution look like for these identified core-genome SNPs? In general, the GWAS studies in yeast tend to use MAF > 2% - 25%, as the power to detect association with rare variation is limited.
Minor points:
1. In Supplemental Note S2, the authors describe how they extract growth parameters of interest in great details. Could the authors add in the rationale for calculating the average growth rate using a time point between 50 to 501 time points (pg 14, line 165)? More specifically, does this time point capture the linear range of the growth curve and time point 50 is after the lag phase of even the most stressed cells?
2. In Supplemental Dataset S1, the authors need to define the color coding for parameter influence.
3. In general, the figures are aesthetically pleasing and well designed. However, it is important to keep in mind of color blindness (e.g. Fig 2A uses red and green colours to denote the strains)
4. Need to properly cite R as per CRAN's guideline https://cran.r-project.org/doc/FAQ/R-FAQ.html#Citing-R

Methods

Are the methods appropriate to the aims of the study, are they well described, and are necessary controls included? Choose an item.

Conclusions

Are the conclusions adequately supported by the data shown? Choose an item.

Reporting Standards

Does the manuscript adhere to the journal's guidelines on minimum standards of reporting? Choose an item.

Choose an item.

Statistics

Are you able to assess all statistics in the manuscript, including the appropriateness of statistical tests used? Choose an item.

Quality of Written English

Please indicate the quality of language in the manuscript: Choose an item.

Declaration of Competing Interests

Please complete a declaration of competing interests, considering the following questions:

- Have you in the past five years received reimbursements, fees, funding, or salary from an organisation that may in any way gain or lose financially from the publication of this manuscript, either now or in the future?
- Do you hold any stocks or shares in an organisation that may in any way gain or lose financially from the publication of this manuscript, either now or in the future?
- Do you hold or are you currently applying for any patents relating to the content of the manuscript?
- Have you received reimbursements, fees, funding, or salary from an organization that holds or has applied for patents relating to the content of the manuscript?
- Do you have any other financial competing interests?
- Do you have any non-financial competing interests in relation to this paper?

If you can answer no to all of the above, write 'I declare that I have no competing interests' below. If your reply is yes to any, please give details below.

I declare that I have no competing interests

I agree to the open peer review policy of the journal. I understand that my name will be included on my report to the authors and, if the manuscript is accepted for publication, my named report including any attachments I upload will be posted on the website along with the authors' responses. I agree for my report to be made available under an Open Access Creative Commons CC-BY license (http://creativecommons.org/licenses/by/4.0/). I understand that any comments which I do not wish to be included in my named report can be included as confidential comments to the editors, which will not be published.

I agree to the open peer review policy of the journal

To further support our reviewers, we have joined with Publons, where you can gain additional credit to further highlight your hard work (see: https://publons.com/journal/530/gigascience). On publication of this paper, your review will be automatically added to Publons, you can then choose whether or not to claim your Publons credit. I understand this statement.

Yes Choose an item.
