# Supplementary material for: Linking genetic, metabolic, and phenotypic diversity among Saccharomyces cerevisiae strains using multi-omics associations
Source: Gigascience. 2019 Jan 31;8(4):giz015. doi: 10.1093/gigascience/giz015 (PMC6446221; doi:10.1093/gigascience/giz015)
Supplement: Reviewer_3_Report_Revision_1 -- Amy Lee — 1/9/2019 Reviewed [file giz015_reviewer_3_report_revision_1.pdf]

Reviewer Report

Title: Linking genetic, metabolic and phenotypic diversity among S. cerevisiae strains using multi-omics associations

Version: Revision 1 **Date:** 1/9/2019

Reviewer name: Amy Lee

Reviewer Comments to Author:

1. The authors have added Dataset S7 of raw growth data and associated analysis scripts. However, this needs to be explicitly stated in the main manuscript and not just in the supplemental notes. Also, need to add a legend for Dataset S7 on page 30 of the current manuscript under the heading Supplemental Items.
2. Update the SRA accession number to the appropriate Bioproject - PRJNA412468, as the current SRR accession numbers provided (SRR6114130 to SRR6114127) only include 4 strains.
3. As reviewer #2 and I both pointed out, the metabolomics data need to be submitted to MetaboLights. I still cannot find MTBLS780 (as of Jan 9, 2019)
4. In the excel file containing Dataset S1, the colour coding in the table is still not defined. (e.g. does green correspond to P1 and purple correspond to P5?)
5. page 15, line 302, need to replace base modification to SNPs/INDELs

Methods

Are the methods appropriate to the aims of the study, are they well described, and are necessary controls included? Choose an item.

Conclusions

Are the conclusions adequately supported by the data shown? Choose an item.

Reporting Standards

Does the manuscript adhere to the journal's guidelines on minimum standards of reporting? Choose an item.

Choose an item.

Statistics

Are you able to assess all statistics in the manuscript, including the appropriateness of statistical tests used? Choose an item.

Quality of Written English

Please indicate the quality of language in the manuscript: Choose an item.

Declaration of Competing Interests

Please complete a declaration of competing interests, considering the following questions:

- Have you in the past five years received reimbursements, fees, funding, or salary from an organisation that may in any way gain or lose financially from the publication of this manuscript, either now or in the future?
- Do you hold any stocks or shares in an organisation that may in any way gain or lose financially from the publication of this manuscript, either now or in the future?
- Do you hold or are you currently applying for any patents relating to the content of the manuscript?
- Have you received reimbursements, fees, funding, or salary from an organization that holds or has applied for patents relating to the content of the manuscript?
- Do you have any other financial competing interests?
- Do you have any non-financial competing interests in relation to this paper?

If you can answer no to all of the above, write 'I declare that I have no competing interests' below. If your reply is yes to any, please give details below.

No

I agree to the open peer review policy of the journal. I understand that my name will be included on my report to the authors and, if the manuscript is accepted for publication, my named report including any attachments I upload will be posted on the website along with the authors' responses. I agree for my report to be made available under an Open Access Creative Commons CC-BY license (http://creativecommons.org/licenses/by/4.0/). I understand that any comments which I do not wish to be included in my named report can be included as confidential comments to the editors, which will not be published.

I agree to the open peer review policy of the journal

To further support our reviewers, we have joined with Publons, where you can gain additional credit to further highlight your hard work (see: https://publons.com/journal/530/gigascience). On publication of this paper, your review will be automatically added to Publons, you can then choose whether or not to claim your Publons credit. I understand this statement.

Yes Choose an item.
